# Morphological and phylogenetic analyzes reveal two new species of *Melanconiella* from Fujian Province, China

**DOI:** 10.3389/fmicb.2023.1229705

**Published:** 2023-08-16

**Authors:** Taichang Mu, Jinhui Chen, Zhiying Zhao, Weibin Zhang, Steven L. Stephenson, Chenjie Yang, Mengjia Zhu, Hailan Su, Pu Liu, Xiayu Guan, Junzhi Qiu

**Affiliations:** ^1^Key Lab of Biopesticide and Chemical Biology, Ministry of Education, State Key Laboratory of Ecological Pest Control for Fujian and Taiwan Crops, College of Life Sciences, Fujian Agriculture and Forestry University, Fuzhou, Fujian, China; ^2^Department of Biological Sciences, University of Arkansas, Fayetteville, AR, United States; ^3^Agricultural BioResources Institute, Fujian Academy of Agricultural Sciences, Fuzhou, China; ^4^Engineering Research Center of Edible and Medicinal Fungi, Ministry of Education, Jilin Agricultural University, Changchun, China; ^5^College of Horticulture, Fujian Agriculture and Forestry University, Fuzhou, Fujian, China

**Keywords:** leaf disease, Melanconiellaceae, multi-gene phylogeny, new species, taxonomy

## Abstract

**Introduction:**

Species of *Melanconiella* include a diverse array of plant pathogens as well as endophytic fungi. Members of this genus have been frequently collected from the family Betulaceae (birches) in Europe and North America. Little, however, if known concerning the distribution of *Melanconiella* and/or their potential as pathogens of other plant hosts.

**Methods:**

Fungi were noted and isolated from diseased leaves of *Loropetalum chinense* (Chinese fringe flower) and *Camellia sinensis* (tea) in Fujian Province, China. Genomic DNA was extracted from fungal isolates and the nucleotide sequences of four loci were determined and sued to construct phylogenetic trees. Morphological characteristics of fungal structures were determined via microscopic analyses.

**Results:**

Four strains and two new species of *Melanconiella* were isolated from infected leaves of *L. chinense* and *C. sinensis* in Fujian Province, China. Based on morphology and a multi-gene phylogeny of the internal transcribed spacer regions with the intervening 5.8S nrRNA gene (ITS), the 28S large subunit of nuclear ribosomal RNA (LSU), the second largest subunit of RNA polymerase II (RPB2), and the translation elongation factor 1-α gene (TEF1-α), *Melanconiellaloropetali* sp. nov. and *Melanconiellacamelliae* sp. nov. were identified and described herein. Detailed descriptions, illustrations, and a key to the known species of *Melanconiella* are provided.

**Discussion:**

These data identify new species of *Melanconiella*, expanding the potential range and distribution of these dark septate fungi. The developed keys provide a reference source for further characterization of these fungi.

## 1. Introduction

Species of *Melanconiella* (Melanconiellaceae, Diaporthales) are important fungal wood pathogens and endophytes in Europe and North America (Du et al., 2017). Based on the dark-colored ascospore characteristics of the fungus, the genus *Melanconiella* was established and typified as *M. spodiaea*, with numerous revisions and adjustments occurring over the years (Saccardo, 1882; Voglmayr et al., 2012). These fungi belong within the broader category of dark septate fungi, that includes the dark septate root endophytes (DSE) which are conidial Ascomycetes (Jumpponen and Trappe, 1998; Ruotsalainen et al., 2022). DSE have been isolated from over 600 plant species, and have been distributed into >320 genera, occurring from the tropics to arctic and alpine habitats. These fungi comprise a heterogeneous group overlapping with soil, saprotrophic rhizoplane-inhabiting, mycorrhizal, and obligate/facultatively pathogenic fungi within ecosystems and in terms of their biology. Of note, many DSE have been examined in particular due to their effects on plant resistances to a wide range of biotic and abiotic stress (Santos et al., 2021). Within the past 30 years, 21 species in the genus *Melanconiella* have been recorded (Voglmayr et al., 2012). Wehmeyer (1926) indicated that most ascospores are arranged in a single row and only one type of conidia are needed to distinguish between *Melanconiella* and *Melanconis*. However, Wehmeyer (1941) considered *Melanconiella* to be a synonym of *Melanconis*, based on new classification standards. This viewpoint was supported by most scholars and led to confusion between *Melanconiella* and *Melanconis* (Voglmayr et al., 2012).

Since the development of modern genetic methods, molecular phylogenetic analysis has been applied to the taxonomy of *Melanconiella.* Based on the nucleotide sequence of the 28S large subunit of nuclear ribosomal RNA (LSU), the genus *Melanconiella* was separated from Melanconidaceae by Castlebury et al. (2002). Based on a multi-gene phylogeny (ITS, LSU, RPB2 and TEF1-α), Voglmayr et al. (2012) confirmed that *Melanconiella* was a special branch of *Melanconis* and suggested 13 accepted species. The genus *Melanconiella* was later accommodated into the Melanconiellaceae (Senanayake et al., 2017). A new species (*M. syzygii*) from diseased leaves of *Syzygium* sp. was added to *Melanconiella* by Crous et al. (2016). Du et al. (2017) reported a new species (*M. cornuta*) from *Cornus controversa* in Shaanxi Province. However, on the basis of phylogenetic analysis of combined ITS, LSU, CAL, RPB2 and TEF1-α sequence data, Fan et al. (2018) suggested that *M.cornuta* should be transferred from *Melanconiella* to *Sheathospora.* Meanwhile, two additional species (*M. betulicola* and *M. corylina*) were added to *Melanconiella* (Fan et al., 2018).

In the present study, four specimens of *Melanconiella* were collected from diseased leaves of *Loropetalum chinense* and *Camellia sinensis* in Fujian Province, China. Here, we sought to:

i. Determine/extend the host range and geographical distribution of *Melanconiella*;
ii. Report new species *Melanconiella loropetali* sp. nov. and *Melanconiella camelliae* sp. nov. with detailed descriptions and illustrations;
iii. Compare these new species with other species in the genus *Melanconiella*; and
iv. Provide a key to all known species of *Melanconiella.*

## 2. Materials and methods

### 2.1. Fungal isolates and morphology

Specimen samples were collected from the Wuyi Mountain National Nature Reserve, Fujian Province, China. Colonies of the two new species *Melanconiella* described herein were isolated from diseased leaves of *Loropetalum chinense* and *Camellia sinensis* using standard issue isolation methods (Senanayake et al., 2020; Jiang et al., 2022). Tissue fragments about 25 mm^2^ in total extent were taken from the margin of leaves with typical spot symptoms. These were sterilized by immersion in 75% ethanol solution for 60 s, placed in sterile deionized water for 45 s, transferred to 5% sodium hypochlorite solution for 30 s, and then rinsed three times in sterile deionized water for 60 s. The fragments were dried with sterilized filter paper and then transferred onto PDA plates (PDA medium: deionized water 1,000 mL, potato 200 g, agar 20 g, dextrose 20 g, pH ~7.0, available after sterilization) and incubated at 25°C for 5–7 days (Cai et al., 2009). Growing edges of fungal hyphae were removed to new PDA plates (at least two times) to obtain pure cultures. To promote sporulation and visualize the appearance of colonies, hyphae were inoculated onto the center of PDA prepared with pine needle and synthetic low nutrient agar SNA (SNA medium: deionized water 1,000 mL, KH_2_PO_4_ 1 g, KNO_3_ 1 g, MgSO_4_^.^7H_2_O 0.5 g, KCl 0.5 g, dextrose 0.2 g, sucrose 0.2 g, agar 12 g, available after sterilization) and incubated at 25°C under alternating conditions of 12 h near ultraviolet light and 12 h dark (Cai et al., 2009; Zhang et al., 2023).

Following 7–14 days of incubation, morphological characteristics of the (*Melanconiella*) isolates were recorded as per previous reports (Cai et al., 2009). Photographs of the colonies were taken at 7 days and 14 days after inoculation using a digital camera (Canon EOS 6D MarkII). Micromorphological characters of conidiomata were observed using a stereomicroscope (Nikon SMZ74), as well as by a compound microscope and by scanning electron microscopy (SEM, Nikon Ni-U; HITACHI SU3500). Measurements of micromorphological structures were determined using Digimizer software. All strains were stored in 10% sterilized glycerin and sterile water at 4°C for detailed studies in the future. The specimens were deposited in the Herbarium Mycologicum Academiae Sinicae, Institute of Microbiology, Chinese Academy of Sciences, Beijing, China (HMAS), accession numbers given in text. Living cultures were deposited in the China General Microbiological Culture Collection Center (CGMCC). Taxonomic information of the new taxa was submitted to MycoBank (http://www.mycobank.org; Crous et al., 2004).

### 2.2. DNA extraction and amplification

Genomic DNA was extracted from *Melanconiella* fungal mycelia grown on PDA for 14 days, using the Fungal DNA Mini Kit (OMEGA-D3390, Feiyang Biological Engineering Corporation, Guangzhou, China) according to the product manual. Nucleotide sequences were obtained from four gene loci including the internal transcribed spacer regions with the intervening 5.8S nrRNA gene (ITS), the 28S large subunit of nuclear ribosomal RNA (LSU), the second largest subunit of RNA polymerase II (RPB2), and the translation elongation factor 1-α gene (TEF1-α). These were amplified by primer pairs and the polymerase chain reaction (PCR) programs as described (Table 1).

**Table 1:** Gene regions and PCR primers and programs used in this study.

Gene	Primers	Sequence (5′-3′)	PCR Cycles	References
ITS	ITS5	GGA AGT AAA AGT CGT AAC AAG G	(95°C: 30 s, 54°C: 30 s, 72°C: 1 min) × 35 cycles	White et al. (1990)
	ITS4	TCC TCC GCT TAT TGA TAT GC		
LSU	LR0R	GTA CCC GCT GAA CTT AAG C	(95°C: 30 s, 52°C: 30 s, 72°C: 1 min) × 35 cycles	Vilgalys and Hester (1990) and Rehner and Samuels (1994)
	LR5	TCC TGA GGG AAA CTT CG		
TEF1-α	EF1728F	CATCGAGAAGTTCGAGAAGG	(95°C: 30 s, 54°C: 30 s, 72°C: 1 min) × 35 cycles	Chaverri and Samuels (2003) and Jaklitsch et al. (2006)
	TEF1LLErev	AAC TTG CAG GCA ATG TGG		
RPB2	fRPB2-5f	GAY GAY MGW GAT CAY TTY GG	(95°C: 30 s, 56°C: 30 s, 72°C: 1 min) × 35 cycles	Liu et al. (1999)
	fRPB2-7cr	CCC ATW GCY TGC TTM CCC AT		

PCR was performed using a Bio-Rad Thermocycler (California, United States). Amplification reactions were performed in a 25 μL reaction volume which contained 12.5 μL of 2 × Rapid Taq Master Mix (Vazyme, Nanjing, China), 1 μL of each forward and reverse primer (10 μM) (Sangon, Shanghai, China), and 1 μL of template genomic DNA in the amplifier. These were adjusted with distilled deionized water to a total volume of 25 μL. PCR products were visualized on using 1% agarose gel electrophoresis. Bidirectional (both strand) sequencing was conducted by the Tsingke Company Limited (Fuzhou, China). Consensus sequences were assembled using MEGA 7.0 (Kumar et al., 2016).

### 2.3. Phylogenetic analysis

To construct the phylogenetic trees for *Melanconiella*, the sequences generated from the four strains considered in this study, and all available reference sequences were downloaded from GenBank. Multiple sequence alignments for ITS, LSU, RPB2 and TEF1-α were constructed and carried out using the MAFFT v.7.11 online program (http://mafft.cbrc.jp/alignment/server/; Katoh et al., 2019) and corrected manually using MEGA 7.0 (Kumar et al., 2016). Phylogenetic analyzes were based on maximum likelihood (ML) and Bayesian inference (BI) methods.

**Figure 1:**
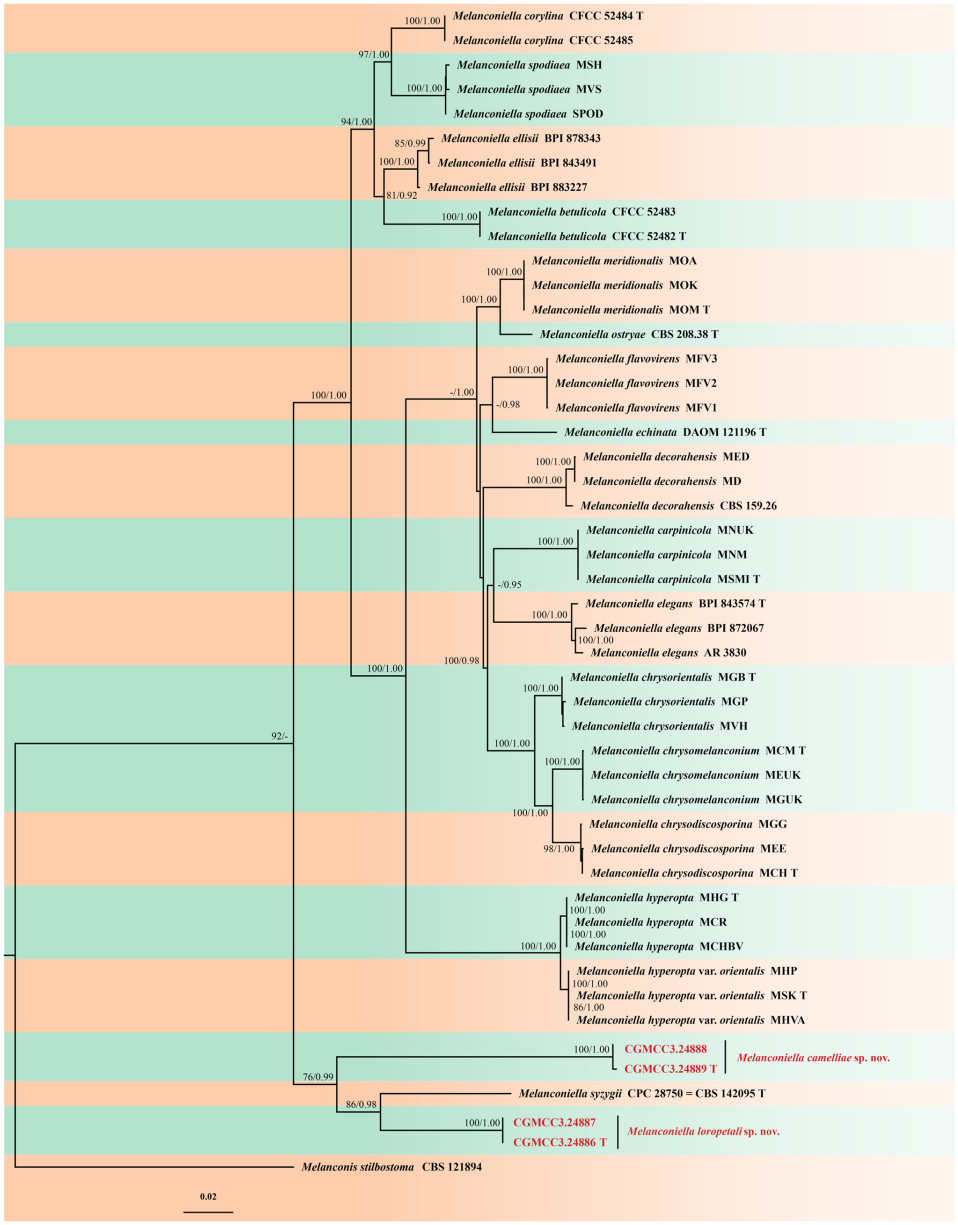
Phylogram of *Melanconiella* based on combined ITS, LSU, RPB2 and TEF1-α genes, with *Melanconis stilbostoma* (CBS 121894) as outgroup. The ML and BI bootstrap support values above 70% and 0.90 BYPP are shown at the first and second position, respectively. Strains marked with “T” are ex-type or ex-epitype. Strains from this study are shown in red. The scale bar at the left–bottom represents 0.02 substitutions per site.

The ML was run on the CIPRES Science Gatewayportal by RaxML-HPC2 on XSEDE v. 8.2.12 (Miller et al., 2010; Stamatakis, 2014). Bayesian analysis was performed in MrBayes on XSEDE v. 3.2.7a (Huelsenbeck and Ronquist, 2001; Ronquist and Huelsenbeck, 2003; Ronquist et al., 2012) and the best evolutionary model for each partition was determined using MrModeltest v. 2.3 (Posada and Crandall, 1998; Nylander, 2004; Darriba et al., 2012) and included the analyzes (Dong et al., 2020). Four simultaneous Markov Chain Monte Carlo (MCMC) chains were run for 160,000 generations. In addition, a sampling frequency of 100 generations was performed in the test. The burn-in fraction was set to 0.25 and posterior probabilities (PP) were determined from the remaining trees (He et al., 2022). The consensus trees were plotted using FigTree v. 1.4.4 (http://tree.bio.ed.ac.uk/software/figtree; Rambaut, 2018) and edited using Adobe Illustrator CS 6.0 (Figure 1). New sequences generated in this study were deposited in GenBank (https://www.ncbi.nlm.nih.gov; Table 2).

**Table 2:** Species and GenBank accession numbers of DNA sequences used in this study, with new sequences indicated in bold.

Species	Strain/voucher number	Host	Country	GenBank accession number			
				ITS	LSU	RPB2	TEF1-α
*Melanconiella betulicola*	CFCC 52482^T^	*Betula albosinensis*	China	MK096312	MK096352	MK096397	MK096272
	CFCC 52483	*Betula albosinensis*	China	MK096313	MK096353	MK096398	MK096273
*Melanconiella camelliae*	**CGMCC3.24888**	*Camellia sinensis*	**China**	**OQ932924**	**OQ940521**	**OQ947402**	**OQ947400**
	**CGMCC3.24889** ^**T**^	*Camellia sinensis*	**China**	**OQ932925**	**OQ940522**	**OQ947403**	**OQ947401**
*Melanconiella carpinicola*	MNM	*Carpinus betulus*	Austria	JQ926232	JQ926232	JQ926304	JQ926370
	MNUK	*Carpinus betulus*	United Kingdom	JQ926234	JQ926234	JQ926306	JQ926372
	MSMI^T^	*Carpinus betulus*	Austria	JQ926235	JQ926235	JQ926307	JQ926373
*Melanconiella chrysodiscosporina*	MCH^T^	*Carpinus betulus*	Austria	JQ926238	JQ926238	JQ926310	JQ926376
	MEE	*Carpinus betulus*	Austria	JQ926240	JQ926240	JQ926312	JQ926378
	MGG	*Carpinus betulus*	Austria	JQ926242	JQ926242	JQ926314	JQ926380
*Melanconiella chrysomelanconium*	MCM^T^	*Carpinus betulus*	Austria	JQ926247	JQ926247	JQ926319	JQ926385
	MEUK	*Carpinus betulus*	United Kingdom	JQ926249	JQ926249	JQ926321	JQ926387
	MGUK	*Carpinus betulus*	United Kingdom	JQ926255	JQ926255	JQ926327	JQ926393
*Melanconiella chrysorientalis*	MGB^T^	*Carpinus orientalis*	Croatia	JQ926256	JQ926256	JQ926328	JQ926394
	MGP	*Carpinus orientalis*	Croatia	JQ926257	JQ926257	JQ926329	JQ926395
	MVH	*Carpinus orientalis*	Croatia	JQ926259	JQ926259	JQ926331	JQ926397
*Melanconiella corylina*	CFCC 52484^T^	*Corylus mandshurica*	China	MK096314	MK096354	MK096399	MK096274
	CFCC 52485	*Corylus mandshurica*	China	MK096315	MK096355	MK096400	MK096275
*Melanconiella decorahensis*	CBS 159.26	*Betula* sp.	United States	JQ926260	JQ926260	JQ926332	JQ926398
	MD	*Betula pendula*	France	JQ926261	JQ926261	JQ926333	JQ926399
	MED	*Betula pendula*	France	JQ926262	JQ926262	JQ926334	JQ926400
*Melanconiella echinata*	DAOM 121196^T^	*Carpinus caroliniana*	United States	JQ926263	JQ926263	–	–
*Melanconiella elegans*	AR 3830	*Carpinus caroliniana*	United States	JQ926264	JQ926264	JQ926335	JQ926401
	BPI 843574^T^	*Carpinus caroliniana*	United States	JQ926266	JQ926266	JQ926337	JQ926403
	BPI 872067	*Carpinus caroliniana*	United States	JQ926267	JQ926267	JQ926338	JQ926404
*Melanconiella ellisii*	BPI 843491	*Carpinus caroliniana*	United States	JQ926268	JQ926268	–	JQ926405
	BPI 878343	*Carpinus caroliniana*	United States	JQ926271	JQ926271	JQ926339	JQ926406
	BPI 883227	*Carpinus caroliniana*	United States	JQ926269	JQ926269	–	–
*Melanconiella flavovirens*	MFV1	*Carpinus caroliniana*	Austria	JQ926274	JQ926274	JQ926342	JQ926409
	MFV2	*Carpinus caroliniana*	Austria	JQ926275	JQ926275	JQ926343	JQ926410
	MFV3	*Carpinus caroliniana*	Italy	JQ926276	JQ926276	JQ926344	JQ926411
*Melanconiella hyperopta*	MCHBV	*Carpinus betulus*	Austria	JQ926280	JQ926280	JQ926346	JQ926413
	MCR	*Carpinus betulus*	Austria	JQ926283	JQ926283	JQ926349	JQ926416
	MHG^T^	*Carpinus betulus*	Switzerland	JQ926285	JQ926285	JQ926351	JQ926418
*Melanconiella hyperopta* var. *orientalis*	MHP	*Carpinus orientalis*	Croatia	JQ926288	JQ926288	JQ926352	JQ926420
	MHVA	*Carpinus orientalis*	Croatia	JQ926287	JQ926287	JQ926353	JQ926419
	MSK^T^	*Carpinus orientalis*	Croatia	JQ926286	JQ926286	JQ926354	JQ926421
*Melanconiella loropetali*	**CGMCC3.24886** ^**T**^	*Loropetalum chinense*	**China**	**OQ928185**	**OQ940480**	**OQ935353**	**OQ947398**
	**CGMCC3.24887**	*Loropetalum chinense*	**China**	**OQ928186**	**OQ940481**	**OQ935354**	**OQ947399**
*Melanconiella meridionalis*	MOA	*Ostrya carpinifolia*	Austria	JQ926289	JQ926289	JQ926355	JQ926422
	MOK	*Ostrya carpinifolia*	Croatia	JQ926290	JQ926290	JQ926356	JQ926423
	MOM^T^	*Ostrya carpinifolia*	Austria	JQ926291	JQ926291	JQ926357	JQ926424
*Melanconiella ostryae*	CBS 208.38^T^	*Ostrya virginiana*	United States	JQ926297	JQ926297	JQ926363	JQ926430
*Melanconiella spodiaea*	MVS	*Carpinus orientalis*	Croatia	JQ926299	JQ926299	JQ926365	JQ926432
	MSH	*Carpinus betulus*	Austria	JQ926298	JQ926298	JQ926364	JQ926431
	SPOD	*Carpinus betulus*	United States	JQ926300	JQ926300	JQ926366	JQ926433
*Melanconiella syzygii*	CPC 28750 = CBS 142095^T^	*Syzygium* sp.	Malaysia	KY173417	KY173508	–	–
*Melanconis stilbostoma*	CBS 121894	*Betula pendula*	Italy	JQ926229	JQ926229	JQ926302	JQ926368

Strains marked with “T” are ex-type or ex-epitype.

## 3. Results

### 3.1. Phylogeny

Four isolates of *Melanconiella* were identified as representing two new species based on an analysis of combined ITS, LSU, RPB2, and TEF1-α gene nucleotide sequences, and combined with 47 isolates of *Melanconiella* along with *Melanconis stilbostoma* (CBS 121894) as the outgroup taxon (Voglmayr et al., 2012). The dataset had an aligned length of 6,068 characters including gaps (i.e., ITS: 1–1723, LSU: 1724–3,416, RPB2: 3417–4,590, TEF1-α: 4591–6,068). Of these characters, 4,369 were constant, 357 were variable and parsimony-uninformative, and 1,342 were parsimony-informative.

The Bayesian analysis was performed for 160,000 generations, resulting in 3202 total trees, of which 2,402 trees were used to calculate the posterior probabilities. The BI posterior probabilities were plotted on the ML tree. For the ML and BI analyzes, GTR + I + G for ITS, RPB2, TEF1-α, and LSU [Lsetnst = 6, rates = invgamma; Prsetstatefreqpr = Dirichlet (1,1,1,1)], were selected and incorporated into the analysis. Bayesian analysis resulted in an average standard deviation of split frequencies = 0.009565. The topology of the ML tree was consistent with that of the Bayesian tree. Hence, the ML tree is presented (Figure 1).

ML bootstrap support values (≥70%) and Bayesian posterior probability (≥0.90) are shown as the first and second positions, respectively, above the nodes. The 48 strains were assigned to 20 species clades based on the four gene loci phylogeny (Figure 1). The four strains studied herein represented two novel species. The new species *Melanconiella loropetali* showed a close relationship to *M. syzygii* (CBS 142095), with good support (ML-BS:86% and BYPP:0.98). The new species *M.camelliae* (CGMCC3.24889) showed a close relationship to *M. syzygii* (CBS 142095) and *M. loropetali* (CGMCC3.24886) with good support (ML-BS:76% and BYPP:0.99).

### 3.2. Taxonomy

**Figure 2:**
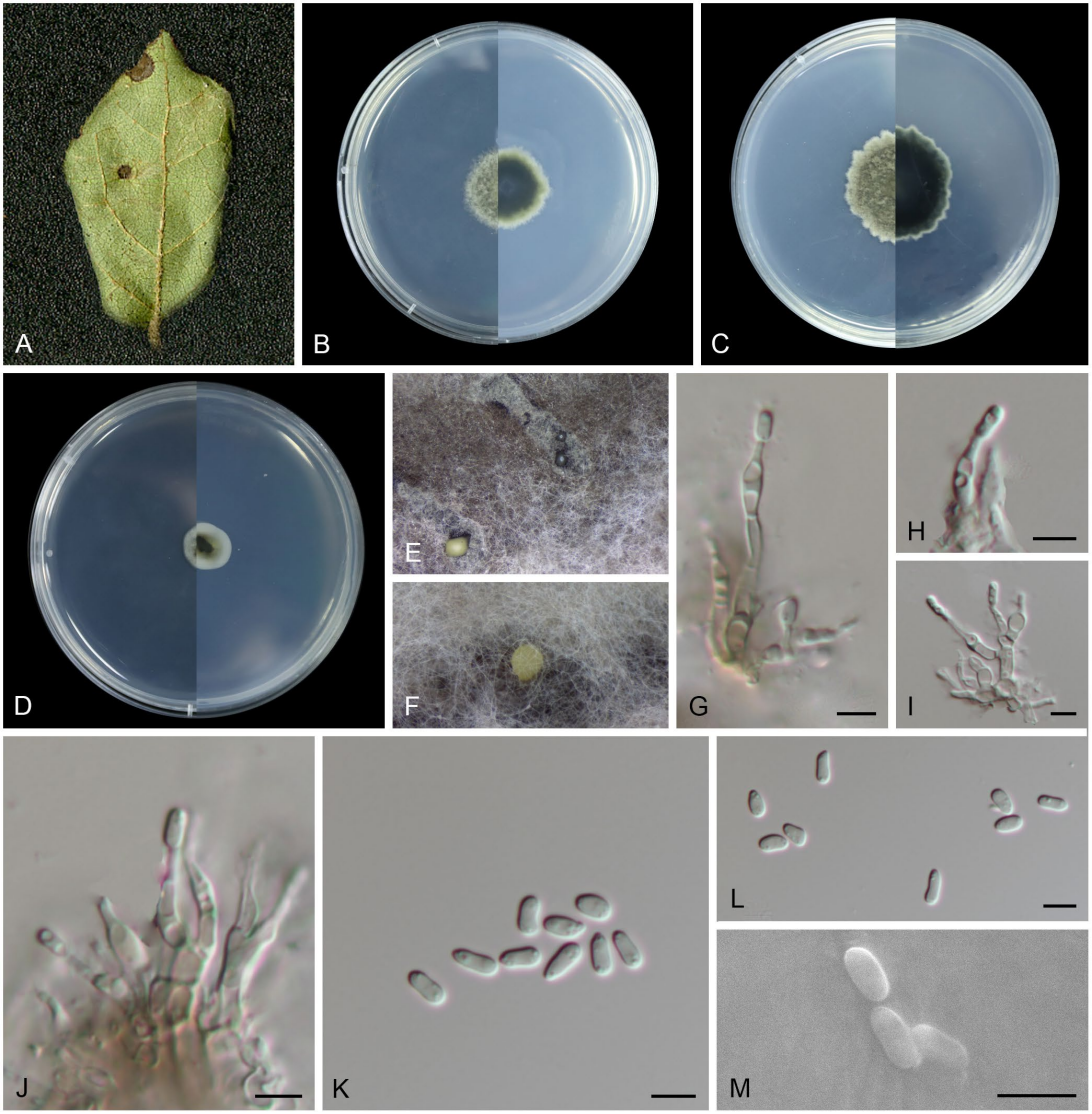
Morphological characteristics *Melanconiella loropetali* (holotype HMAS 257907) **(A)** Symptomatic leaves of *Loropetalum chinense*. **(B)** Surface and reverse sides of colony after incubation for 7 days on PDA **(C)** and 14 days. **(D)** Surface and reverse sides of colony after incubation for 7 days on SNA. **(E,F)** Conidiomata. **(G-J)** Conidiophores, conidiogenous cells and conidia. **(K-M)** Conidia. **(G-M)** Scale bars: 5 μm.

***Melanconiella loropetali*** T.C. Mu and Jun Z. Qiu, sp. nov. (Figure 2).

MycoBank number: MB848666.

Holotype: CHINA, Fujian Province, Fujian Wuyi Mountain National Nature Reserve, 117°41′19.82″E, 27°44′53.91″N, from diseased leaves of *Loropetalum chinense*, 7 September 2022, T.C. Mu (holotype HMAS 257907; ex-type living culture CGMCC3.24886).

Etymology: The epithet “*loropetali*” pertains to the generic name of the host plant *Loropetalum chinense*.

Description: Leaf spots circular, sunken in the middle, brown or tan, 4–10 mm diam. Conidiomata acervular to pycnidial, erumpent on agar, solitary, globose, black or creamy, 330–410 μm diam, black and cream conidial droplets exuding from the ostioles. Conidiophores hyaline to light brown, smooth, fusiform, subcylindrical, 1–5-septate, branched and septate at the base, 9.2–26.8 × 1.6–3.5 μm. Conidiogenous cells hyaline, smooth, phialidic, subglobular and globular, subcylindrical, 1.8–4.3 × 1.5–3.4 μm. Conidia unicellular, hyaline, smooth, narrowly ellipsoid to fusoid, subcylindrical, thin-walled, (3.3–6.6 × 1.6–3.0 μm, mean ± SD = 4.57 ± 0.75 × 2.17 ± 0.33 μm, L/W ratio = 2.2, *n* = 30). Sexual morph was not observed.

Culture characteristics: Colonies on PDA flat with feathery margin, aerial mycelium white or dark brick-red, floccose. On PDA surface pale mouse gray to mouse gray, reverse black. On SNA surface and reverse gray in the center and white margin. PDA attaining 18.5–22.8 mm in diameter after 7 days, at 25°C, with a calculated growth rate 2.6–3.2 mm/day. SNA attaining 9.8–13.6 mm in diameter after 7 days, at 25°C, slow growing, calculated growth rate 1.4–1.9 mm/day.

Other specimens examined: CHINA, Fujian Province, Fujian Wuyi Mountain National Nature Reserve, 117°41′19.82″E, 27°44′53.91″N, from diseased leaves of *Loropetalum chinense*, 7 September 2022, T.C. Mu (paratype HMAS 257908; ex-paratype living culture CGMCC3.24887).

Notes: In this study, a member of the genus *Melanconiella* was collected from the Hamamelidaceae. Two strains (CGMCC3.24886; CGMCC3.24887) were isolated from diseased leaves of *Loropetalum chinense* and identified as *Melanconiella loropetali* sp. nov. Phylogenetic analysis using four genes showed that *M. loropetali* formed an independent clade (Figure 1) and was phylogenetically distinct from *M. syzygii* (CBS 142095) with high statistical support (86% ML/0.98 PP, Figure 1). The nucleotide comparison of ITS sequences of *M. syzygii* revealed 58 bp (58/601 bp, 9.65%) nucleotide differences. The nucleotide comparison of LSU sequences of *M. syzygii* revealed 14 bp (14/817 bp, 1.71%) nucleotide differences. Morphologically, *M. loropetali* differs from *M. syzygii* in having smaller conidia and conidiophores (3.3–6.6 × 1.6–3.0 vs. 8.0–11.0 × 5.0–6.0 μm; 9.2–26.8 × 1.6–3.5 vs. 12.0–30.0 × 4.0–6.0 μm [Crous et al., 2016]). Therefore, we describe this fungus as a new species.

**Figure 3:**
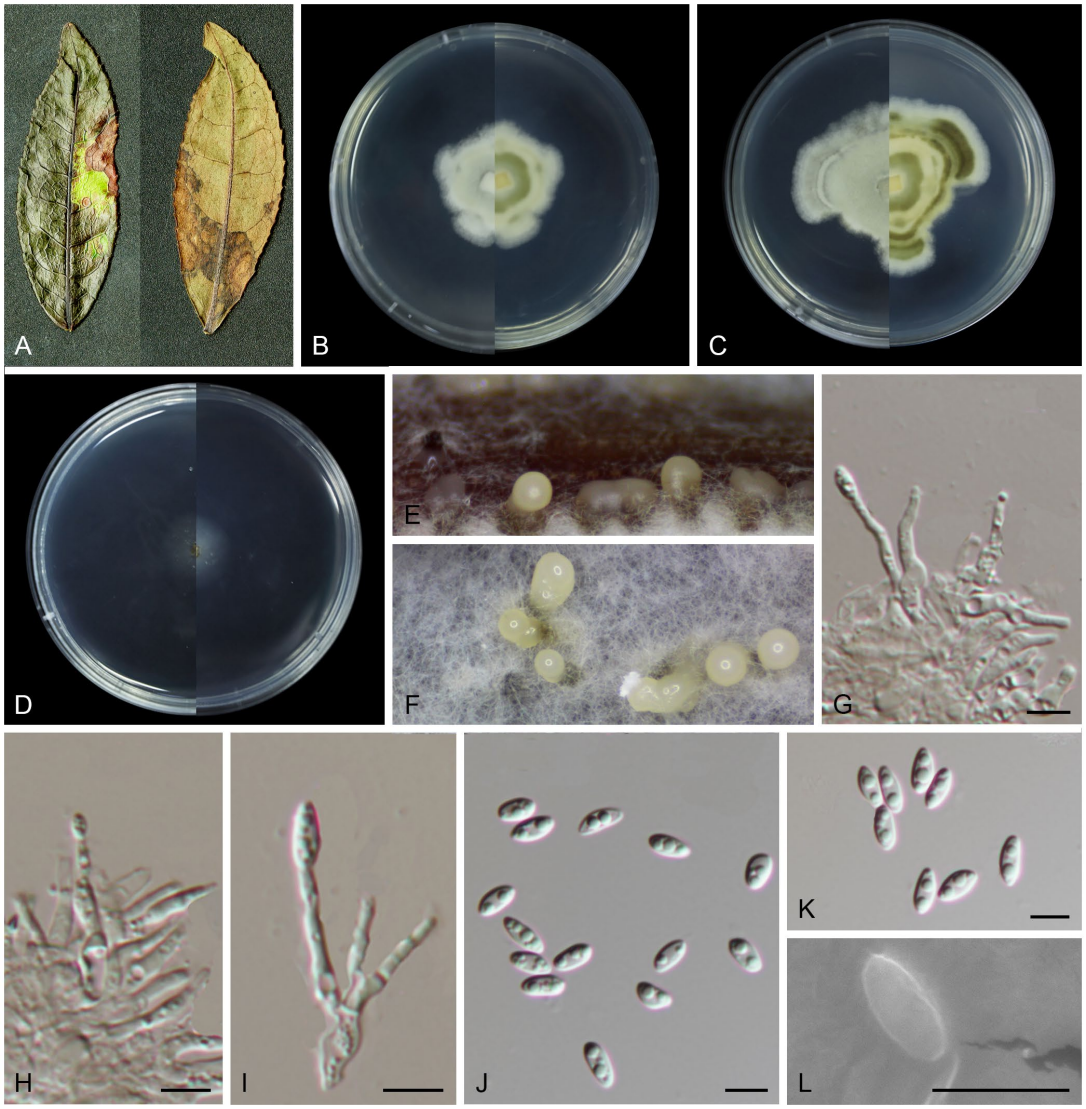
Morphological characteristics *Melanconiella camelliae* (holotype HMAS 257910) **(A)** Symptomatic leaves of *Camellia sinensis*. **(B)** Surface and reverse sides of colony after incubation for 7 days on PDA **(C)** and 14 days. **(D)** Surface and reverse sides of colony after incubation for 7 days on SNA. **(E,F)** Conidiomata. **(G-I)** Conidiophores, conidiogenous cells and conidia. **(J-L)** Conidia. **(G-L)** Scale bars: 5 μm.

***Melanconiella camelliae*** T.C. Mu and Jun Z. Qiu, sp. nov. (Figure 3).

MycoBank number: MB848667.

Holotype: CHINA, Fujian Province, Fujian Wuyi Mountain National Nature Reserve, 117°41′19.81″E, 27°44′53.92″N, from diseased leaves of *Camellia sinensis*, 7 September 2022, T.C. Mu (holotype HMAS 257910; ex-type living culture CGMCC3.24889).

Etymology: The epithet “*camelliae*” refers to the generic name of the host plant *Camellia sinensis*.

Description: Leaf spots irregular, brown or umber. Conidiomata acervular to pycnidial, solitary, 180–260 μm diam, with cream conidial droplets exuding from the ostioles. Conidiophores, hyaline, smooth, 1–3-septate, mostly straight, cylindrical-clavate, branched at the base, 10.4–18.6 × 1.4–2.1 μm. Conidiogenous cells hyaline, smooth, circular, elliptical, phialidic, 1.5–3.5 × 1.0–1.7 μm. Conidia hyaline, smooth, oblong elliptical and fusoid, multi-guttulate, (4.7–5.9 × 2.0–2.8 μm, mean ± SD = 5.36 ± 0.35 × 2.32 ± 0.23 μm, L/W ratio = 2.3, n = 30). Sexual morph was not observed.

Culture characteristics: Colonies on PDA flat with irregular stripes, aerial mycelium white, cottony. On PDA surface white, reverse yellowish and darker. Colonies on SNA sparse hyphae, slow growing. PDA attaining 26.3–31.9 mm in diameter after 7 days, at 25°C, with a calculated growth rate 3.7–4.5 mm/day. SNA attaining 13.8–18.8 mm in diameter after 7 days, at 25°C, slow growing, calculated growth rate 1.9–2.6 mm/day.

Other specimens examined: CHINA, Fujian Province, Fujian Wuyi Mountain National Nature Reserve, 117°41′19.81″E, 27°44′53.92″N, from diseased leaves of *Camellia sinensis*, 7 September 2022, T.C. Mu (paratype HMAS 257909; ex-paratype living culture CGMCC3.24888).

Notes: In this study a member of the *Melanconiella* was collected for the first time from the Theaceae. Two strains (CGMCC3.24888; CGMCC3.24889) were isolated from diseased leaves of *Camellia sinensis* and identified as *Melanconiella camelliae* sp. nov. Phylogenetic analysis of four genes showed that *M. camelliae* formed an independent clade (Figure 1) and was phylogenetically distinct from *M. syzygii* and *M. loropetali* with moderate statistical support (76% ML/0.99 PP, Figure 1). The nucleotide comparison of ITS sequences of *M. syzygii* revealed 116 bp (116/488 bp, 23.77%) nucleotide differences. The nucleotide comparison of LSU sequences of *M. syzygii* revealed 53 bp (53/821 bp, 6.46%) nucleotide differences. Morphologically, *M. camelliae* differs from *M. syzygii* in having smaller conidia and conidiophores (4.7–5.9 × 2.0–2.8 vs. 8.0–11.0 × 5.0–6.0 μm; 10.4–18.6 × 1.4–2.1 vs. 12.0–30.0 × 4.0–6.0 μm [Crous et al., 2016]). *Melanconiella camelliae* differs from *M. loropetali* in having smaller conidiophores (10.4–18.6 × 1.4–2.1 vs. 9.2–26.8 × 1.6–3.5 μm). Therefore, we describe this fungus as a new species.

### 3.3. Key to species of *Melanconiella*

1. Conidiophores septate……………2.

1. Conidiophores aseptate……………4.

2. On *Camellia sinensis*; conidiophores 1–3-septate; conidial size 4.7–5.9 × 2.0–2.8 μm, L/W ratio = 2.3……………*M. camelliae* sp. nov.

2. Conidiomata solitary, globose……………3.

3. On *Syzygium*; conidiophores ampulliform, unbranched; conidiogenous cells integrated, terminal; conidial size 8.0–11.0 × 5.0–6.0 μm……………*M. syzygii.*

3. On *Loropetalum*; conidiophores 1–5-septate; conidial size 3.3–6.6 × 1.6–3.0 μm……………*M. loropetali* sp.nov.

4. Ascospores dark brown; conidia dark brown……………5.

4. Ascospores hyaline; conidia hyaline or dark brown……………7.

5. Ascospores without appendages; conidia pip-shaped, multiguttulate when fresh, with a large guttule when dead; on *Betula* spp. in the north temperate zone……………*M. decorahensis.*

5. Conidia usually ellipsoid, guttulate……………6.

6. Perithecia 0.3–0.5 mm diam, up to 20 per stroma; conidia variable in shape, ovoid, obovoid, usually not distinctly pip-shaped; on *Carpinus* in Europe……………*M. spodiaea.*

6. Ectostromatic disc inconspicuous and cracked around the margin at maturity; conidia narrowly ellipsoid, 1–3 guttulate; on *Corylus mandshurica* in China……………*M. corylina.*

7. On *Carpinus*……………8.

7. On other hosts……………16.

8. Ascospores with obvious ascospore wall and with monomorphic, (sub) globose to bullet-shaped ascospore cells mostly larger than 7 μm in width; commonly with yellow ectostroma……………9.

8. Ascospores without obvious ascospore wall and with slightly to distinctly dimorphic cells mostly smaller than 7 μm in width; ectostroma gray, brownish, yellowish, pale brown, cream or yellow……………11.

9. Ascospores 16.0–27.5 × 7.5–15.5 μm; with obvious conidiomata containing dark brown conidia; on *C. betulus*……………*M. chrysomelanconium.*

9. Ascospores 15.5–22.0 × 6.0–15.0 μm; hyaline conidia……………10.

10. Entostroma crumbly, of subhyaline to yellowish hyphae; conidial size 12.5–19.0 × 4.5–6.0 μm; on *C. betulus*……………*M. chrysodiscosporina.*

10. Asci rarely biseriate ascospores; conidial size 11.5–15.5 × 5.5–7.5 μm; on *C. orientalis*……………*M. chrysorientalis.*

11.Ascospores were usually more than 5.5 μm in width and 18.5 μm in length……………12.

11. Ascospores were usually less than 5.5 μm in width and 18.5 μm in length……………14.

12. On *Carpinus* in Europe; ectostromatic discs concave, flat or slightly pustulate and little projecting above the perithecial level, paler to fawn; central column pale yellow to grayish brown……………13.

12. On *C. caroliniana* in North America; ectostromatic discs well-developed, distinctly projecting, often pulvinate, with circular, angular or ellipsoid outline, brownish, brown, cream or yellow; central column yellow when young, later green to brown; entostroma well-developed, of compacted hyphae, green or brown……………*M. echinata.*

13. Central column pale, yellowish, pale grayish brown or brownish; conidiogenous cells 10.0–30.0 × 1.5–3.0 μm; on *C. betulus*……………*M. hyperopta.*

13. Ascospores 2.5–3.6 × 1.8–2.3 μm; conidia often with one to three larger and few to numerous small guttules; On *C. orientalis*……………*M. hyperopta* var. *orientalis.*

14. Pseudostromata perithecia only rarely projecting, 0.8–1.7 mm diam; conidiogenous cells 10.0–18.0 × 1.5–2.5 μm; on *C. betulus* in Europe……………*M. carpinicola.*

14. On *C. caroliniana* or *Betula*……………15.

15. Ectostromatic discs distinctly projecting, mostly circular, angular or ellipsoid, often pulvinate, pale yellow to pale brown; or concealed by ostioles; ostioles evenly spaced in the disc; central column yellow, gray or brownish; conidiogenous cells 10.0–25.0 × 2.0–3.0 μm……………*M. elegans.*

15. Ectostromatic disc mostly circular; ostioles black……………16.

16. Ectostromatic disc mostly irregularly shaped, usually surrounded by irregular edges or flaps of bark, drab, light to dark gray or tanned; ostioles mostly marginal in the disc; central column gray or pale brown; conidial size 8.5–13.0 × 2.5–4.0 μm……………*M. ellisii.*

16. Ectostromatic disc usually buff to hazel; conidial size 9.5–15.0 × 2.0–5.5 μm; on *Betula* in China……………*M. betulicola.*

17. On *Corylus*; ascospores broadly fusoid; ascospore ends obviously subacute with persistent knob-like hyaline appendages; conidia often with one or two larger and numerous small guttules……………*M. flavovirens.*

17. On *Ostrya*; ascospores fusoid; ascospore ends narrowly rounded to subacute, without or with cap-like appendages……………18.

18. Ascospores 20.0–37.5 × 5.0–9.5 μm; conidia hyaline cylindrical to suballantoid; on *Ostrya carpinifolia* in Europe……………*M. meridionalis.*

18. Ascospores 14.5–23.0 × 4.0–8.5 μm; conidia dark brown with a light brown equatorial zone, without guttules or granules; on *Ostrya virginiana* in North America……………*M. ostryae.*

## 4. Discussion

In this study, fungal specimens were collected from diseased leaves of *Loropetalum chinense* and *Camellia sinensis* in China. Based on combined morphological and multi-gene phylogenetic analyzes, two new species, *Melanconiella loropetali* sp. nov. and *Melanconiella camelliae* sp. nov., were identified, with detailed descriptions and illustrations provided. To better clarify the relationship between species, as well as provide distinguishing characteristics useful for separating the isolates from similar species of *Melanconiella*, we provide a key to *Melanconiella.* These results extend the host range and geographical distribution of *Melanconiella.*

*Melanconis spodiaea*, the type species of the genus *Melanconiella*, was introduced by Saccardoin 1882. Currently, 40 names were recorded in the Index Fungorum and 43 species in MycoBank (http://www.indexfungorum.org/; https://www.mycobank.org/; accessed 22 April 2023).

In the late 19th and early 20th centuries, 21 species were classified within *Melanconiella*. Based on a multi-gene phylogeny (ITS, LSU, RPB2 and TEF1-α), Voglmayr et al. (2012) confirmed 13 species in the genus *Melanconiella*, including *M. carpinicola* (Fuckel) Voglmayr & Jaklitsch, *M. chrysodiscosporina* Voglmayr & Jaklitsch, *M. chrysomelanconium* Voglmayr & Jaklitsch, *M. chrysorientalis* Voglmayr & Jaklitsch, *M. echinata* Voglmayr & Jaklitsch, *M. elegans* Voglmayr & Jaklitsch, *M. ellisii* (Rehm ex Ellis & Everh.) Voglmayr & Jaklitsch, *M. flavovirens* (G.H. Otth) Voglmayr & Jaklitsch, *M. hyperopta* (Nitschke ex G.H. Otth) Voglmayr & Jaklitsch, *M. hyperopta* var. *orientalis* Voglmayr & Jaklitsch, *M. meridionalis* Voglmayr & Jaklitsch, *M. ostryae* (Dearn.) Voglmayr & Jaklitsch, and *M. spodiaea* (Tul. & C. Tul.) Sacc. (Voglmayr et al., 2012).

Voglmayr et al. (2012) considered species of *Melanconiella* to occur only in Europe and North America, and collected essentially from Betulaceae. Nevertheless, Crous et al. (2016) reported a new species (*M. syzygii*) from diseased leaves of *Syzygium* sp. in Malaysia, while *M. betulicola* and *M. corylina* were reported from symptomatic branches in China by Fan et al. (2018). In the present study, two new species are reported from diseased leaves of *Loropetalum chinense and Camellia sinensis* in China. It is likely that in Asia additional species of *Melanconiella* are present potentially from additional plant hosts. Both *M. loropetali* and *M. camelliae* were closely related to *M. syzygii* in their phylogenetic analyzes, with similar conidiomata characteristic. These reports, along with our own, may indicate a wider host specificity for *Melanconiella* than previously considered and may reflect local adaptations. However, *Melanconiella* may also exhibit opportunistic pathogenesis, with its main association with plants occurring as part of endophytic interactions (Du et al., 2017). Root inoculation of the cowpea plant (*Vochysia divergens*) with *M. elegans* (strain-21 W2) resulted in improved nutrition, growth, and photosynthesis under salt stress (Farias et al., 2020). However, research comparing endophytic vs. parasitic outcomes is lacking, and a more global analysis of the biology of *Melanconiella* is needed in order to better understand the ecology of these fungi.

## Data availability statement

The datasets presented in this study can be found in online repositories. The names of the repository/repositories and accession number(s) can be found in the article/Supplementary material.

## Author contributions

TM and JQ designed the experimental plan. TM and JC analyzed the data with help from ZZ, XG, and WZ. TM, CY, and MZ collected the samples from the field. TM and HS wrote the manuscript. SS, PL, and JQ reviewed this manuscript. All authors contributed to the article and approved the submitted version.

## Funding

The research was financed by the National Key R & D Program of China (nos. 2017YFE0122000 and 2022YFD1600300), the National Natural Science Foundation of China (nos. U1803232, 32270029, and 31670026), A Key Project from the Fujian Provincial Department of Science and Technology (no. 2020N5005), and Fujian Provincial Major Science and Technology Project (no. 2022NZ029017).

## Conflict of interest

The authors declare that the research was conducted in the absence of any commercial or financial relationships that could be construed as a potential conflict of interest.

## Publisher’s note

All claims expressed in this article are solely those of the authors and do not necessarily represent those of their affiliated organizations, or those of the publisher, the editors and the reviewers. Any product that may be evaluated in this article, or claim that may be made by its manufacturer, is not guaranteed or endorsed by the publisher.
