# Supplementary material for: Morphological and phylogenetic analyzes reveal two new species of Melanconiella from Fujian Province, China
Source: Front Microbiol. 2023 Aug 16;14:1229705. doi: 10.3389/fmicb.2023.1229705 (PMC10469505; doi:10.3389/fmicb.2023.1229705)
Supplement: Supplementary file 1 [file Data_Sheet_1.PDF]

frontiers

Supplementary Material

Morphological and phylogenetic analyses reveal two new species of *Melanconiella* from Fujian Province, China

Taichang Mu[1], Jinhui Chen[1], Zhiying Zhao[1], Weibin Zhang[1], Steven L. Stephenson[2], Chenjie Yang[1], Mengjia Zhu[1], Hailan Su[3], Pu Liu[4*], Xiayu Guan[5*], Junzhi Qiu[1*]

***Correspondence:**

Pu Liu, pul@jlau.edu.cn; Xiayu Guan, 47126940@qq.com; Junzhi Qiu, junzhiqiu@126.com

1. The concatenated ITS, LSU, RPB2 and TEF1-α sequence alignment

>Melanconiella_chrysomelanconium

CACACCGCCCGTCGCTACTACCGATTGAATGGTTCAGTGAGGCGTCCGGACTGGCCCAG
GGAGGTCGGCAACGACCACCCAGGGCCGGAAAGCTCTCCAAACTCGATCATTTAGAGG
AAGTAAAAGTCGTAACAAGGTCTCCGTTGGTGAACCAGCGGAGGGATCATTGATGGAAT
ACACGTCCTATACCCCTTTGTGAACATATACCTATCTCGTTGCCTCGGCGCCAGGCCCGG
GGTGCCCCCCAGGGGGTACCCCCCACCTATGGACCTCACGGTCCCGGGCGGAGCAGGCC
CGCCGGCGGCCCCTTTAAACTCTTGTTTTTAGAACCTATCTCTTCTGAGTGATACAAAAA
ATGAATCAAAACTTTCAACAACGGATCTCTTGGCTCTGGCATCGATGAAGAACGCAGCG
AAATGCGATAAGTAATGTGAATTGCAGAATTCAGTGAATCATCGAATCTTTGAACGCAC
ATTGCGCCCGCTGGGATTCCGGCGGGCATGCCTGTTCGAGCGTCATTTCAAACCCCTCAG
GCGTCCGCGCCTGGTGTTGGGGCATAGCCTGTAAAAAGGCTAGCCCTTAAATTCAGTGG
CGTGCTCGCTGAGACTCCCGGGTGTAGTAATCTCCTTCTCGCCCGGGCAGGCTCAGCGGC
GCACTTGCCGTAAAACCCGCTATCACGCACCTTCTGAAAGTTGACCTCGGATCAGGTAG
GAATACCCGCTGAACTTAAGCATATCAATAAGCGGAGGAAAAGAAACCAACAGGGATT
GCCCTAGTAACGGCGAGTGAAGCGGCAACAGCTCAAATTTGAAATCTGGCCCCCCACGG
GGCCCGAGTTGTAATTTGCAGAGGATGTCTTCTGGCGCGGTGCCTTCCGAGTTCCCTGGA
ACGGGACGCCATAGAGGGTGAGAGCCCCGTAATGGTCGGATACCAAGCCTGTGTGAAG
CTCCTTCGACGAGTCGAGTAGTTTGGGAATGCTGCTCTAAATGGGAGGTAAATCTCTTCT
AAAGCTAAATACTGGCCAGAGACCGATAGCGCACAAGTAGAGTGATCGAAAGATGAAA
AGCACCTTGAAAAGGGGGTTAAACAGTACGTGAAATTGTTGAAAGGGAAGCGCTCATG
ACCAGACTTGCGCCGGGCGGCTCATCAGGTGGTTCTGCCCCTGTGTACTCCGTCCCGGTT
CAGGCCAGCATCGGTTCGCGCCGGGGGACAAGAACGGTGGGAACGTGGCCCTCCCTCG
GGGGGGTGTTATAGCCCGCCGTAACGATATCCTGGTGCGGACCGAGGTTCGCGCACTCC

GCAAGGATGCTGGCGTAATGGTTATCAGTGACCCGTCTTGAAACACGGACCAAGGAGTC
GTCCTTTAGAGCGAGCGTCTGGGTGTCAAAACCCGCACGCGTAATGAAAGTGAAATCAG
GTGAGAGCCCCTTGTGGGCGCATCATCGACCGATCCTGATGTTCTCGGATGGATTTGAGT
AAGAGTTTTAACGGACGGACCCGAAAGACAGTGAACTATGCTTGTATAGGGTGAAGCCA
GAGGAAACTCTGGTGGAGGCTCGCAGCGGTTCTGACGTGCAAATCGATCGTCAAATATG
AGCATGGGGGCGAAAGACTAATCGAACCACACCGCCCGTCGCTACTACCGATTGAATGG
TTCAGTGAGGCGTCCGGACTGGCCCAGGGAGGTCGGCAACGACCACCCAGGGCCGGAA
AGCTCTCCAAACTCGATCATTTAGAGGAAGTAAAAGTCGTAACAAGGTCTCCGTTGGTG
AACCAGCGGAGGGATCATTGATGGAATACACGTCCTATACCCCTTTGTGAACATATACC
TATCTCGTTGCCTCGGCGCCAGGCCCGGGGTGCCCCCCAGGGGGTACCCCCCACCTATG
GACCTCACGGTCCCGGGCGGAGCAGGCCCGCCGGCGGCCCCTTTAAACTCTTGTTTTTAG
AACCTATCTCTTCTGAGTGATACAAAAAATGAATCAAAACTTTCAACAACGGATCTCTTG
GCTCTGGCATCGATGAAGAACGCAGCGAAATGCGATAAGTAATGTGAATTGCAGAATTC
AGTGAATCATCGAATCTTTGAACGCACATTGCGCCCGCTGGGATTCCGGCGGGCATGCC
TGTTCGAGCGTCATTTCAAACCCCTCAGGCGTCCGCGCCTGGTGTTGGGGCATAGCCTGT
AAAAAGGCTAGCCCTTAAATTCAGTGGCGTGCTCGCTGAGACTCCCGGGTGTAGTAATC
TCCTTCTCGCCCGGGCAGGCTCAGCGGCGCACTTGCCGTAAAACCCGCTATCACGCACCT
TCTGAAAGTTGACCTCGGATCAGGTAGGAATACCCGCTGAACTTAAGCATATCAATAAG
CGGAGGAAAAGAAACCAACAGGGATTGCCCTAGTAACGGCGAGTGAAGCGGCAACAGC
TCAAATTTGAAATCTGGCCCCCCACGGGGCCCGAGTTGTAATTTGCAGAGGATGTCTTCT
GGCGCGGTGCCTTCCGAGTTCCCTGGAACGGGACGCCATAGAGGGTGAGAGCCCCGTAA
TGGTCGGATACCAAGCCTGTGTGAAGCTCCTTCGACGAGTCGAGTAGTTTGGGAATGCT
GCTCTAAATGGGAGGTAAATCTCTTCTAAAGCTAAATACTGGCCAGAGACCGATAGCGC
ACAAGTAGAGTGATCGAAAGATGAAAAGCACCTTGAAAAGGGGGTTAAACAGTACGTG
AAATTGTTGAAAGGGAAGCGCTCATGACCAGACTTGCGCCGGGCGGCTCATCAGGTGGT
TCTGCCCCTGTGTACTCCGTCCCGGTTCAGGCCAGCATCGGTTCGCGCCGGGGGACAAG
AACGGTGGGAACGTGGCCCTCCCTCGGGGGGGTGTTATAGCCCGCCGTAACGATATCCT
GGTGCGGACCGAGGTTCGCGCACTCCGCAAGGATGCTGGCGTAATGGTTATCAGTGACC
CGTCTTGAAACACGGACCAAGGAGTCGTCCTTTAGAGCGAGCGTCTGGGTGTCAAAACC
CGCACGCGTAATGAAAGTGAAATCAGGTGAGAGCCCCTTGTGGGCGCATCATCGACCGA
TCCTGATGTTCTCGGATGGATTTGAGTAAGAGTTTTAACGGACGGACCCGAAAGACAGT
GAACTATGCTTGTATAGGGTGAAGCCAGAGGAAACTCTGGTGGAGGCTCGCAGCGGTTC
TGACGTGCAAATCGATCGTCAAATATGAGCATGGGGGCGAAAGACTAATCGAACGGTCC
GCTCCTGGCAAAGCTGTTCCGAAACATCGTCAAGAAGCTGACCGGCGACATGATGGCCT
ACATGAAGCGTTGTGTCAGCGAGGGTAAGCATTTTGATCTAGCGTTGGGCATCCGTCAC
TCGACCCTCACGAATGGCCTCAAGTATTCTCTTGCGACTGGAAACTGGGGCGAGCAGAA
GAAGGCGTCGAGTTCCACCGCTGGTGTCTCGCAGGTGTTGAACCGGTACACCTTCTCCTC
CACCCTGTCGCATTTGCGGCGCACGAACACGCCCATCGGTCGTGACGGCAAGCTGGCCA
AGCCTCGTCAGCTGCACAATACACACTGGGGTCTCGTCTGTCCAGCAGAGACTCCCGAG
GGACAGGCTTGCGGTCTCGTCAAGAACCTCTCTTTAATGTGCTACGTTAGCGTTGGTTCA
CCAGCTGAGCCTATCAAGGACTTCATGGTGCAACGTAATATGGAAGTCTTGGAGGAATA
CGAGCCCGGCTCCAGCCCTGACGCAACCAAGATCTTCATCAACGGCACCTGGGTTGGTG
TACACAGTGAACCAGCACACCTGGTGACGCTTGTGCAGGAGCTTCGGCGCAAATGCATC
ATTTCTCATGAAGTCTCCCTCGTTCGCGACATTCGCGACCGCGAGTTCAAGATCTTCTCT
GACGCTGGTCGTGTCATGCGGCCACTCTTCGTTATCAATCAAGAGGACAATCCCGAGAC
TGGAGCGCAGCAGGGCACTCTAGCTCTCACCAAGCACCACATTCGCCGTCTCGAGGAAG
ATGCTCAGTACCAACGCAAGAAGCATGACGACGACTATTTTGGCTGGGATGGTCTTCAG

AACAGCGGTTGTATCGAGTATCTGGATGCCGAGGAAGAGGAGACATCGATGATCTGCAT
GAGTCCTGAGGATCTTGAGGACTACCGCCAGCGCAAGGCTAGGGGCAAGGATGCCAAG
GATGATGAACCGGAGGACGATGGCAGAAGTCTGAACGCTCGTGTGAAGACGAAGATTA
ACCCCGACATCCACATGTACACCCACTGCGAGATCCATCCGAGCATGCTCCTGGGAATA
TGCGCCAGCATCATTCCCTTCCCGGATCACAATCAGGTGTGTAAAATTCTCGCAGTACTA
CTATCACTCCTGCTGACCACATATATCTAGTCACCTCGCATCATCCAATCCAGCATCACC
ACATTACCATCGCATAAGCTTCAAGATCAAGTCAAAATGGTCGCCCTCTCTTCGTCCCGG
CCGCCTGCCCTGGCCTGCTTGGGGGGACGGGGGAGGTTGCCCTTTTTTGCCCACCACAC
ATTTTCGCTTTGTTGTGCGGGGTTTTGCGTCTGGGTCTTATCAGATAAACGTGACCCACC
CAACCGCCACCTCAATCACCCTCCGCTAAGACACCCTGGCGCGCGCCTCTTTTCTACCCT
CCAATTTGACAACGGCAATACTGACCCGGGTTTTCTACTGCAGCTGCTGAGCTCGGCAA
GGGTTCCTTCAAGTACGCATGGGTCCTTGACAAGCTCAAGGCCGAGCGTGAGCGTGGTA
TCACCATCGACATTGCTCTGTGGAAGTTCGAGACTCCCAAGTTCTATGTCACCGTCATCG
GTAAGCTCACCCCTTGGTGGTGTCGCGCAGGCCTGCCTGCCCCACCTCTCTGCGCTGCCC
CGCCACGCGCGACGCCGCGGCTCCTGGTGCTTTTGTGCAACCCGTGCTGACGCAAATAT
CACCACAGACGCTCCCGGTCACCGTGACTTCATCAAGAACATGATCACTGGTACCTCTC
AGGCTGACTGCGCCATTCTCATCATTGCCTCCGGTACTGGTGAGTTCGAGGCTGGTATCT
CCAAGGATGGCCAGACCCGTGAGCACGCTCTGCTCGCCTACACCCTCGGTGTCAAGCAG
CTCATTGTTGCTTGCAACAAGATGGACACCGCTGGCTGGAAGCAGGACCGTTTTGAGGA
GATCAAGAAGGAGACCACCAACTTCATCAAGAAGGTCGGCTTCAACCCCAAGCAGGTC
GCTTTCGTCCCCATCTCCGGTTTCCACGGCGACAACATGCTTGAGGCCTCCCCCAACATG
CCCTGGTACAAGGGCTGGACCAAGGAGACCCAGGACAAGAAGGAGGCCAAGGGCATGA
CCCTGCTCGACGCCATTGACTCCATCGAGCCCCCCAAGCGCCCCACCGAGAAGCCCCTG
CGTCTGCCCCTCCAGGATGTTTACAAGATCGGCGGTATCGGAACTGTACCGGTCGGCCG
TATCGAGACTGGTGTCCTGAAGCCCGGTATGGTCGTCACCTTCGCCCCCTCCAACGTCAC
CACTGAGGTCAAGTCCGTGGAGATGCACCACGAGCAGCTCACCGAGGGTCAGCCCGGTG
ACAACGTTGGTTTCAACATCAAGAACGTCTCCGTGAAGGAGATTCGCCGTGGCAACGTC
GCTGGTGACTCCAAGAACGACCCCCCCTTGGGCGCCGCTTCCTTCAACGCTCAGGTCATT
GTCCTGAACCACCCCGGCCAGGTCGGTGCTGGATAC

>Melanconiella_chrysomelanconium

CACACCGCCCGTCGCTACTACCGATTGAATGGTTCAGTGAGGCGTCCGGACTGGCCCAG
GGAGGTCGGCAACGACCACCCAGGGCCGGAAAGCTCTCCAAACTCGATCATTTAGAGG
AAGTAAAAGTCGTAACAAGGTCTCCGTTGGTGAACCAGCGGAGGGATCATTGATGGAAT
ACACGTCCTATACCCCTTTGTGAACATATACCTATCTCGTTGCCTCGGCGCCAGGCCCGG
GGTGCCCCCCAGGGGGTACCCCCCACCTATGGACCTCACGGTCCCGGGCGGAGCAGGCC
CGCCGGCGGCCCCTTTAAACTCTTGTTTTTAGAACCTATCTCTTCTGAGTGATACAAAAA
ATGAATCAAAACTTTCAACAACGGATCTCTTGGCTCTGGCATCGATGAAGAACGCAGCG
AAATGCGATAAGTAATGTGAATTGCAGAATTCAGTGAATCATCGAATCTTTGAACGCAC
ATTGCGCCCGCTGGGATTCCGGCGGGCATGCCTGTTCGAGCGTCATTTCAAACCCCTCAG
GCGTCCGCGCCTGGTGTTGGGGCATAGCCTGTAAAAAGGCTAGCCCTTAAATTCAGTGG
CGTGCTCGCTGAGACTCCCGGGTGTAGTAATCTCCTTCTCGCCCGGGCAGGCTCAGCGGC
GCACTTGCCGTAAAACCCGCTATCACGCACCTTCTGAAAGTTGACCTCGGATCAGGTAG
GAATACCCGCTGAACTTAAGCATATCAATAAGCGGAGGAAAAGAAACCAACAGGGATT
GCCCTAGTAACGGCGAGTGAAGCGGCAACAGCTCAAATTTGAAATCTGGCCCCCCACGG
GGCCCGAGTTGTAATTTGCAGAGGATGTCTTCTGGCGCGGTGCCTTCCGAGTTCCCTGGA
ACGGGACGCCATAGAGGGTGAGAGCCCCGTAATGGTCGGATACCAAGCCTGTGTGAAG

CTCCTTCGACGAGTCGAGTAGTTTGGGAATGCTGCTCTAAATGGGAGGTAAATCTCTTCT
AAAGCTAAATACTGGCCAGAGACCGATAGCGCACAAGTAGAGTGATCGAAAGATGAAA
AGCACCTTGAAAAGGGGGTTAAACAGTACGTGAAATTGTTGAAAGGGAAGCGCTCATG
ACCAGACTTGCGCCGGGCGGCTCATCAGGTGGTTCTGCCCCTGTGTACTCCGTCCCGGTT
CAGGCCAGCATCGGTTCGCGCCGGGGGACAAGAACGGTGGGAACGTGGCCCTCCCTCG
GGGGGGTGTTATAGCCCGCCGTAACGATATCCTGGTGCGGACCGAGGTTCGCGCACTCC
GCAAGGATGCTGGCGTAATGGTTATCAGTGACCCGTCTTGAAACACGGACCAAGGAGTC
GTCCTTTAGAGCGAGCGTCTGGGTGTCAAAACCCGCACGCGTAATGAAAGTGAAATCAG
GTGAGAGCCCCTTGTGGGCGCATCATCGACCGATCCTGATGTTCTCGGATGGATTTGAGT
AAGAGTTTTAACGGACGGACCCGAAAGACAGTGAACTATGCTTGTATAGGGTGAAGCCA
GAGGAAACTCTGGTGGAGGCTCGCAGCGGTTCTGACGTGCAAATCGATCGTCAAATATG
AGCATGGGGGCGAAAGACTAATCGAACCACACCGCCCGTCGCTACTACCGATTGAATGG
TTCAGTGAGGCGTCCGGACTGGCCCAGGGAGGTCGGCAACGACCACCCAGGGCCGGAA
AGCTCTCCAAACTCGATCATTTAGAGGAAGTAAAAGTCGTAACAAGGTCTCCGTTGGTG
AACCAGCGGAGGGATCATTGATGGAATACACGTCCTATACCCCTTTGTGAACATATACC
TATCTCGTTGCCTCGGCGCCAGGCCCGGGGTGCCCCCCAGGGGGTACCCCCCACCTATG
GACCTCACGGTCCCGGGCGGAGCAGGCCCGCCGGCGGCCCCTTTAAACTCTTGTTTTTAG
AACCTATCTCTTCTGAGTGATACAAAAAATGAATCAAAACTTTCAACAACGGATCTCTTG
GCTCTGGCATCGATGAAGAACGCAGCGAAATGCGATAAGTAATGTGAATTGCAGAATTC
AGTGAATCATCGAATCTTTGAACGCACATTGCGCCCGCTGGGATTCCGGCGGGCATGCC
TGTTCGAGCGTCATTTCAAACCCCTCAGGCGTCCGCGCCTGGTGTTGGGGCATAGCCTGT
AAAAAGGCTAGCCCTTAAATTCAGTGGCGTGCTCGCTGAGACTCCCGGGTGTAGTAATC
TCCTTCTCGCCCGGGCAGGCTCAGCGGCGCACTTGCCGTAAAACCCGCTATCACGCACCT
TCTGAAAGTTGACCTCGGATCAGGTAGGAATACCCGCTGAACTTAAGCATATCAATAAG
CGGAGGAAAAGAAACCAACAGGGATTGCCCTAGTAACGGCGAGTGAAGCGGCAACAGC
TCAAATTTGAAATCTGGCCCCCCACGGGGCCCGAGTTGTAATTTGCAGAGGATGTCTTCT
GGCGCGGTGCCTTCCGAGTTCCCTGGAACGGGACGCCATAGAGGGTGAGAGCCCCGTAA
TGGTCGGATACCAAGCCTGTGTGAAGCTCCTTCGACGAGTCGAGTAGTTTGGGAATGCT
GCTCTAAATGGGAGGTAAATCTCTTCTAAAGCTAAATACTGGCCAGAGACCGATAGCGC
ACAAGTAGAGTGATCGAAAGATGAAAAGCACCTTGAAAAGGGGGTTAAACAGTACGTG
AAATTGTTGAAAGGGAAGCGCTCATGACCAGACTTGCGCCGGGCGGCTCATCAGGTGGT
TCTGCCCCTGTGTACTCCGTCCCGGTTCAGGCCAGCATCGGTTCGCGCCGGGGGACAAG
AACGGTGGGAACGTGGCCCTCCCTCGGGGGGGTGTTATAGCCCGCCGTAACGATATCCT
GGTGCGGACCGAGGTTCGCGCACTCCGCAAGGATGCTGGCGTAATGGTTATCAGTGACC
CGTCTTGAAACACGGACCAAGGAGTCGTCCTTTAGAGCGAGCGTCTGGGTGTCAAAACC
CGCACGCGTAATGAAAGTGAAATCAGGTGAGAGCCCCTTGTGGGCGCATCATCGACCGA
TCCTGATGTTCTCGGATGGATTTGAGTAAGAGTTTTAACGGACGGACCCGAAAGACAGT
GAACTATGCTTGTATAGGGTGAAGCCAGAGGAAACTCTGGTGGAGGCTCGCAGCGGTTC
TGACGTGCAAATCGATCGTCAAATATGAGCATGGGGGCGAAAGACTAATCGAACGGTCC
GCTCCTGGCAAAGCTGTTCCGAAACATCGTCAAGAAGCTGACCGGCGACATGATGGCCT
ACATGAAGCGTTGTGTCAGCGAGGGTAAGCATTTTGATCTAGCGTTGGGCATCCGTCAC
TCGACCCTCACGAATGGCCTCAAGTATTCTCTTGCGACTGGAAACTGGGGCGAGCAGAA
GAAGGCGTCGAGTTCCACCGCTGGTGTCTCGCAGGTGTTGAACCGGTACACCTTCTCCTC
CACCCTGTCGCATTTGCGGCGCACGAACACGCCCATCGGTCGTGACGGCAAGCTGGCCA
AGCCTCGTCAGCTGCACAATACACACTGGGGTCTCGTCTGTCCAGCAGAGACTCCCGAG
GGACAGGCTTGCGGTCTCGTCAAGAACCTCTCTTTAATGTGCTACGTTAGCGTTGGTTCA
CCAGCTGAGCCTATCAAGGACTTCATGGTGCAACGTAATATGGAAGTCTTGGAGGAATA

CGAGCCCGGCTCCAGCCCTGACGCAACCAAGATCTTCATCAACGGCACCTGGGTTGGTG
TACACAGTGAACCAGCACACCTGGTGACGCTTGTGCAGGAGCTTCGGCGCAAATGCATC
ATTTCTCATGAAGTCTCCCTCGTTCGCGACATTCGCGACCGCGAGTTCAAGATCTTCTCT
GACGCTGGTCGTGTCATGCGGCCACTCTTCGTTATCAATCAAGAGGACAATCCCGAGAC
TGGAGCGCAGCAGGGCACTCTAGCTCTCACCAAGCACCACATTCGCCGTCTCGAGGAAG
ATGCTCAGTACCAACGCAAGAAGCATGACGACGACTATTTTGGCTGGGATGGTCTTCAG
AACAGCGGTTGTATCGAGTATCTGGATGCCGAGGAAGAGGAGACATCGATGATCTGCAT
GAGTCCTGAGGATCTTGAGGACTACCGCCAGCGCAAGGCTAGGGGCAAGGATGCCAAG
GATGATGAACCGGAGGACGATGGCAGAAGTCTGAACGCTCGTGTGAAGACGAAGATTA
ACCCCGACATCCACATGTACACCCACTGCGAGATCCATCCGAGCATGCTCCTGGGAATA
TGCGCCAGCATCATTCCCTTCCCGGATCACAATCAGGTGTGTAAAATTCTCGCAGTACTA
CTATCACTCCTGCTGACCACATATATCTAGTCACCTCGCATCATCCAATCCAGCATCACC
ACATTACCATCGCATAAGCTTCAAGATCAAGTCAAAATGGTCGCCCTCTCTTCGTCCCGG
CCGCCTGCCCTGGCCTGCTTGGGGGGACGGGGGAGGTTGCCCTTTTTTTGCCCACCACAC
ATTTTCGCTTTGTTGTGCGGGGTTTTGCGTCTGGGTCTTATCAGATAAACGTGACCCACC
CAACCGCCACCTCAATCACCCTCCGCTAAGACACCCTGGCGCGCGCCTCTTTTCTACCCT
CCAATTTGACAAGGGCAATACTGACCCGGGTTTTCTACTGCAGCTGCTGAGCTCGGCAA
GGGTTCCTTCAAGTACGCATGGGTCCTTGACAAGCTCAAGGCCGAGCGTGAGCGTGGTA
TCACCATCGACATTGCTCTGTGGAAGTTCGAGACTCCCAAGTTCTATGTCACCGTCATCG
GTAAGCTCACCCCTTGGTGGTGTCGCGCAGGCCTGCCTGCCCCACCTCTCTGCGCTGCCC
CGCCACGCGCGACGCCGCGGCTCCTGGTGCTTTTGTGCAACCCGTGCTGACGCAAATAT
CACCACAGACGCTCCCGGTCACCGTGACTTCATCAAGAACATGATCACTGGTACCTCTC
AGGCTGACTGCGCCATTCTCATCATTGCCTCCGGTACTGGTGAGTTCGAGGCTGGTATCT
CCAAGGATGGCCAGACCCGTGAGCACGCTCTGCTCGCCTACACCCTCGGTGTCAAGCAG
CTCATTGTTGCTTGCAACAAGATGGACACCGCTGGCTGGAAGCAGGACCGTTTTGAGGA
GATCAAGAAGGAGACCACCAACTTCATCAAGAAGGTCGGCTTCAACCCCAAGCAGGTC
GCTTTCGTCCCCATCTCCGGTTTCCACGGCGACAACATGCTTGAGGCCTCCCCCAACATG
CCCTGGTACAAGGGCTGGACCAAGGAGACCCAGGACAAGAAGGAGGCCAAGGGCATGA
CCCTGCTCGACGCCATTGACTCCATCGAGCCCCCCAAGCGCCCCACCGAGAAGCCCCTG
CGTCTGCCCCTCCAGGATGTTTACAAGATCGGCGGTATCGGAACTGTACCGGTCGGCCG
TATCGAGACTGGTGTCCTGAAGCCCGGTATGGTCGTCACCTTCGCCCCCTCCAACGTCAC
CACTGAGGTCAAGTCCGTGGAGATGCACCACGAGCAGCTCACCGAGGGTCAGCCCGGTG
ACAACGTTGGTTTCAACATCAAGAACGTCTCCGTGAAGGAGATTCGCCGTGGCAACGTC
GCTGGTGACTCCAAGAACGACCCCCCCTTGGGCGCCGCTTCCTTCAACGCTCAGGTCATT
GTCCTGAACCACCCCGGCCAGGTCGGTGCTGGATAC

>Melanconiella_chrysorientalis

CACACCGCCCGTCGCTACTACCGATTGAATGGTTCAGTGAGGCGTCCGGACTGGCCCAG
GGAGGTCGGCAACGACCACCCAGGGCCGGAAAGTTCTCCAAACTCGATCATTTAGAGGA
AGTAAAAGTCGTAACAAGGTCTCCGTTGGTGAACCAGCGGAGGGATCATTGATGGAAA
ACACGTCCCATACCCCTTTGTGAACATATACCTATCTCGTTGCCTCGGCGCCAGGCCCGG
GGTGCCCCCCAGGGGGTACCCCCCACCTACGGACCTCACGGTCCCGGGTGGAGCAGGCC
CGCCGGCGGCCCCTCTAAACTCTTGTTTTTAAAACCTATCTCTTCTGAGTCTTGTTACCTA
GAATGAATCAAAACTTTCAACAACGGATCTCTTGGCTCTGGCATCGATGAAGAACGCAG
CGAAATGCGATAAGTAATGTGAATTGCAGAATTCAGTGAACCATCGAATCTTTGAACGC
ACATTGCGCCCGCTGGGATTCCGGCGGGCATGCCTGTTCGAGCGTCATTTCAACCCCTCA
AGCGTCCGCGCTTGGTGTTGGGGCATAGCCTGTAAAAAGGCTAGCCCTTAAATTCAGTG

GCGTGCTCGCTGAGACTCCCGGGTGTAGTAATCTCCTTCTCGCCCGGGCAGGCTCAGCG
GTGCACTTGCCGTAAAACCCGCTATCACGCAGCTTCTGAAAGTTGACCTCGGATCAGGT
AGGAATACCCGCTGAACTTAAGCATATCAATAAGCGGAGGAAAAGAAACCAACAGGGA
TTGCCCTAGTAACGGCGAGTGAAGCGGCAACAGCTCAAATTTGAAATCTGGCCCCCCAC
GGGGCCCGAGTTGTACTTTGCAGAGGATGTCTTCTGGCGCGGTGCCTTCCGAGTTCCCTG
GAACGGGACGCCACAGAGGGTGAGAGCCCCGTAATGGTCGGATACCAAGCCTGTGTGA
AGCTCCCTCGACGAGTCGAGTAGTTTGGGAATGCTGCTCTAAATGGGAGGTAAATCTCT
TCTAAAGCTAAATACCGGCCAGAGACCGATAGCGCACAAGTAGAGTGATCGAAAGATG
AAAAGCACCTTGAAAAGGGGGTTAAACAGTACGTGAAATTGTTGAAAGGGAAGCGCTC
ATGACCAGACTTGCGCCGGGCGGCTCAGCAGGTGGTTCTGCCCCTGTGTACTCCGTCCCG
GTTCAGGCCAGCATCGGTTCGCGCCGGGGGATAAGAACGGTGGGAACGTGGCCCTCCCT
CGGGGGGGTGTTATAGCCCGCCGTGACGATACCCTGGTGCGGACCGAGGTTCGCGCACT
CCGCAAGGATGCTGGCGTAATGGTTATCAGTGACCCGTCTTGAAACACGGACCAAGGAG
TCGTCCTTTAGAGCGAGCGTCTGGGTGTCAAAACCCGCACGCGTAATGAAAGTGAAATC
AGGTGAGAGCCCCCCGTGGGCGCATCATCGACCGATCCTGATGTTCTCGGATGGATTTG
AGTAAGAGTTTTAACGGACGGACCCGAAAGACAGTGAACTATGCTTGTATAGGGTGAAG
CCAGAGGAAACTCTGGTGGAGGCTCGCAGCGGTTCTGACGTGCAAATCGATCGTCAAAT
ATGAGCATGGGGGCGAAAGACTAATCGAACCACACCGCCCGTCGCTACTACCGATTGAA
TGGTTCAGTGAGGCGTCCGGACTGGCCCAGGGAGGTCGGCAACGACCACCCAGGGCCG
GAAAGTTCTCCAAACTCGATCATTTAGAGGAAGTAAAAGTCGTAACAAGGTCTCCGTTG
GTGAACCAGCGGAGGGATCATTGATGGAAAACACGTCCCATACCCCTTTGTGAACATAT
ACCTATCTCGTTGCCTCGGCGCCAGGCCCGGGGTGCCCCCCAGGGGGTACCCCCCACCT
ACGGACCTCACGGTCCCGGGTGGAGCAGGCCCGCCGGCGGCCCCTCTAAACTCTTGTTT
TTAAAACCTATCTCTTCTGAGTCTTGTTACCTAGAATGAATCAAAACTTTCAACAACGGA
TCTCTTGGCTCTGGCATCGATGAAGAACGCAGCGAAATGCGATAAGTAATGTGAATTGC
AGAATTCAGTGAACCATCGAATCTTTGAACGCACATTGCGCCCGCTGGGATTCCGGCGG
GCATGCCTGTTCGAGCGTCATTTCAACCCCTCAAGCGTCCGCGCTTGGTGTTGGGGCATA
GCCTGTAAAAAGGCTAGCCCTTAAATTCAGTGGCGTGCTCGCTGAGACTCCCGGGTGTA
GTAATCTCCTTCTCGCCCGGGCAGGCTCAGCGGTGCACTTGCCGTAAAACCCGCTATCAC
GCAGCTTCTGAAAGTTGACCTCGGATCAGGTAGGAATACCCGCTGAACTTAAGCATATC
AATAAGCGGAGGAAAAGAAACCAACAGGGATTGCCCTAGTAACGGCGAGTGAAGCGGC
AACAGCTCAAATTTGAAATCTGGCCCCCCACGGGGCCCGAGTTGTACTTTGCAGAGGAT
GTCTTCTGGCGCGGTGCCTTCCGAGTTCCCTGGAACGGGACGCCACAGAGGGTGAGAGC
CCCGTAATGGTCGGATACCAAGCCTGTGTGAAGCTCCCTCGACGAGTCGAGTAGTTTGG
GAATGCTGCTCTAAATGGGAGGTAAATCTCTTCTAAAGCTAAATACCGGCCAGAGACCG
ATAGCGCACAAGTAGAGTGATCGAAAGATGAAAAGCACCTTGAAAAGGGGGTTAAACA
GTACGTGAAATTGTTGAAAGGGAAGCGCTCATGACCAGACTTGCGCCGGGCGGCTCAGC
AGGTGGTTCTGCCCCTGTGTACTCCGTCCCGGTTCAGGCCAGCATCGGTTCGCGCCGGGG
GATAAGAACGGTGGGAACGTGGCCCTCCCTCGGGGGGGTGTTATAGCCCGCCGTGACGA
TACCCTGGTGCGGACCGAGGTTCGCGCACTCCGCAAGGATGCTGGCGTAATGGTTATCA
GTGACCCGTCTTGAAACACGGACCAAGGAGTCGTCCTTTAGAGCGAGCGTCTGGGTGTC
AAAACCCGCACGCGTAATGAAAGTGAAATCAGGTGAGAGCCCCCCGTGGGCGCATCAT
CGACCGATCCTGATGTTCTCGGATGGATTTGAGTAAGAGTTTTAACGGACGGACCCGAA
AGACAGTGAACTATGCTTGTATAGGGTGAAGCCAGAGGAAACTCTGGTGGAGGCTCGCA
GCGGTTCTGACGTGCAAATCGATCGTCAAATATGAGCATGGGGGCGAAAGACTAATCGA
ACGGTCCGCTCCTGGCAAAGCTGTTCCGAAACATCGTCAAGAAGCTGACCGGCGACATG
ATGGCCTACATGAAGCGTTGCGTCAGCGAGGGTAAGCATTTTGATCTGGCGTTGGGCAT

CCGCCACTCAACCCTCACGAATGGCCTCAAGTATTCTCTTGCGACTGGAAACTGGGGCG
AGCAGAAGAAGGCGTCGAGTTCCACCGCTGGTGTCTCGCAGGTGCTGAACCGGTACACG
TTCTCGTCCACCCTGTCGCATTTGCGGCGCACGAACACGCCCATCGGTCGTGACGGCAA
GCTGGCCAAGCCTCGTCAGCTGCACAATACACACTGGGGTCTCGTCTGTCCAGCAGAGA
CTCCCGAGGGACAGGCTTGCGGTCTCGTCAAGAACCTCTCTTTAATGTGCTACGTTAGCG
TTGGTTCACCAGCTGAGCCTATCAAGGACTTCATGGTGCAGCGCAATATGGAAGTCTTG
GAGGAATACGAGCCCGGCTCCAGCCCTGACGCAACCAAGATCTTCATCAACGGCACCTG
GGTTGGTGTACACAGTGAACCGGCACACTTGGTGACGCTTGTGCAGGAGCTTCGGCGCA
AATGCATCATTTCTCACGAAGTCTCCCTCGTCCGCGACATTCGCGACCGCGAGTTCAAGA
TCTTCTCTGACGCTGGTCGTGTCATGCGGCCACTCTTCGTTGTCAATCAAGACGATAATC
CCGAGACTGGAGCGCAGCAGGGCACTCTAGCTCTCACCAAGCACCACATTCGCCGTCTC
GAGGAAGATGCTCAGTACCATCGCAAGAAGCATGACGACGACTATTTTGGCTGGGATGG
TCTTCAGAACAGCGGTTGCATCGAGTATCTGGATGCCGAGGAAGAGGAGACAGCGATG
ATCTGCATGAGTCCTGAGGATCTTGAGGACTACCGCCAGCGCAAGGCTAGGGGCAAGGA
TGCCAAGGATGATGAACCGGAGGACGATGGCAGGAGTCTAAATGCCCGTGTGAAGACG
AAGATTAACCCCGACATCCACATGTACACCCACTGCGAGATCCATCCGAGCATGCTCCT
AGGAATATGCGCCAGCATCATTCCCTTCCCGGATCACAATCAGGTGTGTAAAATTCTCGC
AGTACTACTATCACTCCTGCTGACCACATATATCTAGTCACCTCGCATCATCCAAACCAG
CATCATCACATCACCATCGCACAAACATCAAGATGAGGTCAAAAAATGGTTGCCATGTC
TTCGTCCCGGCCGTCTGCCCTGGCCTGCTTTTGAGGGGGGGGTGGCCCTTTTTTCGCCC
ACCACGCATTTTTGCTTTGTTGTGCGGGGTTTTGCGTCTGGGTCTTATCTCAGATAAACGT
GACCCACCCAACCGCCACCTCAATCACCCTCCGCTAACACACCCTTGCGCGCGCCTCCTT
TTCACCTCCAAATTTGACAACGTCAATGCTGACCCGGGTTTTCTACTGCAGCTGCTGAGC
TCGGCAAGGGTTCCTTCAAGTACGCATGGGTCCTTGACAAGCTCAAGGCCGAGCGTGAG
CGTGGTATCACAATCGACATTGCTCTGTGGAAGTTCGAGACTCCCAAGTACTATGTCACC
GTCATTGGTAAGCTCTACCCCCTGGTGGTGTCGCGCAGGCCTGGCTGCCCAGCCTCTCCG
CGCTGCCCCGCCCCGCGCGACTCCGCGGCTCCTGGTGCTTTTGCGCAACCCGTGCTAACG
CCAATGTCACCACAGACGCTCCCGGTCACCGTGACTTCATCAAGAACATGATCACTGGT
ACCTCCCAGGCTGACTGCGCCATTCTCATCATTGCCTCCGGTACTGGTGAGTTCGAGGCT
GGTATCTCCAAGGATGGCCAGACCCGTGAGCACGCTCTGCTCGCCTACACCCTCGGTGT
CAAGCAGCTCATTGTTGCTTGCAACAAGATGGATACCGCTGGTTGGAAGCAGGACCGTT
TTGAGGAGATCAAGAAGGAAACCACCAACTTCATCAAGAAGGTCGGCTTCAACCCCAA
GCAGGTCGCCTTCGTCCCCATCTCCGGTTTCCACGGCGACAACATGCTTGAGGCCTCCGC
CAACATGCCCTGGTACAAGGGCTGGACCAAGGAGACCCAGGACAAGAAGGAGGCCAAG
GGTATGACACTGCTCGACGCCATTGACTCCATCGAGCCCCCCAAGCGCCCCACCGAGAA
GCCCCTGCGTCTGCCCCTCCAGGATGTCTACAAGATCGGCGGTATTGGAACTGTACCCGT
CGGCCGTATCGAGACTGGTATCCTGAAGCCCGGTATGGTCGTCACCTTCGCCCCCTCCAA
CGTCACCACTGAGGTCAAGTCCGTGGAGATGCACCACGAGCAGCTCACCGAGGGACAG
CCCGGTGACAACGTTGGTTTCAACGTCAAGAACGTCTCCGTCAAGGAAATTCGCCGTGG
CAACGTCGCTGGTGACTCCAAGAACGACCCCCCCTTGGGCGCCGCTTCCTTCAACGCTCA
GGTCATCGTCTTGAACCACCCCGGCCAGGTCGGTGCTGGATAC

>Melanconiella_chrysorientalis

CACACCGCCCGTCGCTACTACCGATTGAATGGTTCAGTGAGGCGTCCGGACTGGCCCAG
GGAGGTCGGCAACGACCACCCAGGGCCGGAAAGTTCTCCAAACTCGATCATTTAGAGGA
AGTAAAAGTCGTAACAAGGTCTCCGTTGGTGAACCAGCGGAGGGATCATTGATGGAAA
ACACGTCCCATACCCCTTTGTGAACATATACCTATCTCGTTGCCTCGGCGCCAGGCCCGG

GGTGCCCCCCAGGGGGTACCCCCCACCTACGGACCTCACGGTCCCGGGTGGAGCAGGCC
CGCCGGCGGCCCCTCTACACTCTTGTTTTTAAAACCTATCTCTTCTGAGTCTTGTTACCTA
GAATGAATCAAAACTTTCAACAACGGATCTCTTGGCTCTGGCATCGATGAAGAACGCAG
CGAAATGCGATAAGTAATGTGAATTGCAGAATTCAGTGAACCATCGAATCTTTGAACGC
ACATTGCGCCCGCTGGGATTCCGGCGGGCATGCCTGTTCGAGCGTCATTTCAACCCCTCA
AGCGTCCGCGCTTGGTGTTGGGGCATAGCCTGTAAAAAGGCTAGCCCTTAAATTCAGTG
GCGTGCTCGCTGAGACTCCCGGGTGTAGTAATCTCCTTCTCGCCCGGGCAGGCTCAGCG
GTGCACTTGCCGTAAAACCCGCTATCACGCAGCTTCTGAAAGTTGACCTCGGATCAGGT
AGGAATACCCGCTGAACTTAAGCATATCAATAAGCGGAGGAAAAGAAACCAACAGGGA
TTGCCCTAGTAACGGCGAGTGAAGCGGCAACAGCTCAAATTTGAAATCTGGCCCCCCAC
GGGGCCCGAGTTGTACTTTGCAGAGGATGTCTTCTGGCGCGGTGCCTTCCGAGTTCCCTG
GAACGGGACGCCACAGAGGGTGAGAGCCCCGTAATGGTCGGATACCAAGCCTGTGTGA
AGCTCCCTCGACGAGTCGAGTAGTTTGGGAATGCTGCTCTAAATGGGAGGTAAATCTCT
TCTAAAGCTAAATACCGGCCAGAGACCGATAGCGCACAAGTAGAGTGATCGAAAGATG
AAAAGCACCTTGAAAAGGGGGTTAAACAGTACGTGAAATTGTTGAAAGGGAAGCGCTC
ATGACCAGACTTGCGCCGGGCGGCTCAGCAGGTGGTTCTGCCCCTGTGTACTCCGTCCCG
GTTCAGGCCAGCATCGGTTCGCGCCGGGGGATAAGAACGGTGGGAACGTGGCCCTCCCT
CGGGGGGGTGTTATAGCCCGCCGTGACGATACCCTGGCGCGGACCGAGGTTCGCGCACT
CCGCAAGGATGCTGGCGTAATGGTTATCAGTGACCCGTCTTGAAACACGGACCAAGGAG
TCGTCCTTTAGAGCGAGCGTCTGGGTGTCAAAACCCGCACGCGTAATGAAAGTGAAATC
AGGTGAGAGCCCCCCGTGGGCGCATCATCGACCGATCCTGATGTTCTCGGATGGATTTG
AGTAAGAGTTTTAACGGACGGACCCGAAAGACAGTGAACTATGCTTGTATAGGGTGAAG
CCAGAGGAAACTCTGGTGGAGGCTCGCAGCGGTTCTGACGTGCAAATCGATCGTCAAAT
ATGAGCATGGGGGCGAAAGACTAATCGAACCACACCGCCCGTCGCTACTACCGATTGAA
TGGTTCAGTGAGGCGTCCGGACTGGCCCAGGGAGGTCGGCAACGACCACCCAGGGCCG
GAAAGTTCTCCAAACTCGATCATTTAGAGGAAGTAAAAGTCGTAACAAGGTCTCCGTTG
GTGAACCAGCGGAGGGATCATTGATGGAAAACACGTCCCATACCCCTTTGTGAACATAT
ACCTATCTCGTTGCCTCGGCGCCAGGCCCGGGGTGCCCCCCAGGGGGTACCCCCCACCT
ACGGACCTCACGGTCCCGGGTGGAGCAGGCCCGCCGGCGGCCCCTCTACACTCTTGTTTT
TAAAACCTATCTCTTCTGAGTCTTGTTACCTAGAATGAATCAAAACTTTCAACAACGGAT
CTCTTGGCTCTGGCATCGATGAAGAACGCAGCGAAATGCGATAAGTAATGTGAATTGCA
GAATTCAGTGAACCATCGAATCTTTGAACGCACATTGCGCCCGCTGGGATTCCGGCGGG
CATGCCTGTTCGAGCGTCATTTCAACCCCTCAAGCGTCCGCGCTTGGTGTTGGGGCATAG
CCTGTAAAAAGGCTAGCCCTTAAATTCAGTGGCGTGCTCGCTGAGACTCCCGGGTGTAG
TAATCTCCTTCTCGCCCGGGCAGGCTCAGCGGTGCACTTGCCGTAAAACCCGCTATCACG
CAGCTTCTGAAAGTTGACCTCGGATCAGGTAGGAATACCCGCTGAACTTAAGCATATCA
ATAAGCGGAGGAAAAGAAACCAACAGGGATTGCCCTAGTAACGGCGAGTGAAGCGGCA
ACAGCTCAAATTTGAAATCTGGCCCCCCACGGGGCCCGAGTTGTACTTTGCAGAGGATG
TCTTCTGGCGCGGTGCCTTCCGAGTTCCCTGGAACGGGACGCCACAGAGGGTGAGAGCC
CCGTAATGGTCGGATACCAAGCCTGTGTGAAGCTCCCTCGACGAGTCGAGTAGTTTGGG
AATGCTGCTCTAAATGGGAGGTAAATCTCTTCTAAAGCTAAATACCGGCCAGAGACCGA
TAGCGCACAAGTAGAGTGATCGAAAGATGAAAAGCACCTTGAAAAGGGGGTTAAACAG
TACGTGAAATTGTTGAAAGGGAAGCGCTCATGACCAGACTTGCGCCGGGCGGCTCAGCA
GGTGGTTCTGCCCCTGTGTACTCCGTCCCGGTTCAGGCCAGCATCGGTTCGCGCCGGGGG
ATAAGAACGGTGGGAACGTGGCCCTCCCTCGGGGGGGTGTTATAGCCCGCCGTGACGAT
ACCCTGGCGCGGACCGAGGTTCGCGCACTCCGCAAGGATGCTGGCGTAATGGTTATCAG
TGACCCGTCTTGAAACACGGACCAAGGAGTCGTCCTTTAGAGCGAGCGTCTGGGTGTCA

AAACCCGCACGCGTAATGAAAGTGAAATCAGGTGAGAGCCCCCGTGGGCGCATCATC
GACCGATCCTGATGTTCTCGGATGGATTTGAGTAAGAGTTTTAACGGACGGACCCGAAA
GACAGTGAACTATGCTTGTATAGGGTGAAGCCAGAGGAAACTCTGGTGGAGGCTCGCAG
CGGTTCTGACGTGCAAATCGATCGTCAAATATGAGCATGGGGGCGAAAGACTAATCGAA
CGGTCCGCTCCTGGCAAAGCTGTTCCGAAACATCGTCAAGAAGCTGACCGGCGACATGA
TGGCCTACATGAAGCGTTGCGTCAGCGAGGGTAAGCATTTTGATCTGGCGTTGGGCATC
CGCCACTCAACCCTCACGAATGGCCTCAAGTATTCTCTTGCGACTGGAAACTGGGGCGA
GCAGAAGAAGGCGTCGAGTTCCACCGCTGGTGTCTCGCAGGTGCTGAACCGGTACACGT
TCTCGTCCACCCTGTCGCATTTGCGGCGCACGAACACGCCCATCGGTCGTGACGGCAAG
CTGGCCAAGCCTCGTCAGCTGCACAATACACACTGGGGTCTCGTCTGTCCAGCAGAGAC
TCCCGAGGGACAGGCTTGCGGTCTCGTCAAGAACCTCTCTTTAATGTGCTACGTTAGCGT
TGGTTCACCAGCTGAGCCTATCAAGGACTTCATGGTGCAGCGCAATATGGAAGTCTTGG
AGGAATACGAGCCCGGCTCCAGCCCTGACGCAACCAAGATCTTCATCAACGGCACCTGG
GTTGGTGTACACAGTGAACCGGCACACTTGGTGACGCTTGTGCAGGAGCTTCGGCGCAA
ATGCATCATTTCTCACGAAGTCTCCCTCGTCCGCGACATTCGCGACCGCGAGTTCAAGAT
CTTCTCTGACGCTGGTCGTGTCATGCGGCCACTCTTCGTTGTCAATCAAGACGATAATCC
CGAGACTGGAGCGCAGCAGGGCACTCTAGCTCTCACCAAGCACCACATTCGCCGTCTCG
AGGAAGATGCTCAGTACCATCGCAAGAAGCATGACGACGACTATTTTGGCTGGGATGGT
CTTCAGAACAGCGGTTGCATCGAGTATCTGGATGCCGAGGAAGAGGAGACAGCGATGA
TCTGCATGAGTCCTGAGGATCTTGAGGACTACCGCCAGCGCAAGGCTAGGGGCAAGGAT
GCCAAGGATGATGAACCGGAGGACGATGGCAGGAGTCTAAATGCCCGTGTGAAGACGA
AGATTAACCCCGACATCCACATGTACACCCACTGCGAGATCCATCCGAGCATGCTCCTG
GGAATATGCGCCAGCATCATTCCCTTCCCGGATCACAATCAGGTGTGTAAAATTCTCGCA
GTACTACTATCACTCCTGCTGACCACATATATCTAGTCACCTCGCATCATCCAAACCAGC
ATCATCACATCACCATCGCACAAACATCAAGATGAGGTCAAAAAATGGTTGCCATGTCT
TCGTCCCGGCCGTCTGCCCTGGCCTGCTTTTGAGGGGGGGGGGGTGCCCTTTTTTCGCCC
ACCACGCATTTTTGCTTTGTTGTGCGGGGTTTTGCGTCTGGGTCTTATCTCAGATAAACGT
GACCCACCCAACCGCCACCTCAATCACCCTCCGCTAACACACCCTTGCGCGCGCCTCCTT
TTCACCTCCAAATTTGACAACGTCAATGCTGACCCGGGTTTTCTACTGCAGCTGCTGAGC
TCGGCAAGGGTTCCTTCAAGTACGCATGGGTCCTTGACAAGCTCAAGGCCGAGCGTGAG
CGTGGTATCACAATCGACATTGCTCTGTGGAAGTTCGAGACTCCCAAGTACTATGTCACC
GTCATTGGTAAGCTCTACCCCCTGGTGGTGTCGCGCAGGCCTGGCTGCCCAGCCTCTCCG
CGCTGCCCCGCCCCGCGCGACTCCGCGGCTCCTGGTGCTTTTGCGCAACCCGTGCTAACG
CCAATGTCACCACAGACGCTCCCGGTCACCGTGACTTCATCAAGAACATGATCACTGGT
ACCTCCCAGGCTGACTGCGCCATTCTCATCATTGCCTCCGGTACTGGTGAGTTCGAGGCT
GGTATCTCCAAGGATGGCCAGACCCGTGAGCACGCTCTGCTCGCCTACACCCTCGGTGT
CAAGCAGCTCATTGTTGCTTGCAACAAGATGGATACCGCTGGTTGGAAGCAGGACCGTT
TTGAGGAGATCAAGAAGGAAACCACCAACTTCATCAAGAAGGTCGGCTTCAACCCCAA
GCAGGTCGCCTTCGTCCCCATCTCCGGTTTCCACGGCGACAACATGCTTGAGGCCTCCGC
CAACATGCCCTGGTACAAGGGCTGGACCAAGGAGACCCAGGACAAGAAGGAGGCCAAG
GGTATGACACTGCTCGACGCCATTGACTCCATCGAGCCCCCCAAGCGCCCCACCGAGAA
GCCCCTGCGTCTGCCCCTCCAGGATGTCTACAAGATCGGCGGTATTGGAACTGTACCCGT
CGGCCGTATCGAGACTGGTATCCTGAAGCCCGGTATGGTCGTCACCTTCGCCCCCTCCAA
CGTCACCACTGAGGTCAAGTCCGTGGAGATGCACCACGAGCAGCTCACCGAGGGACAG
CCCGGTGACAACGTTGGTTTCAACGTCAAGAACGTCTCCGTCAAGGAAATTCGCCGTGG
CAACGTCGCTGGTGACTCCAAGAACGACCCCCCCTTGGGCGCCGCTTCCTTCAACGCTCA
GGTCATCGTCTTGAACCACCCCGGCCAGGTCGGTGCTGGATAC

>Melanconiella_chrysorientalis

ACCGATTGAATGGTTCAGTGAGGCGTCCGGACTGGCCCAGGGAGGTCGGCAACGACCAC
CCAGGGCCGGAAAGTTCTCCAAACTCGATCATTTAGAGGAAGTAAAAGTCGTAACAAGG
TCTCCGTTGGTGAACCAGCGGAGGGATCATTGATGGAAAACACGTCCCATACCCCTTTG
TGAACATATACCTATCTCGTTGCCTCGGCGCCAGGCCCGGGGTGCCCCCCAGGGGGTAC
CCCCCACCTACGGACCTCACGGTCCCGGGTGGAGCAGGCCCGCCGGCGGCCCCCCTACA
CTCTTGTTTTTAAAACCTATCTCTTCTGAGTCTTGTTACCTAGAATGAATCAAAACTTTCA
ACAACGGATCTCTTGGCTCTGGCATCGATGAAGAACGCAGCGAAATGCGATAAGTAATG
TGAATTGCAGAATTCAGTGAACCATCGAATCTTTGAACGCACATTGCGCCCGCTGGGAT
TCCGGCGGGCATGCCTGTTCGAGCGTCATTTCAACCCCTCAAGCGTCCGCGCTTGGTGTT
GGGGCATAGCCTGTAAAAAGGCTAGCCCTTAAATTCAGTGGCGTGCTCGCTGAGACTCC
CGGGTGTAGTAATCTCCTTCTCGCCCGGGCAGGCTCAGCGGTGCACTTGCCGTAAAACC
CGCTATCACGCAGCTTCTGAAAGTTGACCTCGGATCAGGTAGGAATACCCGCTGAACTT
AAGCATATCAATAAGCGGAGGAAAAGAAACCAACAGGGATTGCCCTAGTAACGGCGAG
TGAAGCGGCAACAGCTCAAATTTGAAATCTGGCCCCCCACGGGGCCCGAGTTGTACTTT
GCAGAGGATGTCTTCTGGCGCGGTGCCTTCCGAGTTCCCTGGAACGGGACGCCACAGAG
GGTGAGAGCCCCGTAATGGTCGGATACCAAGCCTGTGTGAAGCTCCCTCGACGAGTCGA
GTAGTTTGGGAATGCTGCTCTAAATGGGAGGTAAATCTCTTCTAAAGCTAAATACCGGC
CAGAGACCGATAGCGCACAAGTAGAGTGATCGAAAGATGAAAAGCACCTTGAAAAGGG
GGTTAAACAGTACGTGAAATTGTTGAAAGGGAAGCGCTCATGACCAGACTTGCGCCGGG
CGGCTCAGCAGGTGGTTCTGCCCCTGTGTACTCCGTCCCGGTTCAGGCCAGCATCGGTTC
GCGCCGGGGGATAAGAACGGTGGGAACGTGGCCCTCCCTCGGGGGGGTGTTATAGCCC
GCCGTGACGATACCCTGGTGCGGACCGAGGTTCGCGCACTCCGCAAGGATGCTGGCGTA
ATGGTTATCAGTGACCCGTCTTGAAACACGGACCAAGGAGTCGTCCTTTAGAGCGAGCG
TCTGGGTGTCAAAACCCGCACGCGTAATGAAAGTGAAATCAGGTGAGAGCCCCCCGTGG
GCGCATCATCGACCGATCCTGATGTTCTCGGATGGATTTGAGTAAGAGTTTTAACGGAC
GGACCCGAAAGACAGTGAACTATGCTTGTATAGGGTGAAGCCAGAGGAAACTCTGGTG
GAGGCTCGCAGCGGTTCTGACGTGCAAATCGATCGTCAAATATGAGCATGGGGGCGAAA
GACTAATCGAACACCGATTGAATGGTTCAGTGAGGCGTCCGGACTGGCCCAGGGAGGTC
GGCAACGACCACCCAGGGCCGGAAAGTTCTCCAAACTCGATCATTTAGAGGAAGTAAA
AGTCGTAACAAGGTCTCCGTTGGTGAACCAGCGGAGGGATCATTGATGGAAAACACGTC
CCATACCCCTTTGTGAACATATACCTATCTCGTTGCCTCGGCGCCAGGCCCGGGGTGCCC
CCCAGGGGGTACCCCCCACCTACGGACCTCACGGTCCCGGGTGGAGCAGGCCCGCCGGC
GGCCCCCCTACACTCTTGTTTTTAAAACCTATCTCTTCTGAGTCTTGTTACCTAGAATGAA
TCAAAACTTTCAACAACGGATCTCTTGGCTCTGGCATCGATGAAGAACGCAGCGAAATG
CGATAAGTAATGTGAATTGCAGAATTCAGTGAACCATCGAATCTTTGAACGCACATTGC
GCCCGCTGGGATTCCGGCGGGCATGCCTGTTCGAGCGTCATTTCAACCCCTCAAGCGTCC
GCGCTTGGTGTTGGGGCATAGCCTGTAAAAAGGCTAGCCCTTAAATTCAGTGGCGTGCT
CGCTGAGACTCCCGGGTGTAGTAATCTCCTTCTCGCCCGGGCAGGCTCAGCGGTGCACTT
GCCGTAAAACCCGCTATCACGCAGCTTCTGAAAGTTGACCTCGGATCAGGTAGGAATAC
CCGCTGAACTTAAGCATATCAATAAGCGGAGGAAAAGAAACCAACAGGGATTGCCCTA
GTAACGGCGAGTGAAGCGGCAACAGCTCAAATTTGAAATCTGGCCCCCCACGGGGCCCG
AGTTGTACTTTGCAGAGGATGTCTTCTGGCGCGGTGCCTTCCGAGTTCCCTGGAACGGGA
CGCCACAGAGGGTGAGAGCCCCGTAATGGTCGGATACCAAGCCTGTGTGAAGCTCCCTC
GACGAGTCGAGTAGTTTGGGAATGCTGCTCTAAATGGGAGGTAAATCTCTTCTAAAGCT
AAATACCGGCCAGAGACCGATAGCGCACAAGTAGAGTGATCGAAAGATGAAAAGCACC

TTGAAAAGGGGGTTAAACAGTACGTGAAATTGTTGAAAGGGAAGCGCTCATGACCAGA
CTTGCGCCGGGCGGCTCAGCAGGTGGTTCTGCCCCTGTGTACTCCGTCCCGGTTCAGGCC
AGCATCGGTTCGCGCCGGGGGATAAGAACGGTGGGAACGTGGCCCTCCCTCGGGGGGG
TGTTATAGCCCGCCGTGACGATACCCTGGTGCGGACCGAGGTTCGCGCACTCCGCAAGG
ATGCTGGCGTAATGGTTATCAGTGACCCGTCTTGAAACACGGACCAAGGAGTCGTCCTT
TAGAGCGAGCGTCTGGGTGTCAAAACCCGCACGCGTAATGAAAGTGAAATCAGGTGAG
AGCCCCCCGTGGGCGCATCATCGACCGATCCTGATGTTCTCGGATGGATTTGAGTAAGA
GTTTTAACGGACGGACCCGAAAGACAGTGAACTATGCTTGTATAGGGTGAAGCCAGAGG
AAACTCTGGTGGAGGCTCGCAGCGGTTCTGACGTGCAAATCGATCGTCAAATATGAGCA
TGGGGGCGAAAGACTAATCGAACGGTCCGCTCCTGGCAAAGCTGTTCCGAAACATCGTC
AAGAAGCTGACCGGCGACATGATGGCCTACATGAAGCGTTGCGTCAGCGAGGGTAAGC
ATTTTGATCTGGCGTTGGGCATCCGCCACTCAACCCTCACGAATGGCCTCAAGTATTCTC
TTGCGACTGGAAACTGGGGCGAGCAGAAGAAGGCGTCGAGTTCCACCGCTGGTGTCTCG
CAGGTGCTGAACCGGTACACGTTCTCGTCCACCCTGTCGCATTTGCGGCGCACGAACAC
GCCCATCGGTCGTGACGGCAAGCTGGCCAAGCCTCGTCAGCTGCACAATACACACTGGG
GTCTCGTCTGTCCAGCAGAGACTCCCGAGGGACAGGCTTGCGGTCTCGTCAAGAACCTC
TCTTTAATGTGCTACGTTAGCGTTGGTTCACCAGCTGAGCCTATCAAGGACTTCATGGTG
CAGCGCAATATGGAAGTCTTGGAGGAATACGAGCCCGGCTCCAGCCCTGACGCAACCAA
GATCTTCATCAACGGCACCTGGGTTGGTGTACACAGTGAACCGGCACACTTGGTGACGC
TTGTGCAGGAGCTTCGGCGCAAATGCATCATTTCTCACGAAGTCTCCCTCGTCCGCGACA
TTCGCGACCGCGAGTTCAAGATCTTCTCTGACGCTGGTCGTGTCATGCGGCCACTCTTCG
TTGTCAATCAAGACGATAATCCCGAGACTGGAGCGCAGCAGGGCACTCTAGCTCTCACC
AAGCACCACATTCGCCGTCTCGAGGAAGATGCTCAGTACCATCGCAAGAAGCATGACGA
CGACTATTTTGGCTGGGATGGTCTTCAGAACAGCGGTTGCATCGAGTATCTGGATGCCG
AGGAAGAGGAGACAGCGATGATCTGCATGAGTCCTGAGGATCTTGAGGACTACCGCCA
GCGCAAGGCTAGGGGCAAGGATGCCAAGGATGATGAACCGGAGGACGATGGCAGGAGT
CTAAATGCCCGTGTGAAGACGAAGATTAACCCCGACATCCACATGTACACCCACTGCGA
GATCCATCCGAGCATGCTCCTAGGAATATGCGCCAGCATCATTCCCTTCCCGGATCACAA
TCAGGTGTGTAAAATTCTCGCAGTACTACTATCACTCCTGCTGACCACATATATCTAGTC
ACCTCGTCCAAACCAGCATCATCACATCACCATCGCACAAACATCAAGATGAGGTCAAA
AAATGGTTGCCATGTCTTCGTCCCGGCCGTCTGCCCTGGCCTGCTTTTGAGGGGGGGGGG
TGGCCCTTTTTTCGCCCACCACGCATTTTTGCTTTGTTGTGCGGGGTTTTGCGTCTGGGTC
TTATCTCAGATAAACGTGACCCACCCAACCGCCACCTCAATCACCCTCCGCTAACACACC
CTTGCGCGCGCCTCCTTTTCACCTCCAAATTTGACAACGTCAATGCTGACCCGGGTTTTC
TACTGCAGCTGCTGAGCTCGGCAAGGGTTCCTTCAAGTACGCATGGGTCCTTGACAAGC
TCAAGGCCGAGCGTGAGCGTGGTATCACAATCGACATTGCTCTGTGGAAGTTCGAGACT
CCCAAGTACTATGTCACCGTCATTGGTAAGCTCTACCCCCTGGTGGTGTCGCGCAGGCCT
GGCTGCCCAGCCTCTCCGCGCTGCCCCGCCCCGCGCGACTCCGCGGCTCCTGGTGCTTTT
GCGCAACCCGTGCTAACGCCAATGTCACCACAGACGCTCCCGGTCACCGTGACTTCATC
AAGAACATGATCACTGGTACCTCCCAGGCTGACTGCGCCATTCTCATCATTGCCTCCGGT
ACTGGTGAGTTCGAGGCTGGTATCTCCAAGGATGGCCAGACCCGTGAGCACGCTCTGCT
CGCCTACACCCTCGGTGTCAAGCAGCTCATTGTTGCTTGCAACAAGATGGATACCGCTG
GTTGGAAGCAGGACCGTTTTGAGGAGATCAAGAAGGAAACCACCAACTTCATCAAGAA
GGTCGGCTTCAACCCCAAGCAGGTCGCCTTCGTCCCCATCTCCGGTTTCCACGGCGACAA
CATGCTTGAGGCCTCCGCCAACATGCCCTGGTACAAGGGCTGGACCAAGGAGACCCAGG
ACAAGAAGGAGGCCAAGGGTATGACACTGCTCGACGCCATTGACTCCATCGAGCCCCCC
AAGCGCCCCACCGAGAAGCCCCTGCGTCTGCCCCTCCAGGATGTCTACAAGATCGGCGG
TATTGGAACTGTACCCGTCGGCCGTATCGAGACTGGTATCCTGAAGCCCGGTATGGTCGT

CACCTTCGCCCCCTCCAACGTCACCACTGAGGTCAAGTCCGTGGAGATGCACCACGAGC
AGCTCACCGAGGGACAGCCCGGTGACAACGTTGGTTTCAACGTCAAGAACGTCTCCGTC
AAGGAAATTCGCCGTGGCAACGTCGCTGGTGACTCCAAGAACGACCCCCCCTTGGGCGC
CGCTTCCTTCAACGCTCAGGTCATCGTCTTGAACCACCCCGGCCAGGTCGGTGCTGGATA
C

>Melanconiella_corylina

CATTGCTGGAACACGTCCTCCAGGGCGTACCCAGATACCCTTTGTGAACTTATACCTATA
TCGTTGCCTCGGCGCTGGCCGGGGGCCCCTCCAGGGCCCCCCCACTAGGGGACCTACAC
GGTCCCTCTCAGTGGAGCAGGCCCGCCGGTGGCCCTATAAACTCTTGTTTTATAACGTA
TCTCCTCTGAGTAACGAAACTATAAATGAATCAAAACTTTCAACAACGGATCTCTTGGTT
CTGGCATCGATGAAGAACGCAGCGAAATGCGATAAGTAATGTGAATTGCAGAATTCAGT
GAATCATCGAATCTTTGAACGCACATTGCGCCCGCTGGTATTCCGGCGGGCATGCCTGTT
CGAGCGTCATTTCAACCCTCAAGCTTTACCGCTTGGTGTTGGGGCGTAGCCTGTAGAAGG
CTAGCCCTGAAATTCAGTGGCGTGCTCGCTAAGACTCCGAGCGTAGTAATTTTTTCTCGC
TTAGGTTGGATTAGCGGTGCTCAGCCGTAAAACCTCGGCCCGAGTTGTAATTTGCAGAG
GATGCTTCTGGCGCGGTGCCTTCCGAGTTCCCTGGAACGGGACGCCACAGAGGGTGAGA
GCCCCGTATGGTCGGACACCAAGCCTGTGTGAAGCTCCTTCGACGAGTCGAGTAGTTTG
GGAATGCTGCTCAAAATGGGAGGTAAATCTCTTCTAAAGCTAAATACTGGCCAGAGACC
GATAGCGCACAAGTAGAGTGATCGAAAGATGAAAAGCACCTTGAAAAGGGGGTTAAAC
AGTACGTGAAATTGTTGAAAGGGAAGCGCTTGTGACCAGACTTGCGCCGGGCGGCTCAT
CTGGGGTTCTCCCCTGTGTACTCCGCTCGGTTCAGGCCAGCATCGGTTCTCGCTGGGGGA
TAAGAACGGTGGGAACGTGGCCCTCCCTCGGGGGGGTGTTATAGCCCGCCGTACGATAC
CCTAGCGGGGACCGAGGTTCGCGCTCCGCAAGGATGCTGGCGTAATGGTCATCAGCGAC
CCGTCTTACCGGTGACATGATGGCGTACATGAAGCGTTGTGTCAGCGAGGGCAAGCATT
TCGATCTGGCTTTGGGCATCCGCCACTCCACCCTCACAAATGGTCTCAAGTACTCTCTTG
CGACTGGAAATTGGGGCGAGCAGAAGAAGGCGATGAGTTCCACCGCTGGTGTCTCGCA
GGTGCTGAACCGGTACACATTTTCGTCTACCCTTTCGCATTTGCGGCGCACGAACACGCC
CATTGGTCGTGACGGCAAGCTGGCCAAGCCTCGTCAGCTGCACAACACTCACTGGGGCC
TCGTTTGTCCAGCAGAGACTCCTGAGGGTCAGGCTTGCGGTCTTGTCAAGAACCTGTCTT
TAATGTGCTACGTCAGCGTTGGCTCACCGGCCGAGCCTATCAAGGACTTCATGGTGCAG
CGGAATATGGAGGTTTTGGAGGAATACGAGCCCGGCTCCAGCCCTGACGCGACCAAGAT
ATTCATCAACGGCACATGGGTTGGTGTCCACAACGAGCCAGCACACTTGGTCACGCTTG
TGCAAGAGCTTCGACGCAAATGCATCATTTCTCACGAAGTCTCCCTCGTTCGCGACATTC
GCGATCGCGAGTTCAAGATCTTCTCCGACGCTGGTCGTGTGATGCGGCCTCTCTTTGTCG
TCAACCAAGAGGACAACCCCGAGACTGGAGCCCAGCAGGGCACTCTCGCCCTCACCAA
GCACCACATTCGCCGTCTCGAGGAAGATGCTCAGTACCAGCGCAAGAAGCATGATGAAG
ACTATTTTGGCTGGGATGGTCTTCAGAACAGCGGCTGTATCGAGTACCTGGATGCCGAG
GAAGAGGAGACAGCAATGATCTGCATGAGTCCTGAGGATCTTGAAGACTACCGGCAGC
GCAAGGCTAGGGGCAAGGATGCCAAGGATGATGAGCCTGAGGATGATGGCAGGAGTCT
GAACGCTCGTGTGAAGACGAAGATCAACACCGACATCCACATGTACACCCACTGCGAGA
TCCATCCGAGCATGCTCCTGGGAATCTGCGCCAGCCATCATTATCACCATCGCAAAAAC
ACAAAAGGCAGACGGTCGCGATTTCTTCATCGCGTCGCGACCGTTGGCCTTGGCAGGCT
GTGTCCTTTTTTCACCCATCGCACATTTTCGCTTTGTGGTGCGGGGTTTTGCGTCTGTGTC
TTATCTCAGATAAACGTGACCCACCCAACCACCACCTCCATCATCCTCTGCCAACGCACC
CCGGATGCACCACTGGCCCTCACCATGACAACGACGATGCTAACTTAATTCTTCTATCAC

AGCTGCTGAGCTCGGCAAGGGTTCCTTCAAGTACGCATGGGTCCTTGACAAGCTCAAGG
CCGAGCGTGAGCGTGGTATCACCATCGACATTGCTCTGTGGAAGTTCGAGACTCCCAAG
TACTATGTCACCGTCATTGGTAAGCTTGGCCCCTCTGGACCATGTCGCGCAGGCCTGGCT
GCCCCACCCATCTGCGCTGCCCCGCCATGGGCGACACCGCGGCTTGTTACACCTTTTGCG
CAACCCTTGCTAACGCAAACGTCGCCACAGACGCTCCCGGTCACCGTGACTTCATCAAG
AACATGATCACTGGTACCTCCCAGGCTGACTGCGCCGTTCTCATCATCGCCTCCGGTACT

>Melanconiella_corylina

CATTGCTGGAACACGTCCTCCAGGGCGTACCCAGATACCCTTTGTGAACTTATACCTATA
TCGTTGCCTCGGCGCTGGCCGGGGGCCCCTCCAGGGCCCCCCCACTAGGGGACCTACAC
GGTCCCTCTCAGTGGAGCAGGCCCGCCGGTGGCCCTATAAACTCTTGTTTTTATAACGTA
TCTCCTCTGAGTAACGAAACTATAAATGAATCAAAACTTTCAACAACGGATCTCTTGGTT
CTGGCATCGATGAAGAACGCAGCGAAATGCGATAAGTAATGTGAATTGCAGAATTCAGT
GAATCATCGAATCTTTGAACGCACATTGCGCCCGCTGGTATTCCGGCGGGCATGCCTGTT
CGAGCGTCATTTCAACCCTCAAGCTTTACCGCTTGGTGTTGGGGCGTAGCCTGTAGAAGG
CTAGCCCTGAAATTCAGTGGCGTGCTCGCTAAGACTCCGAGCGTAGTAATTTTTCTCGC
TTAGGTTGGATTAGCGGTGCTCAGCCGTAAAACCTCGGCCCGAGTTGTAATTTGCAGAG
GATGCTTCTGGCGCGGTGCCTTCCGAGTTCCCTGGAACGGGACGCCACAGAGGGTGAGA
GCCCCGTATGGTCGGACACCAAGCCTGTGTGAAGCTCCTTCGACGAGTCGAGTAGTTTG
GGAATGCTGCTCAAAATGGGAGGTAAATCTCTTCTAAAGCTAAATACTGGCCAGAGACC
GATAGCGCACAAGTAGAGTGATCGAAAGATGAAAAGCACCTTGAAAAGGGGGTTAAAC
AGTACGTGAAATTGTTGAAAGGGAAGCGCTTGTGACCAGACTTGCGCCGGGCGGCTCAT
CTGGGGTTCTCCCCTGTGTACTCCGCTCGGTTCAGGCCAGCATCGGTTCTCGCTGGGGGA
TAAGAACGGTGGGAACGTGGCCCTCCCTCGGGGGGGTGTTATAGCCCGCCGTACGATAC
CCTAGCGGGGACCGAGGTTCGCGCTCCGCAAGGATGCTGGCGTAATGGTCATCAGCGAC
CCGTCTTACCGGTGACATGATGGCGTACATGAAGCGTTGTGTCAGCGAGGGCAAGCATT
TCGATCTGGCTTTGGGCATCCGCCACTCCACCCTCACAAATGGTCTCAAGTACTCTCTTG
CGACTGGAAATTGGGGCGAGCAGAAGAAGGCGATGAGTTCCACCGCTGGTGTCTCGCA
GGTGCTGAACCGGTACACATTTTCGTCTACCCTTTCGCATTTGCGGCGCACGAACACGCC
CATTGGTCGTGACGGCAAGCTGGCCAAGCCTCGTCAGCTGCACAACACTCACTGGGGCC
TCGTTTGTCCAGCAGAGACTCCTGAGGGTCAGGCTTGCGGTCTTGTCAAGAACCTGTCTT
TAATGTGCTACGTCAGCGTTGGCTCACCGGCCGAGCCTATCAAGGACTTCATGGTGCAG
CGGAATATGGAGGTTTTGGAGGAATACGAGCCCGGCTCCAGCCCTGACGCGACCAAGAT
ATTCATCAACGGCACATGGGTTGGTGTCCACAACGAGCCAGCACACTTGGTCACGCTTG
TGCAAGAGCTTCGACGCAAATGCATCATTTCTCACGAAGTCTCCCTCGTTCGCGACATTC
GCGATCGCGAGTTCAAGATCTTCTCCGACGCTGGTCGTGTGATGCGGCCTCTCTTTGTCG
TCAACCAAGAGGACAACCCCGAGACTGGAGCCCAGCAGGGCACTCTCGCCCTCACCAA
GCACCACATTCGCCGTCTCGAGGAAGATGCTCAGTACCAGCGCAAGAAGCATGATGAAG
ACTATTTTGGCTGGGATGGTCTTCAGAACAGCGGCTGTATCGAGTACCTGGATGCCGAG
GAAGAGGAGACAGCAATGATCTGCATGAGTCCTGAGGATCTTGAAGACTACCGGCAGC
GCAAGGCTAGGGGCAAGGATGCCAAGGATGATGAGCCTGAGGATGATGGCAGGAGTCT
GAACGCTCGTGTGAAGACGAAGATCAACACCGACATCCACATGTACACCCACTGCGAGA
TCCATCCGAGCATGCTCCTGGGAATCTGCGCCAGCCATCATTATCACCATCGCAAAAAC
ACAAAAGGCAGACGGTCGCGATTTCTTCATCGCGTCGCGACCGTTGGCCTTGGCAGGCT
GTGTCCTTTTTTCACCCATCGCACATTTTCGCTTTGTGGTGCGGGGTTTTGCGTCTGTGTC
TTATCTCAGATAAACGTGACCCACCCAACCACCACCTCCATCATCCTCTGCCAACGCACC
CCGGATGCACCACTGGCCCTCACCATGACAACGACGATGCTAACTTAATTCTTCTATCAC

AGCTGCTGAGCTCGGCAAGGGTTCCTTCAAGTACGCATGGGTCCTTGACAAGCTCAAGG
CCGAGCGTGAGCGTGGTATCACCATCGACATTGCTCTGTGGAAGTTCGAGACTCCCAAG
TACTATGTCACCGTCATTGGTAAGCTTGGCCCCTCTGGACCATGTCGCGCAGGCCTGGCT
GCCCCACCCATCTGCGCTGCCCCGCCATGGGCGACACCGCGGCTTGTTACACCTTTTGCG
CAACCCTTGCTAACGCAAACGTCGCCACAGACGCTCCCGGTCACCGTGACTTCATCAAG
AACATGATCACTGGTACCTCCCAGGCTGACTGCGCCGTTCTCATCATCGCCTCCGGTACT

>Melanconiella_decorahensis

CACACCGCCCGTCGCTACTACCGATTGAATGGTTCAGTGAGGCGTCCGGACTGGCCCAG
GGAGGTCGGCAACGACCACCCAGGGCCGGAAAGTTCTCCAAACTCGATCATTTAGAGGA
AGTAAAAGTCGTAACAAGGTCTCCGTTGGTGAACCAGCGGAGGGATCATTGATGGAACA
CACGTCCCATACCCCTTTGTGAACTTATACCTATATCTCGTTGCCTCGGCGCCAGGCCTG
GGGTTCCCCCTCGGGGGTTCCCCCAGCCAGTGGACTCCAGCGGTCCCGGTTGGAGCAG
GCCCGCCGGTGGCCTCCCGAAACTCTTGTTTTTAGAACCTATCCTCTGACATACTTAAAA
AAAAAAAAAATGAATCAAAACTTTCAACAACGGATCTCTTGGCTCTGGCATCGATGAAG
AACGCAGCGAAATGCGATAAGTAATGTGAATTGCAGAATTCAGTGAATCATCGAATCTT
TGAACGCACATTGCGCCCGCTGGAATTCCGGCGGGCATGCCTGTTCGAGCGTCATTTCA
ACCCCTCAAGCCCCCGTGCTTGGTGTTGGGGCCTAGCCTGTAGAAAGGCTAGCCCTTAA
ATTCAGTGGCGTGCTCGCTGAGACTCCCGGGTGTAGTAATCTCCTTCTCACCTGGGCTGG
CTCGGCGGTGCACTTGCCGTAAAACCCGCTATCACGCACCTTCTGAAAGTTGACCTCGG
ATCAGGTAGGAATACCCGCTGAACTTAAGCATATCAATAAGCGGAGGAAAAGAAACCA
ACAGGGATTGCCCTAGTAACGGCGAGTGAAGCGGCAACAGCTCAAATTTGAAATCTGGT
CCCCCCTGGGGGCCCGAGTTGTAATTTGCAGAGGATGTCTTCTGGCGCGGTGCCTTCCGA
GTTCCCTGGAACGGGACGCCACAGAGGGTGAGAGCCCCGTAATGGTCGGATACCAAGC
CTGTGTGAAGCTCCTTCGACGAGTCGAGTAGTTTGGGAATGCTGCTCTAAATGGGAGGT
AAATCTCTTCTAAAGCTAAATACTGGCCAGAGACCGATAGCGCACAAGTAGAGTGATCG
AAAGATGAAAAGCACCTTGAAAAGGGGGTTAAACAGTACGTGAAATTGTTGAAAGGGA
AGCGCTCATGACCAGACTTGCGCCGGGCGGCTCAGCAGGTGGTTCTGCCCCTGTGTACT
CCGTCCCGGTTCAGGCCAGCATCGGTTCTTGCCGGGGGATAAGAACGATAGGAACGTGG
CCCTCCCTCGGGGGGGTGTTATAGCCCGTCGTAACGATACCCTGGCGGGGACCGAGGTT
CGCGCATTCGCAAGGATGCTGGCGTAATGGTTATCAGTGACCCGTCTTGAAACACGGAC
CAAGGAGTCGTCCTTTAGAGCGAGCGTCTGGGTGTTAAAACCCGCACGCGTAATGAAAG
TGAAATTAGGTGAGAGCCCTCACGGGCGCATCATCGACCGATCCTGATGTTCTCGGACG
GATTTGAGTAAGAGTTTTAACGGACGGACCCGAAAGACAGTGAACTATGCTTGTATAGG
GTGAAGCCAGAGGAAACTCTGGTGGAGGCTCGCAGCGGTTCTGACGTGCAAATCGATCG
TCAAATATGAGCATGGGGGCGAAAGACTAATCGAACACACCGCCCGTCGCTACTACCGA
TTGAATGGTTCAGTGAGGCGTCCGGACTGGCCCAGGGAGGTCGGCAACGACCACCCAGG
GCCGGAAAGTTCTCCAAACTCGATCATTTAGAGGAAGTAAAAGTCGTAACAAGGTCTCC
GTTGGTGAACCAGCGGAGGGATCATTGATGGAACACACGTCCCATACCCCTTTGTGAAC
TTATACCTATATCTCGTTGCCTCGGCGCCAGGCCTGGGGTTCCCCCTCGGGGGTTCCCCC
CAGCCAGTGGACTCCAGCGGTCCCGGTTGGAGCAGGCCCGCCGGTGGCCTCCCGAAACT
CTTGTTTTTAGAACCTATCCTCTGACATACTTAAAAAAAAAAAAAAATGAATCAAAACTTT
CAACAACGGATCTCTTGGCTCTGGCATCGATGAAGAACGCAGCGAAATGCGATAAGTAA
TGTGAATTGCAGAATTCAGTGAATCATCGAATCTTTGAACGCACATTGCGCCCGCTGGA
ATTCCGGCGGGCATGCCTGTTCGAGCGTCATTTCAACCCCTCAAGCCCCCGTGCTTGGTG
TTGGGGCCTAGCCTGTAGAAAGGCTAGCCCTTAAATTCAGTGGCGTGCTCGCTGAGACT

CCCGGGTGTAGTAATCTCCTTCTCACCTGGGCTGGCTCGGCGGTGCACTTGCCGTAAAAC
CCGCTATCACGCACCTTCTGAAAGTTGACCTCGGATCAGGTAGGAATACCCGCTGAACT
TAAGCATATCAATAAGCGGAGGAAAAGAAACCAACAGGGATTGCCCTAGTAACGGCGA
GTGAAGCGGCAACAGCTCAAATTTGAAATCTGGTCCCCCCTGGGGGCCCGAGTTGTAAT
TTGCAGAGGATGTCTTCTGGCGCGGTGCCTTCCGAGTTCCCTGGAACGGGACGCCACAG
AGGGTGAGAGCCCCGTAATGGTCGGATACCAAGCCTGTGTGAAGCTCCTTCGACGAGTC
GAGTAGTTTGGGAATGCTGCTCTAAATGGGAGGTAAATCTCTTCTAAAGCTAAATACTG
GCCAGAGACCGATAGCGCACAAGTAGAGTGATCGAAAGATGAAAAGCACCTTGAAAAG
GGGGTTAAACAGTACGTGAAATTGTTGAAAGGGAAGCGCTCATGACCAGACTTGCGCCG
GGCGGCTCAGCAGGTGGTTCTGCCCCTGTGTACTCCGTCCCGGTTCAGGCCAGCATCGGT
TCTTGCCGGGGGATAAGAACGATAGGAACGTGGCCCTCCCTCGGGGGGGTGTTATAGCC
CGTCGTAACGATACCCTGGCGGGGACCGAGGTTCGCGCATTCGCAAGGATGCTGGCGTA
ATGGTTATCAGTGACCCGTCTTGAAACACGGACCAAGGAGTCGTCCTTTAGAGCGAGCG
TCTGGGTGTTAAAACCCGCACGCGTAATGAAAGTGAAATTAGGTGAGAGCCCTCACGGG
CGCATCATCGACCGATCCTGATGTTCTCGGACGGATTTGAGTAAGAGTTTTAACGGACG
GACCCGAAAGACAGTGAACTATGCTTGTATAGGGTGAAGCCAGAGGAAACTCTGGTGG
AGGCTCGCAGCGGTTCTGACGTGCAAATCGATCGTCAAATATGAGCATGGGGGCGAAAG
ACTAATCGAAGGTCCGCTCCTGGCAAAGCTGTTCCGAAACATCGTCAAGAAGCTGACCG
GCGACATGATGGCGTATATGAAGCGTTGCGTTAGCGAGGGCAAGCATTTTGATCTGGCG
TTAGGCATCCGCCACTCAACGCTCACAAATGGCCTCAAGTACTCTCTCGCGACTGGAAA
CTGGGGTGAGCAGAAGAAGGCGATGAGTTCCACCGCTGGTGTCTCGCAGGTGCTGAATC
GGTACACCTTCTCGTCCACCCTATCGCATTTGCGGCGCACGAACACGCCCATCGGTCGTG
ACGGCAAGCTGGCCAAGCCTCGTCAGCTGCACAACACACACTGGGGTCTCGTCTGTCCA
GCAGAGACTCCCGAGGGACAGGCTTGTGGTCTCGTCAAGAACCTGTCTCTAATGTGCTA
CGTTAGTGTTGGCTCACCGGCTGAGCCTATCAAGGACTTCATGGTGCAGCGTAATATGG
AAGTCTTGGAGGAATACGAACCCGGTTCCAGCCCTGACGCAACCAAGATCTTCATCAAC
GGCACGTGGGTTGGTGTGCACAGTGAACCAGCACACTTGGTCACACTTGTGCAGGAGCT
TCGGCGCAAATGCATCATTTCTCACGAAGTCTCCCTCGTTCGCGACATTCGCGACCGTGA
GTTCAAGATCTTCTCCGACGCTGGTCGTGTCATGCGGCCTCTCTTCGTTGTCAATCAAGA
GGATAATCCCGAGACTGGAGCGCAGCAGGGCACTCTGGCTCTCACCAAACACCACATTC
GCCGTCTCGAGGAAGATGCTCAGTACCATCGCAAGAAGCATGATGACGACTATTTCGGA
TGGGATGGGCTTCAGAACAGCGGTTGCATCGAGTACCTGGATGCCGAGGAAGAGGAGA
CAGCGATGATCTGCATGAGTCCTGAGGATCTAGAGGACTACCGCCAGCGCAAGGCTAGG
GGCAAGGATGCCAAGGATGATGAGCCGGAGGAGGATGGCAGGAGTCTGAACGCTCGTG
TGAAGACGAAGATCAACCCCGACATCCACATGTATACCCACTGCGAGATCCATCCGAGC
ATGCTCCTGGGAATATGCGCCAGTATCATTCCCTTTCCGGATCACAATCAGGTGTGTAAA
ATTCTTGCAGCACTAATATCACCCCTGCTGACCACATATATCTAGTCACCTCGCATCAAT
CAAATCAGCATCATCACAAGTGCGCAAACATCAAAATCAAGGCAAATGGTCGCTATCTT
TTCGTCGCGGCCGTTTGCCTTGGGCCCCTGAGCGCATGGCCTTTTTTCGCCCACTGCACA
TTTTCGCTTTGTGGTGCGGGGTTTTGCGTCTGGGTCTTATCTCAGATAAACGTGACCCAC
TCAACCGCCACCTCCATCATGCTCCGCCGCCAGCGCACCATTGCGCGCGCCTCTCTTTGA
CCCTCACATTTTGAACAACTACAATGCTGACGCGGATTTTCCTACTGCAGCTGCTGAACT
CGGCAAGGGTTCCTTCAAGTACGCATGGGTCCTTGACAAGCTCAAGGCCGAGCGTGAGC
GTGGTATCACCATCGACATTGCTCTGTGGAAGTTCGAGACTCCCAAGTACTATGTCACCG
TCATTGGTAAGCTCTACCACCCCCCCACCCCGTCCTGATGATTTCTCGCAGGCGTGGCTG
CCACGGCCCGCTGCGCTCCCCTTCCGACGCGATAGCGCGGTTGATGATGATTTTGCGCCC
CCCATGCTAACGCAAATGCCACCACAGACGCTCCCGGTCACCGTGACTTCATCAAGAAC
ATGATCACTGGTACCTCCCAGGCTGACTGCGCCATTCTCATCATTGCCTCCGGTACTGGT

GAGTTCGAGGCTGGTATCTCCAAGGATGGCCAGACCCGTGAGCACGCTCTGCTCGCCTA
CACCCTCGGTGTCAAGCAGCTCATTGTTGCTTGCAACAAGATGGATACCGCTGGCTGGA
AGCAGGACCGTTTTGAGGAGATCAAGAAGGAGACGACCAACTTCATCAAGAAGGTCGG
CTTCAACCCCAAGCAGGTTGCTTTCGTCCCCATCTCCGGCTTCCACGGCGACAACATGCT
TGAGGCCTCCGCCAACATGCCCTGGTACAAGGGCTGGACCAAGGAGACCCAGGACAAG
AAGGAGGCCAAGGGCACGACCCTGCTCGACGCCATTGACGCCATTGAGCCCCCCAAGCG
CCCCACCGAGAAGCCCCTGCGTCTGCCCCTCCAGGATGTCTACAAGATCGGCGGTATTG
GAACGGTACCTGTCGGCCGTATCGAGACTGGTGTCCTTAAGCCCGGTATGGTCGTTACCT
TCGCCCCCTCCAACGTCACCACTGAAGTCAAGTCCGTGGAAATGCACCACGAGCAGCTC
ACCGAGGGTCAGCCCGGTGACAACGTTGGCTTCAACGTCAAGAACGTCTCCGTCAAGGA
GATCCGCCGTGGCAACGTCGCTGGTGACTCCAAGAACGACCCCCCACTGGCCGCCGCTT
CCTTCAACGCCCAGGTCATCGTTCTGAACCACCCCGGCCAGGTTGGTGCTGGATAC

>Melanconiella_decorahensis

CACACCGCCCGTCGCTACTACCGATTGAATGGTTCAGTGAGGCGTCCGGACTGGCCCAG
GGAGGTCGGCAACGACCACCCAGGGCCGGAAAGTTCTCCAAACTCGATCATTTAGAGGA
AGTAAAAGTCGTAACAAGGTCTCCGTTGGTGAACCAGCGGAGGGATCATTGATGGAACA
TACGTCCCATACCCCTTTGTGAACTTATACCTATATCTCGTTGCCTCGGCGCCAGGCCTG
GGGTTCCCCCTCGGGGGTTCCCCCCAGCCAGTGGACTCCAGCGGTCCCGGTTGGAGCAG
GCCCGCCGGTGGCCTCCCGAAACTCTTGTTTTTAGAACCTATCCTCTGAGATACTTAAAA
AAAAATGAATCAAAACTTTCAACAACGGATCTCTTGGCTCTGGCATCGATGAAGAACGC
AGCGAAATGCGATAAGTAATGTGAATTGCAGAATTCAGTGAATCATCGAATCTTTGAAC
GCACATTGCGCCCGCTGGAATTCCGGCGGGCATGCCTGTTCGAGCGTCATTTCAACCCCT
CAAGCCCCCGTGCTTGGTGTTGGGGCCTAGCCTGTAGAAAGGCTAGCCCTGAAATTCAG
TGGCGTGCTCGCTGAGACTCCCGGGTGTAGTAATCTCCTTCTCACCTAGGCTGGCTCGGC
GGTGCACTTGCCGTAAAACCCGCTATCACGCACCTTCTGAAAGTTGACCTCGGATCAGG
TAGGAATACCCGCTGAACTTAAGCATATCAATAAGCGGAGGAAAAGAAACCAACAGGG
ATTGCCCTAGTAACGGCGAGTGAAGCGGCAACAGCTCAAATTTGAAATCTGGTCCCCCC
TGGGGGCCCGAGTTGTAATTTGCAGAGGATGTTTCTGGCGCGGTGCCTTCCGAGTTCCC
TGGAACGGGACGCCACAGAGGGTGAGAGCCCCGTAATGGTCGGATACCAAGCCTGTGT
GAAGCTCCTTCGACGAGTCGAGTAGTTTGGGAATGCTGCTCTAAATGGGAGGTAAATCT
CTTCTAAAGCTAAATACTGGCCAGAGACCGATAGCGCACAAGTAGAGTGATCGAAAGAT
GAAAAGCACCTTGAAAAGGGGGTTAAACAGTACGTGAAATTGTTGAAAGGGAAGCGCT
CATGACCAGACTTGCGCCGGGCGGCTCAGCAGGCGGTTCTGCCCCTGTGTACTCCGTCCC
GGTTCAGGCCAGCATCGGTTCTTGCCGGGGGATAAGAACGATAGGAACGTGGCCCTCCC
TCGGGGGGGTGTTATAGCCCGTCGTAACGATACCCTGGCGGGGWCCGAGGTTCGCGCAT
TCGCAAGGATGCTGGCGTAATGGTTATCAGTGACCCGTCTTGAAACACGGACCAAGGAG
TCGTCCTTTAGAGCGAGCGTCTGGGTGTTAAAACCCGCACGCGTAATGAAAGTGAAATT
AGGTGAGAGCCCTCACGGGCGCATCATCGACCGATCCTGATGTTCTCGGACGGATTTGA
GTAAGAGTTTTAACGGACGGACCCGAAAGACAGTGAACTATGCTTGTATAGGGTGAAGC
CAGAGGAAACTCTGGTGGAGGCTCGCAGCGGTTCTGACGTGCAAATCGATCGTCAAATA
TGAGCATGGGGGCGAAAGACTAATCGAACCACACCGCCCGTCGCTACTACCGATTGAAT
GGTTCAGTGAGGCGTCCGGACTGGCCCAGGGAGGTCGGCAACGACCACCCAGGGCCGG
AAAGTTCTCCAAACTCGATCATTTAGAGGAAGTAAAAGTCGTAACAAGGTCTCCGTTGG
TGAACCAGCGGAGGGATCATTGATGGAACATACGTCCCATACCCCTTTGTGAACTTATA
CCTATATCTCGTTGCCTCGGCGCCAGGCCTGGGGTTCCCCCTCGGGGGTTCCCCCCAGCC

AGTGGACTCCAGCGGTCCCGGTTGGAGCAGGCCCGCCGGTGGCCTCCCGAAACTCTTGT
TTTTAGAACCTATCCTCTGAGATACTTAAAAAAAAATGAATCAAAACTTTCAACAACGG
ATCTCTTGGCTCTGGCATCGATGAAGAACGCAGCGAAATGCGATAAGTAATGTGAATTG
CAGAATTCAGTGAATCATCGAATCTTTGAACGCACATTGCGCCCGCTGGAATTCCGGCG
GGCATGCCTGTTCGAGCGTCATTTCAACCCCTCAAGCCCCCGTGCTTGGTGTTGGGGCCT
AGCCTGTAGAAAGGCTAGCCCTGAAATTCAGTGGCGTGCTCGCTGAGACTCCCGGGTGT
AGTAATCTCCTTCTCACCTAGGCTGGCTCGGCGGTGCACTTGCCGTAAAACCCGCTATCA
CGCACCTTCTGAAAGTTGACCTCGGATCAGGTAGGAATACCCGCTGAACTTAAGCATAT
CAATAAGCGGAGGAAAAGAAACCAACAGGGATTGCCCTAGTAACGGCGAGTGAAGCGG
CAACAGCTCAAATTTGAAATCTGGTCCCCCCTGGGGGCCCGAGTTGTAATTTGCAGAGG
ATGTTTTCTGGCGCGGTGCCTTCCGAGTTCCCTGGAACGGGACGCCACAGAGGGTGAGA
GCCCCGTAATGGTCGGATACCAAGCCTGTGTGAAGCTCCTTCGACGAGTCGAGTAGTTT
GGGAATGCTGCTCTAAATGGGAGGTAAATCTCTTCTAAAGCTAAATACTGGCCAGAGAC
CGATAGCGCACAAGTAGAGTGATCGAAAGATGAAAAGCACCTTGAAAAGGGGGTTAAA
CAGTACGTGAAATTGTTGAAAGGGAAGCGCTCATGACCAGACTTGCGCCGGGCGGCTCA
GCAGGCGGTTCTGCCCCTGTGTACTCCGTCCCGGTTCAGGCCAGCATCGGTTCTTGCCGG
GGGATAAGAACGATAGGAACGTGGCCCTCCCTCGGGGGGGTGTTATAGCCCGTCGTAAC
GATACCCTGGCGGGGWCCGAGGTTCGCGCATTCGCAAGGATGCTGGCGTAATGGTTATC
AGTGACCCGTCTTGAAACACGGACCAAGGAGTCGTCCTTTAGAGCGAGCGTCTGGGTGT
TAAAACCCGCACGCGTAATGAAAGTGAAATTAGGTGAGAGCCCTCACGGGCGCATCATC
GACCGATCCTGATGTTCTCGGACGGATTTGAGTAAGAGTTTTAACGGACGGACCCGAAA
GACAGTGAACTATGCTTGTATAGGGTGAAGCCAGAGGAAACTCTGGTGGAGGCTCGCAG
CGGTTCTGACGTGCAAATCGATCGTCAAATATGAGCATGGGGGCGAAAGACTAATCGAA
CGGTCCGCTCCTGGCAAAGCTGTTCCGAAACATCGTCAAGAAGCTGACCGGCGACATGA
TGGCGTATATGAAGCGTTGCGTTAGCGAGGGCAAGCATTTTGATCTGGCGTTAGGCATC
CGCCACTCAACGCTCACAAATGGCCTCAAGTACTCTCTCGCGACTGGAAACTGGGGTGA
GCAGAAGAAGGCGATGAGTTCCACCGCTGGTGTCTCGCAGGTGCTGAATCGGTACACCT
TCTCGTCCACCCTATCGCATTTGCGGCGCACGAACACGCCCATCGGTCGTGACGGAAAG
CTGGCCAAGCCTCGTCAGCTGCACAACACACACTGGGGTCTCGTCTGTCCAGCAGAGAC
TCCCGAGGGACAGGCTTGTGGTCTCGTCAAGAACCTGTCTCTAATGTGCTACGTTAGTGT
TGGCTCACCGGCTGAGCCTATCAAGGACTTCATGGTGCAGCGTAATATGGAAGTCTTGG
AGGAATACGAACCCGGTTCCAGCCCTGACGCAACCAAGATCTTCATCAACGGCACGTGG
GTTGGTGTGCACAGTGAACCAGCACACTTGGTCACACTTGTGCAGGAGCTTCGGCGCAA
ATGCATCATTTCTCACGAAGTCTCCCTCGTTCGCGACATTCGCGACCGTGAGTTCAAGAT
CTTCTCCGACGCTGGTCGTGTCATGCGGCCTCTCTTCGTTGTCAATCAAGAGGATAATCC
CGAGACTGGAGCGCAGCAGGGCACTCTGGCTCTCACCAAACACCACATTCGCCGTCTCG
AGGAAGATGCTCAGTACCATCGCAAGAAGCATGATGACGACTATTTCGGATGGGATGGG
CTTCAGAACAGCGGTTGCATCGAGTACCTGGATGCCGAGGAAGAGGAGACAGCGATGA
TCTGCATGAGTCCTGAGGATCTAGAGGACTACCGCCAGCGCAAGGCTAGGGGCAAGGAT
GCCAAGGATGATGAGCCGGAGGAGGATGGCAGGAGTCTGAACGCTCGTGTGAAGACGA
AGATCAACCCCGACATCCACATGTATACCCACTGCGAGATCCATCCGAGCATGCTCCTG
GGAATATGCGCCAGTATCATTCCCTTTCCGGATCACAATCAGGTGTGTAAAATTCTTGCA
GCACTAATATCACCCCTGCTGACCACATATATCTAGTCACCTCGCATGAATCAAATCAGC
ATCATCACAAGTGCGCAAACATCAAAATCAAGGCAAATGGTCGCTATCTTTTCGTCGCG
GCCGTTTGCCTTGGGCCCCTGAGCGCATGGCCTTTTTTCGCCCACTGCACATTTTCGCTTT
GTGGTGCGGGGTTTTGCGTCTGGGTCTTATCTCAGATAAACGTGACCCACTCAACCGCCA
CCTCCATCATGCTCCGCCGCCAGCACACCATTGCGCGCGCCTCTCTTTGACCCTTACATT
TTGAACATGTACAATGCTGACGCGGATTTTCTACTGCAGCTGCTGAACTCGGCAAGGGTT

CCTTCAAGTACGCATGGGTCCTTGACAAGCTCAAGGCCGAGCGTGAGCGTGGTATCACC
ATCGACATTGCTCTGTGGAAGTTCGAGACTCCCAAGTACTATGTCACCGTCATTGGTAAG
CTCTACCACCCCCCCACCCCGTCCTGATGATTTCTCGCAGGCGGGGCTGCCACGGCCCGC
TGCGCTCCCCTTCAGACGCGATAGCGCGGCTGATGATGATTTTGCGCACCCCATGCTAAC
GCAAATGCCACCACAGACGCTCCCGGTCACCGTGACTTCATCAAGAACATGATCACTGG
TACCTCCCAGGCTGACTGCGCCATTCTCATCATTGCCTCCGGTACTGGTGAGTTCGAGGC
TGGTATCTCCAAGGATGGCCAGACCCGTGAGCACGCTCTGCTCGCCTACACCCTCGGTGT
CAAGCAGCTCATTGTTGCTTGCAACAAGATGGACACCGCTGGCTGGAAGCAGGACCGTT
TTGAGGAGATCAAGAAGGAGACGACCAACTTCATCAAGAAGGTCGGCTTCAACCCCAA
GCAGGTTGCTTTCGTCCCCATCTCCGGCTTCCACGGCGACAACATGCTTGAGGCCTCCGC
CAACATGCCCTGGTACAAGGGCTGGACCAAGGAGACCCAGGACAAGAAGGAGGCCAAG
GGCATGACCCTGCTCGACGCCATTGACGCCATTGAGCCCCCCAAGCGCCCCACCGAGAA
GCCCCTGCGTCTGCCCCTCCAGGATGTCTACAAGATCGGCGGTATTGGAACGGTACCTGT
CGGCCGTATCGAGACTGGTGTCCTGAAGCCCGGTATGGTCGTTACCTTCGCCCCCTCCAA
CGTCACCACTGAAGTCAAGTCCGTGGAAATGCACCACGAGCAGCTCACCGAGGGTCAGC
CCGGTGACAACGTTGGCTTCAACGTCAAGAACGTCTCCGTCAAGGAGATCCGCCGTGGC
AACGTCGCTGGTGACTCCAAGAACGACCCCCCACTGGCCGCCGCTTCCTTCAACGCCCA
GGTCATCGTTCTGAACCACCCCGGCCAGGTTGGTGCTGGATAC

>Melanconiella_decorahensis

CACACCGCCCGTCGCTACTACCGATTGAATGGTTCAGTGAGGCGTCCGGACTGGCCCAG
GGAGGTCGGCAACGACCACCCAGGGCCGGAAAGTTCTCCAAACTCGATCATTTAGAGGA
AGTAAAAGTCGTAACAAGGTCTCCGTTGGTGAACCAGCGGAGGGATCATTGATGGAACA
TACGTCCCATACCCCTTTGTGAACTTATACCTATATCTCGTTGCCTCGGCGCCAGGCCTG
GGGTTCCCCCTCGGGGGTTCCCCCCAGCCAGTGGACTCCAGCGGTCCCGGTTGGAGCAG
GCCCGCCGGTGGCCTCCCGAAACTCTTGTTTTTAGAACCTATCCTCTGAGATACTTAAAA
AAAAATGAATCAAAACTTTCAACAACGGATCTCTTGGCTCTGGCATCGATGAAGAACGC
AGCGAAATGCGATAAGTAATGTGAATTGCAGAATTCAGTGAATCATCGAATCTTTGAAC
GCACATTGCGCCCGCTGGAATTCCGGCGGGCATGCCTGTTCGAGCGTCATTTCAACCCCT
CAAGCCCCCGTGCTTGGTGTTGGGGCCTAGCCTGTAGAAAGGCTAGCCCTGAAATTCAG
TGGCGTGCTCGCTGAGACTCCCGGGTGTAGTAATCTCCTTCTCACCTAGGCTGGCTCGGC
GGTGCACTTGCCGTAAAACCCGCTATCACGCACCTTCTGAAAGTTGACCTCGGATCAGG
TAGGAATACCCGCTGAACTTAAGCATATCAATAAGCGGAGGAAAAGAAACCAACAGGG
ATTGCCCTAGTAACGGCGAGTGAAGCGGCAACAGCTCAAATTTGAAATCTGGTCCCCCC
TGGGGGCCCGAGTTGTAATTTGCAGAGGATGTTTTCTGGCGCGGTGCCTTCCGAGTTCCC
TGGAACGGGACGCCACAGAGGGTGAGAGCCCCGTAATGGTCGGATACCAAGCCTGTGT
GAAGCTCCTTCGACGAGTCGAGTAGTTTGGGAATGCTGCTCTAAATGGGAGGTAAATCT
CTTCTAAAGCTAAATACTGGCCAGAGACCGATAGCGCACAAGTAGAGTGATCGAAAGAT
GAAAAGCACCTTGAAAAGGGGGTTAAACAGTACGTGAAATTGTTGAAAGGGAAGCGCT
CATGACCAGACTTGCGCCGGGCGGCTCAGCAGGCGGTTCTGCCCCTGTGTACTCCGTCCC
GGTTCAGGCCAGCATCGGTTCTTGCCGGGGGATAAGAACGATAGGAACGTGGCCCTCCC
TCGGGGGGGTGTTATAGCCCGTCGTAACGATACCCTGGCGGGGACCGAGGTTCGCGCAT
TCGCAAGGATGCTGGCGTAATGGTTATCAGTGACCCGTCTTGAAACACGGACCAAGGAG
TCGTCCTTTAGAGCGAGCGTCTGGGTGTTAAAACCCGCACGCGTAATGAAAGTGAAATT
AGGTGAGAGCCCTCACGGGCGCATCATCGACCGATCCTGATGTTCTCGGACGGATTTGA
GTAAGAGTTTTAACGGACGGACCCGAAAGACAGTGAACTATGCTTGTATAGGGTGAAGC

CAGAGGAAACTCTGGTGGAGGCTCGCAGCGGTTCTGACGTGCAAATCGATCGTCAAATA
TGAGCATGGGGGCGAAAGACTAATCGAACCACACCGCCCGTCGCTACTACCGATTGAAT
GGTTCAGTGAGGCGTCCGGACTGGCCCAGGGAGGTCGGCAACGACCACCCAGGGCCGG
AAAGTTCTCCAAACTCGATCATTTAGAGGAAGTAAAAGTCGTAACAAGGTCTCCGTTGG
TGAACCAGCGGAGGGATCATTGATGGAACATACGTCCCATACCCCTTTGTGAACTTATA
CCTATATCTCGTTGCCTCGGCGCCAGGCCTGGGGTTCCCCCTCGGGGGTTCCCCCCAGCC
AGTGGACTCCAGCGGTCCCGGTTGGAGCAGGCCCGCCGGTGGCCTCCCGAAACTCTTGT
TTTTAGAACCTATCCTCTGAGATACTTAAAAAAAAATGAATCAAAACTTTCAACAACGG
ATCTCTTGGCTCTGGCATCGATGAAGAACGCAGCGAAATGCGATAAGTAATGTGAATTG
CAGAATTCAGTGAATCATCGAATCTTTGAACGCACATTGCGCCCGCTGGAATTCCGGCG
GGCATGCCTGTTCGAGCGTCATTTCAACCCCTCAAGCCCCCGTGCTTGGTGTTGGGGCCT
AGCCTGTAGAAAGGCTAGCCCTGAAATTCAGTGGCGTGCTCGCTGAGACTCCCGGGTGT
AGTAATCTCCTTCTCACCTAGGCTGGCTCGGCGGTGCACTTGCCGTAAAACCCGCTATCA
CGCACCTTCTGAAAGTTGACCTCGGATCAGGTAGGAATACCCGCTGAACTTAAGCATAT
CAATAAGCGGAGGAAAAGAAACCAACAGGGATTGCCCTAGTAACGGCGAGTGAAGCGG
CAACAGCTCAAATTTGAAATCTGGTCCCCCCTGGGGGCCCGAGTTGTAATTTGCAGAGG
ATGTTTTCTGGCGCGGTGCCTTCCGAGTTCCCTGGAACGGGACGCCACAGAGGGTGAGA
GCCCCGTAATGGTCGGATACCAAGCCTGTGTGAAGCTCCTTCGACGAGTCGAGTAGTTT
GGGAATGCTGCTCTAAATGGGAGGTAAATCTCTTCTAAAGCTAAATACTGGCCAGAGAC
CGATAGCGCACAAGTAGAGTGATCGAAAGATGAAAAGCACCTTGAAAAGGGGGTTAAA
CAGTACGTGAAATTGTTGAAAGGGAAGCGCTCATGACCAGACTTGCGCCGGGCGGCTCA
GCAGGCGGTTCTGCCCCTGTGTACTCCGTCCCGGTTCAGGCCAGCATCGGTTCTTGCCGG
GGGATAAGAACGATAGGAACGTGGCCCTCCCTCGGGGGGGTGTTATAGCCCGTCGTAAC
GATACCCTGGCGGGGACCGAGGTTCGCGCATTCGCAAGGATGCTGGCGTAATGGTTATC
AGTGACCCGTCTTGAAACACGGACCAAGGAGTCGTCCTTTAGAGCGAGCGTCTGGGTGT
TAAAACCCGCACGCGTAATGAAAGTGAAATTAGGTGAGAGCCCTCACGGGCGCATCATC
GACCGATCCTGATGTTCTCGGACGGATTTGAGTAAGAGTTTTAACGGACGGACCCGAAA
GACAGTGAACTATGCTTGTATAGGGTGAAGCCAGAGGAAACTCTGGTGGAGGCTCGCAG
CGGTTCTGACGTGCAAATCGATCGTCAAATATGAGCATGGGGGCGAAAGACTAATCGAA
CGGTCCGCTCCTGGCAAAGCTGTTCCGAAACATCGTCAAGAAGCTGACCGGCGACATGA
TGGCGTATATGAAGCGTTGCGTTAGCGAGGGCAAGCATTTTGATCTGGCGTTAGGCATC
CGCCACTCAACGCTCACAAATGGCCTCAAGTACTCTCTCGCGACTGGAAACTGGGGTGA
GCAGAAGAAGGCGATGAGTTCCACCGCTGGTGTCTCGCAGGTGCTGAATCGGTACACCT
TCTCGTCTACCCTATCGCATTTGCGGCGCACGAACACGCCCATCGGTCGTGACGGAAAG
CTGGCCAAGCCTCGTCAGCTGCACAACACACACTGGGGTCTCGTCTGTCCAGCAGAGAC
TCCCGAGGGACAGGCTTGTGGTCTCGTCAAGAACCTGTCTCTAATGTGCTACGTTAGTGT
TGGCTCACCGGCTGAGCCTATCAAGGACTTCATGGTGCAGCGTAATATGGAAGTCTTGG
AGGAATACGAACCCGGTTCCAGCCCTGACGCAACCAAGATCTTCATCAACGGCACGTGG
GTTGGTGTGCACAGTGAACCAGCACACTTGGTCACACTTGTGCAGGAGCTTCGGCGCAA
ATGCATCATTTCTCACGAAGTCTCCCTCGTTCGCGACATTCGCGACCGTGAGTTCAAGAT
CTTCTCCGACGCTGGTCGTGTCATGCGGCCTCTCTTCGTTGTCAATCAAGAGGATAATCC
CGAGACTGGAGCGCAGCAGGGCACTCTGGCTCTCACCAAACACCACATTCGCCGTCTCG
AGGAAGATGCTCAGTACCATCGCAAGAAGCATGATGACGACTATTTCGGATGGGATGGG
CTTCAGAACAGCGGTTGCATCGAGTACCTGGATGCCGAGGAAGAGGAGACAGCGATGA
TCTGCATGAGTCCTGAGGATCTAGAGGACTACCGCCAGCGCAAGGCTAGGGGCAAGGAT
GCCAAGGATGATGAGCCGGAGGAGGATGGCAGGAGTCTGAACGCTCGTGTGAAGACGA
AGATCAACCCCGACATCCACATGTATACCCACTGCGAGATCCATCCGAGCATGCTCCTG
GGAATATGCGCCAGTATCATTCCCTTTCCGGATCACAATCAGGTGTGTAAAATTCTTGCA

GCACTAATATCACCCCTGCTGACCACATATATCTAGTCACCTCGCATGAATCAAATCAGC
ATCATCACAAGTGCGCAAACATCAAAATCAAGGCAAATGGTCGCTATCTTTTCGTCGCG
GCCGTTTGCCTTGGGCCCCTGAGCGCATGGCCTTTTTTCGCCCACTGCACATTTTCGCTTT
GTGGTGCGGGGTTTTGCGTCTGGGTCTTATCTCAGATAAACGTGACCCACTCAACCGCCA
CCTCCATCATGCTCCGCCGCCAGCACACCATTGCGCGCGCCTCTCTTTGACCCTTACATT
TTGAACATGTACAATGCTGACGCGGATTTTCTACTGCAGCTGCTGAACTCGGCAAGGGTT
CCTTCAAGTACGCATGGGTCCTTGACAAGCTCAAGGCCGAGCGTGAGCGTGGTATCACC
ATCGACATTGCTCTGTGGAAGTTCGAGACTCCCAAGTACTATGTCACCGTCATTGGTAAG
CTCTACCACCCCCCCACCCCGTCCTGATGATTTCTCGCAGGCGGGGCTGCCACGGCCCGC
TGCGCTCCCCTTCAGACGCGATAGCGCGGCTGATGATGATTTTGCGCACCCCATGCTAAC
GCAAATGCCACCACAGACGCTCCCGGTCACCGTGACTTCATCAAGAACATGATCACTGG
TACCTCCCAGGCTGACTGCGCCATTCTCATCATTGCCTCCGGTACTGGTGAGTTCGAGGC
TGGTATCTCCAAGGATGGCCAGACCCGTGAGCACGCTCTGCTCGCCTACACCCTCGGTGT
CAAGCAGCTCATTGTTGCTTGCAACAAGATGGACACCGCTGGCTGGAAGCAGGACCGTT
TTGAGGAGATCAAGAAGGAGACGACCAACTTCATCAAGAAGGTCGGCTTCAACCCCAA
GCAGGTTGCTTTCGTCCCCATCTCCGGCTTCCACGGCGACAACATGCTTGAGGCCTCCGC
CAACATGCCCTGGTACAAGGGCTGGACCAAGGAGACCCAGGACAAGAAGGAGGCCAAG
GGCATGACCCTGCTCGACGCCATTGACGCCATTGAGCCCCCCAAGCGCCCCACCGAGAA
GCCCCTGCGTCTGCCCCTCCAGGATGTCTACAAGATCGGCGGTATTGGAACGGTACCTGT
CGGCCGTATCGAGACTGGTGTCCTGAAGCCCGGTATGGTCGTTACCTTCGCCCCCTCCAA
CGTCACCACTGAAGTCAAGTCCGTGGAAATGCACCACGAGCAGCTCACCGAGGGTCAGC
CCGGTGACAACGTTGGCTTCAACGTCAAGAACGTCTCCGTCAAGGAGATCCGCCGTGGC
AACGTCGCTGGTGACTCCAAGAACGACCCCCCACTGGCCGCCGCTTCCTTCAACGCCCA
GGTCATCGTTCTGAACCACCCCGGCCAGGTTGGTGCTGGATAC

>Melanconiella_betulicola

CATTGCTGGAACACGTCCCCCAGGGCGTTCCCAGATACCCTTTGTGAACTTATACCTATT
TCGTTGCCTCGGCGCTGGCTGGGGGCCCTTCTATGGGGCTCCCCCACGGTCTCCGTGGAG
CAGGCCCGCCGGCGGCCCTACAAACTCTTGTTTTTATAACGTATCTCTTCTGAGTAACAA
ACTTACAAATGAATCAAAACTTTCAACAACGGATCTCTTGGTTCTGGCATCGATGAAGA
ACGCAGCGAAATGCGATAAGTAATGTGAATTGCAGAATTCAGTGAATCATCGAATCTTT
GAACGCACATTGCGCCCGCTGGTATTCCGGCGGGCATGCCTGTTCGAGCGTCATTTCAAC
CCTCAAGCTTTACCGCTTGGTGTTGGGGCGTAGCCTGTAAAAAGGCTAGCCCTGAAATTC
AGTGGCGTGCTCGCTAAGACTCCGAGCGTAGTAATTTTTTTCTCGCTTAGGTTGGATTAG
CGGTGCTCAGCCGTAAAACCTCGGCCCGAGTTGTAATTTGCAGAGGATGCTTCTGGCGC
GGTGCCTTCCGAGTTCCCTGGAACGGGACGCCACAGAGGGTGAGAGCCCCGTATGGTCG
GACACCAAGCCTGTGTGAAGCTCCTTCGACGAGTCGAGTAGTTTGGGAATGCTGCTCAA
AATGGGAGGTAAATCTCTTCTAAAGCTAAATACCGGCCAGAGACCGATAGCGCACAAGT
AGAGTGATCGAAAGATGAAAAGCACCTTGAAAAGGGGGTTAAACAGTACGTGAAATTG
TTGAAAGGGAAGCGCTTGTGACCAGACTTGTGCCGGGCGGCTCATCAGGGGTTCTCCCC
TGTGTACTCCGCCCGGTTCAGGCCAGCATCGGTTTTCGCTGGGGGATAAGAACGGTGGG
AACGTGGCCCCCCCTCGGGGGGGTGTTATAGCCCGCCGTACGATACCCTAGCGGGGACC
GAGGTTCGCGCTCCGCAAGGATGCTGGCGTAATGGTCATCAGCGACCCGTCTTACGGGT
GATATGATGGCGTACATGAAGCGTTGTGTCAGCGAGGGCAAGCATTTCGACCTAGCGCT
GGGCATTCGCCATTCCACCCTCACAAATGGTTTGAAGTACTCTCTTGCGACTGGAAACTG
GGGTGAGCAGAAGAAGGCAATGAGTTCTACCGCTGGCGTCTCGCAGGTGCTCAACCGAT

ACACGTTTTCGTCTACCCTATCGCATTTGCGCCGCACGAATACGCCCATTGGCCGTGACG
GCAAGCTAGCCAAGCCTCGTCAGCTTCACAACACTCACTGGGGCCTCGTCTGTCCAGCA
GAGACACCTGAGGGACAAGCTTGCGGTCTCGTCAAGAACCTGTCTTTAATGTGCTACGT
CAGTGTTGGCTCACCAGCCGAGCCTATCAAGGACTTCATGGTGCAGCGAAATATGGAAG
TTTTGGAGGAATACGAGCCGGGCTCCAGCCCCGACGCGACCAAGATCTTCATCAACGGC
ACGTGGGTTGGTGTCCACAACGAACCAGCACACTTAGTCACGCTTGTGCAAGAGCTTCG
ACGCAAATGCATCATTTCTCACGAGGTCTCCCTCGTTCGCGATATTCGCGATCGCGAGTT
CAAGATCTTCTCCGACGCTGGTCGTGTGATGCGGCCTCTTTTCGTCGTCAACCAAGAGGA
CAACCCCGAGACTGGAGCGCAGCAGGGCACTCTGGCTCTCACCAAGCATCACATTCGCC
GTCTCGAGGAAGATGCTCAGTATCAGCGCAAGAAGCATGACGAAGACTATTTGGCTGG
GATGGCCTTCAGAACAGCGGCTGCATTGAGTACCTGGATGCCGAGGAAGAGGAGACAG
CAATGATCTGCATGAGTCCTGAGGATCTCGAGGACTACCGGCAGCGCAAGGCCAGCGGC
AAGGATGCCAAGCAGGAAGAGCCTGAGGATGATGGCAGGAGTTTGAACGCTCGTGTGA
AGACGAAGATCAATACCGACATCCACATGTACACCCACTGCGAAATCCATCCGAGCATG
CTCTTGGGAATCTGCGCCAGCCGTCATCATCACAATCGCACAAACAAACTCCAAAGCAA
ACGGTCGCGATTTCCTTGCCCGGCCGCTTTCTTAGTTGGCTGTGTGTCCTTTTTTTGCCCG
TCGCATATTTTCGCTTTGGTGCGGGGTTTTGCATCTGGGTCTTATCTCAGATAAACGTGA
CCCACCGACCCGCCACCCCAATCACTCTGCCAACACACTCTTGCGCACCACTCACCGTCC
CACCATGACAACGGCGATGCTAACTCAATTCTTTCAACAGCTGCTGAACTCGGCAAGGG
TTCCTTCAAGTACGCATGGGTCCTTGACAAGCTCAAGGCCGAGCGTGAGCGTGGTATCA
CCATCGACATTGCTCTGTGGAAGTTCGAGACTCCCAAGTACTATGTCACCGTCATTGGTA
AGTTTGTCCGTTTTGACGATATCGCGCAGACCTGGCTACCCCGCCTACGCACTGCTCCAC
CAGGGGCACCATGGTGGCTCCTTACACTTGTGCGCAACCCGTGCTAACGCAAGTGTCAC
CACAGACGCTCCCGGTCACCGTGACTTCATCAAGAACATGATCACTGGTACCTCCCAGG
CTGACTGCGCCGTTCTCATCATTGCCTCTGGTACT

>Melanconiella_echinata

GGTGAACCAGCGGAGGGATCATTGACAAACACACGTCTCCTACCCTTTGTGAACTTATA
CCCATCGTTGCCTCGGCGCCAGGCCCGGGGTGCCCCCCAGGGGGTGCCCCCCACCCGGG
ACCTTACGGTCCCGGGTGGAGCAGGCCCGCCGGCGGCCCACCTAAACTCTTGTTTTTAG
AACCTATCTCCTCTGAGTTTGTTACCTAGAATGAATTAAAACTTTCAACAACGGATCTCT
TGGCTCTGGCATCGATGAAGAACGCAGCGAAATGCGATAAGTAATGTGAATTGCAGAAT
TCAGTGAATCATCGAATCTTTGAACGCACATTGCGCCCGCTGGAATTCCGGCGGGCATG
CCTGTTCGAGCGTCATTTCAACCCCTCAAGCTTCAGCGCTTGGTGTTGGGGCATAGCCTG
TAAAAAGGCTAGCCCTTAAATTCAGTGGCGTGCGCGCCGAGAACCCCGGGCGTAGTAAT
CTCCTTCTCTCCCGGGCAGGCTCGGTGGTGCACTTGCCGTAAAACCCGCTATCACGCACC
TTCTGAAAGTTGACCTCGGATCAGGTAGGAATACCCGCTGAACTTAAGCATATCAATAA
GCGGAGGAAAAGAAACCAACAGGGATTGCCTTAGTAACGGCGAGTGAAGCGGCAACAG
CTCAAATTTGAAATCTGGCCCCCCCCGGGGTCCGAGTTGTAATTTGCAGAGGATGTCTTC
TGGCGCGGTGCCTTCCGAGTTCCCTGGAACGGGACGCCACAGAGGGTGAGAGCCCCGTA
ATGGTCGGATACCAAGCCTGTGTGAAGCTCCTTCGACGAGTCGAGTAGTTTGGGAATGC
TGCTCTAAATGGGAGGTAAATCTCTTCTAAAGCTAAATACTGGCCAGAGACCGATAGCG
CACAAGTAGAGTGATCGAAAGATGAAAAGCACCTTGAAAAGGGGGTTAAACAGTACGT
GAAATTGTTGAAAGGGAAGCGCTCATGACCAGACTTGCGCCGGGCGGCTCAGCAGGTG
GTTCCGCCCCTGTGTACTCCGTCCCGGTTCAGGCCAGCATCGGTTCTCGCCGGGGGATAA
GCACGGTGGGAACGTGGCCCCCCCTCGGGGGGGTGTTATAGCCCGCCGTTACGATACCC
TGGCGGGGACCGAGGTTCGCGCATCTGCAAGGATGCTGGCGTAATGGTTATCAGTGACC

CGTCTTGAAACACGGACCAAGGAGTCGTCCTTTAGAGCGAGCGTCTGGGTGTCAAAACC
CGCACGCGTAATGAAAGTGAAATTAGGTGAGAGCCCTCGCGGGCGCATCATCGACCGAT
CCTGATGTTCTCGGACGGATTTGAGTAAGAGTTTTAACGGACGGACCCGAAAGACAGTG
AACTATGCTTGGATAGGGTGAAGCCAGAGGAAACTCTGGTGGAGGCTCGCAGCGGTTCT
GACGTGCAAATCGATCGTCAAATCTGAGCATGGGGGCGAAAGACTAATCGAACGGTGA
ACCAGCGGAGGGATCATTGACAAACACACGTCTCCTACCCTTTGTGAACTTATACCCATC
GTTGCCTCGGCGCCAGGCCCGGGGTGCCCCCCAGGGGGTGCCCCCCACCCGGGACCTTA
CGGTCCCGGGTGGAGCAGGCCCGCCGGCGGCCCACCTAAACTCTTGTTTTTAGAACCTA
TCTCCTCTGAGTTTGTTACCTAGAATGAATTAAAACTTTCAACAACGGATCTCTTGGCTC
TGGCATCGATGAAGAACGCAGCGAAATGCGATAAGTAATGTGAATTGCAGAATTCAGTG
AATCATCGAATCTTTGAACGCACATTGCGCCCGCTGGAATTCCGGCGGGCATGCCTGTTC
GAGCGTCATTTCAACCCCTCAAGCTTCAGCGCTTGGTGTTGGGGCATAGCCTGTAAAAA
GGCTAGCCCTTAAATTCAGTGGCGTGCGCGCCGAGAACCCCGGGCGTAGTAATCTCCTT
CTCTCCCGGGCAGGCTCGGTGGTGCACTTGCCGTAAAACCCGCTATCACGCACCTTCTGA
AAGTTGACCTCGGATCAGGTAGGAATACCCGCTGAACTTAAGCATATCAATAAGCGGAG
GAAAAGAAACCAACAGGGATTGCCTTAGTAACGGCGAGTGAAGCGGCAACAGCTCAAA
TTTGAAATCTGGCCCCCCCCGGGGTCCGAGTTGTAATTTGCAGAGGATGTCTTCTGGCGC
GGTGCCTTCCGAGTTCCCTGGAACGGGACGCCACAGAGGGTGAGAGCCCCGTAATGGTC
GGATACCAAGCCTGTGTGAAGCTCCTTCGACGAGTCGAGTAGTTTGGGAATGCTGCTCT
AAATGGGAGGTAAATCTCTTCTAAAGCTAAATACTGGCCAGAGACCGATAGCGCACAAG
TAGAGTGATCGAAAGATGAAAAGCACCTTGAAAAGGGGGTTAAACAGTACGTGAAATT
GTTGAAAGGGAAGCGCTCATGACCAGACTTGCGCCGGGCGGCTCAGCAGGTGGTTCCGC
CCCTGTGTACTCCGTCCCGGTTCAGGCCAGCATCGGTTCTCGCCGGGGGATAAGCACGG
TGGGAACGTGGCCCCCCCTCGGGGGGGTGTTATAGCCCGCCGTTACGATACCCTGGCGG
GGACCGAGGTTCGCGCATCTGCAAGGATGCTGGCGTAATGGTTATCAGTGACCCGTCTT
GAAACACGGACCAAGGAGTCGTCCTTTAGAGCGAGCGTCTGGGTGTCAAAACCCGCACG
CGTAATGAAAGTGAAATTAGGTGAGAGCCCTCGCGGGCGCATCATCGACCGATCCTGAT
GTTCTCGGACGGATTTGAGTAAGAGTTTTAACGGACGGACCCGAAAGACAGTGAACTAT
GCTTGGATAGGGTGAAGCCAGAGGAAACTCTGGTGGAGGCTCGCAGCGGTTCTGACGTG
CAAATCGATCGTCAAATCTGAGCATGGGGGCGAAAGACTAATCGAAC?????????????????
??
??
??
??
??
??
??
??
??
??
??
??
??
??
??
??
??

??
??
??
??
??
??
??
??
??
??
??
???

>Melanconiella_elegans

CACACCGCCCGTCGCTACTACCGATTGAATGGTTCAGTGAGGCGTCCGGACTGGCCCAG
GGAGGTCGGCAACGACCACCCAGGGCCGGAAAGTTCTCCAAACTCGATCATTTAGAGGA
AGTAAAAGTCGTAACAAGGTCTCCGTTGGTGAACCAGCGGAGGGATCATTGAAAAATAT
GGAATATACGTCCCATAAACCCTTTGTGAATATATACCTACCCTCGTTGCCTCGGCGTCA
GGCCCGGGGTTCCCCCCCCAGGGGGGTTCCCCCCACCTACTACGGTAGGTGGAGCAGGC
CCGCCGGTGGCCCCCCTAAACTCTTGTTTTTAGAACCTATTCTCCTCTGAGTTTTTATAC
CAAAATAAGTCAAAACTTTCAACAACGGATCTCTTGGCTCTGGCATCGATGAAGAACGC
AGCGAAATGCGATAAGTAATGTGAATTGCAGAATTCAGTGAATCATCGAATCTTTGAAC
GCACATTGCGCCCGCTGGAATTCCGGCGGGCATGCCTGTTCGAGCGTCATTTCAACCCCT
CAAGCTTTATCGCTTGGTGTTGGGGCATAGCCTGTAAAAAGGCTAGCCCTTAAATTCAGT
GGCGTGCTCGCTGAGACTCCCGGGTGTAGTAATACTCCTTCTCGCCCGGGCTTGGTCTCA
GCGGTGCACTCGCCGTAAAACCCGCTATCACGCACCTTCTGAAAGTTGACCTCGGATCA
GGTAGGAATACCCGCTGAACTTAAGCATATCAATAAGCGGAGGAAAAGAAACCAACAG
GGATTGCCCTAGTAACGGCGAGTGAAGCGGCAACAGCTCAAATTTGAAATCTGGCCTCC
CCACGGGGGTCCGAGTTGTAATTTGCAGAGGATGTCTTCTGGCGCGGTGCCTTCCGAGTT
CCCTGGAACGGGACGCCACAGAGGGTGAGAGCCCCGTAGCGGTCGGATACCAAGCCTG
TGTGAAGCTCCTTCGACGAGTCGAGTAGTTTGGGAATGCTGCTCTAAATGGGAGGTAAA
TCTCTTCTAAAGCTAAATACTGGCCAGAGACCGATAGCGCACAAGTAGAGTGATCGAAA
GATGAAAAGCACCTTGAAAAGGGGGTTAAACAGTACGTGAAATTGTTGAAAGGGAAGC
GCTCATGACCAGACTTGCGCCGGGCGGCTCAGCAGGTGGTTCTGCCCCTGTGTACTCCGT
CCCGGTTCAGGCCAGCATCGGTTCTCGTTGGGGGATAAGAACGGTGGAACGTGGCCCT
CCCTCGGGGGGGTGTTATAGCCCGCCGTAACGATACCCTGGCGGGGACCGAGGTTCGCG
CATCTTGCAAGGATGCTGGCGTAATGGTTATCAGCGACCCGTCTTGAAACACGGACCAA
GGAGTCGTCCTTTAGAGCGAGCGTCTGGGTGTCAAAACCCGCACGCGTAATGAAAGTGA
AATTAGGTGAGAGCCCTCACGGGCGCATCATCGACCGATCCTGATGTTCTCGGACGGAT
TTGAGTAAGAGTTTTAACGGACGGACCCGAAAGACAGTGAACTATGCTTGTATAGGGTG
AAGCCAGAGGAAACTCTGGTGGAGGCTCGCAGCGGTTCTGACGTGCAAATCGATCGTCA
AATATGAGCATGGGGGCGAAAGACTAATCGAACCACACCGCCCGTCGCTACTACCGATT
GAATGGTTCAGTGAGGCGTCCGGACTGGCCCAGGGAGGTCGGCAACGACCACCCAGGG
CCGGAAAGTTCTCCAAACTCGATCATTTAGAGGAAGTAAAAGTCGTAACAAGGTCTCCG
TTGGTGAACCAGCGGAGGGATCATTGAAAAATATGGAATATACGTCCCATAAACCCTTT
GTGAATATATACCTACCCTCGTTGCCTCGGCGTCAGGCCCGGGGTTCCCCCCCCAGGGG
GGTTCCCCCCACCTACTACGGTAGGTGGAGCAGGCCCGCCGGTGGCCCCCCTAAACTCT

TGTTTTTAGAACCTATTCTCCTCTGAGTTTTTATACCAAAATAAGTCAAAACTTTCAACA
ACGGATCTCTTGGCTCTGGCATCGATGAAGAACGCAGCGAAATGCGATAAGTAATGTGA
ATTGCAGAATTCAGTGAATCATCGAATCTTTGAACGCACATTGCGCCCGCTGGAATTCCG
GCGGGCATGCCTGTTCGAGCGTCATTTCAACCCCTCAAGCTTTATCGCTTGGTGTTGGGG
CATAGCCTGTAAAAAGGCTAGCCCTTAAATTCAGTGGCGTGCTCGCTGAGACTCCCGGG
TGTAGTAATACTCCTTCTCGCCCGGGCTTGGTCTCAGCGGTGCACTCGCCGTAAAACCCG
CTATCACGCACCTTCTGAAAGTTGACCTCGGATCAGGTAGGAATACCCGCTGAACTTAA
GCATATCAATAAGCGGAGGAAAAGAAACCAACAGGGATTGCCCTAGTAACGGCGAGTG
AAGCGGCAACAGCTCAAATTTGAAATCTGGCCTCCCCACGGGGGTCCGAGTTGTAATTT
GCAGAGGATGTCTTCTGGCGCGGTGCCTTCCGAGTTCCCTGGAACGGGACGCCACAGAG
GGTGAGAGCCCCGTAGCGGTCGGATACCAAGCCTGTGTGAAGCTCCTTCGACGAGTCGA
GTAGTTTGGGAATGCTGCTCTAAATGGGAGGTAAATCTCTTCTAAAGCTAAATACTGGC
CAGAGACCGATAGCGCACAAGTAGAGTGATCGAAAGATGAAAAGCACCTTGAAAAGGG
GGTTAAACAGTACGTGAAATTGTTGAAAGGGAAGCGCTCATGACCAGACTTGCGCCGGG
CGGCTCAGCAGGTGGTTCTGCCCCTGTGTACTCCGTCCCGGTTCAGGCCAGCATCGGTTC
TCGTTGGGGGATAAGAACGGTGGGAACGTGGCCCTCCCTCGGGGGGGTGTTATAGCCCG
CCGTAACGATACCCTGGCGGGGACCGAGGTTCGCGCATCTTGCAAGGATGCTGGCGTAA
TGGTTATCAGCGACCCGTCTTGAAACACGGACCAAGGAGTCGTCCTTTAGAGCGAGCGT
CTGGGTGTCAAAACCCGCACGCGTAATGAAAGTGAAATTAGGTGAGAGCCCTCACGGGC
GCATCATCGACCGATCCTGATGTTCTCGGACGGATTTGAGTAAGAGTTTTAACGGACGG
ACCCGAAAGACAGTGAACTATGCTTGTATAGGGTGAAGCCAGAGGAAACTCTGGTGGA
GGCTCGCAGCGGTTCTGACGTGCAAATCGATCGTCAAATATGAGCATGGGGGCGAAAGA
CTAATCGAACGGTCCGCTCCTGGCAAAGCTGTTCCGAAACATCGTCAAGAAGCTGACCG
GCGACATGATGGCGTACATGAAGCGTTGCGTCAGCGAGGGCAAGCATTTCGATCTGGCG
TTGGGCATCCGCCACTCAACCCTCACAAATGGCCTCAAGTACTCTCTTGCGACGGGAAA
TTGGGGCGAGCAGAAGAAGGCGATGAGTTCCACCGCTGGTGTCTCGCAGGTGCTGAACC
GGTATACGTTCTCCTCCACCCTATCGCATTTGCGGCGCACGAATACGCCTATCGGCCGTG
ACGGCAAGCTGGCCAAGCCTCGTCAGCTGCACAATACACACTGGGGTCTCGTCTGTCCA
GCAGAGACCCCCGAGGGACAGGCTTGCGGTCTCGTCAAGAACCTGTCGTTAATGTGCTA
CGTGAGTGTCGGTTCACCGGCTGAGCCTATCAAGGACTTCATGGTGCAGCGTAATATGG
AAGTTTTGGAGGAATACGAGCCCGGCTCCAGCCCTGACGCAACCAAGATCTTCATCAAC
GGCACGTGGGTTGGTGTGCACAATGAGCCAGCACACTTGGTCACGCTTGTGCAGGAGCT
TCGGCGCAAATGCATCATTTCCCACGAAGTCTCCCTCGTTCGCGACATTCGCGACCGCGA
GTTCAAGATCTTCTCCGACGCTGGTCGTGTCATGCGGCCTCTCTTCGTTGTCAATCAGGA
TGACAATCCCGAGACGGGGGCCCAGCAAGGCACCCTGGCTCTCACCAAGCACCATATTC
GCCGCCTTGAGGAAGATGCTCAGTACCATCGCAAGAAGCATGATGACGACTATTTCGGC
TGGGATGGTCTTCAGAACAGCGGTTGCATCGAGTACTTGGATGCCGAGGAGGAGGAGAC
AGCAATGATCTGCATGAGTCCTGAGGATCTTGAGGACTACCGCCAGCGCAAGGCTAGGG
GCAAGGATGCCAAGGATGAGGAGCCGGAGGACGATGGCAGGAGTCTGAACGCTCGTGT
GAAGACGAAGATCAACCCCGACATCCACATGTACACGCACTGCGAGATCCATCCGAGCA
TGCTCCTTGGAATCTGCGCCAGCATCATCCCCTTCCCGGACCACAATCAGGTGTGTAAAC
TTCTCCCAGTAACTACTACCACTCCTGCTGACCACATCTACCTAGTCACCTCGCAGCAAT
CAAATCAGCACCATCACCATCGCGCAATCGTCAAGATCAAGCCCAAATGTCCGCCATCT
TTTCGTCGCGGCCGTTTACCCCGGCCCGCGGGGGGGGGGGGGGTTGTCCCTTTTTCGCCC
ACCGCACATTTTCGCTTTGGTGCGGGGTTTTGCGTCTGGGTCTTATCTCAGATAAACGTG
ACCCACCCAACCGCCACCTCAGTCGTCCCCCGCCAACACACCCTTGCGCGCGCCTCTTTT
TCTAACACTCGAAACTTGACAACGACAATGCTGACTCTAGTTTTCTACTACAGCTGCTGA

GCTCGGAAAGGGTTCCTTCAAGTACGCATGGGTCCTTGACAAGCTCAAGGCCGAGCGTG
AGCGTGGTATCACCATCGACATTGCTCTGTGGAAGTTCGAGACTCCCAAGTACTATGTCA
CCGTCATTGGTAAGCTCTATCCCTGCTGCTCTGGTCGCCCCGCCTCACTACCCGGCCTTG
CCAGGCGCGAGACCGCGACTTCTGATGCCTTTGCGCAACCCGTGCTAACGCAAGTGTTA
CCACAGACGCTCCCGGTCACCGTGACTTCATCAAGAACATGATCACTGGTACCTCCCAG
GCTGACTGCGCCATTCTCATCATTGCCTCCGGTACTGGTGAGTTCGAGGCTGGTATCTCC
AAGGATGGCCAGACTCGTGAGCACGCTCTGCTCGCCTACACCCTCGGTGTCAAGCAGCT
CATTGTTGCTTGCAACAAGATGGACACCGCTGGCTGGAAGCAGGAGCGTTTCGAGGAGA
TCAAGAAGGAGACCACCAACTTCATCAAGAAGGTTGGCTTCAACCCCAAGCAGGTCGCT
TTCGTCCCCATCTCCGGCTTCCACGGCGACAACATGCTTGAGGCCTCCTCCAACATGCCC
TGGTACAAGGGCTGGTCCAAGGAGACCCAGGACAAGAAGGAGGCCAAGGGCATGACCC
TGCTCGATGCCATTGACGCCATTGAGCCCCCCAAGCGTCCCACCGAGAAGCCCCTGCGT
CTGCCCCTCCAGGATGTCTACAAGATCGGCGGTATCGGAACTGTACCTGTCGGCCGTATC
GAGACTGGTATCCTGAAGCCCGGTATGGTCGTCACCTTCGCCCCCTCCAACGTCACCACT
GAGGTCAAGTCCGTGGAGATGCACCACGAGCAGCTCACCGAGGGTCAGCCTGGTGACA
ACGTTGGTTTCAACGTCAAGAACGTCTCCGTCAAGGAGATTCGCCGTGGCAACGTCGCT
GGTGACTCCAAGAACGACCCCCCCATGGCCGCCGCCTCCTTCAACGCCCAGGTCATCGT
CCTGAACCACCCCGGCCAGGTCGGTGCTGGATAC

>Melanconiella_elegans

GTAACAAGGTCTCCGTTGGTGAACCAGCGGAGGGATCATTGAAAAATATGGAATATACC
TCCCATAAACCCTTTGTGAATATATACCTACCCTCGTTGCCTCGGCGTCAGGCCCGGGGT
TCCCCCCCAGGGGGGTTCCCCCCACCTACTACGGTAGGTGGAGCAGGCCCGCCGGTGGC
CCCCCTAAACTCTTGTTTTTAGAACCTATTCTCCTCTGAGTTTTTTATACCAAAATAAGT
CAAAACTTTCAACAACGGATCTCTTGGCTCTGGCATCGATGAAGAACGCAGCGAAATGC
GATAAGTAATGTGAATTGCAGAATTCAGTGAATCATCGAATCTTTGAACGCACATTGCG
CCCGCTGGAATTCCGGCGGGCATGCCTGTTCGAGCGTCATTTCAACCCCTCAAGCTTTAT
CGCTTGGTGTTGGGGCATAGCCTGTAAAAAGGCTAGCCCTTAAATTCAGTGGCGTGCTC
GCTGAGACTCCCGGGTGTAGTAATACTCCTTCTCGCCCGGGCTTGGTCTCAGCGGTGCAC
TCGCCGTAAAACCCGCTATCACGCACCTTCTGAAAGTTGACCTCGGATCAGGTAGGAAT
ACCCGCTGAACTTAAGCATATCAATAACAGGAGGAAAAGAAACCAACAGGGATTGCCC
TAGTAACGGCGAGTGAAGCGGCAACAGCTCAAATTTGAAATCTGGCCTCCCCACGGGGG
TCCGAGTTGTAATTTGCAGAGGATGTCTTCTGGCGCGGTGCCTTCCGAGTTCCCTGGAAC
GGGACGCCACAGAGGGTGAGAGCCCCGTAGCGGTCGGATACCAAGCCTGTGTGAAGCT
CCTTCGACGAGTCGAGTAGTTTGGGAATGCTGCTCTAAATGGGAGGTAAATCTCTTCTAA
AGCTAAATACTGGCCAGAGACCGATAGCGCACAAGTAGAGTGATCGAAAGATGAAAAG
CACCTTGAAAAGGGGGTTAAACAGTACGTGAAATTGTTGAAAGGGAAGCGCTCATGACC
AGACTTGCGCCGGGCGGCTCAGCAGGTGGTTCTGCCCCTGTGTACTCCGTCCCGGTTCAG
GCCAGCATCGGTTCTCGTTGGGGGATAAGAACGGTGGGAACGTGGCCCTCCCTCGGGGG
GGTGTTATAGCCCGCCGTAACGATACCCTGGCGGGGACCGAGGTTCGCGCATCTTGCAA
GGATGCTGGCGTAATGGTTATCAGTGACCCGTCTTGAAACACGGACCAAGGAGTCGTCC
TTTAGAGCGAGCGTCTGGGTGTCAAAACCCGCACGCGTAATGAAAGTGAAATTAGGTGA
GAGCCCTCACGGGCGCATCATCGACCGATCCTGATGTTCTCGGACGGATTTGAGTAAGA
GTTTTAACGGACGGACCCGAAAGACAGTGAACTATGCTTGTATAGGGTGAAGCCAGAGG
AAACTCTGGTGGAGGCTCGCAGCGGTTCTGACGTGCAAATCGATCGTCAAATATGAGCA
TGGGGGCGAAAGACTAATCGAACGTAACAAGGTCTCCGTTGGTGAACCAGCGGAGGGA
TCATTGAAAAATATGGAATATACCTCCCATAAACCCTTTGTGAATATATACCTACCCTCG

TTGCCTCGGCGTCAGGCCCGGGGTTCCCCCCAGGGGGGTTCCCCCACCTACTACGGTA
GGTGGAGCAGGCCCGCCGGTGGCCCCCTAAACTCTTGTTTTAGAACCTATTCTCCTCT
GAGTTTTTTATACCAAAATAAGTCAAAACTTTCAACAACGGATCTCTTGGCTCTGGCAT
CGATGAAGAACGCAGCGAAATGCGATAAGTAATGTGAATTGCAGAATTCAGTGAATCAT
CGAATCTTTGAACGCACATTGCGCCCGCTGGAATTCCGGCGGGCATGCCTGTTCGAGCG
TCATTTCAACCCCTCAAGCTTTATCGCTTGGTGTTGGGGCATAGCCTGTAAAAAGGCTAG
CCCTTAAATTCAGTGGCGTGCTCGCTGAGACTCCCGGGTGTAGTAATACTCCTTCTCGCC
CGGGCTTGGTCTCAGCGGTGCACTCGCCGTAAAACCCGCTATCACGCACCTTCTGAAAG
TTGACCTCGGATCAGGTAGGAATACCCGCTGAACTTAAGCATATCAATAACAGGAGGAA
AAGAAACCAACAGGGATTGCCCTAGTAACGGCGAGTGAAGCGGCAACAGCTCAAATTT
GAAATCTGGCCTCCCCACGGGGGTCCGAGTTGTAATTTGCAGAGGATGTCTTCTGGCGC
GGTGCCTTCCGAGTTCCCTGGAACGGGACGCCACAGAGGGTGAGAGCCCCGTAGCGGTC
GGATACCAAGCCTGTGTGAAGCTCCTTCGACGAGTCGAGTAGTTTGGGAATGCTGCTCT
AAATGGGAGGTAAATCTCTTCTAAAGCTAAATACTGGCCAGAGACCGATAGCGCACAAG
TAGAGTGATCGAAAGATGAAAAGCACCTTGAAAAGGGGGTTAAACAGTACGTGAAATT
GTTGAAAGGGAAGCGCTCATGACCAGACTTGCGCCGGGCGGCTCAGCAGGTGGTTCTGC
CCCTGTGTACTCCGTCCCGGTTCAGGCCAGCATCGGTTCTCGTTGGGGGATAAGAACGGT
GGGAACGTGGCCCTCCCTCGGGGGGGTGTTATAGCCCGCCGTAACGATACCCTGGCGGG
GACCGAGGTTCGCGCATCTTGCAAGGATGCTGGCGTAATGGTTATCAGTGACCCGTCTT
GAAACACGGACCAAGGAGTCGTCCTTTAGAGCGAGCGTCTGGGTGTCAAAACCCGCACG
CGTAATGAAAGTGAAATTAGGTGAGAGCCCTCACGGGCGCATCATCGACCGATCCTGAT
GTTCTCGGACGGATTTGAGTAAGAGTTTTAACGGACGGACCCGAAAGACAGTGAACTAT
GCTTGTATAGGGTGAAGCCAGAGGAAACTCTGGTGGAGGCTCGCAGCGGTTCTGACGTG
CAAATCGATCGTCAAATATGAGCATGGGGGCGAAAGACTAATCGAACGGTCCGCTCCTG
GCAAAGCTGTTCCGAAACATCGTCAAGAAGCTGACCGGCGACATGATGGCGTACATGAA
GCGTTGCGTCAGCGAGGGCAAGCATTTCGATCTGGCGTTGGGCATCCGCCACTCAACCC
TCACAAATGGCCTCAAGTACTCTCTTGCGACTGGAAATTGGGGCGAGCAGAAGAAGGCG
ATGAGTTCCACCGCTGGTGTCTCGCAGGTGCTGAACCGGTATACGTTCTCCTCCACCCTA
TCGCATTTGCGGCGCACGAATACGCCTATCGGCCGTGACGGCAAGCTGGCCAAGCCTCG
TCAGCTGCACAATACACACTGGGGTCTCGTCTGTCCAGCAGAGACCCCCGAGGGACAGG
CTTGCGGTCTCGTCAAGAACCTGTCGTTAATGTGCTACGTGAGTGTCGGTTCACCGGCTG
AGCCTATCAAGGACTTCATGGTGCAGCGTAATATGGAAGTTTTGGAGGAATACGAGCCC
GGCTCCAGCCCTGACGCAACCAAGATCTTCATCAACGGCACGTGGGTTGGTGTGCACAA
TGAGCCAGCACACTTGGTCACGCTTGTGCAGGAGCTTCGGCGCAAATGCATCATTTCCC
ACGAAGTCTCCCTCGTTCGCGACATTCGCGACCGCGAGTTCAAGATCTTCTCCGACGCTG
GTCGTGTCATGCGGCCTCTCTTCGTTGTCAATCAAGATGACAATCCCGAGACGGGGGCC
CAGCAAGGCACCCTGGCTCTCACCAAGCACCATATTCGCCGTCTTGAGGAAGATGCTCA
GTATCATCGCAAGAAGCATGATGACGACTATTTCGGCTGGGATGGTCTTCAGAACAGCG
GTTGCATCGAGTACTTGGATGCCGAGGAGGAGGAGACAGCAATGATCTGCATGAGTCCT
GAGGATCTTGAGGACTACCGCCAGCGCAAGGCTAGGGGCAAGGATGCCAAGGATGAGG
AGCCGGAGGACGATGGCAGGAGTCTGAACGCTCGTGTGAAGACGAAGATCAACCCCGA
CATCCACATGTACACGCACTGCGAGATCCATCCGAGCATGCTCCTTGGAATCTGCGCCA
GCATCATCCCCTTCCCGGACCACAATCAGGTGTGTAAACTTCTCCCAGTAACTACTACCA
CTCCTGCTGACCGCATCTACCTAGTCACCTCGCATCAATCAAATCAGCACCATCACCATC
GCGCAATCGTCAAGATCAAGCCCAAATGTCCGCCATCTTTTCGTCGCGGCCGTTTACCCC
GGCCCGCGGGGAGGGGGGGTTGTCCCTTTTTCGCCCACCGCACATTTTCGCTTTGGTGCG
GGGTTTTGCGTCTGGGTCTTATCTCAGATAAACGTGACCCACCCAACCGCCACCTCAGTC

GTCCCCCGCCAACACACCCTTGCGCGCGCCTCTTTTTCTAACACTCGAAACTTGACAACG
ACAATGCTGACTCTAGTTTTCTACTACAGCTGCTGAGCTCGGAAAGGGTTCCTTCAAGTA
CGCATGGGTCCTTGACAAGCTCAAGGCCGAGCGTGAGCGTGGTATCACCATCGACATTG
CTCTGTGGAAGTTCGAGACTCCCAAGTACTATGTCACCGTCATTGGTAAGCTCTATCCCT
GCTGCTCTCGCGCAGGCCTGGCCGCCCCGCCTCACTACCCGGCCCTGCCAGGCGCGAGA
CCGCGACTTCTGATGCTTTTGCGCAACCCGTGCTAACGCAAGTGTTACTACAGACGCTCC
CGGTCACCGTGACTTCATCAAGAACATGATCACTGGTACCTCCCAGGCTGACTGCGCCA
TTCTCATCATTGCCTCCGGTACTGGTGAGTTCGAGGCTGGTATCTCCAAGGATGGCCAGA
CTCGTGAGCACGCTCTGCTCGCCTACACCCTCGGTGTCAAGCAGCTCATTGTTGCTTGCA
ACAAGATGGACACCGCTGGCTGGAAGCAGGAGCGTTTCGAGGAGATCAAGAAGGAGAC
CACCAACTTCATCAAGAAGGTTGGCTTCAACCCCAAGCAGGTCGCTTTCGTCCCCATCTC
CGGCTTCCACGGCGACAACATGCTTGAGGCCTCCTCCAACATGCCCTGGTACAAGGGCT
GGTCCAAGGAGACCCAGGACAAGAAGGAGGCCAAGGGCATGACCCTGCTCGATGCCAT
TGACGCCATTGAGCCCCCCAAGCGTCCCACCGAGAAGCCCCTGCGTCTGCCCCTCCAGG
ATGTCTACAAGATCGGCGGTATCGGAACTGTACCTGTCGGCCGTATCGAGACTGGTATC
CTGAAGCCCGGTATGGTCGTCACCTTCGCCCCCTCCAACGTCACCACTGAGGTCAAGTCC
GTGGAGATGCACCACGAGCAGCTCACCGAGGGTCAGCCTGGTGACAACGTTGGTTTCAA
CGTCAAGAACGTCTCCGTCAAGGAGATTCGCCGTGGCAACGTCGCTGGTGACTCCAAGA
ACGACCCCCCCATGGCCGCCGCCTCCTTCAACGCCCAGGTCATCGTCCTGAACCACCCCG
GCCAGGTCGGTGCTGGATAC

>Melanconiella_elegans

GTAACAAGGTCTCCGTTGGTGAACCAGCGGAGGGATCATTGAAAAATATGGAATATACG
TCCCATAAACCCTTTGTGAATATATACCTACCCTCGTTGCCTCGGCGTCAGGCCCGGGGT
TCCCCCCCAGGGGGGTTCCCCCCACCTACTACGGTAGGTGGAGCAGGCCCGCCGGTGGC
CCCCCTAAACTCTTGTTTTTAGAACCTATTCTCCTCTGAGTTTTTTATACCAAAATAAGTC
AAAACTTTCAACAACGGATCTCTTGGCTCTGGCATCGATGAAGAACGCAGCGAAATGCG
ATAAGTAATGTGAATTGCAGAATTCAGTGAATCATCGAATCTTTGAACGCACATTGCGC
CCGCTGGAATTCCGGCGGGCATGCCTGTTCGAGCGTCATTTCAACCCCTCAAGCTTTATC
GCTTGGTGTTGGGGCATAGCCTGTAAAAAGGCTAGCCCTTAAATTCAGTGGCGTGCTCG
CTGAGACTCCCGGGTGTAGTAATACTCCTTCTCGCCCGGGCTTGGTCTCAGCGGTGCACT
CGCCGTAAAACCCGCTATCACGCACCTTCTGAAAGTTGACCTCGGATCAGGTAGGAATA
CCCGCTGAACTTAAGCATATCAATAAGCGGAGGAAAAGAAACCAACAGGGATTGCCCT
AGTAACGGCGAGTGAAGCGGCAACAGCTCAAATTTGAAATCTGGCCTCCCCACGGGGGT
CCGAGTTGTAATTTGCAGAGGATGTCTTCTGGCGCGGTGCCTTCCGAGTTCCCTGGAACG
GGACGCCACAGAGGGTGAGAGCCCCGTAGCGGTCGGATACCAAGCCTGTGTGAAGCTC
CTTCGACGAGTCGAGTAGTTTGGGAATGCTGCTCTAAATGGGAGGTAAATCTCTTCTAA
AGCTAAATACTGGCCAGAGACCGATAGCGCACAAGTAGAGTGATCGAAAGATGAAAAG
CACCTTGAAAAGGGGGTTAAACAGTACGTGAAATTGTTGAAAGGGAAGCGCTCATGACC
AGACTTGCGCCGGGCGGCTCAGCAGGTGGTTCTGCCCCTGTGTACTCCGTCCCGGTTCAG
GCCAGCATCGGTTCTCGTTGGGGGATAAGAACGGTGGGAACGTGGCCCTCCCTCGGGGG
GGTGTTATAGCCCGCCGTAACGATACCCTGGCGGGGACCGAGGTTCGCGCATCTTGCAA
GGATGCTGGCGTAATGGTTATCAGCGACCCGTCTTGAAACACGGACCAAGGAGTCGTCC
TTTAGAGCGAGCGTCTGGGTGTCAAAACCCGCACGCGTAATGAAAGTGAAATTAGGTGA
GAGCCCTCACGGGCGCATCATCGACCGATCCTGATGTTCTCGGACGGATTTGAGTAAGA
GTTTTAACGGACGGACCCGAAAGACAGTGAACTATGCTTGTATAGGGTGAAGCCAGAGG
AAACTCTGGTGGAGGCTCGCAGCGGTTCTGACGTGCAAATCGATCGTCAAATATGAGCA

TGGGGGCGAAAGACTAATCGAACGTAACAAGGTCTCCGTTGGTGAACCAGCGGAGGGA
TCATTGAAAAATATGGAATATACGTCCCATAAACCCTTTGTGAATATATACCTACCCTCG
TTGCCTCGGCGTCAGGCCCGGGGTTCCCCCCCAGGGGGGTTCCCCCCACCTACTACGGTA
GGTGGAGCAGGCCCGCCGGTGGCCCCCCTAAACTCTTGTTTTAGAACCTATTCTCCTCT
GAGTTTTTTATACCAAAATAAGTCAAAACTTTCAACAACGGATCTCTTGGCTCTGGCATC
GATGAAGAACGCAGCGAAATGCGATAAGTAATGTGAATTGCAGAATTCAGTGAATCATC
GAATCTTTGAACGCACATTGCGCCCGCTGGAATTCCGGCGGGCATGCCTGTTCGAGCGT
CATTTCAACCCCTCAAGCTTTATCGCTTGGTGTTGGGGCATAGCCTGTAAAAAGGCTAGC
CCTTAAATTCAGTGGCGTGCTCGCTGAGACTCCCGGGTGTAGTAATACTCCTTCTCGCCC
GGGCTTGGTCTCAGCGGTGCACTCGCCGTAAAACCCGCTATCACGCACCTTCTGAAAGTT
GACCTCGGATCAGGTAGGAATACCCGCTGAACTTAAGCATATCAATAAGCGGAGGAAA
AGAAACCAACAGGGATTGCCCTAGTAACGGCGAGTGAAGCGGCAACAGCTCAAATTTG
AAATCTGGCCTCCCCACGGGGGTCCGAGTTGTAATTTGCAGAGGATGTCTTCTGGCGCG
GTGCCTTCCGAGTTCCCTGGAACGGGACGCCACAGAGGGTGAGAGCCCCGTAGCGGTCG
GATACCAAGCCTGTGTGAAGCTCCTTCGACGAGTCGAGTAGTTTGGGAATGCTGCTCTA
AATGGGAGGTAAATCTCTTCTAAAGCTAAATACTGGCCAGAGACCGATAGCGCACAAGT
AGAGTGATCGAAAGATGAAAAGCACCTTGAAAAGGGGGTTAAACAGTACGTGAAATTG
TTGAAAGGGAAGCGCTCATGACCAGACTTGCGCCGGGCGGCTCAGCAGGTGGTTCTGCC
CCTGTGTACTCCGTCCCGGTTCAGGCCAGCATCGGTTCTCGTTGGGGGATAAGAACGGT
GGGAACGTGGCCCTCCCTCGGGGGGGTGTTATAGCCCGCCGTAACGATACCCTGGCGGG
GACCGAGGTTCGCGCATCTTGCAAGGATGCTGGCGTAATGGTTATCAGCGACCCGTCTT
GAAACACGGACCAAGGAGTCGTCCTTTAGAGCGAGCGTCTGGGTGTCAAAACCCGCACG
CGTAATGAAAGTGAAATTAGGTGAGAGCCCTCACGGGCGCATCATCGACCGATCCTGAT
GTTCTCGGACGGATTTGAGTAAGAGTTTTAACGGACGGACCCGAAAGACAGTGAACTAT
GCTTGTATAGGGTGAAGCCAGAGGAAACTCTGGTGGAGGCTCGCAGCGGTTCTGACGTG
CAAATCGATCGTCAAATATGAGCATGGGGGCGAAAGACTAATCGAACTCCTGGCAAAG
CTGTTCCGAAACATCGTCAAGAAGCTGACCGGCGACATGATGGCGTACATGAAGCGTTG
CGTCAGCGAGGGCAAGCATTTCGATCTGGCGTTGGGCATCCGCCACTCAACCCTCACAA
ATGGCCTCAAGTACTCTCTTGCGACTGGAAATTGGGGCGAGCAGAAAAAGGCGATGAGT
TCCACCGCTGGTGTCTCGCAGGTGCTGAACCGGTATACGTTCTCCTCCACCCTATCGCAT
TTGCGGCGCACGAATACGCCTATCGGCCGTGACGGCAAGCTGGCCAAGCCTCGTCAGCT
GCACAATACACACTGGGGTCTCGTCTGTCCAGCAGAGACCCCCGAGGGACAGGCTTGCG
GTCTCGTCAAGAACCTGTCGTTAATGTGCTACGTGAGTGTCGGTTCACCGGCTGAGCCTA
TCAAGGACTTCATGGTGCAGCGTAATATGGAAGTTTTGGAGGAATACGAGCCCGGCTCC
AGCCCTGACGCAACCAAGATCTTCATCAACGGCACGTGGGTTGGTGTGCACAATGAGCC
AGCACACTTGGTCACGCTTGTGCAGGAGCTTCGGCGCAAATGCATCATTTCCCACGAAG
TCTCCCTCGTTCGCGACATTCGCGACCGCGAGTTCAAGATCTTCTCCGACGCTGGTCGTG
TCATGCGGCCTCTCTTCGTTGTCAATCAAGATGACAACCCCGAGACGGGGGCCCAGCAA
GGTACCCTGGCTCTCACCAAGCACCATATTCGCCGTCTTGAGGAAGATGCTCAGTACCAT
CGCAAGAAGCATGACGACGACTATTTCGGCTGGGATGGTCTTCAGAACAGCGGTTGCAT
CGAGTACTTGGATGCCGAGGAGGAGGAGACAGCAATGATCTGCATGAGTCCTGAGGAT
CTTGAGGACTACCGCCAGCGCAAGGCTAGGGGCAAGGATGCCAAGGATGAGGAGCCGG
AGGACGATGGCAGGAGTCTGAACGCTCGTGTGAAGACGAAGATCAACCCCGACATCCA
CATGTACACGCACTGATCAAATCAGCACCATCACCATCGCGCAATCGTCAAGATCAAGC
CCAAATGCCCGCCATCTTTTCGTCGCGACCGTTTACCCAGGTCTGCGGCGGGTGGGTTGT
CCCTTTTTCGCCCACCGCACATTTTCGCTTTGGTGCGGGGTTTTGCGTCTGGGTCTTATCT
CAGATAAACGTGACCCACCCAACCGCCACCTCAAGTCGTCCCCCGCCAACACACCCTTG

CGCGCGCCTCTTTTTCTGACACTCGAATCTTGACGACGACAATGCTGACTCTAGTTTTCT
ACTATAGCTGCTGAGCTCGGAAAGGGTTCCTTCAAGTACGCATGGGTCCTTGACAAGCT
CAAGGCCGAGCGTGAGCGTGGTATCACCATCGACATTGCTCTGTGGAAGTTCGAGACTC
CCAAGTACTATGTCACCGTCATTGGTAAGCTCTATCCCTGCTGCTCTCGCGCAGGCCCGG
CCGCCCCGCCTCACTACCCGGCCCTGCCAGGCGCGAGACCGCGACTTCTGATGCTTTTGC
GCAACCCGTGCTAACGCAAGTGTTACTACAGACGCTCCCGGTCACCGTGACTTCATCAA
GAACATGATCACTGGTACCTCCCAGGCTGACTGCGCCATTCTCATCATTGCCTCTGGTAC
TGGTGAGTTCGAGGCTGGTATCTCCAAGGATGGCCAGACTCGTGAGCACGCTTTGCTCG
CCTACACCCTCGGTGTCAAGCAGCTTATTGTTGCTTGCAACAAGATGGACACCGCTGGCT
GGAAGCAGGAGCGTTTCGAGGAGATCAAGAAGGAGACCACCAACTTCATCAAGAAGGT
TGGCTTCAACCCCAAGCAGGTCGCTTTCGTCCCCATCTCCGGCTTCCACGGCGACAACAT
GCTTGAGGCCTCCTCCAACATGCCCTGGTACAAGGGCTGGTCCAAGGAGACCCAGGACA
AGAAGGAGGCCAAGGGCATGACCCTGCTCGATGCCATTGACGCCATTGAGCCCCCCAAG
CGTCCCACCGAGAAGCCCCTGCGTCTGCCCCTCCAGGATGTCTACAAGATCGGCGGTAT
CGGAACTGTACCTGTCGGCCGTATCGAGACTGGTGTCCTGAAGCCCGGTATGGTCGTCA
CCTTCGCCCCCTCCAACGTCACCACTGAGGTCAAGTCCGTGGAGATGCACCACGAGCAG
CTCACCGAGGGTCAGCCTGGTGACAACGTTGGTTTCAACGTCAAGAACGTCTCCGTCAA
GGAGGTTCGCCGTGGCAACGTCGCTGGTGACTCCAAGAACGACCCCCCCATGGCCGCCG
CCTCCTTCAACGCCCAGGTCATCGTCCTGAACCACCCCGGCCAGGTCGGTGCTGGATAC

>Melanconiella_ellisii

AAAGTCGTAACAAGGTCTCCGTTGGTGAACCAGCGGAGGGATCATTGCTGGAACACGTC
CCCAGGGCGTACCCAGATACCCTTTGTGAACTTATACCTATATCGTTGCCTCGGCGCTGG
CTGGGAGCCCCTYGGGGCYCCCCCACGGTTCTCCGTGGAGCAGGCCCGCCGGCGGCCCT
CTAAACTCTTGTTTTTATAACGTATCTCTTCTGAGTAACAAAATTATAAATGAATCAAAA
CTTTCAACAACGGATCTCTTGGTTCTGGCATCGATGAAGAACGCAGCGAAATGCGATAA
GTAATGTGAATTGCAGAATTCAGTGAATCATCGAATCTTTGAACGCACATTGCGCCCGCT
GGTATTCCGGCGGGCATGCCTGTTCGAGCGTCATTTCAACCCTCAAGCTTCACCGCTTGG
TGTTGGGGCGTAGCCTGTARAAAGGCTAGCCCTGAAATTCAGTGGCGTGCTCGCTAAGA
CTCCGAGCGTAGTAATTTTTTCTCGCTTAGGTTGGATTAGCGGTGCTCAGCCGTAAAACC
TGCTATCATGCACTTCTGAAAGTTGACCTCGGATCAGGTAGGAATACCCGCTGAACTTA
AGCATATCAATAAGCGGAGGAAAAGAAACCAACAGGGATTGCCCTAGTAACGGCGAGT
GAAGCGGCAACAGCTCAAATTTGAAATCTGGCTTCGGCCCGAGTTGTAATTTGCAGAGG
ATGCTTCTGGCGCGGTGCCTTCCGAGTTCCCTGGAACGGGACGCCACAGAGGGTGAGAG
CCCCGTATGGTCGGACACCAAGCCTGTGTGAAGCTCCTTCGACGAGTCGAGTAGTTTGG
GAATGCTGCTCAAAATGGGAGGTAAATCTCTTCTAAAGCTAAATACCGGCCAGAGACCG
ATAGCGCACAAGTAGAGTGATCGAAAGATGAAAAGCACCTTGAAAAGGGGGTTAAACA
GTACGTGAAATTGTTGAAAGGGAAGCGCTTGTGACCAGACTTGTGCCGGGCGGCTCATC
AGGGGTTCTCCCCTGTGTACTCCGCCCGGTTCAGGCCAGCATCGGTTTTCGCTGGGGGAT
AAGAACGGTGGGAACGTGGCCCCCCCTCGGGGGGGTGTTATAGCCCGCCGTACGATACC
CTAGCGGGGACCGAGGTTCGCGCTCCGCAAGGATGCTGGCGTAATGGTCATCAGCGACC
CGTCTTGAAACACGGACCAAGGAGTCGTCCATTAGAGCGAGCGTTTGGGTGTAAAACCC
GCACGCGTAATGAAAGTGAAATTAGGTGAGAGCTTCGGCGCATCATCGACCGATCCTGA
TGTTCTCGGATGGATTTGAGTAAGAGTTTTAACGGACGGACCCGAAAGACAGTGAACTA
TGCTTGTATAGGGTGAAGCCAGAGGAAACTCTGGTGGAGGCTCGCAGCGGTTCTGACGT
GCAAATCGATCGTCAAATATGAGCATGGGGGCGAAAGACTAATCGAACAAAGTCGTAA
CAAGGTCTCCGTTGGTGAACCAGCGGAGGGATCATTGCTGGAACACGTCCCCAGGGCGT

ACCCAGATACCCTTTGTGAACTTATACCTATATCGTTGCCTCGGCGCTGGCTGGGAGCCC
CTYGGGGCYCCCCCACGGTTCTCCGTGGAGCAGGCCCGCCGGCGGCCCTCTAAACTCTT
GTTTTTATAACGTATCTCTTCTGAGTAACAAAATTATAAATGAATCAAAACTTTCAACAA
CGGATCTCTTGGTTCTGGCATCGATGAAGAACGCAGCGAAATGCGATAAGTAATGTGAA
TTGCAGAATTCAGTGAATCATCGAATCTTTGAACGCACATTGCGCCCGCTGGTATTCCGG
CGGGCATGCCTGTTCGAGCGTCATTTCAACCCTCAAGCTTCACCGCTTGGTGTTGGGGCG
TAGCCTGTARAAAGGCTAGCCCTGAAATTCAGTGGCGTGCTCGCTAAGACTCCGAGCGT
AGTAATTTTTCTCGCTTAGGTTGGATTAGCGGTGCTCAGCCGTAAAACCTGCTATCATG
CACTTCTGAAAGTTGACCTCGGATCAGGTAGGAATACCCGCTGAACTTAAGCATATCAA
TAAGCGGAGGAAAAGAAACCAACAGGGATTGCCCTAGTAACGGCGAGTGAAGCGGCAA
CAGCTCAAATTTGAAATCTGGCTTCGGCCCGAGTTGTAATTTGCAGAGGATGCTTCTGGC
GCGGTGCCTTCCGAGTTCCCTGGAACGGGACGCCACAGAGGGTGAGAGCCCCGTATGGT
CGGACACCAAGCCTGTGTGAAGCTCCTTCGACGAGTCGAGTAGTTTGGGAATGCTGCTC
AAAATGGGAGGTAAATCTCTTCTAAAGCTAAATACCGGCCAGAGACCGATAGCGCACA
AGTAGAGTGATCGAAAGATGAAAAGCACCTTGAAAAGGGGGTTAAACAGTACGTGAAA
TTGTTGAAAGGGAAGCGCTTGTGACCAGACTTGTGCCGGGCGGCTCATCAGGGGTTCTC
CCCTGTGTACTCCGCCCGGTTCAGGCCAGCATCGGTTTTCGCTGGGGGATAAGAACGGT
GGGAACGTGGCCCCCCCTCGGGGGGGTGTTATAGCCCGCCGTACGATACCCTAGCGGGG
ACCGAGGTTCGCGCTCCGCAAGGATGCTGGCGTAATGGTCATCAGCGACCCGTCTTGAA
ACACGGACCAAGGAGTCGTCCATTAGAGCGAGCGTTTGGGTGTAAAACCCGCACGCGTA
ATGAAAGTGAAATTAGGTGAGAGCTTCGGCGCATCATCGACCGATCCTGATGTTCTCGG
ATGGATTTGAGTAAGAGTTTTAACGGACGGACCCGAAAGACAGTGAACTATGCTTGTAT
AGGGTGAAGCCAGAGGAAACTCTGGTGGAGGCTCGCAGCGGTTCTGACGTGCAAATCG
ATCGTCAAATATGAGCATGGGGGCGAAAGACTAATCGAAC?????????????????????????????
???
???
???
???
???
???
???
???
???
???
???
???
???CATCTTTCAAATCATCATCATCACA
CTCGGCACAAACACCAAGGCTAATGGTCGCGATTCCTTCGTCGCGGCCGTTGGCCTTGG
CTGGCTGTGTCTTTTTTTCGCCCACCACACATTTTTGCTTTGGTGCGGGGTTTTGCGTCTG
GGTCTTATCTCAGATAAACGTGACCCACCCAACCGCCACCTCAGTCATCCCTCCGCCAAC
GCACCCATGCACACCACTCACATCCTCACACCATGACAACAACGATGCTGACTCCATCC
TTCTATCACAGCTGCTGAGCTCGGCAAGGGTTCCTTCAAGTACGCATGGGTCCTTGACAA
GCTCAAGGCCGAGCGTGAGCGTGGTATCACCATCGACATTGCTCTGTGGAAGTTCGAGA
CTCCCAAGTACTATGTCACCGTCATTGGTAAGCTTTCTCCCCCTGACGATGCGCTGCCCC
ACCATGGGCGACATGGCGGCCTCCTGACACTTGTGCGCAATCCGYGCTAACGCAAATGT
GACCACAGACGCTCCCGGTCACCGTGACTTCATCAAGAACATGATCACTGGTACCTCCC
AGGCTGACTGCGCCATTCTCATCATTGCCTCCGGTACTGGTGAGTTCGAGGCCGGTATCT

CCAAGGATGGCCAGACCCGYGAGCACGCTCTGCTCGCCTACACCCTCGGTGTCAAGCAG
CTCATTGTTGCTTGCAACAAGATGGACACCGCTGGCTGGAAGCAGGACCGTTTCGAGGA
GATCAAGAAGGAGACCACCAACTTCATCAAGAAGGTCGGCTTCAACCCCAAGCAGGTTG
CTTTCGTCCCCATCTCCGGCTTCCACGGCGACAACATGCTTGAGGCCTCCGCCAACATGC
CCTGGTACAAGGGCTGGACCAAGGAGACCCAGGACAAGAAGGAGGCCAAGGGCATGAC
CCTGCTCGACGCCATTGACGCCATCGAGCCCCCCAAGCGTCCTACCGAGAAGCCCCTGC
GTCTGCCCCTGCAGGATGTCTACAAGATCGGTGGTATTGGAACTGTACCTGTCGGCCGTA
TCGAGACTGGTGTCCTGAAGCCCGGTATGGTCGTCACCTTCGCTCCCTCCAACGTCACCA
CTGAGGTCAAGTCCGTTGAGATGCACCACGAGCAGCTCACTGAGGGTCTCCCCGGTGAC
AACGTTGGTTTCAACGTCAAGAACGTCTCCGTCAAGGAGATTCGCCGTGGCAACGTCGC
TGGTGACTCCAAGAACGACCCCCCTATGGCCGCCGCTTCCTTCAACGCCCAGGTCATTGT
CCTGAACCACCCCGGCCAGGTCGGCGCTGGATAC

>Melanconiella_ellisii

TCGTAACAAGGTCTCCGTTGGTGAACCAGCGGAGGGATCATTGCTGGAACACGTCCCCA
GGGCGTACCCAGATACCCTTTGTGAACTTATACCTATATCGTTGCCTCGGCGCTGGCTGG
GAGCCCCTCGSGGCTCCCCCACGGTTCTCCGTGGAGCAGGCCCGCCGGCGGCCCTRTAA
ACTCTTGTTTTTATAACGTATCTCTTCTGAGTAACAAAATTATAAATGAATCAAAACTTT
CAACAACGGATCTCTTGGTTCTGGCATCGATGAAGAACGCAGCGAAATGCGATAAGTAA
TGTGAATTGCAGAATTCAGTGAATCATCGAATCTTTGAACGCACATTGCGCCCGCTGGTA
TTCCGGCGGGCATGCCTGTTCGAGCGTCATTTCAACCCTCAAGCTTCACCGCTTGGTGTT
GGGGCGTAGCCTGTAAAAAGGCTAGCCCTGAAATTCAGTGGCGTGCTCGCTAAGACTCC
GAGCGTAGTAATTTTTTCTCGCTTAGGTTGGATTAGCGGTGCTCAGCCGTAAAACCTGCT
ATCATGCACTTCTGAAAGTTGACCTCGGATCAGGTAGGAATACCCGCTGAACTTAAGCA
TATCAATAAGCGGAGGAAAAGAAACCAACAGGGATTGCCCTAGTAACGGCGAGTGAAG
CGGCAACAGCTCAAATTTGAAATCTGGCTTCGGCCCGAGTTGTAATTTGCAGAGGATGC
TTCTGGCGCGGTGCCTTCCGAGTTCCCTGGAACGGGACGCCACAGAGGGTGAGAGCCCC
GTATGGTCGGACACCAAGCCTGTGTGAAGCTCCTTCGACGAGTCGAGTAGTTTGGGAAT
GCTGCTCAAAATGGGAGGTAAATCTCTTCTAAAGCTAAATACCGGCCAGAGACCGATAG
CGCACAAGTAGAGTGATCGAAAGATGAAAAGCACCTTGAAAAGGGGGTTAAACAGTAC
GTGAAATTGTTGAAAGGGAAGCGCTTGTGACCAGACTTGTGCCGGGCGGCTCATCAGGG
GTTCTCCCCTGTGTACTCCGCCCGGTTCAGGCCAGCATCGGTTTTCGCTGGGGGATAAGA
ACGGTGGGAACGTGGCCCCCCCTCGGGGGGGTGTTATAGCCCGCCGTACGATACCCTAG
CGGGGACCGAGGTTCGCGCTCCGCAAGGATGCTGGCGTAATGGTCATCAGCGACCCGTC
TTGAAACACGGACCAAGGAGTCGTCCATTAGAGCGAGCGTTTGGGTGTAAAACCCGCAC
GCGTAATGAAAGTGAAATTAGGTGAGAGCTTCGGCGCATCATCGACCGATCCTGATGTT
CTCGGATGGATTTGAGTAAGAGTTTTAACGGACGGACCCGAAAGACAGTGAACTATGCT
TGTATAGGGTGAAGCCAGAGGAAACTCTGGTGGAGGCTCGCAGCGGTTCTGACGTGCAA
ATCGATCGTCAAATATGAGCATGGGGGCGAAAGACTAATCGAACTCGTAACAAGGTCTC
CGTTGGTGAACCAGCGGAGGGATCATTGCTGGAACACGTCCCCAGGGCGTACCCAGATA
CCCTTTGTGAACTTATACCTATATCGTTGCCTCGGCGCTGGCTGGGAGCCCCTCGSGGCT
CCCCCACGGTTCTCCGTGGAGCAGGCCCGCCGGCGGCCCTRTAAACTCTTGTTTTTATAA
CGTATCTCTTCTGAGTAACAAAATTATAAATGAATCAAAACTTTCAACAACGGATCTCTT
GGTTCTGGCATCGATGAAGAACGCAGCGAAATGCGATAAGTAATGTGAATTGCAGAATT
CAGTGAATCATCGAATCTTTGAACGCACATTGCGCCCGCTGGTATTCCGGCGGGCATGC
CTGTTCGAGCGTCATTTCAACCCTCAAGCTTCACCGCTTGGTGTTGGGGCGTAGCCTGTA
AAAAGGCTAGCCCTGAAATTCAGTGGCGTGCTCGCTAAGACTCCGAGCGTAGTAATTTT

TTCTCGCTTAGGTTGGATTAGCGGTGCTCAGCCGTAAAACCTGCTATCATGCACTTCTGA
AAGTTGACCTCGGATCAGGTAGGAATACCCGCTGAACTTAAGCATATCAATAAGCGGAG
GAAAAGAAACCAACAGGGATTGCCCTAGTAACGGCGAGTGAAGCGGCAACAGCTCAAA
TTTGAAATCTGGCTTCGGCCCGAGTTGTAATTTGCAGAGGATGCTTCTGGCGCGGTGCCT
TCCGAGTTCCCTGGAACGGGACGCCACAGAGGGTGAGAGCCCCGTATGGTCGGACACCA
AGCCTGTGTGAAGCTCCTTCGACGAGTCGAGTAGTTTGGGAATGCTGCTCAAAATGGGA
GGTAAATCTCTTCTAAAGCTAAATACCGGCCAGAGACCGATAGCGCACAAGTAGAGTGA
TCGAAAGATGAAAAGCACCTTGAAAAGGGGGTTAAACAGTACGTGAAATTGTTGAAAG
GGAAGCGCTTGTGACCAGACTTGTGCCGGGCGGCTCATCAGGGGTTCTCCCCTGTGTACT
CCGCCCGGTTCAGGCCAGCATCGGTTTTCGCTGGGGGATAAGAACGGTGGGAACGTGGC
CCCCCCTCGGGGGGGTGTTATAGCCCGCCGTACGATACCCTAGCGGGGACCGAGGTTCG
CGCTCCGCAAGGATGCTGGCGTAATGGTCATCAGCGACCCGTCTTGAAACACGGACCAA
GGAGTCGTCCATTAGAGCGAGCGTTTGGGTGTAAAACCCGCACGCGTAATGAAAGTGAA
ATTAGGTGAGAGCTTCGGCGCATCATCGACCGATCCTGATGTTCTCGGATGGATTTGAGT
AAGAGTTTTAACGGACGGACCCGAAAGACAGTGAACTATGCTTGTATAGGGTGAAGCCA
GAGGAAACTCTGGTGGAGGCTCGCAGCGGTTCTGACGTGCAAATCGATCGTCAAATATG
AGCATGGGGGCGAAAGACTAATCGAACGGTCCGCTCTTGGCAAAGCTGTTCCGAAACAT
CGTCAAGAAGCTGACGGGTGACATGATGGCGTACATGAAGCGTTGTGTCAGCGAGGGC
AAGCATTTCGATTTGGCATTGGGCATTCGCCACTCCACCCTCACAAATGGTCTGAAGTAC
TCTCTCGCGACTGGAAACTGGGGTGAGCAGAAGAAGGCGATGAGTTCTACCGCTGGTGT
CTCGCAGGTGCTAAACCGGTACACGTTTTCGTCTACGTTATCGCATTTGCGGCGTACGAA
CACGCCCATCGGCCGTGACGGCAAGCTGGCCAAGCCTCGTCAGCTGCACAACACTCACT
GGGGTCTGGTTTGTCCAGCAGAGACTCCTGAGGGACAGGCTTGCGGTCTCGTCAAGAAC
CTGTCTTTAATGTGCTACGTCAGCGTTGGCTCACCGGCCGAGCCTATCAAGGACTTCATG
GTGCAGAGGAACATGGAAGTTTTGGAAGAATACGAGCCCGGCTCCAGCCCTGACGCGA
CCAAGATCTTCATCAACGGCACGTGGGTTGGTGTCCACAACGAACCAGCACATTTGGTC
ACGCTTGTGCAAGAGCTTCGACGCAAATGTATCATTTCTCACGAAGTCTCTCTCGTTCGC
GACATTCGTGATCGCGAGTTCAAGATCTTTTCCGACGCTGGTCGTGTGATGCGGCCCCTC
TTCGTCGTCAACCAAGAGGACAACCCCGAGACTGGAGCGCAGCAGGGCACTCTGGCTCT
CACCAAGCACCACATTCGCCGTCTCGAGGAAGATGCTCAGTACCAGCGCAAGAAGCATG
ATGAAGACTATTTTGGCTGGGATGGTCTTCAGAACAGTGGCTGCATCGAGTACCTGGAT
GCCGAGGAAGAGGAAACAGCAATGATCTGCATGAGTCCTGAGGATCTTGAGGACTACC
GGCAGCGGAAGGCCAGGGGCAAGGATGCCAAGGATGATGAGCCTGAGGATGATGGCAG
GAGTCTGAACGCTCGTGTGAAGACGAAGATCAACACCGACATCCACATGTATACCCATT
GCGAGATCCATCCGAGCATGCTCTTGGGAATCTGTGCCAGCATCATTCCCTTCCCGGACC
ACAATCAGGTATGTGAAAGCTTCTCAGTACTACCATGACTCCTGCTGACCACATATATCT
AGTCGCCCCGCATCTATCAAATCATCATCATCACACTCGGCACAAACACCAAGGCTAAT
GGTCGCGATTCCTTCGTCGCGGCCGTTGGCCTTGGCTGGCTGTGTCTTTTTTTCGCCCACC
ACACATTTTTGCTTTGGTGCGGGGTTTTGCGTCTGGGTCTTATCTCAGATAAACGTGACC
CACCCAACCGCCACCTCAGTCATCCCTCCGCCAACGCACCCATGCACACCACTCACATCC
TCACACCATGACAACAACGATGCTGACTCCATCCTTCTATCACAGCTGCTGAGCTCGGCA
AGGGTTCCTTCAAGTACGCATGGGTCCTTGACAAGCTCAAGGCCGAGCGTGAGCGTGGT
ATCACCATCGACATTGCTCTGTGGAAGTTCGAGACTCCCAAGTACTATGTCACCGTCATT
GGTAAGCTTTCTCCTCCCCCTGACGATGCGCTGCCCCACCATGGGCGACATGGCGGCCTC
CTGACACTTGTGCGCAATCCGTGCTAACGCAAATGTGACCACAGACGCTCCCGGTCACC
GTGACTTCATCAAGAACATGATCACTGGTACCTCCCAGGCTGACTGCGCCATTCTCATCA
TTGCCTCCGGTACTGGTGAGTTCGAGGCCGGTATCTCCAAGGATGGCCAGACCCGTGAG

CACGCTCTGCTCGCCTACACCCTCGGTGTCAAGCAGCTCATTGTTGCTTGCAACAAGATG
GACACCGCTGGCTGGAAGCAGGACCGTTTCGAGGAGATCAAGAAGGAGACCACCAACT
TCATCAAGAAGGTCGGCTTCAACCCCAAGCAGGTTGCTTTCGTCCCCATCTCCGGCTTCC
ACGGCGACAACATGCTTGAGGCCTCCGCCAACATGCCCTGGTACAAGGGCTGGACCAAG
GAGACCCAGGACAAGAAGGAGGCCAAGGGCATGACCCTGCTCGACGCCATTGACGCCA
TCGAGCCCCCCAAGCGTCCCACCGAGAAGCCCCTGCGTCTGCCCCTGCAGGATGTCTAC
AAGATCGGTGGTATTGGAACTGTACCTGTCGGCCGTATCGAGACTGGTGTCCTGAAGCC
CGGTATGGTCGTCACCTTCGCTCCCTCCAACGTCACCACTGAGGTCAAGTCCGTTGAGAT
GCACCACGAGCAGCTCACTGAGGGTCTCCCCGGTGACAACGTTGGTTTCAACGTCAAGA
ACGTCTCCGTCAAGGAAATTCGCCGTGGCAACGTCGCTGGTGACTCCAAGAACGACCCC
CCTATGGCCGCCGCTTCCTTCAACGCCCAGGTCATTGTCCTGAACCACCCCGGCCAGGTC
GGCGCTGGATAC

>Melanconiella_ellisii

GACCACCCAGGGCCGGAAAGTTCTCCAAACTCGATCATTTAGAGGAAGTAAAAGTCGTA
ACAAGGTCTCCGTTGGTGAACCAGCGGAGGGATCATTGCTGGAACACGTCCCCAGGGCG
TACCCAGATACCCTTTGTGAACTTATACCTATATCGTTGCCTCGGCGCTGGCCGGGAGCC
CCTTGGGGCCCCCCCACGGTTCTCCGTGGAGCAGGCCCGCCGGCGGCCCTCTAAACTCTT
GTTTTTATAACGTATCTCTTCTGAGTAACAAAATTATAAATGAATCAAAACTTTCAACAA
CGGATCTCTTGGTTCTGGCATCGATGAAGAACGCAGCGAAATGCGATAAGTAATGTGAA
TTGCAGAATTCAGTGAATCATCGAATCTTTGAACGCACATTGCGCCCGCTGGTATTCCGG
CGGGCATGCCTGTTCGAGCGTCATTTCAACCCTCAAGCTTTACCGCTTGGTGTTGGGGCG
TAGCCTGTAAAAAGGCTAGCCCTGAAATTCAGTGGCGTGCTCGCTAAGACTCCGAGCGT
AGTAATTTTTTCTCGCTTAGGTTGGATTAGCGGTGCTCAGCCGTAAAACCTGCTATCATG
CACTTCTGAAAGTTGACCTCGGATCAGGTAGGAATACCCGCTGAACTTAAGCATATCAA
TAAGCGGAGGAAAAGAAACCAACAGGGATTGCCCTAGTAACGGCGAGTGAAGCGGCAA
CAGCTCAAATTTGAAATCTGGCTTCGGCCCGAGTTGTAATTTGCAGAGGATGCTTCTGGC
GCGGTGCCTTCCGAGTTCCCTGGAACGGGACGCCACAGAGGGTGAGAGCCCCGTATGGT
CGGACACCAAGCCTGTGTGAAGCTCCTTCGACGAGTCGAGTAGTTTGGGAATGCTGCTC
AAAATGGGAGGTAAATCTCTTCTAAAGCTAAATACCGGCCAGAGACCGATAGCGCACA
AGTAGAGTGATCGAAAGATGAAAAGCACCTTGAAAAGGGGGTTAAACAGTACGTGAAA
TTGTTGAAAGGGAAGCGCTTGTGACCAGACTTGTGCCGGGCGGCTCATCAGGGGTTCTC
CCCTGTGTACTCCGCCCGGTTCAGGCCAGCATCGGTTTTCGCTGGGGGATAAGAACGGT
GGGAACGTGGCCCCCCCTCGGGGGGGTGTTATAGCCCGCCGTACGATACCCTAGCGGGG
ACCGAGGTTCGCGCTCCGCAAGGATGCTGGCGTAATGGTCATCAGCGACCCGTCTTGAA
ACACGGACCAAGGAGTCGTCCATTAGAGCGAGCGTTTGGGTGTAAAACCCGCACGCGTA
ATGAAAGTGAAATTAGGTGAGAGCTTCGGCGCATCATCGACCGATCCTGATGTTCTCGG
ATGGATTTGAGTAAGAGTTTTAACGGACGGACCCGAAAGACAGTGAACTATGCTTGTAT
AGGGTGAAGCCAGAGGAAACTCTGGTGGAGGCTCGCAGCGGTTCTGACGTGCAAATCG
ATCGTCAAATATGAGCATGGGGGCGAAAGACTAATCGAACGACCACCCAGGGCCGGAA
AGTTCTCCAAACTCGATCATTTAGAGGAAGTAAAAGTCGTAACAAGGTCTCCGTTGGTG
AACCAGCGGAGGGATCATTGCTGGAACACGTCCCCAGGGCGTACCCAGATACCCTTTGT
GAACTTATACCTATATCGTTGCCTCGGCGCTGGCCGGGAGCCCCTTGGGGCCCCCCCACG
GTTCTCCGTGGAGCAGGCCCGCCGGCGGCCCTCTAAACTCTTGTTTTTATAACGTATCTC
TTCTGAGTAACAAAATTATAAATGAATCAAAACTTTCAACAACGGATCTCTTGGTTCTGG
CATCGATGAAGAACGCAGCGAAATGCGATAAGTAATGTGAATTGCAGAATTCAGTGAAT
CATCGAATCTTTGAACGCACATTGCGCCCGCTGGTATTCCGGCGGGCATGCCTGTTCGAG

CGTCATTTCAACCCTCAAGCTTTACCGCTTGGTGTTGGGGCGTAGCCTGTAAAAAGGCTA
GCCCTGAAATTCAGTGGCGTGCTCGCTAAGACTCCGAGCGTAGTAATTTTTCTCGCTTA
GGTTGGATTAGCGGTGCTCAGCCGTAAAACCTGCTATCATGCACTTCTGAAAGTTGACCT
CGGATCAGGTAGGAATACCCGCTGAACTTAAGCATATCAATAAGCGGAGGAAAAGAAA
CCAACAGGGATTGCCCTAGTAACGGCGAGTGAAGCGGCAACAGCTCAAATTTGAAATCT
GGCTTCGGCCCGAGTTGTAATTTGCAGAGGATGCTTCTGGCGCGGTGCCTTCCGAGTTCC
CTGGAACGGGACGCCACAGAGGGTGAGAGCCCCGTATGGTCGGACACCAAGCCTGTGT
GAAGCTCCTTCGACGAGTCGAGTAGTTTGGGAATGCTGCTCAAAATGGGAGGTAAATCT
CTTCTAAAGCTAAATACCGGCCAGAGACCGATAGCGCACAAGTAGAGTGATCGAAAGA
TGAAAAGCACCTTGAAAAGGGGGTTAAACAGTACGTGAAATTGTTGAAAGGGAAGCGC
TTGTGACCAGACTTGTGCCGGGCGGCTCATCAGGGGTTCTCCCCTGTGTACTCCGCCCGG
TTCAGGCCAGCATCGGTTTTCGCTGGGGGATAAGAACGGTGGGAACGTGGCCCCCCCTC
GGGGGGGTGTTATAGCCCGCCGTACGATACCCTAGCGGGGACCGAGGTTCGCGCTCCGC
AAGGATGCTGGCGTAATGGTCATCAGCGACCCGTCTTGAAACACGGACCAAGGAGTCGT
CCATTAGAGCGAGCGTTTGGGTGTAAAACCCGCACGCGTAATGAAAGTGAAATTAGGTG
AGAGCTTCGGCGCATCATCGACCGATCCTGATGTTCTCGGATGGATTTGAGTAAGAGTTT
TAACGGACGGACCCGAAAGACAGTGAACTATGCTTGTATAGGGTGAAGCCAGAGGAAA
CTCTGGTGGAGGCTCGCAGCGGTTCTGACGTGCAAATCGATCGTCAAATATGAGCATGG
GGGCGAAAGACTAATCGAAC??
??
??
??
??
??
??
??
??
??
??
??
??
??
??
??
??
??
??
??
??
??
??
??
??
??
??
??

???
??

>Melanconiella_flavovirens

CACACCGCCCGTCGCTACTACCGATTGAATGGTTCAGTGAGGCGTTCGGACTGGCCCAG
GGAGGTCGGCAACGACCACCCAGGGCCGGAAAGTTCTCCAAACTCGATCATTTAGAGGA
AGTAAAAGTCGTAACAAGGTCTCCGTTGGTGAACCAGCGGAGGGATCATTGATGGAAA
ACACATTCCTATACCCCTTTGTGAACTTATACCTATTCTCGTTGCCTCGGCGCCAGGCCT
GGGGTTCCCCCTCGGGGGTGCCCCCCACCCAAGGGACCTTACGGTCCCGGGGGCGGAGC
AGGCCCGCCGGTGGCCCACCTAAACTCTTGTTTAGAACCTGTCTCCTCTGAGTTTGTTA
CCTAGAAAATGAATCAAAACTTTCAACAACGGATCTCTTGGCTCTGGCATCGATGAAGA
ACGCAGCGAAATGCGATAAGTAATGTGAATTGCAGAATTCAGTGAATCATCGAATCTTT
GAACGCACATTGCGCCCGCTGGAATTCCGGCGGGCATGCCTGTTCGAGCGTCATTTCAA
CCCCTCAAGCTTCAGCGCTTGGTGTTGGGGCATAGCCTGTGAAAAGGCTAGCCCTCAAA
TTCAGTGGCGTGCTCGCTGAGACCCCCGGGTGTAGTAATCTCCTTCTCGCCCGGGACGGC
TCAGCGGTGTACCTGCCGTAAAACCCGCTATCACGCACCTTCTGAAAGTTGACCTCGGAT
CAGGTAGGAATACCCGCTGAACTTAAGCATATCAATAAGCGGAGGAAAAGAAACCAAC
AGGGATTGCCCTAGTAACGGCGAGTGAAGCGGCAACAGCTCAAATTTGAAATCTGGTCC
CCCCTGGGGTCCCGAGTTGTAATTTGCAGAGGATGTCTTCTGGCGCGGTGCCTTCCGAGT
TCCCTGGAACGGGACGCCACAGAGGGTGAGAGCCCCGTAATGGTCGGATACCAAGCCT
GTGTGAAGCTCCTTCGACGAGTCGAGTAGTTTGGGAATGCTGCTCTAAATGGGAGGTAA
ATCTCTTCTAAAGCTAAATACTGGCCAGAGACCGATAGCGCACAAGTAGAGTGATCGAA
AGATGAAAAGCACCTTGAAAAGGGGGTTAAACAGTACGTGAAATTGTTGAAAGGGAAG
CGCTCATGACCAGACTTGCGCCGGGCGGCTCAGCAGGGGTTCTGCCCCTGTGTACTCCGT
CCCGGTTCAGGCCAGCATCGGTTCTCGCTGGGGGATAAGAGCGGTGGAACGTGGCCCC
CCTTCGGGGGGGTGTTATAGCCCGCCGTTATGATACCCTGGCGGGGACCGAGGTTCGCG
CATTCGCAAGGATGCTGGCGTAATGGTTATCAGTGACCCGTCTTGAAACACGGACCAAG
GAGTCGTCCTTTAGAGCGAGCGTCTGGGTGTTAAAACCCGCACGCGTAATGAAAGTGAA
ATTAGGTGAGAGCCCTCACGGGCGCATCATCGACCGATCCTGATGTTCTCGGATGGATTT
GAGTAAGAGTTTTAACGGACGGACCCGAAAGACAGTGAACTATGCTTGTATAGGGTGAA
GCCAGAGGAAACTCTGGTGGAGGCTCGCAGCGGTTCTGACGTGCAAATCGATCGTCAAA
TATGAGCATGGGGGCGAAAGACTAATCCACACCGCCCGTCGCTACTACCGATTGAATGG
TTCAGTGAGGCGTTCGGACTGGCCCAGGGAGGTCGGCAACGACCACCCAGGGCCGGAA
AGTTCTCCAAACTCGATCATTTAGAGGAAGTAAAAGTCGTAACAAGGTCTCCGTTGGTG
AACCAGCGGAGGGATCATTGATGGAAAACACATTCCTATACCCCTTTGTGAACTTATAC
CTATTCTCGTTGCCTCGGCGCCAGGCCTGGGGTTCCCCCTCGGGGGTGCCCCCCACCCAA
GGGACCTTACGGTCCCGGGGGCGGAGCAGGCCCGCCGGTGGCCCACCTAAACTCTTGTT
TTAGAACCTGTCTCCTCTGAGTTTGTTACCTAGAAAATGAATCAAAACTTTCAACAACGG
ATCTCTTGGCTCTGGCATCGATGAAGAACGCAGCGAAATGCGATAAGTAATGTGAATTG
CAGAATTCAGTGAATCATCGAATCTTTGAACGCACATTGCGCCCGCTGGAATTCCGGCG
GGCATGCCTGTTCGAGCGTCATTTCAACCCCTCAAGCTTCAGCGCTTGGTGTTGGGGCAT
AGCCTGTGAAAAGGCTAGCCCTCAAATTCAGTGGCGTGCTCGCTGAGACCCCCGGGTGT
AGTAATCTCCTTCTCGCCCGGGACGGCTCAGCGGTGTACCTGCCGTAAAACCCGCTATCA
CGCACCTTCTGAAAGTTGACCTCGGATCAGGTAGGAATACCCGCTGAACTTAAGCATAT
CAATAAGCGGAGGAAAAGAAACCAACAGGGATTGCCCTAGTAACGGCGAGTGAAGCGG
CAACAGCTCAAATTTGAAATCTGGTCCCCCCTGGGGTCCCGAGTTGTAATTTGCAGAGG
ATGTCTTCTGGCGCGGTGCCTTCCGAGTTCCCTGGAACGGGACGCCACAGAGGGTGAGA

GCCCCGTAATGGTCGGATACCAAGCCTGTGTGAAGCTCCTTCGACGAGTCGAGTAGTTT
GGGAATGCTGCTCTAAATGGGAGGTAAATCTCTTCTAAAGCTAAATACTGGCCAGAGAC
CGATAGCGCACAAGTAGAGTGATCGAAAGATGAAAAGCACCTTGAAAAGGGGGTTAAA
CAGTACGTGAAATTGTTGAAAGGGAAGCGCTCATGACCAGACTTGCGCCGGGCGGCTCA
GCAGGGGTTCTGCCCCTGTGTACTCCGTCCCGGTTCAGGCCAGCATCGGTTCTCGCTGGG
GGATAAGAGCGGTGGGAACGTGGCCCCCCTTCGGGGGGGTGTTATAGCCCGCCGTTATG
ATACCCTGGCGGGGACCGAGGTTCGCGCATTCGCAAGGATGCTGGCGTAATGGTTATCA
GTGACCCGTCTTGAAACACGGACCAAGGAGTCGTCCTTTAGAGCGAGCGTCTGGGTGTT
AAAACCCGCACGCGTAATGAAAGTGAAATTAGGTGAGAGCCCTCACGGGCGCATCATC
GACCGATCCTGATGTTCTCGGATGGATTTGAGTAAGAGTTTTAACGGACGGACCCGAAA
GACAGTGAACTATGCTTGTATAGGGTGAAGCCAGAGGAAACTCTGGTGGAGGCTCGCAG
CGGTTCTGACGTGCAAATCGATCGTCAAATATGAGCATGGGGGCGAAAGACTAATCGGT
CCGCTCCTGGCAAAGCTGTTCCGAAACATCGTCAAGAAGCTGACCGGCGACATGATGGC
GTACATGAAGCGTTGCGTCAGCGAGGGCAAGCATTTCGATCTGGCGTTGGGCATCCGCC
ACTCAACCCTCACAAATGGCCTCAAGTACTCTCTCGCGACAGGAAACTGGGGCGAGCAG
AAGAAGGCCATGAGTTCCACCGCTGGTGTCTCACAGGTGCTGAACCGGTACACGTTCTC
GTCTACCCTATCGCATTTGCGGCGCACGAACACGCCTATCGGTCGTGACGGCAAGCTCG
CCAAGCCTCGTCAGCTGCACAATACACACTGGGGTCTCGTCTGTCCTGCAGAGACTCCC
GAGGGACAGGCTTGTGGTCTCGTCAAGAACCTATCGTTAATGTGCTACGTTAGTGTTGGT
TCACCGGCTGAGCCTATCAAGGACTTCATGGTGCAGCGTAATATGGAAGTCTTGGAGGA
ATACGAGCCCGGCTCCAGTCCTGACGCAACCAAGATCTTCATCAACGGCACGTGGGTTG
GTGTGCACAATGAACCAGCACACTTGGTCACGCTTGTGCAGGAGCTCCGGCGCAAATGC
ATCATTTCTCACGAAGTCTCCCTCGTTCGCGACATTCGCGACCGCGAGTTCAAGATCTTC
TCTGACGCTGGTCGTGTCATGCGGCCTCTCTTTGTTGTCAATCAAGAGGATAATCCCGAG
ACTGGAGCGCAGCAGGGTACTCTGGCTCTCACCAAGCACCACATTCGCCGTCTCGAGGA
AGATGCTCAATACCATCGCAAGAAGCATGATGACGACTATTTCGGCTGGGATGGTCTTC
AGAACAGCGGTTGCATCGAGTACCTGGATGCCGAGGAAGAGGAGACAGCAATGATCTG
CATGAGTCCTGAGGATCTTGAGGACTACCGTCAGCGCAAGGCTAGGGGCAAGGATGCCA
AGGATGACGAACCGGAGGACGATGGCAGGAGTCTGAACGCTCGTGTGAAGACGAAGAT
CAACCCCGACATCCACATGTACACCCACTGCGAGATCCATCCGAGCATGCTCCTGGGAA
TATGCGCCAGCATTATTCCCTTCCCGGATCACAATCAGGTGTGTAAATTTCTCGCAGTAC
TAATTTCCCTCCTGCTGACCACATATATCTAGTCACCTCGTCACCAAACCAACATCATCA
CATCACCATGGCGCAAACATCAAGATCACGCCAAAAATGGTCGCCATCTCTTCGTCGCG
GCCCTTTGTACTGCCCACCTGGCGGGTGCCCTTTTTTCGCCCACCGCACATTTTCGCTTTG
TGGTGCGGGGTTTTGCGTCTGGGTCTTATCTCAGATAAACGTGACCCACCAAACCGCCAC
CTCATTCATCCTCCGCCAACACCCCCTTGCGTGTGCCTCTTTTTTCACCCTCAGAAATTTG
ACAACGACAATGCTGACTCGGATTTTTTACTGCAGCTGCTGAGCTCGGCAAGGGTTCCTT
CAAGTACGCATGGGTCCTTGACAAGCTCAAGGCCGAGCGTGAGCGTGGTATCACCATCG
ACATTGCTCTGTGGAAGTTCGAGACTCCCAAGTACTATGTCACCGTCATTGGTAAGCTCT
ACCCCCTGGTGTTGTCGCGCAGGCCTGTTTGTCCCGCCTCACTGCGCTGTCGCGCCATGC
GCGACACCGCCGCTTTTGAAGCTTTTGCGCAACCCGTGCTAACGCAAATGTCACCACAG
ACGCTCCCGGTCACCGTGACTTCATCAAGAACATGATCACTGGTACCTCCCAGGCCGAC
TGCGCCATTCTCATCATTGCCTCCGGTACTGGTGAGTTTGAGGCTGGTATCTCCAAGGAT
GGCCAGACCCGTGAGCACGCTCTGCTCGCCTACACCCTCGGTGTCAAGCAGCTCATTGTT
GCTTGCAACAAGATGGATACCGCTGGCTGGAAGCAGGACCGTTTTGAGGAGATCAAGA
AGGAGACCACCAACTTCATCAAGAAGGTCGGCTTCAACCCCAAGCAGGTCGCTTTCGTC
CCCATCTCCGGCTTCCACGGCGACAACATGCTTGAGGCCTCCTCCAACATGCCCTGGTAC

AAGGGCTGGACCAAGGAGACCCAGGACAAGAAGGAGGCCAAGGGTATGACCCTGCTCG
ACGCCATTGACTCCATCGAGCCCCCAAGCGCCCACCGAGAAGCCCCTGCGTCTGCCC
CTGCAGGACGTCTACAAGATCGGCGGTATTGGAACCGTACCCGTCGGCCGTATCGAGAC
TGGTATCCTGAAGCCCGGTATGGTCGTCACCTTCGCCCCCTCCAACGTCACCACTGAGGT
CAAGTCCGTGGAGATGCACCACGAGCAGCTCACCGAGGGCCAGCCCGGTGACAACGTT
GGTTTCAACGTCAAGAACGTCTCCGTCAAGGAGATTCGTCGTGGCAACGTCGCTGGTGA
CTCCAAGAACGACCCCCCCTTGGGCGCCGCTTCCTTCAACGCCCAGGTCATCGTTCTCAA
CCACCCCGGCCAGGTCGGTGCTGGATAC

>Melanconiella_flavovirens

CACACCGCCCGTCGCTACTACCGATTGAATGGTTCAGTGAGGCGTTCGGACTGGCCCAG
GGAGGTCGGCAACGACCACCCAGGGCCGGAAAGTTCTCCAAACTCGATCATTTAGAGGA
AGTAAAAGTCGTAACAAGGTCTCCGTTGGTGAACCAGCGGAGGGATCATTGATGGAAA
ACACATTCCTATACCCCTTTGTGAACTTATACCTATTCTCGTTGCCTCGGCGCCAGGCCT
GGGGTTCCCCCTCGGGGGTGCCCCCCACCCAAGGGACCTTACGGTCCCGGGGGCGGAGC
AGGCCCGCCGGTGGCCCACCTAAACTCTTGTTTTAGAACCTGTCTCCTCTGAGTTTGTTA
CCTAGAAAATGAATCAAAACTTTCAACAACGGATCTCTTGGCTCTGGCATCGATGAAGA
ACGCAGCGAAATGCGATAAGTAATGTGAATTGCAGAATTCAGTGAATCATCGAATCTTT
GAACGCACATTGCGCCCGCTGGAATTCCGGCGGGCATGCCTGTTCGAGCGTCATTTCAA
CCCCTCAAGCTTCAGCGCTTGGTGTTGGGGCATAGCCTGTGAAAAGGCTAGCCCTCAAA
TTCAGTGGCGTGCTCGCTGAGACCCCCGGGTGTAGTAATCTCCTTCTCGCCCGGGACGGC
TCAGCGGTGTACCTGCCGTAAAACCCGCTATCACGCACCTTCTGAAAGTTGACCTCGGAT
CAGGTAGGAATACCCGCTGAACTTAAGCATATCAATAAGCGGAGGAAAAGAAACCAAC
AGGGATTGCCCTAGTAACGGCGAGTGAAGCGGCAACAGCTCAAATTTGAAATCTGGTCC
CCCCTGGGGTCCCGAGTTGTAATTTGCAGAGGATGTCTTCTGGCGCGGTGCCTTCCGAGT
TCCCTGGAACGGGACGCCACAGAGGGTGAGAGCCCCGTAATGGTCGGATACCAAGCCT
GTGTGAAGCTCCTTCGACGAGTCGAGTAGTTTGGGAATGCTGCTCTAAATGGGAGGTAA
ATCTCTTCTAAAGCTAAATACTGGCCAGAGACCGATAGCGCACAAGTAGAGTGATCGAA
AGATGAAAAGCACCTTGAAAAGGGGGTTAAACAGTACGTGAAATTGTTGAAAGGGAAG
CGCTCATGACCAGACTTGCGCCGGGCGGCTCAGCAGGGGTTCTGCCCCTGTGTACTCCGT
CCCGGTTCAGGCCAGCATCGGTTCTCGCTGGGGGATAAGAGCGGTGGGAACGTGGCCCC
CCTTCGGGGGGGTGTTATAGCCCGCCGTTATGATACCCTGGCGGGGACCGAGGTTCGCG
CATTCGCAAGGATGCTGGCGTAATGGTTATCAGTGACCCGTCTTGAAACACGGACCAAG
GAGTCGTCCTTTAGAGCGAGCGTCTGGGTGTTAAAACCCGCACGCGTAATGAAAGTGAA
ATTAGGTGAGAGCCCTCACGGGCGCATCATCGACCGATCCTGATGTTCTCGGATGGATTT
GAGTAAGAGTTTTAACGGACGGACCCGAAAGACAGTGAACTATGCTTGTATAGGGTGAA
GCCAGAGGAAACTCTGGTGGAGGCTCGCAGCGGTTCTGACGTGCAAATCGATCGTCAAA
TATGAGCATGGGGGCGAAAGACTAATCGAACCACACCGCCCGTCGCTACTACCGATTGA
ATGGTTCAGTGAGGCGTTCGGACTGGCCCAGGGAGGTCGGCAACGACCACCCAGGGCC
GGAAAGTTCTCCAAACTCGATCATTTAGAGGAAGTAAAAGTCGTAACAAGGTCTCCGTT
GGTGAACCAGCGGAGGGATCATTGATGGAAAACACATTCCTATACCCCTTTGTGAACTT
ATACCTATTCTCGTTGCCTCGGCGCCAGGCCTGGGGTTCCCCCTCGGGGGTGCCCCCCAC
CCAAGGGACCTTACGGTCCCGGGGGCGGAGCAGGCCCGCCGGTGGCCCACCTAAACTCT
TGTTTTAGAACCTGTCTCCTCTGAGTTTGTTACCTAGAAAATGAATCAAAACTTTCAACA
ACGGATCTCTTGGCTCTGGCATCGATGAAGAACGCAGCGAAATGCGATAAGTAATGTGA
ATTGCAGAATTCAGTGAATCATCGAATCTTTGAACGCACATTGCGCCCGCTGGAATTCCG
GCGGGCATGCCTGTTCGAGCGTCATTTCAACCCCTCAAGCTTCAGCGCTTGGTGTTGGGG

CATAGCCTGTGAAAAGGCTAGCCCTCAAATTCAGTGGCGTGCTCGCTGAGACCCCCGGG
TGTAGTAATCTCCTTCTCGCCCGGGACGGCTCAGCGGTGTACCTGCCGTAAAACCCGCTA
TCACGCACCTTCTGAAAGTTGACCTCGGATCAGGTAGGAATACCCGCTGAACTTAAGCA
TATCAATAAGCGGAGGAAAAGAAACCAACAGGGATTGCCCTAGTAACGGCGAGTGAAG
CGGCAACAGCTCAAATTTGAAATCTGGTCCCCCCTGGGGTCCCGAGTTGTAATTTGCAG
AGGATGTCTTCTGGCGCGGTGCCTTCCGAGTTCCCTGGAACGGGACGCCACAGAGGGTG
AGAGCCCCGTAATGGTCGGATACCAAGCCTGTGTGAAGCTCCTTCGACGAGTCGAGTAG
TTTGGGAATGCTGCTCTAAATGGGAGGTAAATCTCTTCTAAAGCTAAATACTGGCCAGA
GACCGATAGCGCACAAGTAGAGTGATCGAAAGATGAAAAGCACCTTGAAAAGGGGGTT
AAACAGTACGTGAAATTGTTGAAAGGGAAGCGCTCATGACCAGACTTGCGCCGGGCGG
CTCAGCAGGGGTTCTGCCCCTGTGTACTCCGTCCCGGTTCAGGCCAGCATCGGTTCTCGC
TGGGGGATAAGAGCGGTGGGAACGTGGCCCCCCTTCGGGGGGGTGTTATAGCCCGCCGT
TATGATACCCTGGCGGGGACCGAGGTTCGCGCATTCGCAAGGATGCTGGCGTAATGGTT
ATCAGTGACCCGTCTTGAAACACGGACCAAGGAGTCGTCCTTTAGAGCGAGCGTCTGGG
TGTTAAAACCCGCACGCGTAATGAAAGTGAAATTAGGTGAGAGCCCTCACGGGCGCATC
ATCGACCGATCCTGATGTTCTCGGATGGATTTGAGTAAGAGTTTTAACGGACGGACCCG
AAAGACAGTGAACTATGCTTGTATAGGGTGAAGCCAGAGGAAACTCTGGTGGAGGCTC
GCAGCGGTTCTGACGTGCAAATCGATCGTCAAATATGAGCATGGGGGCGAAAGACTAAT
CGAACGGTCCGCTCCTGGCAAAGCTGTTCCGAAACATCGTCAAGAAGCTGACCGGCGAC
ATGATGGCGTACATGAAGCGTTGCGTCAGCGAGGGCAAGCATTTCGATCTGGCGTTGGG
CATCCGCCACTCAACCCTCACAAATGGCCTCAAGTACTCTCTCGCGACAGGAAACTGGG
GCGAGCAGAAGAAGGCCATGAGTTCCACCGCTGGTGTCTCACAGGTGCTGAACCGGTAC
ACGTTCTCGTCTACCCTATCGCATTTGCGGCGCACGAACACGCCTATCGGTCGTGACGGC
AAGCTCGCCAAGCCTCGTCAGCTGCACAATACACACTGGGGTCTCGTCTGTCCTGCAGA
GACTCCCGAGGGACAGGCTTGTGGTCTCGTCAAGAACCTATCGTTAATGTGCTACGTTA
GTGTTGGTTCACCGGCTGAGCCTATCAAGGACTTCATGGTGCAGCGTAATATGGAAGTC
TTGGAGGAATACGAGCCCGGCTCCAGTCCTGACGCAACCAAGATCTTCATCAACGGCAC
GTGGGTTGGTGTGCACAATGAACCAGCACACTTGGTCACGCTTGTGCAGGAGCTCCGGC
GCAAATGCATCATTTCTCACGAAGTCTCCCTCGTTCGCGACATTCGCGACCGCGAGTTCA
AGATCTTCTCTGACGCTGGTCGTGTCATGCGGCCTCTCTTTGTTGTCAATCAAGAGGATA
ATCCCGAGACTGGAGCGCAGCAGGGTACTCTGGCTCTCACCAAGCACCACATTCGCCGT
CTCGAGGAAGATGCTCAATACCATCGCAAGAAGCATGATGACGACTATTTCGGCTGGGA
TGGTCTTCAGAACAGCGGTTGCATCGAGTACCTGGATGCCGAGGAAGAGGAGACAGCA
ATGATCTGCATGAGTCCTGAGGATCTTGAGGACTACCGTCAGCGCAAGGCTAGGGGCAA
GGATGCCAAGGATGACGAACCGGAGGACGATGGCAGGAGTCTGAACGCTCGTGTGAAG
ACGAAGATCAACCCCGACATCCACATGTACACCCACTGCGAGATCCATCCGAGCATGCT
CCTGGGAATATGCGCCAGCATTATTCCCTTCCCGGATCACAATCAGGTGTGTAAATTTCT
CGCAGTACTAATTTCCCTCCTGCTGACCACATATATCTAGTCACCTCGTCATCACCAAAC
CAACATCATCACATCACCATGGCGCAAACATCAAGATCACGCCAAAAATGGTCGCCATC
TCTTCGTCGCGGCCCTTTGTACTGCCCACCTGGCGGGTGCCCTTTTTTCGCCCACCGCAC
ATTTTCGCTTTGTGGTGCGGGGTTTTGCGTCTGGGTCTTATCTCAGATAAACGTGACCCA
CCCAACCGCCACCTCATTCATCCTCCGCCAACACCCCCCTTGCGTGTGCCTCTTTTTTCACC
CTCAGAAATTTGACAACGACAATGCTGACTCGGATTTTTTACTGCAGCTGCTGAGCTCGG
CAAGGGTTCCTTCAAGTACGCATGGGTCCTTGACAAGCTCAAGGCCGAGCGTGAGCGTG
GTATCACCATCGACATTGCTCTGTGGAAGTTCGAGACTCCCAAGTACTATGTCACCGTCA
TTGGTAAGCTCTACCCCCTGGTGTTGTCGCGCAGGCCTGTTTGTCCCGCCTCACTGCGCT
GTCGCGCCATGCGCGACACCGCCGCTTTTGAAGCTTTTGCGCAACCCGTGCTAACGCAA

ATGTCACCACAGACGCTCCCGGTCACCGTGACTTCATCAAGAACATGATCACTGGTACC
TCCCAGGCCGACTGCGCCATTCTCATCATTGCCTCCGGTACTGGTGAGTTTGAGGCTGGT
ATCTCCAAGGATGGCCAGACCCGTGAGCACGCTCTGCTCGCCTACACCCTCGGTGTCAA
GCAGCTCATTGTTGCTTGCAACAAGATGGATACCGCTGGCTGGAAGCAGGACCGTTTTG
AGGAGATCAAGAAGGAGACCACCAACTTCATCAAGAAGGTCGGCTTCAACCCCAAGCA
GGTCGCTTTCGTCCCCATCTCCGGCTTCCACGGCGACAACATGCTTGAGGCCTCCTCCAA
CATGCCCTGGTACAAGGGCTGGACCAAGGAGACCCAGGACAAGAAGGAGGCCAAGGGT
ATGACCCTGCTCGACGCCATTGACTCCATCGAGCCCCCCAAGCGCCCCACCGAGAAGCC
CCTGCGTCTGCCCCTGCAGGACGTCTACAAGATCGGCGGTATTGGAACCGTACCCGTCG
GCCGTATCGAGACTGGTATCCTGAAGCCCGGTATGGTCGTCACCTTCGCCCCCTCCAACG
TCACCACTGAGGTCAAGTCCGTGGAGATGCACCACGAGCAGCTCACCGAGGGCCAGCCC
GGTGACAACGTTGGTTTCAACGTCAAGAACGTCTCCGTCAAGGAGATTCGTCGTGGCAA
CGTCGCTGGTGACTCCAAGAACGACCCCCCCTTGGGCGCCGCTTCCTTCAACGCCCAGGT
CATCGTTCTCAACCACCCCGGCCAGGTCGGTGCTGGATAC

>Melanconiella_flavovirens

CACACCGCCCGTCGCTACTACCGATTGAATGGTTCAGTGAGGCGTTCGGACTGGCCCAG
GGAGGTCGGCAACGACCACCCAGGGCCGGAAAGTTCTCCAAACTCGATCATTTAGAGGA
AGTAAAAGTCGTAACAAGGTCTCCGTTGGTGAACCAGCGGAGGGATCATTGATGGAAA
ACACATTCCTATACCCCTTTGTGAACTTATACCTATTCTCGTTGCCTCGGCGCCAGGCCT
GGGGTTCCCCCTCGGGGGTGCCCCCCACCCAAGGGACCTTACGGTCCCGGGGGCGGAGC
AGGCCCGCCGGTGGCCCACCTAAACTCTTGTTTTAGAACCTGTCTCCTCTGAGTTTGTTA
CCTAGAAAATGAATCAAAACTTTCAACAACGGATCTCTTGGCTCTGGCATCGATGAAGA
ACGCAGCGAAATGCGATAAGTAATGTGAATTGCAGAATTCAGTGAATCATCGAATCTTT
GAACGCACATTGCGCCCGCTGGAATTCCGGCGGGCATGCCTGTTCGAGCGTCATTTCAA
CCCCTCAAGCTTCAGCGCTTGGTGTTGGGGCATAGCCTGTGAAAAGGCTAGCCCTCAAA
TTCAGTGGCGTGCTCGCTGAGACCCCCGGGTGTAGTAATCTCCTTCTCGCCCGGGACGGC
TCAGCGGTGTACCTGCCGTAAAACCCGCTATCACGCACCTTCTGAAAGTTGACCTCGGAT
CAGGTAGGAATACCCGCTGAACTTAAGCATATCAATAAGCGGAGGAAAAGAAACCAAC
AGGGATTGCCCTAGTAACGGCGAGTGAAGCGGCAACAGCTCAAATTTGAAATCTGGTCC
CCCCTGGGGTCCCGAGTTGTAATTTGCAGAGGATGTCTTCTGGCGCGGTGCCTTCCGAGT
TCCCTGGAACGGGACGCCACAGAGGGTGAGAGCCCCGTAATGGTCGGATACCAAGCCT
GTGTGAAGCTCCTTCGACGAGTCGAGTAGTTTGGGAATGCTGCTCTAAATGGGAGGTAA
ATCTCTTCTAAAGCTAAATACTGGCCAGAGACCGATAGCGCACAAGTAGAGTGATCGAA
AGATGAAAAGCACCTTGAAAAGGGGGTTAAACAGTACGTGAAATTGTTGAAAGGGAAG
CGCTCATGACCAGACTTGCGCCGGGCGGCTCAGCAGGGGTTCTGCCCCTGTGTACTCCGT
CCCGGTTCAGGCCAGCATCGGTTCTCGCTGGGGGATAAGAGCGGTGGGAACGTGGCCCC
CCTTCGGGGGGGTGTTATAGCCCGCCGTTATGATACCCTGGCGGGGACCGAGGTTCGCG
CATTCGCAAGGATGCTGGCGTAATGGTTATCAGTGACCCGTCTTGAAACACGGACCAAG
GAGTCGTCCTTTAGAGCGAGCGTCTGGGTGTTAAAACCCGCACGCGTAATGAAAGTGAA
ATTAGGTGAGAGCCCTCACGGGCGCATCATCGACCGATCCTGATGTTCTCGGATGGATTT
GAGTAAGAGTTTTAACGGACGGACCCGAAAGACAGTGAACTATGCTTGTATAGGGTGAA
GCCAGAGGAAACTCTGGTGGAGGCTCGCAGCGGTTCTGACGTGCAAATCGATCGTCAAA
TATGAGCATGGGGGCGAAAGACTAATCGAACCACACCGCCCGTCGCTACTACCGATTGA
ATGGTTCAGTGAGGCGTTCGGACTGGCCCAGGGAGGTCGGCAACGACCACCCAGGGCC
GGAAAGTTCTCCAAACTCGATCATTTAGAGGAAGTAAAAGTCGTAACAAGGTCTCCGTT
GGTGAACCAGCGGAGGGATCATTGATGGAAAACACATTCCTATACCCCTTTGTGAACTT

ATACCTATTCTCGTTGCCTCGGCGCCAGGCCTGGGGTTCCCCCTCGGGGGTGCCCCCCAC
CCAAGGGACCTTACGGTCCCGGGGGCGGAGCAGGCCCGCCGGTGGCCCACCTAAACTCT
TGTTTTAGAACCTGTCTCCTCTGAGTTTGTTACCTAGAAAATGAATCAAAACTTTCAACA
ACGGATCTCTTGGCTCTGGCATCGATGAAGAACGCAGCGAAATGCGATAAGTAATGTGA
ATTGCAGAATTCAGTGAATCATCGAATCTTTGAACGCACATTGCGCCCGCTGGAATTCCG
GCGGGCATGCCTGTTCGAGCGTCATTTCAACCCCTCAAGCTTCAGCGCTTGGTGTTGGGG
CATAGCCTGTGAAAAGGCTAGCCCTCAAATTCAGTGGCGTGCTCGCTGAGACCCCCGGG
TGTAGTAATCTCCTTCTCGCCCGGGACGGCTCAGCGGTGTACCTGCCGTAAAACCCGCTA
TCACGCACCTTCTGAAAGTTGACCTCGGATCAGGTAGGAATACCCGCTGAACTTAAGCA
TATCAATAAGCGGAGGAAAAGAAACCAACAGGGATTGCCCTAGTAACGGCGAGTGAAG
CGGCAACAGCTCAAATTTGAAATCTGGTCCCCCCTGGGGTCCCGAGTTGTAATTTGCAG
AGGATGTCTTCTGGCGCGGTGCCTTCCGAGTTCCCTGGAACGGGACGCCACAGAGGGTG
AGAGCCCCGTAATGGTCGGATACCAAGCCTGTGTGAAGCTCCTTCGACGAGTCGAGTAG
TTTGGGAATGCTGCTCTAAATGGGAGGTAAATCTCTTCTAAAGCTAAATACTGGCCAGA
GACCGATAGCGCACAAGTAGAGTGATCGAAAGATGAAAAGCACCTTGAAAAGGGGGTT
AAACAGTACGTGAAATTGTTGAAAGGGAAGCGCTCATGACCAGACTTGCGCCGGGCGG
CTCAGCAGGGGTTCTGCCCCTGTGTACTCCGTCCCGGTTCAGGCCAGCATCGGTTCTCGC
TGGGGGATAAGAGCGGTGGGAACGTGGCCCCCCTTCGGGGGGGTGTTATAGCCCGCCGT
TATGATACCCTGGCGGGGACCGAGGTTCGCGCATTCGCAAGGATGCTGGCGTAATGGTT
ATCAGTGACCCGTCTTGAAACACGGACCAAGGAGTCGTCCTTTAGAGCGAGCGTCTGGG
TGTTAAAACCCGCACGCGTAATGAAAGTGAAATTAGGTGAGAGCCCTCACGGGCGCATC
ATCGACCGATCCTGATGTTCTCGGATGGATTTGAGTAAGAGTTTTAACGGACGGACCCG
AAAGACAGTGAACTATGCTTGTATAGGGTGAAGCCAGAGGAAACTCTGGTGGAGGCTC
GCAGCGGTTCTGACGTGCAAATCGATCGTCAAATATGAGCATGGGGGCGAAAGACTAAT
CGAACGGTCCGCTCCTGGCAAAGCTGTTCCGAAACATCGTCAAGAAGCTGACCGGCGAC
ATGATGGCGTACATGAAGCGTTGCGTCAGCGAGGGCAAGCATTTCGATCTGGCGTTGGG
CATCCGCCACTCAACCCTCACAAATGGCCTCAAGTACTCTCTCGCGACAGGAAACTGGG
GCGAGCAGAAGAAGGCCATGAGTTCCACCGCTGGTGTCTCACAGGTGCTGAACCGGTAC
ACGTTCTCGTCTACCCTATCGCATTTGCGGCGCACGAACACGCCTATCGGTCGTGACGGC
AAGCTCGCCAAGCCTCGTCAGCTGCACAATACACACTGGGGTCTCGTCTGTCCTGCAGA
GACTCCCGAGGGACAGGCTTGTGGTCTCGTCAAGAACCTATCGTTAATGTGCTACGTTA
GTGTTGGTTCACCGGCTGAGCCTATCAAGGACTTCATGGTGCAGCGTAATATGGAAGTC
TTGGAGGAATACGAGCCCGGCTCCAGTCCTGACGCAACCAAGATCTTCATCAACGGCAC
ATGGGTTGGTGTGCACAATGAACCAGCACACTTGGTCACGCTTGTGCAGGAGCTCCGGC
GCAAATGCATCATTTCTCACGAAGTCTCCCTCGTTCGCGACATTCGCGACCGCGAGTTCA
AGATCTTCTCTGACGCTGGTCGTGTCATGCGGCCTCTCTTTGTTGTCAATCAAGAGGATA
ATCCCGAGACTGGAGCGCAGCAGGGTACTCTGGCTCTCACCAAGCACCACATTCGCCGT
CTCGAGGAAGATGCTCAATACCATCGCAAGAAGCATGATGACGACTATTTCGGCTGGGA
TGGTCTTCAGAACAGCGGTTGCATCGAGTACCTGGATGCCGAGGAAGAGGAGACAGCA
ATGATCTGCATGAGTCCTGAGGATCTTGAGGACTACCGTCAGCGCAAGGCTAGGGGCAA
GGATGCCAAGGATGACGAACCGGAGGACGATGGCAGGAGTCTGAACGCTCGTGTGAAG
ACGAAGATCAACCCCGACATCCACATGTACACCCACTGCGAGATCCATCCGAGCATGCT
CCTGGGAATATGCGCCAGCATTATTCCCTTCCCGGATCACAATCAGGTGTGTAAATTTCT
CGCAGTACTAATTTCCCTCCTGCTGACCACATATATCTAGTCACCTCGTCATCACCAAAC
CAACATCATCACATCACCATGGCGCAAACATCAAGATCACGCCAAAAATGGTCGCCATC
TCTTCGTCGCGGCCCTTTGTACTGCCCACCTGGCGGGTGCCCTTTTTTCGCCCACCGCAC
ATTTTCGCTTTGTGGTGCGGGGTTTTGCGTCTGGGTCTTATCTCAGATAAACGTGACCCA

CCCAACCGCCACCTCATTCATCCTCCGCCAACACCCCCTTGCGTGTGCCTCTTTTTCACC
CTCAGAAATTTGACAACGACAATGCTGACTCGGATTTTTACTGCAGCTGCTGAGCTCGG
CAAGGGTTCCTTCAAGTACGCATGGGTCCTTGACAAGCTCAAGGCCGAGCGTGAGCGTG
GTATCACCATCGACATTGCTCTGTGGAAGTTCGAGACTCCCAAGTACTATGTCACCGTCA
TTGGTAAGCTCTACCCCCTGGTGTTGTCGCGCAGGCCTGTTTGTCCCGCCTCACTGCGCT
GTCGCGCCATGCGCGACACCGCCGCTTTTGAAGCTTTTGCGCAACCCGTGCTAACGCAA
ATGTCACCACAGACGCTCCCGGTCACCGTGACTTCATCAAGAACATGATCACTGGTACC
TCCCAGGCCGACTGCGCCATTCTCATCATTGCCTCCGGTACTGGTGAGTTTGAGGCTGGT
ATCTCCAAGGATGGCCAGACCCGTGAGCACGCTCTGCTCGCCTACACCCTCGGTGTCAA
GCAGCTCATTGTTGCTTGCAACAAGATGGATACCGCTGGCTGGAAGCAGGACCGTTTTG
AGGAGATCAAGAAGGAGACCACCAACTTCATCAAGAAGGTCGGCTTCAACCCCAAGCA
GGTCGCTTTCGTCCCCATCTCCGGCTTCCACGGCGACAACATGCTTGAGGCCTCCTCCAA
CATGCCCTGGTACAAGGGCTGGACCAAGGAGACCCAGGACAAGAAGGAGGCCAAGGGT
ATGACCCTGCTCGACGCCATTGACTCCATCGAGCCCCCCAAGCGCCCCACCGAGAAGCC
CCTGCGTCTGCCCCTGCAGGACGTCTACAAGATCGGCGGTATTGGAACCGTACCCGTCG
GCCGTATCGAGACTGGTATCCTGAAGCCCGGTATGGTCGTCACCTTCGCCCCCTCCAACG
TCACCACTGAGGTCAAGTCCGTGGAGATGCACCACGAGCAGCTCACCGAGGGCCAGCCC
GGTGACAACGTTGGTTTCAACGTCAAGAACGTCTCCGTCAAGGAGATTCGTCGTGGCAA
CGTCGCTGGTGACTCCAAGAACGACCCCCCCTTGGGCGCCGCTTCCTTCAACGCCCAGGT
CATCGTTCTCAACCACCCCGGCCAGGTCGGTGCTGGATAC

>Melanconiella_betulicola

CATTGCTGGAACACGTCCCCCAGGGCGTTCCCAGATACCCTTTGTGAACTTATACCTATT
TCGTTGCCTCGGCGCTGGCTGGGGGCCCTTCTATGGGGCTCCCCCACGGTCTCCGTGGAG
CAGGCCCGCCGGCGGCCCTACAAACTCTTGTTTTTATAACGTATCTCTTCTGAGTAACAA
ACTTACAAATGAATCAAAACTTTCAACAACGGATCTCTTGGTTCTGGCATCGATGAAGA
ACGCAGCGAAATGCGATAAGTAATGTGAATTGCAGAATTCAGTGAATCATCGAATCTTT
GAACGCACATTGCGCCCGCTGGTATTCCGGCGGGCATGCCTGTTCGAGCGTCATTTCAAC
CCTCAAGCTTTACCGCTTGGTGTTGGGGCGTAGCCTGTAAAAAGGCTAGCCCTGAAATTC
AGTGGCGTGCTCGCTAAGACTCCGAGCGTAGTAATTTTTTTCTCGCTTAGGTTGGATTAG
CGGTGCTCAGCCGTAAAACCTCGGCCCGAGTTGTAATTTGCAGAGGATGCTTCTGGCGC
GGTGCCTTCCGAGTTCCCTGGAACGGGACGCCACAGAGGGTGAGAGCCCCGTATGGTCG
GACACCAAGCCTGTGTGAAGCTCCTTCGACGAGTCGAGTAGTTTGGGAATGCTGCTCAA
AATGGGAGGTAAATCTCTTCTAAAGCTAAATACCGGCCAGAGACCGATAGCGCACAAGT
AGAGTGATCGAAAGATGAAAAGCACCTTGAAAAGGGGGTTAAACAGTACGTGAAATTG
TTGAAAGGGAAGCGCTTGTGACCAGACTTGTGCCGGGCGGCTCATCAGGGGTTCTCCCC
TGTGTACTCCGCCCGGTTCAGGCCAGCATCGGTTTTCGCTGGGGGATAAGAACGGTGGG
AACGTGGCCCCCCCTCGGGGGGGTGTTATAGCCCGCCGTACGATACCCTAGCGGGGACC
GAGGTTCGCGCTCCGCAAGGATGCTGGCGTAATGGTCATCAGCGACCCGTCTTACGGGT
GATATGATGGCGTACATGAAGCGTTGTGTCAGCGAGGGCAAGCATTTCGACCTAGCGCT
GGGCATTCGCCATTCCACCCTCACAAATGGTTTGAAGTACTCTCTTGCGACTGGAAACTG
GGGTGAGCAGAAGAAGGCAATGAGTTCTACCGCTGGCGTCTCGCAGGTGCTCAACCGAT
ACACGTTTTCGTCTACCCTATCGCATTTGCGCCGCACGAATACGCCCATTGGCCGTGACG
GCAAGCTAGCCAAGCCTCGTCAGCTTCACAACACTCACTGGGGCCTCGTCTGTCCAGCA
GAGACACCTGAGGGACAAGCTTGCGGTCTCGTCAAGAACCTGTCTTTAATGTGCTACGT
CAGTGTTGGCTCACCAGCCGAGCCTATCAAGGACTTCATGGTGCAGCGAAATATGGAAG
TTTTGGAGGAATACGAGCCGGGCTCCAGCCCCGACGCGACCAAGATCTTCATCAACGGC

ACGTGGGTTGGTGTCCACAACGAACCAGCACACTTAGTCACGCTTGTGCAAGAGCTTCG
ACGCAAATGCATCATTTCTCACGAGGTCTCCCTCGTTCGCGATATTCGCGATCGCGAGTT
CAAGATCTTCTCCGACGCTGGTCGTGTGATGCGGCCTCTTTTCGTCGTCAACCAAGAGGA
CAACCCCGAGACTGGAGCGCAGCAGGGCACTCTGGCTCTCACCAAGCATCACATTCGCC
GTCTCGAGGAAGATGCTCAGTATCAGCGCAAGAAGCATGACGAAGACTATTTTGGCTGG
GATGGCCTTCAGAACAGCGGCTGCATTGAGTACCTGGATGCCGAGGAAGAGGAGACAG
CAATGATCTGCATGAGTCCTGAGGATCTCGAGGACTACCGGCAGCGCAAGGCCAGCGGC
AAGGATGCCAAGCAGGAAGAGCCTGAGGATGATGGCAGGAGTTTGAACGCTCGTGTGA
AGACGAAGATCAATACCGACATCCACATGTACACCCACTGCGAAATCCATCCGAGCATG
CTCTTGGGAATCTGCGCCAGCCGTCATCATCACAATCGCACAAACAAACTCCAAAGCAA
ACGGTCGCGATTTCCTTGCCCGGCCGCTTTCTTAGTTGGCTGTGTGTCCTTTTTTTGCCCG
TCGCATATTTTCGCTTTGGTGCGGGGTTTTGCATCTGGGTCTTATCTCAGATAAACGTGA
CCCACCGACCCGCCACCCCAATCACTCTGCCAACACACTCTTGCGCACCACTCACCGTCC
CACCATGACAACGGCGATGCTAACTCAATTCTTTCAACAGCTGCTGAACTCGGCAAGGG
TTCCTTCAAGTACGCATGGGTCCTTGACAAGCTCAAGGCCGAGCGTGAGCGTGGTATCA
CCATCGACATTGCTCTGTGGAAGTTCGAGACTCCCAAGTACTATGTCACCGTCATTGGTA
AGTTTGTCCGTTTTGACGATATCGCGCAGACCTGGCTACCCCGCCTACGCACTGCTCCAC
CAGGGGCACCATGGTGGCTCCTTACACTTGTGCGCAACCCGTGCTAACGCAAGTGTCAC
CACAGACGCTCCCGGTCACCGTGACTTCATCAAGAACATGATCACTGGTACCTCCCAGG
CTGACTGCGCCGTTCTCATCATTGCCTCTGGTACT

>Melanconiella_hyperopta

CACACCGCCCGTCGCTACTACCGATTGAATGGTTCAGTGAGGCGTCCGGACTGGCCCCA
GGAGGTCGGCAACGACCACCCAGGGCCGGAAAGTTCTCCAAACTCGATCATTTAGAGGA
AGTAAAAGTCGTAACAAGGTCTCCGTTGGTGAACCAGCGGAGGGATCATTGATGGAACT
ACGTCCCATATTACCCCTGTGAACTTATACCTATATCGTTGCCTCGGCGTAGGCCCGGGG
GCCCTCTCCCCAGAGGGTCCCCCCACCGGTGGGAGCCCCACGGCTTCCCCCCTGTGGAG
CAGGCCCGTCGGCGGCCCCTATAAACCATTACTTGTAAAGAAGACCTTCTGAGTACAAA
CTTAAAATGAATCAAAACTTTCAACAACGGATCTCTTGGTTCTGGCATCGATGAAGAAC
GCAGCGAAATGCGATACGTAATGCGAATTGCAGAATTCAGTGAGTCATCGAATCTTTGA
ACGCACATTGCGCCCGCTGGACATTCCGGCGGGCATGCCTGTTCGAGCGTCATTTCACCC
CTCAGGCTTCAGCGCCTGGTGTTGGGGCATAGCCTGTCAAAGGGCTAGCCCTCAAATTC
AGTGGCGGAACCGCTGGGACTCCGGGCGTAGTAATTTTTTCTCGCTTAGGTGGTCTCCAG
TGGTGCCCAGCCGTAAAACCCGCTATCATGCACCCCCCCTAGAGATTGACCTCGGATCA
GGTAGGAATACCCGCTGAACTTAAGCATATCAATAAGCGGAGGAAAAGAAACCAACAG
GGATTGCCCTAGTAACGGCGAGTGAAGCGGCAACAGCTCAAATTTGAAATCTGGCCCCC
CCGGGCCCGAGTTGTAATTTGCAGAGGATGTTCTCTGGCGCGGTGCCTTCCGAGTTCCCT
GGAATGGGACGCCACAGAGGGTGAGAGCCCCGTACGGTCGGACTAACCAAGCCTGTGT
GAGACTCCTTCGACGAGTCGAGTAGTTTGGGAATGCTGCTCTAAATGGGAGGTAAATCT
CTTCTAAAGCTAAATACTGGCCAGAGACCGATAGCGCACAAGTAGAGTGATCGAAAGAT
GAAAAGCACCTTGAAAAGGGGGTTAAACAGTACGTGAAATTGTTGAAAGGGAAGCGCT
CCTGACCAGAAACTTGGGCCGGGCGGCTCAGCAGGGGCTCGCCCCCTGTGTACTCCGTC
CCGGTTCCGGGCCAGCATCGGTTCTCGCTGGGGGACAAGAACGGTGGGAACGTGGCCCC
CCCTCGGGGGGGTGTTATAGCCCGCCGTAACGATACCCTGGTGGGGACCGAGGATTCCG
CGCTTCGGCAAGGATGCTGGCATAATGGTCATCAGCGACCCGTCTTGAAACACGGACCA
AGGAGTCGTCCATTAGAGCGAGCGTTTGGGTGTCAAAACCCGTAGCGCGTAATGAAAGT

GAAATTCTAGGTGAGAACCTCCTCGCGGGGGCGCATCATCGACCGATCCTGATGTTCTC
GGACGGATTTGAGTAAGAGTTTTAACGGACGGACCCGAAAGACAGTGAACTATGCTTGT
ATAGGGTGAAGCCAGAGGAAACTCTGGTGGAGGCTCGCAGCGGTTCTGACGTGCAAATC
GATCGTCAAATATGAGCATGGGGGCGAAAGACTAATCGAACCACACCGCCCGTCGCTAC
TACCGATTGAATGGTTCAGTGAGGCGTCCGGACTGGCCCCAGGAGGTCGGCAACGACCA
CCCAGGGCCGGAAAGTTCTCCAAACTCGATCATTTAGAGGAAGTAAAAGTCGTAACAAG
GTCTCCGTTGGTGAACCAGCGGAGGGATCATTGATGGAACTACGTCCCATATTACCCCT
GTGAACTTATACCTATATCGTTGCCTCGGCGTAGGCCCGGGGGCCCTCTCCCCAGAGGGT
CCCCCCACCGGTGGGAGCCCCACGGCTTCCCCCCTGTGGAGCAGGCCCGTCGGCGGCCC
CTATAAACCATTACTTGTAAAGAAGACCTTCTGAGTACAAACTTAAAATGAATCAAAAC
TTTCAACAACGGATCTCTTGGTTCTGGCATCGATGAAGAACGCAGCGAAATGCGATACG
TAATGCGAATTGCAGAATTCAGTGAGTCATCGAATCTTTGAACGCACATTGCGCCCGCT
GGACATTCCGGCGGGCATGCCTGTTCGAGCGTCATTTCACCCCTCAGGCTTCAGCGCCTG
GTGTTGGGGCATAGCCTGTCAAAGGGCTAGCCCTCAAATTCAGTGGCGGAACCGCTGGG
ACTCCGGGCGTAGTAATTTTTTCTCGCTTAGGTGGTCTCCAGTGGTGCCCAGCCGTAAAA
CCCGCTATCATGCACCCCCCCTAGAGATTGACCTCGGATCAGGTAGGAATACCCGCTGA
ACTTAAGCATATCAATAAGCGGAGGAAAAGAAACCAACAGGGATTGCCCTAGTAACGG
CGAGTGAAGCGGCAACAGCTCAAATTTGAAATCTGGCCCCCCCGGGCCCGAGTTGTAAT
TTGCAGAGGATGTTCTCTGGCGCGGTGCCTTCCGAGTTCCCTGGAATGGGACGCCACAG
AGGGTGAGAGCCCCGTACGGTCGGACTAACCAAGCCTGTGTGAGACTCCTTCGACGAGT
CGAGTAGTTTGGGAATGCTGCTCTAAATGGGAGGTAAATCTCTTCTAAAGCTAAATACT
GGCCAGAGACCGATAGCGCACAAGTAGAGTGATCGAAAGATGAAAAGCACCTTGAAAA
GGGGGTTAAACAGTACGTGAAATTGTTGAAAGGGAAGCGCTCCTGACCAGAAACTTGG
GCCGGGCGGCTCAGCAGGGGCTCGCCCCCTGTGTACTCCGTCCCGGTTCCGGGCCAGCA
TCGGTTCTCGCTGGGGGACAAGAACGGTGGGAACGTGGCCCCCCCTCGGGGGGGTGTTA
TAGCCCGCCGTAACGATACCCTGGTGGGGACCGAGGATTCCGCGCTTCGGCAAGGATGC
TGGCATAATGGTCATCAGCGACCCGTCTTGAAACACGGACCAAGGAGTCGTCCATTAGA
GCGAGCGTTTGGGTGTCAAAACCCGTAGCGCGTAATGAAAGTGAAATTCTAGGTGAGAA
CCTCCTCGCGGGGGCGCATCATCGACCGATCCTGATGTTCTCGGACGGATTTGAGTAAG
AGTTTTAACGGACGGACCCGAAAGACAGTGAACTATGCTTGTATAGGGTGAAGCCAGAG
GAAACTCTGGTGGAGGCTCGCAGCGGTTCTGACGTGCAAATCGATCGTCAAATATGAGC
ATGGGGGCGAAAGACTAATCGAACGGTCCGCTCCTGGCAAAGCTGTTCCGAAACATTGT
CAAGAAGCTGACCGGTGACATGATGGCGTACATGAAACGTTGTGTCAGCGAGGGCAAG
CATTTCGATTTGGCGTTGGGCATCCGCCACTCAACCCTCACAAATGGCCTCAAGTACTCT
CTTGCGACTGGAAACTGGGGCGAGCAGAAGAAGGCGATGAGTTCCACCGCTGGTGTCTC
TCAGGTGCTAAACCGGTACACGTTCTCGTCCACTCTATCGCATTTGCGGCGCACGAACAC
TCCCATTGGTCGCGACGGCAAGCTGGCCAAGCCTCGTCAGCTGCACAACACTCACTGGG
GCCTCGTCTGTCCGGCAGAGACTCCTGAGGGACAGGCTTGCGGTCTCGTCAAGAACCTG
TCTTTGATGTGCTACGTCAGCGTCGGTTCACCAGCTGAGCCTATCAAGGACTTCATGGTG
CAACGAAATATGGAAGTCTTGGAGGAATACGAGCCCGGGTCCAGCCCTGACGCAACCA
AGATCTTCATCAACGGCACGTGGGTTGGTGTCCACAATGAACCAGCGCACTTGGTCACG
CTTGTGCAAGAGCTTCGGCGCAAATGCATCATTTCTCACGAAGTCTCCCTTGTTCGTGAC
ATTCGCGATCGCGAGTTCAAGATTTTCTCCGACGCTGGTCGTGTGATGCGCCCTCTCTTT
GTCGTTAATCAAGACGACAATCCCGAGACTGGAGCGCAGCAGGGCACTCTGGCTCTTAC
CAAGCACCACATTCGCCGTCTCGAGGAAGATGCTCAGTACCATCGCAAGAAGCATGATG
AGGACTATTTCGGCTGGGACGGTCTTCAGAACAGCGGTTGCATCGAGTACCTGGATGCC
GAGGAAGAGGAGACAGCAATGATTTGCATGAGTCCTGAGGATCTCGAGGACTACCGCC
AGCGCAAGGCTAGGGGCAAGGATGCCAAGGATGATGAACCTGAGGATGATGGCAGGAG

TCTGAACGCTCGTGTGAAGACGAAGATCAACCCCGACATCCACATGTACACTCACTGCG
AGATCCATCCAAGCATGCTCCTGGGAATCTGCGCCAGCATCATTCCCTTCCCGGATCACA
ACCAGGTATGTGAAACCTTCGCAGTATTAGCATCACTCCTGCTGACCACATATGTCTAGT
CACCCCGCTCAAATCATCACCATCACAATCGATGGCGCAGACATTTAGGCAAAAGCGGC
CGCGATCCTTTCGTCGCAGCCGTCTGTCCTGCCTAGCTGTGTCCTTTTTTGCCCACCACA
CATTTTCGCTTTGTGGTGCGGGGTTTAGCGTCTGGGTCTTATCTCAGAGATAAACGTGAC
CCACCCAACCGCCACCTCCATCAATTCGCCAACGCATCCATGCGCGCATCCCCCAACCTC
AAAATCTGACGAAGACAATGCTGACCCGTATTCTTCATTGCAGCTGCTGAGCTCGGCAA
GGGTTCCTTCAAGTACGCATGGGTCCTTGACAAGCTCAAGGCCGAGCGTGAGCGTGGTA
TCACCATCGACATTGCTCTGTGGAAGTTCGAGACTCCCAAGTACTATGTCACCGTCATTG
GTAAGTCCACCCTCCCTGGCGATGTCGCGCAGGCCTGGCTGCCTCGCCATGCGCGACTTG
GCGGCTTCTGATGCTTTTGCGGAATCCTGCTAATGCAAATATTACCACAGACGCTCCCGG
TCACCGTGACTTCATCAAGAACATGATCACTGGTACCTCCCAGGCTGACTGCGCCGTTCT
CATCATTGCCTCCGGTACTGGTGAGTTCGAGGCTGGTATCTCCAAGGATGGCCAGACCC
GTGAGCACGCTCTGCTCGCCTACACCCTCGGTGTCAAGCAGCTCATTGTTGCTTGCAACA
AGATGGACACTGCTGGCTGGAAGCAGGACCGTTTCGAGGAGATCAAGAAGGAGACCAC
GAACTTCATCAAGAAGGTCGGCTTCAACCCCAAGCAGGTCGCGTTCGTCCCCATCTCCG
GCTTCCACGGCGACAACATGCTTGAGGCCTCCGCCAACATGCCCTGGTACAAGGGCTGG
ACCAAGGAGACCCAGGACAAGAAGGAGGCCAAGGGTATGACCCTGCTCGACGCCATTG
ACGCCATCGAGCCCCCCAAGCGCCCCACCGAGAAGCCCCTTCGTCTGCCCCTCCAGGAC
GTCTACAAGATCGGCGGTATTGGAACTGTACCTGTCGGCCGTATCGAGACTGGTATCCT
GAAGCCCGGTATGGTCGTCACCTTCGCTCCTTCCAACGTCACCACTGAGGTCAAGTCCGT
CGAGATGCACCACGAGCAGCTCACCGAGGGTCAGCCCGGTGACAACGTTGGTTTCAACG
TCAAGAACGTCTCCGTCAAGGAGATTCGCCGTGGCAACGTCGCCGGTGACTCCAAGAAC
GACCCCCCCATGGCCGCCGCTTCCTTCAACGCCCAGGTCATCGTCCTGAACCACCCCGGC
CAGGTCGGTGCTGGATAC

>Melanconiella_hyperopta

CACACCGCCCGTCGCTACTACCGATTGAATGGTTCAGTGAGGCGTCCGGACTGGCCCCA
GGAGGTCGGCAACGACCACCCAGGGCCGGAAAGTTCTCCAAACTCGATCATTTAGAGGA
AGTAAAAGTCGTAACAAGGTCTCCGTTGGTGAACCAGCGGAGGGATCATTGATGGAACT
ACGTCCCATATTACCCCTGTGAACTTATACCTATATCGTTGCCTCGGCGTAGGCCCGGGG
GCCCTCTCCCCAGAGGGTCCCCCCACCGGTGGGAGCCCCACGGCTTCCCCCCTGTGGAG
CAGGCCCGTCGGCGGCCCCTATAAACCATTACTTGTAAAGAAGACCTTCTGAGTACAAA
CTTAAAATGAATCAAAACTTTCAACAACGGATCTCTTGGTTCTGGCATCGATGAAGAAC
GCAGCGAAATGCGATACGTAATGCGAATTGCAGAATTCAGTGAGTCATCGAATCTTTGA
ACGCACATTGCGCCCGCTGGACATTCCGGCGGGCATGCCTGTTCGAGCGTCATTTCACCC
CTCAGGCTTCAGCGCCTGGTGTTGGGGCATAGCCTGTCAAAGGGCTAGCCCTCAAATTC
AGTGGCGGAACCGCTGGGACTCCGGGCGTAGTAATTTTTTCTCGCTTAGGTGGTCTCCAG
TGGTGCCCAGCCGTAAAACCCGCTATCATGCACCCCCCCTAGAGATTGACCTCGGATCA
GGTAGGAATACCCGCTGAACTTAAGCATATCAATAAGCGGAGGAAAAGAAACCAACAG
GGATTGCCCTAGTAACGGCGAGTGAAGCGGCAACAGCTCAAATTTGAAATCTGGCCCCC
CCGGGCCCGAGTTGTAATTTGCAGAGGATGTTCTCTGGCGCGGTGCCTTCCGAGTTCCCT
GGAATGGGACGCCACAGAGGGTGAGAGCCCCGTACGGTCGGACTAACCAAGCCTGTGT
GAGACTCCTTCGACGAGTCGAGTAGTTTGGGAATGCTGCTCTAAATGGGAGGTAAATCT
CTTCTAAAGCTAAATACTGGCCAGAGACCGATAGCGCACAAGTAGAGTGATCGAAAGAT

GAAAAGCACCTTGAAAAGGGGGTTAAACAGTACGTGAAATTGTTGAAAGGGAAGCGCT
CCTGACCAGAAACTTGGGCCGGGCGGCTCAGCAGGGGCTCGCCCCCTGTGTACTCCGTC
CCGGTTCCGGGCCAGCATCGGTTCTCGCTGGGGGACAAGAACGGTGGGAACGTGGCCCC
CCCTCGGGGGGGTGTTATAGCCCGCCGTAACGATACCCTGGTGGGGACCGAGGATTCCG
CGCTTCGGCAAGGATGCTGGCATAATGGTCATCAGCGACCCGTCTTGAAACACGGACCA
AGGAGTCGTCCATTAGAGCGAGCGTTTGGGTGTCAAAACCCGTAGCGCGTAATGAAAGT
GAAATTCTAGGTGAGAACCTCCTCGCGGGGGCGCATCATCGACCGATCCTGATGTTCTC
GGACGGATTTGAGTAAGAGTTTTAACGGACGGACCCGAAAGACAGTGAACTATGCTTGT
ATAGGGTGAAGCCAGAGGAAACTCTGGTGGAGGCTCGCAGCGGTTCTGACGTGCAAATC
GATCGTCAAATATGAGCATGGGGGCGAAAGACTAATCGAACCACACCGCCCGTCGCTAC
TACCGATTGAATGGTTCAGTGAGGCGTCCGGACTGGCCCCAGGAGGTCGGCAACGACCA
CCCAGGGCCGGAAAGTTCTCCAAACTCGATCATTTAGAGGAAGTAAAAGTCGTAACAAG
GTCTCCGTTGGTGAACCAGCGGAGGGATCATTGATGGAACTACGTCCCATATTACCCCT
GTGAACTTATACCTATATCGTTGCCTCGGCGTAGGCCCGGGGGCCCTCTCCCCAGAGGGT
CCCCCCACCGGTGGGAGCCCCACGGCTTCCCCCCTGTGGAGCAGGCCCGTCGGCGGCCC
CTATAAACCATTACTTGTAAAGAAGACCTTCTGAGTACAAACTTAAAATGAATCAAAAC
TTTCAACAACGGATCTCTTGGTTCTGGCATCGATGAAGAACGCAGCGAAATGCGATACG
TAATGCGAATTGCAGAATTCAGTGAGTCATCGAATCTTTGAACGCACATTGCGCCCGCT
GGACATTCCGGCGGGCATGCCTGTTCGAGCGTCATTTCACCCCTCAGGCTTCAGCGCCTG
GTGTTGGGGCATAGCCTGTCAAAGGGCTAGCCCTCAAATTCAGTGGCGGAACCGCTGGG
ACTCCGGGCGTAGTAATTTTTTCTCGCTTAGGTGGTCTCCAGTGGTGCCCAGCCGTAAAA
CCCGCTATCATGCACCCCCCCTAGAGATTGACCTCGGATCAGGTAGGAATACCCGCTGA
ACTTAAGCATATCAATAAGCGGAGGAAAAGAAACCAACAGGGATTGCCCTAGTAACGG
CGAGTGAAGCGGCAACAGCTCAAATTTGAAATCTGGCCCCCCCGGGCCCGAGTTGTAAT
TTGCAGAGGATGTTCTCTGGCGCGGTGCCTTCCGAGTTCCCTGGAATGGGACGCCACAG
AGGGTGAGAGCCCCGTACGGTCGGACTAACCAAGCCTGTGTGAGACTCCTTCGACGAGT
CGAGTAGTTTGGGAATGCTGCTCTAAATGGGAGGTAAATCTCTTCTAAAGCTAAATACT
GGCCAGAGACCGATAGCGCACAAGTAGAGTGATCGAAAGATGAAAAGCACCTTGAAAA
GGGGGTTAAACAGTACGTGAAATTGTTGAAAGGGAAGCGCTCCTGACCAGAAACTTGG
GCCGGGCGGCTCAGCAGGGGCTCGCCCCCTGTGTACTCCGTCCCGGTTCCGGGCCAGCA
TCGGTTCTCGCTGGGGGACAAGAACGGTGGGAACGTGGCCCCCCCTCGGGGGGGTGTTA
TAGCCCGCCGTAACGATACCCTGGTGGGGACCGAGGATTCCGCGCTTCGGCAAGGATGC
TGGCATAATGGTCATCAGCGACCCGTCTTGAAACACGGACCAAGGAGTCGTCCATTAGA
GCGAGCGTTTGGGTGTCAAAACCCGTAGCGCGTAATGAAAGTGAAATTCTAGGTGAGAA
CCTCCTCGCGGGGGCGCATCATCGACCGATCCTGATGTTCTCGGACGGATTTGAGTAAG
AGTTTTAACGGACGGACCCGAAAGACAGTGAACTATGCTTGTATAGGGTGAAGCCAGAG
GAAACTCTGGTGGAGGCTCGCAGCGGTTCTGACGTGCAAATCGATCGTCAAATATGAGC
ATGGGGGCGAAAGACTAATCGAACGGTCCGCTCCTGGCAAAGCTGTTCCGAAACATTGT
CAAGAAGCTGACCGGTGACATGATGGCGTACATGAAACGTTGTGTCAGCGAGGGCAAG
CATTTCGATTTGGCGTTGGGCATCCGCCACTCAACCCTCACAAATGGCCTCAAGTACTCT
CTTGCGACTGGAAACTGGGGCGAGCAGAAGAAGGCGATGAGTTCCACCGCTGGTGTCTC
TCAGGTGCTAAACCGGTACACGTTCTCGTCCACTCTATCGCATTTGCGGCGCACGAACAC
TCCCATTGGTCGCGACGGCAAGCTGGCCAAGCCTCGTCAGCTGCACAACACTCACTGGG
GCCTCGTCTGTCCGGCAGAGACTCCTGAGGGACAGGCTTGCGGTCTCGTCAAGAACCTG
TCTTTGATGTGCTACGTCAGCGTCGGTTCACCAGCTGAGCCTATCAAGGACTTCATGGTG
CAACGAAATATGGAAGTCTTGGAGGAATACGAGCCCGGGTCCAGCCCTGACGCAACCA
AGATCTTCATCAACGGCACGTGGGTTGGTGTCCACAATGAACCAGCGCACTTGGTCACG
CTTGTGCAAGAGCTTCGGCGCAAATGCATCATTTCTCACGAAGTCTCCCTTGTTCGTGAC

ATTCGCGATCGCGAGTTCAAGATTTTCTCCGACGCTGGTCGTGTGATGCGCCCTCTCTTT
GTCGTTAATCAAGACGACAATCCCGAGACTGGAGCGCAGCAGGGCACTCTGGCTCTTAC
CAAGCACCACATTCGCCGTCTCGAGGAAGATGCTCAGTACCATCGCAAGAAGCATGATG
AGGACTATTTCGGCTGGGACGGTCTTCAGAACAGCGGTTGCATCGAGTACCTGGATGCC
GAGGAAGAGGAGACAGCAATGATTTGCATGAGTCCTGAGGATCTCGAGGACTACCGCC
AGCGCAAGGCTAGGGGCAAGGATGCCAAGGATGATGAACCTGAGGATGATGGCAGGAG
TCTGAACGCTCGTGTGAAGACGAAGATCAACCCCGACATCCACATGTACACTCACTGCG
AGATCCATCCAAGCATGCTCCTGGGAATCTGCGCCAGCATCATTCCCTTCCCGGATCACA
ACCAGGTATGTGAAACCTTCGCAGTATTAGCATCACTCCTGCTGACCACATATGTCTAGT
CACCCCGCTCAAATCATCACCATCACAATCGATGGCGCAGACATTTAGGCAAAAGCGGC
CGCGATCCTTTCGTCGCAGCCGTCTGTCCTGCCTAGCTGTGTCCTTTTTTGCCCACCACA
CATTTTCGCTTTGTGGTGCGGGGTTTAGCGTCTGGGTCTTATCTCAGAGATAAACGTGAC
CCACCCAACCGCCACCTCCATCAATTCGCCAACGCATCCATGCGCGCATCCCCCAACCTC
AAAATCTGACGAAGACAATGCTGACCCGTATTCTTCATTGCAGCTGCTGAGCTCGGCAA
GGGTTCCTTCAAGTACGCATGGGTCCTTGACAAGCTCAAGGCCGAGCGTGAGCGTGGTA
TCACCATCGACATTGCTCTGTGGAAGTTCGAGACTCCCAAGTACTATGTCACCGTCATTG
GTAAGTCCACCCTCCCTGGCGATGTCGCGCAGGCCTGGCTGCCTCGCCATGCGCGACTTG
GCGGCTTCTGATGCTTTTGCGGAATCCTGCTAATGCAAATATTACCACAGACGCTCCCGG
TCACCGTGACTTCATCAAGAACATGATCACTGGTACCTCCCAGGCTGACTGCGCCGTTCT
CATCATTGCCTCCGGTACTGGTGAGTTCGAGGCTGGTATCTCCAAGGATGGCCAGACCC
GTGAGCACGCTCTGCTCGCCTACACCCTCGGTGTCAAGCAGCTCATTGTTGCTTGCAACA
AGATGGACACTGCTGGCTGGAAGCAGGACCGTTTCGAGGAGATCAAGAAGGAGACCAC
GAACTTCATCAAGAAGGTCGGCTTCAACCCCAAGCAGGTCGCGTTCGTCCCCATCTCCG
GCTTCCACGGCGACAACATGCTTGAGGCCTCCGCCAACATGCCCTGGTACAAGGGCTGG
ACCAAGGAGACCCAGGACAAGAAGGAGGCCAAGGGTATGACCCTGCTCGACGCCATTG
ACGCCATCGAGCCCCCCAAGCGCCCCACCGAGAAGCCCCTTCGTCTGCCCCTCCAGGAC
GTCTACAAGATCGGCGGTATTGGAACTGTACCTGTCGGCCGTATCGAGACTGGTATCCT
GAAGCCCGGTATGGTCGTCACCTTCGCTCCTTCCAACGTCACCACTGAGGTCAAGTCCGT
CGAGATGCACCACGAGCAGCTCACCGAGGGTCAGCCCGGTGACAACGTTGGTTTCAACG
TCAAGAACGTCTCCGTCAAGGAGATTCGCCGTGGCAACGTCGCCGGTGACTCCAAGAAC
GACCCCCCCATGGCCGCCGCTTCCTTCAACGCCCAGGTCATCGTCCTGAACCACCCCGGC
CAGGTCGGTGCTGGATAC

>Melanconiella_hyperopta

CACACCGCCCGTCGCTACTACCGATTGAATGGTTCAGTGAGGCGTCCGGACTGGCCCCA
GGAGGTCGGCAACGACCACCCAGGGCCGGAAAGTTCTCCAAACTCGATCATTTAGAGGA
AGTAAAAGTCGTAACAAGGTCTCCGTTGGTGAACCAGCGGAGGGATCATTGATGGAACT
ACGTCCCATATTACCCCTGTGAACTTATACCTATATCGTTGCCTCGGCGTAGGCCCGGGG
GCCCTCTCCCCAGAGGGTCCCCCCACCGGTGGGAGCCCCACGGCTTCCCCCCTGTGGAG
CAGGCCCGTCGGCGGCCCCTATAAACCATTACTTGTAAAGAAGACCTTCTGAGTACAAA
CTTAAAATGAATCAAAACTTTCAACAACGGATCTCTTGGTTCTGGCATCGATGAAGAAC
GCAGCGAAATGCGATACGTAATGCGAATTGCAGAATTCAGTGAGTCATCGAATCTTTGA
ACGCACATTGCGCCCGCTGGACATTCCGGCGGGCATGCCTGTTCGAGCGTCATTTCACCC
CTCAGGCTTCAGCGCCTGGTGTTGGGGCATAGCCTGTCAAAGGGCTAGCCCTCAAATTC
AGTGGCGGAACCGCTGGGACTCCGGGCGTAGTAATTTTTCTCGCTTAGGTGGTCTCCAG
TGGTGCCCAGCCGTAAAACCCGCTATCATGCACCCCCCCTAGAGATTGACCTCGGATCA

GGTAGGAATACCCGCTGAACTTAAGCATATCAATAAGCGGAGGAAAAGAAACCAACAG
GGATTGCCCTAGTAACGGCGAGTGAAGCGGCAACAGCTCAAATTTGAAATCTGGCCCCC
CCGGGCCCGAGTTGTAATTTGCAGAGGATGTTCTCTGGCGCGGTGCCTTCCGAGTTCCCT
GGAATGGGACGCCACAGAGGGTGAGAGCCCCGTACGGTCGGACTAACCAAGCCTGTGT
GAGACTCCTTCGACGAGTCGAGTAGTTTGGGAATGCTGCTCTAAATGGGAGGTAAATCT
CTTCTAAAGCTAAATACTGGCCAGAGACCGATAGCGCACAAGTAGAGTGATCGAAAGAT
GAAAAGCACCTTGAAAAGGGGGTTAAACAGTACGTGAAATTGTTGAAAGGGAAGCGCT
CCTGACCAGAAACTTGGGCCGGGCGGCTCAGCAGGGGCTCGCCCCCTGTGTACTCCGTC
CCGGTTCCGGGCCAGCATCGGTTCTCGCTGGGGGACAAGAACGGTGGGAACGTGGCCCC
CCCTCGGGGGGGTGTTATAGCCCGCCGTAACGATACCCTGGTGGGGACCGAGGATTCCG
CGCTTCGGCAAGGATGCTGGCATAATGGTCATCAGCGACCCGTCTTGAAACACGGACCA
AGGAGTCGTCCATTAGAGCGAGCGTTTGGGTGTCAAAACCCGTAGCGCGTAATGAAAGT
GAAATTCTAGGTGAGAACCTCCTCGCGGGGGCGCATCATCGACCGATCCTGATGTTCTC
GGACGGATTTGAGTAAGAGTTTTAACGGACGGACCCGAAAGACAGTGAACTATGCTTGT
ATAGGGTGAAGCCAGAGGAAACTCTGGTGGAGGCTCGCAGCGGTTCTGACGTGCAAATC
GATCGTCAAATATGAGCATGGGGGCGAAAGACTAATCGAACCACACCGCCCGTCGCTAC
TACCGATTGAATGGTTCAGTGAGGCGTCCGGACTGGCCCCAGGAGGTCGGCAACGACCA
CCCAGGGCCGGAAAGTTCTCCAAACTCGATCATTTAGAGGAAGTAAAAGTCGTAACAAG
GTCTCCGTTGGTGAACCAGCGGAGGGATCATTGATGGAACTACGTCCCATATTACCCCT
GTGAACTTATACCTATATCGTTGCCTCGGCGTAGGCCCGGGGGCCCTCTCCCCAGAGGGT
CCCCCCACCGGTGGGAGCCCCACGGCTTCCCCCCTGTGGAGCAGGCCCGTCGGCGGCCC
CTATAAACCATTACTTGTAAAGAAGACCTTCTGAGTACAAACTTAAAATGAATCAAAAC
TTTCAACAACGGATCTCTTGGTTCTGGCATCGATGAAGAACGCAGCGAAATGCGATACG
TAATGCGAATTGCAGAATTCAGTGAGTCATCGAATCTTTGAACGCACATTGCGCCCGCT
GGACATTCCGGCGGGCATGCCTGTTCGAGCGTCATTTCACCCCTCAGGCTTCAGCGCCTG
GTGTTGGGGCATAGCCTGTCAAAGGGCTAGCCCTCAAATTCAGTGGCGGAACCGCTGGG
ACTCCGGGCGTAGTAATTTTTCTCGCTTAGGTGGTCTCCAGTGGTGCCCAGCCGTAAAA
CCCGCTATCATGCACCCCCCCTAGAGATTGACCTCGGATCAGGTAGGAATACCCGCTGA
ACTTAAGCATATCAATAAGCGGAGGAAAAGAAACCAACAGGGATTGCCCTAGTAACGG
CGAGTGAAGCGGCAACAGCTCAAATTTGAAATCTGGCCCCCCCGGGCCCGAGTTGTAAT
TTGCAGAGGATGTTCTCTGGCGCGGTGCCTTCCGAGTTCCCTGGAATGGGACGCCACAG
AGGGTGAGAGCCCCGTACGGTCGGACTAACCAAGCCTGTGTGAGACTCCTTCGACGAGT
CGAGTAGTTTGGGAATGCTGCTCTAAATGGGAGGTAAATCTCTTCTAAAGCTAAATACT
GGCCAGAGACCGATAGCGCACAAGTAGAGTGATCGAAAGATGAAAAGCACCTTGAAAA
GGGGGTTAAACAGTACGTGAAATTGTTGAAAGGGAAGCGCTCCTGACCAGAAACTTGG
GCCGGGCGGCTCAGCAGGGGCTCGCCCCCTGTGTACTCCGTCCCGGTTCCGGGCCAGCA
TCGGTTCTCGCTGGGGGACAAGAACGGTGGGAACGTGGCCCCCCCTCGGGGGGGTGTTA
TAGCCCGCCGTAACGATACCCTGGTGGGGACCGAGGATTCCGCGCTTCGGCAAGGATGC
TGGCATAATGGTCATCAGCGACCCGTCTTGAAACACGGACCAAGGAGTCGTCCATTAGA
GCGAGCGTTTGGGTGTCAAAACCCGTAGCGCGTAATGAAAGTGAAATTCTAGGTGAGAA
CCTCCTCGCGGGGGCGCATCATCGACCGATCCTGATGTTCTCGGACGGATTTGAGTAAG
AGTTTTAACGGACGGACCCGAAAGACAGTGAACTATGCTTGTATAGGGTGAAGCCAGAG
GAAACTCTGGTGGAGGCTCGCAGCGGTTCTGACGTGCAAATCGATCGTCAAATATGAGC
ATGGGGGCGAAAGACTAATCGAACGGTCCGCTCCTGGCAAAGCTGTTCCGAAACATTGT
CAAGAAGCTGACCGGTGACATGATGGCGTACATGAAACGTTGTGTCAGCGAGGGCAAG
CATTTCGATTTGGCGTTGGGCATCCGCCACTCAACCCTCACAAATGGCCTCAAGTACTCT
CTTGCGACTGGAAACTGGGGCGAGCAGAAGAAGGCGATGAGTTCCACCGCTGGTGTCTC
TCAGGTGCTAAACCGGTACACGTTCTCGTCCACTCTATCGCATTTGCGGCGCACGAACAC

TCCCATTGGTCGCGACGGCAAGCTGGCCAAGCCTCGTCAGCTGCACAACACTCACTGGG
GCCTCGTCTGTCCGGCAGAGACTCCTGAGGGACAGGCTTGCGGTCTCGTCAAGAACCTG
TCTTTGATGTGCTACGTCAGCGTCGGTTCACCAGCTGAGCCTATCAAGGACTTCATGGTG
CAACGAAATATGGAAGTCTTGGAGGAATACGAGCCCGGGTCCAGCCCTGACGCAACCA
AGATCTTCATCAACGGCACGTGGGTTGGTGTCCACAATGAACCAGCGCACTTGGTCACG
CTTGTGCAAGAGCTTCGGCGCAAATGCATCATTTCTCACGAAGTCTCCCTTGTTCGTGAC
ATTCGCGATCGCGAGTTCAAGATTTTCTCCGACGCTGGTCGTGTGATGCGCCCTCTCTTT
GTCGTTAATCAAGACGACAATCCCGAGACTGGAGCGCAGCAGGGCACTCTGGCTCTTAC
CAAGCACCACATTCGCCGTCTCGAGGAAGATGCTCAGTACCATCGCAAGAAGCATGATG
AGGACTATTTCGGCTGGGACGGTCTTCAGAACAGCGGTTGCATCGAGTACCTGGATGCC
GAGGAAGAGGAGACAGCAATGATTTGCATGAGTCCTGAGGATCTCGAGGACTACCGCC
AGCGCAAGGCTAGGGGCAAGGATGCCAAGGATGATGAACCTGAGGATGATGGCAGGAG
TCTGAACGCTCGTGTGAAGACGAAGATCAACCCCGACATCCACATGTACACTCACTGCG
AGATCCATCCAAGCATGCTCCTGGGAATCTGCGCCAGCATCATTCCCTTCCCGGATCACA
ACCAGGTATGTGAAACCTTCGCAGTATTAGCATCACTCCTGCTGACCACATATGTCTAGT
CACCCCGCATCACTCAAATCATCACCATCACAATCGATGGCGCAGACATTTAGGCAAAA
GCGGCCGCGATCCTTTCGTCGCAGCCGTCTGTCCTGCCTAGCTGTGTCCTTTTTTTGCCCA
CCACACATTTTCGCTTTGTGGTGCGGGGTTTAGCGTCTGGGTCTTATCTCAGAGATAAAC
GTGACCCACCCAACCGCCACCTCCATCATTCGCCAACGCATCCATGCGCGCATCCCCCA
ACCTCAAAATCTGACGAAGACAATGCTGACCCGTATTCTTCATTGCAGCTGCTGAGCTCG
GCAAGGGTTCCTTCAAGTACGCATGGGTCCTTGACAAGCTCAAGGCCGAGCGTGAGCGT
GGTATCACCATCGACATTGCTCTGTGGAAGTTCGAGACTCCCAAGTACTATGTCACCGTC
ATTGGTAAGTCCACCCTCCCTGGCGATGTCGCGCAGGCCTGGCTGCCTCGCCATGCGCG
ACTTGGCGGCTTCTGATGCTTTTGCGGAATCCTGCTAATGCAAATATTACCACAGACGCT
CCCGGTCACCGTGACTTCATCAAGAACATGATCACTGGTACCTCCCAGGCTGACTGCGC
CGTTCTCATCATTGCCTCCGGTACTGGTGAGTTCGAGGCTGGTATCTCCAAGGATGGCCA
GACCCGTGAGCACGCTCTGCTCGCCTACACCCTCGGTGTCAAGCAGCTCATTGTTGCTTG
CAACAAGATGGACACTGCTGGCTGGAAGCAGGACCGTTTCGAGGAGATCAAGAAGGAG
ACCACGAACTTCATCAAGAAGGTCGGCTTCAACCCCAAGCAGGTCGCGTTCGTCCCCAT
CTCCGGCTTCCACGGCGACAACATGCTTGAGGCCTCCGCCAACATGCCCTGGTACAAGG
GCTGGACCAAGGAGACCCAGGACAAGAAGGAGGCCAAGGGTATGACCCTGCTCGACGC
CATTGACGCCATCGAGCCCCCCAAGCGCCCCACCGAGAAGCCCCTTCGTCTGCCCCTCC
AGGACGTCTACAAGATCGGCGGTATTGGAACTGTACCTGTCGGCCGTATCGAGACTGGT
ATCCTGAAGCCCGGTATGGTCGTCACCTTCGCTCCTTCCAACGTCACCACTGAGGTCAAG
TCCGTCGAGATGCACCACGAGCAGCTCACCGAGGGTCAGCCCGGTGACAACGTTGGTTT
CAACGTCAAGAACGTCTCCGTCAAGGAGATTCGCCGTGGCAACGTCGCCGGTGACTCCA
AGAACGACCCCCCCATGGCCGCCGCTTCCTTCAACGCCCAGGTCATCGTCCTGAACCAC
CCCGGCCAGGTCGGTGCTGGATAC

>Melanconiella_hyperopta_var__orientalis

CACACCGCCCGTCGCTACTACCGATTGAATGGTTCAGTGAGGCGTCCGGACTGGCCCCA
GGAGGTCGGCAACGACCACCCAGGGCCGGAAAGTTCTCCAAACTCGATCATTTAGAGGA
AGTAAAAGTCGTAACAAGGTCTCCGTTGGTGAACCAGCGGAGGGATCATTGATGGAACT
ACGTCCCATATTACCCCTGTGAACTTATACCTATATCGTTGCCTCGGCGTAGGCCCGGGG
GCCCTCTCCCCAGAGGGTCCCCCCACCGGTGGGAGCCCCACGGCTTCCCCCCTGTGGAG
CAGGCCCGTCGGCGGCCCCTATAAACCATTACTTGTAAAGAAGACCTTCTGAGTACAAA

CTTAAAATGAATCAAAACTTTCAACAACGGATCTCTTGGTTCTGGCATCGATGAAGAAC
GCAGCGAAATGCGATACGTAATGCGAATTGCAGAATTCAGTGAGTCATCGAATCTTTGA
ACGCACATTGCGCCCGCTGGACATTCCGGCGGGCATGCCTGTTCGAGCGTCATTTCACCC
CTCAGGCTTCAGCGCCTGGTGTTGGGGCATAGCCTGTCAAAGGGCTAGCCCTCAAATTC
AGTGGCGGACCCGCTGGGACTCCGGGCGTAGTAATTTTTTCTCGCTTAGGTGGTCTCCGG
TGGTGCCCAGCCGTAAAACCCGCTATCATGCACCCCCCTAGAGATTGACCTCGGATCAG
GTAGGAATACCCGCTGAACTTAAGCATATCAATAAGCGGAGGAAAAGAAACCAACAGG
GATTGCCCTAGTAACGGCGAGTGAAGCGGCAACAGCTCAAATTTGAAATCTGGCCCCCC
CGGGCCCGAGTTGTAATTTGCAGAGGATGTTCTCTGGCGCGGTGCCTTCCGAGTTCCCTG
GAATGGGACGCCACAGAGGGTGAGAGCCCCGTACGGTCGGACTAACCAAGCCTGTGTG
AGACTCCTTCGACGAGTCGAGTAGTTTGGGAATGCTGCTCTAAATGGGAGGTAAATCTC
TTCTAAAGCTAAATACTGGCCAGAGACCGATAGCGCACAAGTAGAGTGATCGAAAGAT
GAAAAGCACCTTGAAAAGGGGGTTAAACAGTACGTGAAATTGTTGAAAGGGAAGCGCT
CCTGACCAGAAACTTGGGCCGGGCGGCTCAGCAGGGGCTCGCCCCCTGTGTACTCCGTC
CCGGTTCCGGGCCAGCATCGGTTCTCGCTGGGGGACAAGAACGGTGGGAACGTGGCCCC
CCCTCGGGGGGGTGTTATAGCCCGCCGTAACGATACCCTGGTGGGGACCGAGGATTCCG
CGCTTCGGCAAGGATGCTGGCATAATGGTCATCAGCGACCCGTCTTGAAACACGGACCA
AGGAGTCGTCCATTAGAGCGAGCGTTTGGGTGTCAAAACCCGTAGCGCGTAATGAAAGT
GAAATTCTAGGTGAGAACCTCCTCGCGGGGGCGCATCATCGACCGATCCTGATGTTCTC
GGACGGATTTGAGTAAGAGTTTTAACGGACGGACCCGAAAGACAGTGAACTATGCTTGT
ATAGGGTGAAGCCAGAGGAAACTCTGGTGGAGGCTCGCAGCGGTTCTGACGTGCAAATC
GATCGTCAAATATGAGCATGGGGGCGAAAGACTAATCGAACCACACCGCCCGTCGCTAC
TACCGATTGAATGGTTCAGTGAGGCGTCCGGACTGGCCCCAGGAGGTCGGCAACGACCA
CCCAGGGCCGGAAAGTTCTCCAAACTCGATCATTTAGAGGAAGTAAAAGTCGTAACAAG
GTCTCCGTTGGTGAACCAGCGGAGGGATCATTGATGGAACTACGTCCCATATTACCCCT
GTGAACTTATACCTATATCGTTGCCTCGGCGTAGGCCCGGGGGCCCTCTCCCCAGAGGGT
CCCCCCACCGGTGGGAGCCCCACGGCTTCCCCCCTGTGGAGCAGGCCCGTCGGCGGCCC
CTATAAACCATTACTTGTAAAGAAGACCTTCTGAGTACAAACTTAAAATGAATCAAAAC
TTTCAACAACGGATCTCTTGGTTCTGGCATCGATGAAGAACGCAGCGAAATGCGATACG
TAATGCGAATTGCAGAATTCAGTGAGTCATCGAATCTTTGAACGCACATTGCGCCCGCT
GGACATTCCGGCGGGCATGCCTGTTCGAGCGTCATTTCACCCCTCAGGCTTCAGCGCCTG
GTGTTGGGGCATAGCCTGTCAAAGGGCTAGCCCTCAAATTCAGTGGCGGACCCGCTGGG
ACTCCGGGCGTAGTAATTTTTTCTCGCTTAGGTGGTCTCCGGTGGTGCCCAGCCGTAAAA
CCCGCTATCATGCACCCCCCTAGAGATTGACCTCGGATCAGGTAGGAATACCCGCTGAA
CTTAAGCATATCAATAAGCGGAGGAAAAGAAACCAACAGGGATTGCCCTAGTAACGGC
GAGTGAAGCGGCAACAGCTCAAATTTGAAATCTGGCCCCCCCGGGCCCGAGTTGTAATT
TGCAGAGGATGTTCTCTGGCGCGGTGCCTTCCGAGTTCCCTGGAATGGGACGCCACAGA
GGGTGAGAGCCCCGTACGGTCGGACTAACCAAGCCTGTGTGAGACTCCTTCGACGAGTC
GAGTAGTTTGGGAATGCTGCTCTAAATGGGAGGTAAATCTCTTCTAAAGCTAAATACTG
GCCAGAGACCGATAGCGCACAAGTAGAGTGATCGAAAGATGAAAAGCACCTTGAAAAG
GGGGTTAAACAGTACGTGAAATTGTTGAAAGGGAAGCGCTCCTGACCAGAAACTTGGGC
CGGGCGGCTCAGCAGGGGCTCGCCCCCTGTGTACTCCGTCCCGGTTCCGGGCCAGCATC
GGTTCTCGCTGGGGGACAAGAACGGTGGGAACGTGGCCCCCCCTCGGGGGGGTGTTATA
GCCCGCCGTAACGATACCCTGGTGGGGACCGAGGATTCCGCGCTTCGGCAAGGATGCTG
GCATAATGGTCATCAGCGACCCGTCTTGAAACACGGACCAAGGAGTCGTCCATTAGAGC
GAGCGTTTGGGTGTCAAAACCCGTAGCGCGTAATGAAAGTGAAATTCTAGGTGAGAACC
TCCTCGCGGGGGCGCATCATCGACCGATCCTGATGTTCTCGGACGGATTTGAGTAAGAG
TTTTAACGGACGGACCCGAAAGACAGTGAACTATGCTTGTATAGGGTGAAGCCAGAGGA

AACTCTGGTGGAGGCTCGCAGCGGTTCTGACGTGCAAATCGATCGTCAAATATGAGCAT
GGGGGCGAAAGACTAATCGAACGGTCCGCTCCTGGCAAAGCTGTTCCGAAATATTGTCA
AGAAGCTGACCGGTGACATGATGGCGTACATGAAACGTTGTGTCAGTGAGGGCAAGCAT
TTCGATCTGGCGTTGGGCATCCGCCACTCAACCCTCACAAATGGCCTCAAGTACTCTCTT
GCGACTGGAAACTGGGGCGAGCAGAAGAAGGCGATGAGTTCCACCGCTGGTGTCTCTCA
GGTGCTAAACCGGTACACGTTCTCGTCCACTCTATCGCATTTGCGGCGCACGAACACCCC
CATTGGTCGCGACGGCAAGTTGGCCAAGCCTCGTCAGCTGCACAACACTCACTGGGGCC
TCGTCTGTCCGGCAGAGACTCCTGAGGGACAGGCCTGCGGTCTCGTCAAGAACCTGTCT
TTGATGTGCTACGTCAGCGTCGGTTCACCGGCTGAGCCTATCAAGGACTTCATGGTGCAA
CGAAATATGGAAGTCTTGGAGGAATACGAGCCCGGGTCCAGCCCTGACGCAACCAAGA
TCTTCATCAACGGCACGTGGGTTGGTGTCCACAATGAACCGGCACACTTGGTCACGCTTG
TGCAAGAGCTTCGGCGCAAATGCATCATTTCTCACGAAGTCTCCCTTGTTCGTGACATTC
GCGATCGCGAGTTCAAGATTTTCTCCGACGCTGGTCGTGTGATGCGCCCTCTCTTTGTCG
TCAATCAAGACGACAACCCCGAGACTGGAGCGCAGCAGGGCACTCTGGCTCTTACCAAG
CACCACATTCGCCGTCTCGAGGAAGATGCTCAGTACCATCGCAAGAAGCATGATGAGGA
CTATTTCGGCTGGGACGGTCTTCAGAACAGCGGTTGCATCGAGTACCTGGATGCCGAGG
AAGAGGAGACAGCAATGATTTGCATGAGTCCTGAGGATCTCGAGGACTACCGCCAGCGC
AAGGCTAGGGGCAAGGATGCCAAGGATGATGAACCTGAGGATGATGGCAGGAGTCTGA
ACGCTCGTGTGAAGACGAAGATCAACCCCGACATCCACATGTACACTCACTGCGAGATC
CATCCAAGCATGCTCCTGGGAATCTGCGCCAGTATCATTCCCTTCCCGGATCACAACCAG
GTATGTAAAACCTTCGCAGTATTAGCATCACTCCTGCTGACCACATATGTCTAGTCACCC
CGCATCACTCAAACCATCACCATCACGATCGATGGCGCAGACATTTAGGCAGAAGCGGC
CGCGATCCTTTCGCCGCGGCCGTTTGTCCTGCCTAGCTGTGTCCTTTTTTTGCCCACCACA
CATTTTCGCTTTGTGGTGCGGGGTTTAGCGTCTGGGTCTTATCTCAGAGATAAACGTGAC
CCACCCAACCGCCACCTCCATCATTCGCCAACGCATCCATGCGCGCATCCCCCACCCTCA
AAATCTGACGAAAACCATGCTGACCCGTATTCTTCATTGCAGCTGCTGAGCTCGGCAAG
GGTTCCTTCAAGTACGCATGGGTCCTTGACAAGCTCAAGGCCGAGCGTGAGCGTGGTAT
CACCATCGACATTGCTCTGTGGAAGTTCGAGACTCCCAAGTACTATGTCACCGTCATTGG
TAAGTCCACCCTCCCTGGCGATGTCACGCAGGCCTGGCTGCCTCGCCATGCGCGACTTGG
CGGCTTCTGATGCTTTTGCGGAATCCTGCTAATGCAAATATCACCACAGACGCTCCCGGT
CACCGTGACTTCATCAAGAACATGATCACTGGTACCTCCCAGGCTGACTGCGCCGTTCTC
ATCATTGCCTCCGGTACTGGTGAGTTCGAGGCTGGTATCTCCAAGGATGGCCAGACCCG
TGAGCACGCTCTGCTCGCCTACACCCTCGGTGTCAAGCAGCTCATTGTTGCTTGCAACAA
GATGGACACTGCTGGCTGGAAGCAGGACCGTTTCGAGGAGATCAAGAAGGAGACCACG
AACTTCATCAAGAAGGTCGGCTTCAACCCCAAGCAGGTCGCGTTCGTCCCCATCTCCGG
CTTCCACGGCGACAACATGCTTGAGGCCTCCGCCAACATGCCCTGGTACAAGGGCTGGA
CCAAGGAGACCCAGGACAAGAAGGAGGCCAAGGGTATGACCCTGCTCGACGCCATTGA
CGCCATCGAGCCCCCCAAGCGCCCCACCGAGAAGCCCCTTCGTCTGCCCCTCCAGGACG
TCTACAAGATCGGCGGTATTGGAACTGTACCTGTCGGCCGTATCGAGACTGGTATCCTG
AAGCCCGGTATGGTCGTCACCTTCGCTCCTTCCAACGTCACCACTGAGGTCAAGTCCGTC
GAGATGCACCACGAGCAGCTCACCGAGGGTCAGCCCGGTGACAACGTTGGTTTCAACGT
CAAGAACGTCTCCGTCAAGGAGATTCGCCGTGGCAACGTCGCTGGTGACTCCAAGAACG
ACCCCCCCATGGCCGCCGCTTCCTTCAACGCCCAGGTCATCGTCCTGAACCACCCCGGCC
AGGTCGGTGCTGGATAC

>Melanconiella_hyperopta_var__orientalis

CACACCGCCCGTCGCTACTACCGATTGAATGGTTCAGTGAGGCGTCCGGACTGGCCCCA
GGAGGTCGGCAACGACCACCCAGGGCCGGAAAGTTCTCCAAACTCGATCATTTAGAGGA
AGTAAAAGTCGTAACAAGGTCTCCGTTGGTGAACCAGCGGAGGGATCATTGATGGAACT
ACGTCCCATATTACCCCTGTGAACTTATACCTATATCGTTGCCTCGGCGTAGGCCCGGGG
GCCCTCTCCCCAGAGGGTCCCCCCACCGGTGGGAGCCCCACGGCTTCCCCCCTGTGGAG
CAGGCCCGTCGGCGGCCCCTATAAACCATTACTTGTAAAGAAGACCTTCTGAGTACAAA
CTTAAAATGAATCAAAACTTTCAACAACGGATCTCTTGGTTCTGGCATCGATGAAGAAC
GCAGCGAAATGCGATACGTAATGCGAATTGCAGAATTCAGTGAGTCATCGAATCTTTGA
ACGCACATTGCGCCCGCTGGACATTCCGGCGGGCATGCCTGTTCGAGCGTCATTTCACCC
CTCAGGCTTCAGCGCCTGGTGTTGGGGCATAGCCTGTCAAAGGGCTAGCCCTCAAATTC
AGTGGCGGACCCGCTGGGACTCCGGGCGTAGTAATTTTTTCTCGCTTAGGTGGTCTCCGG
TGGTGCCCAGCCGTAAAACCCGCTATCATGCACCCCCCTAGAGATTGACCTCGGATCAG
GTAGGAATACCCGCTGAACTTAAGCATATCAATAAGCGGAGGAAAAGAAACCAACAGG
GATTGCCCTAGTAACGGCGAGTGAAGCGGCAACAGCTCAAATTTGAAATCTGGCCCCCC
CGGGCCCGAGTTGTAATTTGCAGAGGATGTTCTCTGGCGCGGTGCCTTCCGAGTTCCCTG
GAATGGGACGCCACAGAGGGTGAGAGCCCCGTACGGTCGGACTAACCAAGCCTGTGTG
AGACTCCTTCGACGAGTCGAGTAGTTTGGGAATGCTGCTCTAAATGGGAGGTAAATCTC
TTCTAAAGCTAAATACTGGCCAGAGACCGATAGCGCACAAGTAGAGTGATCGAAAGAT
GAAAAGCACCTTGAAAAGGGGGTTAAACAGTACGTGAAATTGTTGAAAGGGAAGCGCT
CCTGACCAGAAACTTGGGCCGGGCGGCTCAGCAGGGGCTCGCCCCCTGTGTACTCCGTC
CCGGTTCCGGGCCAGCATCGGTTCTCGCTGGGGGACAAGAACGGTGGGAACGTGGCCCC
CCCTCGGGGGGGTGTTATAGCCCGCCGTAACGATACCCTGGTGGGGACCGAGGATTCCG
CGCTTCGGCAAGGATGCTGGCATAATGGTCATCAGCGACCCGTCTTGAAACACGGACCA
AGGAGTCGTCCATTAGAGCGAGCGTTTGGGTGTCAAAACCCGTAGCGCGTAATGAAAGT
GAAATTCTAGGTGAGAACCTCCTCGCGGGGGCGCATCATCGACCGATCCTGATGTTCTC
GGACGGATTTGAGTAAGAGTTTTAACGGACGGACCCGAAAGACAGTGAACTATGCTTGT
ATAGGGTGAAGCCAGAGGAAACTCTGGTGGAGGCTCGCAGCGGTTCTGACGTGCAAATC
GATCGTCAAATATGAGCATGGGGGCGAAAGACTAATCGAACCACACCGCCCGTCGCTAC
TACCGATTGAATGGTTCAGTGAGGCGTCCGGACTGGCCCCAGGAGGTCGGCAACGACCA
CCCAGGGCCGGAAAGTTCTCCAAACTCGATCATTTAGAGGAAGTAAAAGTCGTAACAAG
GTCTCCGTTGGTGAACCAGCGGAGGGATCATTGATGGAACTACGTCCCATATTACCCCT
GTGAACTTATACCTATATCGTTGCCTCGGCGTAGGCCCGGGGGCCCTCTCCCCAGAGGGT
CCCCCCACCGGTGGGAGCCCCACGGCTTCCCCCCTGTGGAGCAGGCCCGTCGGCGGCCC
CTATAAACCATTACTTGTAAAGAAGACCTTCTGAGTACAAACTTAAAATGAATCAAAAC
TTTCAACAACGGATCTCTTGGTTCTGGCATCGATGAAGAACGCAGCGAAATGCGATACG
TAATGCGAATTGCAGAATTCAGTGAGTCATCGAATCTTTGAACGCACATTGCGCCCGCT
GGACATTCCGGCGGGCATGCCTGTTCGAGCGTCATTTCACCCCTCAGGCTTCAGCGCCTG
GTGTTGGGGCATAGCCTGTCAAAGGGCTAGCCCTCAAATTCAGTGGCGGACCCGCTGGG
ACTCCGGGCGTAGTAATTTTTTCTCGCTTAGGTGGTCTCCGGTGGTGCCCAGCCGTAAAA
CCCGCTATCATGCACCCCCCTAGAGATTGACCTCGGATCAGGTAGGAATACCCGCTGAA
CTTAAGCATATCAATAAGCGGAGGAAAAGAAACCAACAGGGATTGCCCTAGTAACGGC
GAGTGAAGCGGCAACAGCTCAAATTTGAAATCTGGCCCCCCCGGGCCCGAGTTGTAATT
TGCAGAGGATGTTCTCTGGCGCGGTGCCTTCCGAGTTCCCTGGAATGGGACGCCACAGA
GGGTGAGAGCCCCGTACGGTCGGACTAACCAAGCCTGTGTGAGACTCCTTCGACGAGTC
GAGTAGTTTGGGAATGCTGCTCTAAATGGGAGGTAAATCTCTTCTAAAGCTAAATACTG
GCCAGAGACCGATAGCGCACAAGTAGAGTGATCGAAAGATGAAAAGCACCTTGAAAAG
GGGGTTAAACAGTACGTGAAATTGTTGAAAGGGAAGCGCTCCTGACCAGAAACTTGGGC
CGGGCGGCTCAGCAGGGGCTCGCCCCCTGTGTACTCCGTCCCGGTTCCGGGCCAGCATC

GGTTCTCGCTGGGGGACAAGAACGGTGGGAACGTGGCCCCCCCTCGGGGGGGTGTTATA
GCCCGCCGTAACGATACCCTGGTGGGGACCGAGGATTCCGCGCTTCGGCAAGGATGCTG
GCATAATGGTCATCAGCGACCCGTCTTGAAACACGGACCAAGGAGTCGTCCATTAGAGC
GAGCGTTTGGGTGTCAAAACCCGTAGCGCGTAATGAAAGTGAAATTCTAGGTGAGAACC
TCCTCGCGGGGGCGCATCATCGACCGATCCTGATGTTCTCGGACGGATTTGAGTAAGAG
TTTTAACGGACGGACCCGAAAGACAGTGAACTATGCTTGTATAGGGTGAAGCCAGAGGA
AACTCTGGTGGAGGCTCGCAGCGGTTCTGACGTGCAAATCGATCGTCAAATATGAGCAT
GGGGGCGAAAGACTAATCGAACGGTCCGCTCCTGGCAAAGCTGTTCCGAAATATTGTCA
AGAAGCTGACCGGTGACATGATGGCGTACATGAAACGTTGTGTCAGTGAGGGCAAGCAT
TTCGATCTGGCGTTGGGCATCCGCCACTCAACCCTCACAAATGGCCTCAAGTACTCTCTT
GCGACTGGAAACTGGGGCGAGCAGAAGAAGGCGATGAGTTCCACCGCTGGTGTCTCTCA
GGTGCTAAACCGGTACACGTTCTCGTCCACTCTATCGCATTTGCGGCGCACGAACACCCC
CATTGGTCGCGACGGCAAGTTGGCCAAGCCTCGTCAGCTGCACAACACTCACTGGGGCC
TCGTCTGTCCGGCAGAGACTCCTGAGGGACAGGCCTGCGGTCTCGTCAAGAACCTGTCT
TTGATGTGCTACGTCAGCGTCGGTTCACCGGCTGAGCCTATCAAGGACTTCATGGTGCAA
CGAAATATGGAAGTCTTGGAGGAATACGAGCCCGGGTCCAGCCCTGACGCAACCAAGA
TCTTCATCAACGGCACGTGGGTTGGTGTCCACAATGAACCGGCACACTTGGTCACGCTTG
TGCAAGAGCTTCGGCGCAAATGCATCATTTCTCACGAAGTCTCCCTTGTTCGTGACATTC
GCGATCGCGAGTTCAAGATTTTCTCCGACGCTGGTCGTGTGATGCGCCCTCTCTTTGTCG
TCAATCAAGACGACAACCCCGAGACTGGAGCGCAGCAGGGCACTCTGGCTCTTACCAAG
CACCACATTCGCCGTCTCGAGGAAGATGCTCAGTACCATCGCAAGAAGCATGATGAGGA
CTATTTCGGCTGGGACGGTCTTCAGAACAGCGGTTGCATCGAGTACCTGGATGCCGAGG
AAGAGGAGACAGCAATGATTTGCATGAGTCCTGAGGATCTCGAGGACTACCGCCAGCGC
AAGGCTAGGGGCAAGGATGCCAAGGATGATGAACCTGAGGATGATGGCAGGAGTCTGA
ACGCTCGTGTGAAGACGAAGATCAACCCCGACATCCACATGTACACTCACTGCGAGATC
CATCCAAGCATGCTCCTGGGAATCTGCGCCAGTATCATTCCCTTCCCGGATCACAACCAG
GTATGTAAAACCTTCGCAGTATTAGCATCACTCCTGCTGACCACATATGTCTAGTCACCC
CGCATCACTCAAACCATCACCATCACGATCGATGGCGCAGACATTTAGGCAGAAGCGGC
CGCGATCCTTTCGCCGCGGCCGTTTGTCCTGCCTAGCTGTGTCCTTTTTTTGCCCACCACA
CATTTTCGCTTTGTGGTGCGGGGTTTAGCGTCTGGGTCTTATCTCAGAGATAAACGTGAC
CCACCCAACCGCCACCTCCATCATTCGCCAACGCATCCATGCGCGCATCCCCCACCCTCA
AAATCTGACGAAAACCATGCTGACCCGTATTCTTCATTGCAGCTGCTGAGCTCGGCAAG
GGTTCCTTCAAGTACGCATGGGTCCTTGACAAGCTCAAGGCCGAGCGTGAGCGTGGTAT
CACCATCGACATTGCTCTGTGGAAGTTCGAGACTCCCAAGTACTATGTCACCGTCATTGG
TAAGTCCACCCTCCCTGGCGATGTCACGCAGGCCTGGCTGCCTCGCCATGCGCGACTTGG
CGGCTTCTGATGCTTTTGCGGAATCCTGCTAATGCAAATATCACCACAGACGCTCCCGGT
CACCGTGACTTCATCAAGAACATGATCACTGGTACCTCCCAGGCTGACTGCGCCGTTCTC
ATCATTGCCTCCGGTACTGGTGAGTTCGAGGCTGGTATCTCCAAGGATGGCCAGACCCG
TGAGCACGCTCTGCTCGCCTACACCCTCGGTGTCAAGCAGCTCATTGTTGCTTGCAACAA
GATGGACACTGCTGGCTGGAAGCAGGACCGTTTCGAGGAGATCAAGAAGGAGACCACG
AACTTCATCAAGAAGGTCGGCTTCAACCCCAAGCAGGTCGCGTTCGTCCCCATCTCCGG
CTTCCACGGCGACAACATGCTTGAGGCCTCCGCCAACATGCCCTGGTACAAGGGCTGGA
CCAAGGAGACCCAGGACAAGAAGGAGGCCAAGGGTATGACCCTGCTCGACGCCATTGA
CGCCATCGAGCCCCCCAAGCGCCCCACCGAGAAGCCCCTTCGTCTGCCCCTCCAGGACG
TCTACAAGATCGGCGGTATTGGAACTGTACCTGTCGGCCGTATCGAGACTGGTATCCTG
AAGCCCGGTATGGTCGTCACCTTCGCTCCTTCCAACGTCACCACTGAGGTCAAGTCCGTC
GAGATGCACCACGAGCAGCTCACCGAGGGTCAGCCCGGTGACAACGTTGGTTTCAACGT

CAAGAACGTCTCCGTCAAGGAGATTCGCCGTGGCAACGTCGCTGGTGACTCCAAGAACG
ACCCCCCCATGGCCGCCGCTTCCTTCAACGCCCAGGTCATCGTCCTGAACCACCCCGGCC
AGGTCGGTGCTGGATAC

>Melanconiella_hyperopta_var__orientalis

TACCGATTGAATGGTTCAGTGAGGCGTCCGGACTGGCCCCAGGAGGTCGGCAACGACCA
CCCAGGGCCGGAAAGTTCTCCAAACTCGATCATTTAGAGGAAGTAAAAGTCGTAACAAG
GTCTCCGTTGGTGAACCAGCGGAGGGATCATTGATGGAACTACGTCCCATATTACCCCT
GTGAACTTATACCTATATCGTTGCCTCGGCGTAGGCCCGGGGGCCCTCTCCCCAGAGGGT
CCCCCCACCGGTGGGAGCCCCACGGCTTCCCCCCTGTGGAGCAGGCCCGTCGGCGGCCC
CTATAAACCATTACTTGTAAAGAAGACCTTCTGAGTACAAACTTAAAATGAATCAAAAC
TTTCAACAACGGATCTCTTGGTTCTGGCATCGATGAAGAACGCAGCGAAATGCGATACG
TAATGCGAATTGCAGAATTCAGTGAGTCATCGAATCTTTGAACGCACATTGCGCCCGCT
GGACATTCCGGCGGGCATGCCTGTTCGAGCGTCATTTCACCCCTCAGGCTTCAGCGCCTG
GTGTTGGGGCATAGCCTGTCAAAGGGCTAGCCCTCAAATTCAGTGGCGGACCCGCTGGG
ACTCCGGGCGTAGTAATTTTTCTCGCTTAGGTGGTCTCCGGTGGTGCCCAGCCGTAAAA
CCCGCTATCATGCACCCCCCTAGAGATTGACCTCGGATCAGGTAGGAATACCCGCTGAA
CTTAAGCATATCAATAAGCGGAGGAAAAGAAACCAACAGGGATTGCCCTAGTAACGGC
GAGTGAAGCGGCAACAGCTCAAATTTGAAATCTGGCCCCCCCGGGCCCGAGTTGTAATT
TGCAGAGGATGTTCTCTGGCGCGGTGCCTTCCGAGTTCCCTGGAATGGGACGCCACAGA
GGGTGAGAGCCCCGTACGGTCGGACTAACCAAGCCTGTGTGAGACTCCTTCGACGAGTC
GAGTAGTTTGGGAATGCTGCTCTAAATGGGAGGTAAATCTCTTCTAAAGCTAAATACTG
GCCAGAGACCGATAGCGCACAAGTAGAGTGATCGAAAGATGAAAAGCACCTTGAAAAG
GGGGTTAAACAGTACGTGAAATTGTTGAAAGGGAAGCGCTCCTGACCAGAAACTTGGGC
CGGGCGGCTCAGCAGGGGCTCGCCCCCTGTGTACTCCGTCCCGGTTCCGGGCCAGCATC
GGTTCTCGCTGGGGGACAAGAACGGTGGGAACGTGGCCCCCCCCTCGGGGGGGTGTTAT
AGCCCGCCGTAACGATACCCTGGTGGGGACCGAGGATTCCGCGCTTCGGCAAGGATGCT
GGCATAATGGTCATCAGCGACCCGTCTTGAAACACGGACCAAGGAGTCGTCCATTAGAG
CGAGCGTTTGGGTGTCAAAACCCGTAGCGCGTAATGAAAGTGAAATTCTAGGTGAGAAC
CTCCTCGCGGGGGCGCATCATCGACCGATCCTGATGTTCTCGGACGGATTTGAGTAAGA
GTTTTAACGGACGGACCCGAAAGACAGTGAACTATGCTTGTATAGGGTGAAGCCAGAGG
AAACTCTGGTGGAGGCTCGCAGCGGTTCTGACGTGCAAATCGATCGTCAAATATGAGCA
TGGGGGCGAAAGACTAATCGAACTACCGATTGAATGGTTCAGTGAGGCGTCCGGACTGG
CCCCAGGAGGTCGGCAACGACCACCCAGGGCCGGAAAGTTCTCCAAACTCGATCATTTA
GAGGAAGTAAAAGTCGTAACAAGGTCTCCGTTGGTGAACCAGCGGAGGGATCATTGAT
GGAACTACGTCCCATATTACCCCTGTGAACTTATACCTATATCGTTGCCTCGGCGTAGGC
CCGGGGGCCCTCTCCCCAGAGGGTCCCCCCACCGGTGGGAGCCCCACGGCTTCCCCCCT
GTGGAGCAGGCCCGTCGGCGGCCCCTATAAACCATTACTTGTAAAGAAGACCTTCTGAG
TACAAACTTAAAATGAATCAAAACTTTCAACAACGGATCTCTTGGTTCTGGCATCGATG
AAGAACGCAGCGAAATGCGATACGTAATGCGAATTGCAGAATTCAGTGAGTCATCGAAT
CTTTGAACGCACATTGCGCCCGCTGGACATTCCGGCGGGCATGCCTGTTCGAGCGTCATT
TCACCCCTCAGGCTTCAGCGCCTGGTGTTGGGGCATAGCCTGTCAAAGGGCTAGCCCTC
AAATTCAGTGGCGGACCCGCTGGGACTCCGGGCGTAGTAATTTTTCTCGCTTAGGTGGT
CTCCGGTGGTGCCCAGCCGTAAAACCCGCTATCATGCACCCCCCTAGAGATTGACCTCG
GATCAGGTAGGAATACCCGCTGAACTTAAGCATATCAATAAGCGGAGGAAAAGAAACC
AACAGGGATTGCCCTAGTAACGGCGAGTGAAGCGGCAACAGCTCAAATTTGAAATCTGG
CCCCCCCGGGCCCGAGTTGTAATTTGCAGAGGATGTTCTCTGGCGCGGTGCCTTCCGAGT

TCCCTGGAATGGGACGCCACAGAGGGTGAGAGCCCCGTACGGTCGGACTAACCAAGCCT
GTGTGAGACTCCTTCGACGAGTCGAGTAGTTTGGGAATGCTGCTCTAAATGGGAGGTAA
ATCTCTTCTAAAGCTAAATACTGGCCAGAGACCGATAGCGCACAAGTAGAGTGATCGAA
AGATGAAAAGCACCTTGAAAAGGGGGTTAAACAGTACGTGAAATTGTTGAAAGGGAAG
CGCTCCTGACCAGAAACTTGGGCCGGGCGGCTCAGCAGGGGCTCGCCCCCTGTGTACTC
CGTCCCGGTTCCGGGCCAGCATCGGTTCTCGCTGGGGGACAAGAACGGTGGGAACGTGG
CCCCCCCCTCGGGGGGGTGTTATAGCCCGCCGTAACGATACCCTGGTGGGGACCGAGGA
TTCCGCGCTTCGGCAAGGATGCTGGCATAATGGTCATCAGCGACCCGTCTTGAAACACG
GACCAAGGAGTCGTCCATTAGAGCGAGCGTTTGGGTGTCAAAACCCGTAGCGCGTAATG
AAAGTGAAATTCTAGGTGAGAACCTCCTCGCGGGGGCGCATCATCGACCGATCCTGATG
TTCTCGGACGGATTTGAGTAAGAGTTTTAACGGACGGACCCGAAAGACAGTGAACTATG
CTTGTATAGGGTGAAGCCAGAGGAAACTCTGGTGGAGGCTCGCAGCGGTTCTGACGTGC
AAATCGATCGTCAAATATGAGCATGGGGGCGAAAGACTAATCGAACGGTCCGCTCCTGG
CAAAGCTGTTCCGAAATATTGTCAAGAAGCTGACCGGTGACATGATGGCGTACATGAAA
CGTTGTGTCAGTGAGGGCAAGCATTTCGATCTGGCGTTGGGCATCCGCCACTCAACCCTC
ACAAATGGCCTCAAGTACTCTCTTGCGACTGGAAACTGGGGCGAGCAGAAGAAGGCGA
TGAGTTCCACCGCTGGTGTCTCTCAGGTGCTAAACCGGTACACGTTCTCGTCCACTCTAT
CGCATTTGCGGCGCACGAACACCCCCATTGGTCGCGACGGCAAGTTGGCCAAGCCTCGT
CAGCTGCACAACACTCACTGGGGCCTCGTCTGTCCGGCAGAGACTCCTGAGGGACAGGC
CTGCGGTCTCGTCAAGAACCTGTCTTTGATGTGCTACGTCAGCGTCGGTTCACCGGCTGA
GCCTATCAAGGACTTCATGGTGCAACGAAATATGGAAGTCTTGGAGGAATACGAGCCCG
GGTCCAGCCCTGACGCAACCAAGATCTTCATCAACGGCACGTGGGTTGGTGTCCACAAT
GAACCGGCACACTTGGTCACGCTTGTGCAAGAGCTTCGGCGCAAATGCATCATTTCTCA
CGAAGTCTCCCTTGTTCGTGACATTCGCGATCGCGAGTTCAAGATTTCTCCGACGCTGG
TCGTGTGATGCGCCCTCTCTTTGTCGTCAATCAAGACGACAACCCCGAGACTGGAGCGC
AGCAGGGCACTCTGGCTCTTACCAAGCACCACATTCGCCGTCTCGAGGAAGATGCTCAG
TACCATCGCAAGAAGCATGATGAGGACTATTTCGGCTGGGACGGTCTTCAGAACAGCGG
TTGCATCGAGTACCTGGATGCCGAGGAAGAGGAGACAGCAATGATTTGCATGAGTCCTG
AGGATCTCGAGGACTACCGCCAGCGCAAGGCTAGGGGCAAGGATGCCAAGGATGATGA
ACCTGAGGATGATGGCAGGAGTCTGAACGCTCGTGTGAAGACGAAGATCAACCCCGAC
ATCCACATGTACACTCACTGCGAGATCCATCCAAGCATGCTCCTGGGAATCTGCGCCAG
TATCATTCCCTTCCCGGATCACAACCAGGTATGTAAAACCTTCGCAGTATTAGCATCACT
CCTGCTGACCACATATGTCTAGTCACCCCGCATCACTCAAACCATCACCATCACGATCGA
TGGCGCAGACATTTAGGCAGAAGCGGCCGCGATCCTTTCGCCGCGGCCGTTTGTCCTGC
CTAGCTGTGTCCTTTTTTTGCCCACCACACATTTTCGCTTTGTGGTGCGGGGTTTAGCGTC
TGGGTCTTATCTCAGAGATAAACGTGACCCACCCAACCGCCACCTCCATCATTCGCCAAC
GCATCCATGCGCGCATCCCCCACCCTCAAAATCTGACGAAAACCATGCTGACCCGTATT
CTTCATTGCAGCTGCTGAGCTCGGCAAGGGTTCCTTCAAGTACGCATGGGTCCTTGACAA
GCTCAAGGCCGAGCGTGAGCGTGGTATCACCATCGACATTGCTCTGTGGAAGTTCGAGA
CTCCCAAGTACTATGTCACCGTCATTGGTAAGTCCACCCTCCCTGGCGATGTCACGCAGG
CCTGGCTGCCTCGCCATGCGCGACTTGGCGGCTTCTGATGCTTTTGCGGAATCCTGCTAA
TGCAAATATCACCACAGACGCTCCCGGTCACCGTGACTTCATCAAGAACATGATCACTG
GTACCTCCCAGGCTGACTGCGCCGTTCTCATCATTGCCTCCGGTACTGGTGAGTTCGAGG
CTGGTATCTCCAAGGATGGCCAGACCCGTGAGCACGCTCTGCTCGCCTACACCCTCGGT
GTCAAGCAGCTCATTGTTGCTTGCAACAAGATGGACACTGCTGGCTGGAAGCAGGACCG
TTTCGAGGAGATCAAGAAGGAGACCACGAACTTCATCAAGAAGGTCGGCTTCAACCCCA
AGCAGGTCGCGTTCGTCCCCATCTCCGGCTTCCACGGCGACAACATGCTTGAGGCCTCCG

CCAACATGCCCTGGTACAAGGGCTGGACCAAGGAGACCCAGGACAAGAAGGAGGCCAA
GGGTATGACCCTGCTCGACGCCATTGACGCCATCGAGCCCCCCAAGCGCCCCACCGAGA
AGCCCCTTCGTCTGCCCCTCCAGGACGTCTACAAGATCGGCGGTATTGGAACTGTACCTG
TCGGCCGTATCGAGACTGGTATCCTGAAGCCCGGTATGGTCGTCACCTTCGCTCCTTCCA
ACGTCACCACTGAGGTCAAGTCCGTCGAGATGCACCACGAGCAGCTCACCGAGGGTCAG
CCCGGTGACAACGTTGGTTTCAACGTCAAGAACGTCTCCGTCAAGGAGATTCGCCGTGG
CAACGTCGCTGGTGACTCCAAGAACGACCCCCCCATGGCCGCCGCTTCCTTCAACGCCC
AGGTCATCGTCCTGAACCACCCCGGCCAGGTCGGTGCTGGATAC

>Melanconiella_meridionalis

CACACCGCCCGTCGCTACTACCGATTGAATGGTTCAGTGAGGCGTTCGGACTGGCCCAG
GGAGGTCGGCAACGACCACCCAGGGCCGGAAAGTTCTCCAAACTCGATCATTTAGAGGA
AGTAAAAGTCGTAACAAGGTCTCCGTTGGTGAACCAGCGGAGGGATCATTGATGGAAA
ACAATTCCTATACCCCTTTGTGAACTTATACCTATCTCGTTGCCTCGGCGCCAGGCCCGG
GGTTCCCCCCAGGGGGTTCCCCCACCAGGGACCTCACGGTCCCGGGTGGAGCAGGCCC
GCCGGCGGCCCCCCTAAACTCTTGTTTTAGAACCTATCCCCTCTGAGTTTGTTACCTAA
ATGAATCAAAACTTTCAACAACGGATCTCTTGGCTCTGGCATCGATGAAGAACGCAGCG
AAATGCGATAAGTAATGTGAATTGCAGAATTCAGTGAATCATCGAATCTTTGAACGCAC
ATTGCGCCCGCTGGAATTCCGGCGGGCATGCCTGTTCGAGCGTCATTTCAACCCCTCAAG
CCCCCGTGCTTGGTGTTGGGGCATAGCCCGTAGAAAGGCTAGCCCTCAAATTCAGTGGC
GTGCTCGCTAAGACTCCCGGGTGTAGTAATCTCCTTCTCGCCCGGGCTGGCTCAGCGGTG
CACTCGCCGTAAAACCCGCCATCGCGCACCTTCTGAAAGTTGACCTCGGATCAGGTAGG
AATACCCGCTGAACTTAAGCATATCAATAAGCGGAGGAAAAGAAACCAACAGGGATTG
CCCTAGTAACGGCGAGTGAAGCGGCAACAGCTCAAATTTGAAATCTGGCCCCCCCGGGG
CCCGAGTTGTAATTTGCAGAGGATGTCTTCTGGCGCGGTGCCTTCCGAGTTCCCTGGAAC
GGGACGCCACAGAGGGTGAGAGCCCCGTAGCGGTCGGATACCAAGCCTGTGTGAAGCT
CCTTCGACGAGTCGAGTAGTTTGGGAATGCTGCTCTAAATGGGAGGTAAATCTCTTCTAA
AGCTAAATACTGGCCAGAGACCGATAGCGCACAAGTAGAGTGATCGAAAGATGAAAAG
CACCTTGAAAAGGGGGTTAAACAGTACGTGAAATTGTTGAAAGGGAAGCGCTCATGACC
AGACTTGCGCCGGGCGGCTCAGCAGGGGTTCTGCCCCTGTGTACTCCGTCCCGGTTCAG
GCCAGCATCGGTTCCCGCTGGGGGATAAGAACGGTAGGAACGTGGCCCCCCCTCGGGGG
GGTGTTATAGCCCGCCGTCACGATACCCTGGCGGGGACCGAGGTTCGCGCACTCCGCAA
GGATGCTGGCGTAATGGTTATCAGTGACCCGTCTTGAAACACGGACCAAGGAGTCGTCC
TTTAGAGCGAGCGTCTGGGTGTCAAAACCCGCACGCGTAATGAAAGTGAAATTAGGTGA
GAGCCCTCACGGGCGCATCATCGACCGATCCTGATGTTCTCGGACGGATTTGAGTAAGA
GTTTTAACGGACGGACCCGAAAGACAGTGAACTATGCTTGTATAGGGTGAAGCCAGAGG
AAACTCTGGTGGAGGCTCGCAGCGGTTCTGACGTGCAAATCGATCGTCAAATATGAGCA
TGGGGGCGAAAGACTAATCGAACCACACCGCCCGTCGCTACTACCGATTGAATGGTTCA
GTGAGGCGTTCGGACTGGCCCAGGGAGGTCGGCAACGACCACCCAGGGCCGGAAAGTT
CTCCAAACTCGATCATTTAGAGGAAGTAAAAGTCGTAACAAGGTCTCCGTTGGTGAACC
AGCGGAGGGATCATTGATGGAAAACAATTCCTATACCCCTTTGTGAACTTATACCTATCT
CGTTGCCTCGGCGCCAGGCCCGGGGTTCCCCCCAGGGGGTTCCCCCACCAGGGACCTC
ACGGTCCCGGGTGGAGCAGGCCCGCCGGCGGCCCCCCTAAACTCTTGTTTTAGAACCT
ATCCCCTCTGAGTTTGTTACCTAAATGAATCAAAACTTTCAACAACGGATCTCTTGGCTC
TGGCATCGATGAAGAACGCAGCGAAATGCGATAAGTAATGTGAATTGCAGAATTCAGTG
AATCATCGAATCTTTGAACGCACATTGCGCCCGCTGGAATTCCGGCGGGCATGCCTGTTC
GAGCGTCATTTCAACCCCTCAAGCCCCCGTGCTTGGTGTTGGGGCATAGCCCGTAGAAA

GGCTAGCCCTCAAATTCAGTGGCGTGCTCGCTAAGACTCCCGGGTGTAGTAATCTCCTTC
TCGCCCGGGCTGGCTCAGCGGTGCACTCGCCGTAAAACCCGCCATCGCGCACCTTCTGA
AAGTTGACCTCGGATCAGGTAGGAATACCCGCTGAACTTAAGCATATCAATAAGCGGAG
GAAAAGAAACCAACAGGGATTGCCCTAGTAACGGCGAGTGAAGCGGCAACAGCTCAAA
TTTGAAATCTGGCCCCCCCGGGGCCCGAGTTGTAATTTGCAGAGGATGTCTTCTGGCGCG
GTGCCTTCCGAGTTCCCTGGAACGGGACGCCACAGAGGGTGAGAGCCCCGTAGCGGTCG
GATACCAAGCCTGTGTGAAGCTCCTTCGACGAGTCGAGTAGTTTGGGAATGCTGCTCTA
AATGGGAGGTAAATCTCTTCTAAAGCTAAATACTGGCCAGAGACCGATAGCGCACAAGT
AGAGTGATCGAAAGATGAAAAGCACCTTGAAAAGGGGGTTAAACAGTACGTGAAATTG
TTGAAAGGGAAGCGCTCATGACCAGACTTGCGCCGGGCGGCTCAGCAGGGGTTCTGCCC
CTGTGTACTCCGTCCCGGTTCAGGCCAGCATCGGTTCCCGCTGGGGGATAAGAACGGTA
GGAACGTGGCCCCCCCTCGGGGGGGTGTTATAGCCCGCCGTCACGATACCCTGGCGGGG
ACCGAGGTTCGCGCACTCCGCAAGGATGCTGGCGTAATGGTTATCAGTGACCCGTCTTG
AAACACGGACCAAGGAGTCGTCCTTTAGAGCGAGCGTCTGGGTGTCAAAACCCGCACGC
GTAATGAAAGTGAAATTAGGTGAGAGCCCTCACGGGCGCATCATCGACCGATCCTGATG
TTCTCGGACGGATTTGAGTAAGAGTTTTAACGGACGGACCCGAAAGACAGTGAACTATG
CTTGTATAGGGTGAAGCCAGAGGAAACTCTGGTGGAGGCTCGCAGCGGTTCTGACGTGC
AAATCGATCGTCAAATATGAGCATGGGGGCGAAAGACTAATCGAACGGTCCGCTCCTGG
CAAAGCTGTTCCGAAATATCGTCAAGAAGCTGACCGGCGACATGATGGCGTACATGAAG
CGTTGCGTCAGCGAGGGTAAGCATTTCGATCTGGCGTTGGGCATCCGCCACTCGACCCTC
ACAAATGGCCTCAAGTACTCTCTTGCGACTGGAAATTGGGGCGAGCAGAAGAAGGCGTC
GAGTTCCACCGCTGGTGTCTCGCAGGTGCTGAACCGGTACACGTTCTCGTCCACCCTATC
GCATTTGCGGCGCACGAACACGCCCATCGGCCGTGACGGCAAGCTGGCCAAGCCTCGTC
AGCTGCACAATACACACTGGGGTCTCGTCTGTCCAGCAGAGACTCCCGAGGGACAGGCT
TGCGGTCTCGTCAAGAACCTGTCTTTAATGTGCTACGTTAGTGTTGGTTCACCGGCTGAG
CCTATCAAGGACTTCATGGTGCAGCGTAATATGGAAGTCTTGGAGGAGTACGAGCCCGG
CTCCAGCCCTGACGCAACCAAGATCTTCATCAACGGCACATGGGTTGGTGTGCACAGTG
AGCCAGCGCACTTGGTCACGCTTGTGCAGGAGCTTCGGCGCAAATGCATCATTTCTCAC
GAAGTCTCCCTCGTTCGCGACATTCGCGACCGCGAGTTCAAGATCTTCTCCGACGCTGGT
CGTGTCATGCGGCCTCTCTTCGTTATCAATCAAGAGGATAATCCCGAGACTGGAATGCA
GCAGGGCACTCTGGCTCTCACCAAGCACCACATTCGCCGTCTCGAGGAAGATGCTCAGT
ACCATCGCAAGAAGCATGATGACGACTATTTCGGCTGGGATGGTCTTCAGAACAGCGGT
TGCATCGAGTACCTGGATGCCGAGGAAGAGGAGACAGCAATGATCTGCATGAGTCCTGA
GGATCTTGAGGACTACCGCCAGCGCAAGGCTAGGGGCAAGGATGCCAAGGATGATGAA
CCGGAGGACGACGGCAGGAGTCTAAACGCTCGGGTGAAGACGAAGATCAACCCTGACA
TCCACATGTACACCCACTGCGAGATCCATCCGAGCATGCTCCTGGGAATATGCGCCAGC
ATCATTCCCTTCCCGGATCACAATCAGGTGTGTAAAATTCTCGCAGTACTAACATTACTC
CTGCTGACCACATATATCTAGTCACCTCGTCCATCAAATCAGCATCATCACAGTCGCGCA
AACAAGGCCAAGCCAAAATGGTCGCCATCTCTTGGTCGCGGCCGTTTGCTCTGGCCCGC
GGGAGGGTGTCCTTTTTTCGCCCACGCACATTTTCGCTTTGTGGTGTGCGGGGTTTTGCG
TCTGGGTCTTATCTCAGATAAACGTGACCCACCCAACCGCCACCTCGACCATCCTCCGCC
AACACACCCCTTGCGCGCCGCTCTTTTTCACCCTCAAATCTTGATAATGACAATGCTGAC
TCGGATTCTCTACTGCAGCTGCTGAGCTCGGCAAGGGTTCCTTCAAGTACGCATGGGTCC
TTGACAAGCTCAAGGCCGAGCGTGAGCGTGGTATCACCATCGACATTGCTCTGTGGAAG
TTCGAGACTCCCAAGTACTATGTCACCGTCATTGGTAAGCTCTACCCCCTGGTGATGTGC
GCAGGCCTGGCTGTCCCGCCTCAGTGCGCTGCCTCGCCATGCGCGACATCCTGGCTTCTG
ATGCTTTTGAGCAATCCCATGCTAACGCAAATTCATCACAGACGCTCCCGGTCACCGTGA

CTTCATCAAGAACATGATCACTGGTACCTCCCAGGCTGACTGCGCCATTCTCATCATTGC
CTCCGGTACTGGTGAGTTCGAGGCTGGTATCTCCAAGGATGGCCAGACTCGTGAGCACG
CTCTGCTCGCCTACACCCTCGGTGTCAAGCAGCTCATTGTTGCTTGCAACAAGATGGACA
CCGCTGGCTGGAAGCAGGACCGTTTCGAGGAGATCAAGAAGGAGACCACCAACTTCATC
AAGAAGGTCGGCTTCAACCCCAAGCAGGTCGCTTTCGTCCCCATCTCCGGCTTCCACGGC
GACAACATGCTTGAGGCCTCCGCCAACATGCCCTGGTACAAGGGCTGGACCAAGGAGAC
CCAGGACAAGAAGGAGGCCAAGGGTATGACCCTGCTCGACGCCATTGATGCCATCGAG
CCCCCCAAGCGCCCCACCGACAAGCCCCTGCGTCTGCCCCTCCAGGATGTCTACAAGAT
CGGCGGTATCGGAACTGTACCTGTCGGCCGTATCGAGACTGGTATCCTGAAGCCCGGTA
TGGTGGTCACCTTCGCCCCCTCTAACGTCACCACTGAGGTCAAGTCCGTGGAGATGCACC
ACGAGCAGCTCACCGAGGGCCAGCCCGGTGACAACGTTGGTTTCAACGTCAAGAACGTC
TCCGTCAAGGAGATTCGCCGTGGCAACGTCGCTGGTGACTCCAAGAACGACCCCCCTAT
GGCCGCCGCTTCCTTCAACGCCCAGGTCATCGTCCTGAACCACCCCGGCCAGGTCGGTG
C

>Melanconiella_meridionalis

CACACCGCCCGTCGCTACTACCGATTGAATGGTTCAGTGAGGCGTTCGGACTGGCCCAG
GGAGGTCGGCAACGACCACCCAGGGCCGGAAAGTTCTCCAAACTCGATCATTTAGAGGA
AGTAAAAGTCGTAACAAGGTCTCCGTTGGTGAACCAGCGGAGGGATCATTGATGGAAA
ACAATTCCTATACCCCTTTGTGAACTTATACCTATCTCGTTGCCTCGGCGCCAGGCCCGG
GGTTCCCCCCAGGGGGTTCCCCCCACCAGGGACCTCACGGTCCCGGGTGGAGCAGGCCC
GCCGGCGGCCCCCCTAAACTCTTGTTTTTAGAACCTATCCCCTCTGAGTTTGTTACCTAA
ATGAATCAAAACTTTCAACAACGGATCTCTTGGCTCTGGCATCGATGAAGAACGCAGCG
AAATGCGATAAGTAATGTGAATTGCAGAATTCAGTGAATCATCGAATCTTTGAACGCAC
ATTGCGCCCGCTGGAATTCCGGCGGGCATGCCTGTTCGAGCGTCATTTCAACCCCTCAAG
CCCCCGTGCTTGGTGTTGGGGCATAGCCCGTAGAAAGGCTAGCCCTCAAATTCAGTGGC
GTGCTCGCTAAGACTCCCGGGTGTAGTAATCTCCTTCTCGCCCGGGCTGGCTCAGCGGTG
CACTCGCCGTAAAACCCGCCATCGCGCACCTTCTGAAAGTTGACCTCGGATCAGGTAGG
AATACCCGCTGAACTTAAGCATATCAATAAGCGGAGGAAAAGAAACCAACAGGGATTG
CCCTAGTAACGGCGAGTGAAGCGGCAACAGCTCAAATTTGAAATCTGGCCCCCCCGGGG
CCCGAGTTGTAATTTGCAGAGGATGTCTTCTGGCGCGGTGCCTTCCGAGTTCCCTGGAAC
GGGACGCCACAGAGGGTGAGAGCCCCGTAGCGGTCGGATACCAAGCCTGTGTGAAGCT
CCTTCGACGAGTCGAGTAGTTTGGGAATGCTGCTCTAAATGGGAGGTAAATCTCTTCTAA
AGCTAAATACTGGCCAGAGACCGATAGCGCACAAGTAGAGTGATCGAAAGATGAAAAG
CACCTTGAAAAGGGGGTTAAACAGTACGTGAAATTGTTGAAAGGGAAGCGCTCATGACC
AGACTTGCGCCGGGCGGCTCAGCAGGGGTTCTGCCCCTGTGTACTCCGTCCCGGTTCAG
GCCAGCATCGGTTCCCGCTGGGGGATAAGAACGGTAGGAACGTGGCCCCCCCTCGGGGG
GGTGTTATAGCCCGCCGTCACGATACCCTGGCGGGGACCGAGGTTCGCGCACTCCGCAA
GGATGCTGGCGTAATGGTTATCAGTGACCCGTCTTGAAACACGGACCAAGGAGTCGTCC
TTTAGAGCGAGCGTCTGGGTGTCAAAACCCGCACGCGTAATGAAAGTGAAATTAGGTGA
GAGCCCTCACGGGCGCATCATCGACCGATCCTGATGTTCTCGGACGGATTTGAGTAAGA
GTTTTAACGGACGGACCCGAAAGACAGTGAACTATGCTTGTATAGGGTGAAGCCAGAGG
AAACTCTGGTGGAGGCTCGCAGCGGTTCTGACGTGCAAATCGATCGTCAAATATGAGCA
TGGGGGCGAAAGACTAATCGAACCACACCGCCCGTCGCTACTACCGATTGAATGGTTCA
GTGAGGCGTTCGGACTGGCCCAGGGAGGTCGGCAACGACCACCCAGGGCCGGAAAGTT
CTCCAAACTCGATCATTTAGAGGAAGTAAAAGTCGTAACAAGGTCTCCGTTGGTGAACC
AGCGGAGGGATCATTGATGGAAAACAATTCCTATACCCCTTTGTGAACTTATACCTATCT

CGTTGCCTCGGCGCCAGGCCCGGGGTTCCCCCAGGGGGTTCCCCCACCAGGGACCTC
ACGGTCCCGGGTGGAGCAGGCCCGCCGGCGGCCCCCCTAAACTCTTGTTTTAGAACCT
ATCCCCTCTGAGTTTGTTACCTAAATGAATCAAAACTTTCAACAACGGATCTCTTGGCTC
TGGCATCGATGAAGAACGCAGCGAAATGCGATAAGTAATGTGAATTGCAGAATTCAGTG
AATCATCGAATCTTTGAACGCACATTGCGCCCGCTGGAATTCCGGCGGGCATGCCTGTTC
GAGCGTCATTTCAACCCCTCAAGCCCCCGTGCTTGGTGTTGGGGCATAGCCCGTAGAAA
GGCTAGCCCTCAAATTCAGTGGCGTGCTCGCTAAGACTCCCGGGTGTAGTAATCTCCTTC
TCGCCCGGGCTGGCTCAGCGGTGCACTCGCCGTAAAACCCGCCATCGCGCACCTTCTGA
AAGTTGACCTCGGATCAGGTAGGAATACCCGCTGAACTTAAGCATATCAATAAGCGGAG
GAAAAGAAACCAACAGGGATTGCCCTAGTAACGGCGAGTGAAGCGGCAACAGCTCAAA
TTTGAAATCTGGCCCCCCCGGGGCCCGAGTTGTAATTTGCAGAGGATGTCTTCTGGCGCG
GTGCCTTCCGAGTTCCCTGGAACGGGACGCCACAGAGGGTGAGAGCCCCGTAGCGGTCG
GATACCAAGCCTGTGTGAAGCTCCTTCGACGAGTCGAGTAGTTTGGGAATGCTGCTCTA
AATGGGAGGTAAATCTCTTCTAAAGCTAAATACTGGCCAGAGACCGATAGCGCACAAGT
AGAGTGATCGAAAGATGAAAAGCACCTTGAAAAGGGGGTTAAACAGTACGTGAAATTG
TTGAAAGGGAAGCGCTCATGACCAGACTTGCGCCGGGCGGCTCAGCAGGGGTTCTGCCC
CTGTGTACTCCGTCCCGGTTCAGGCCAGCATCGGTTCCCGCTGGGGGATAAGAACGGTA
GGAACGTGGCCCCCCCTCGGGGGGGTGTTATAGCCCGCCGTCACGATACCCTGGCGGGG
ACCGAGGTTCGCGCACTCCGCAAGGATGCTGGCGTAATGGTTATCAGTGACCCGTCTTG
AAACACGGACCAAGGAGTCGTCCTTTAGAGCGAGCGTCTGGGTGTCAAAACCCGCACGC
GTAATGAAAGTGAAATTAGGTGAGAGCCCTCACGGGCGCATCATCGACCGATCCTGATG
TTCTCGGACGGATTTGAGTAAGAGTTTTAACGGACGGACCCGAAAGACAGTGAACTATG
CTTGTATAGGGTGAAGCCAGAGGAAACTCTGGTGGAGGCTCGCAGCGGTTCTGACGTGC
AAATCGATCGTCAAATATGAGCATGGGGGCGAAAGACTAATCGAACGGTCCGCTCCTGG
CAAAGCTGTTCCGAAATATCGTCAAGAAGCTGACCGGCGACATGATGGCGTACATGAAG
CGTTGCGTCAGCGAGGGTAAGCATTTCGATCTGGCGTTGGGCATCCGCCACTCGACCCTC
ACAAATGGCCTCAAGTACTCTCTTGCGACTGGAAATTGGGGCGAGCAGAAGAAGGCGTC
GAGTTCCACCGCTGGTGTCTCGCAGGTGCTGAACCGGTACACGTTCTCGTCCACCCTATC
GCATTTGCGGCGCACGAACACGCCCATCGGCCGTGACGGCAAGCTGGCCAAGCCTCGTC
AGCTGCACAATACACACTGGGGTCTCGTCTGTCCAGCAGAGACTCCCGAGGGACAGGCT
TGCGGTCTCGTCAAGAACCTGTCTTTAATGTGCTACGTTAGTGTTGGTTCACCGGCTGAG
CCTATCAAGGACTTCATGGTGCAGCGTAATATGGAAGTCTTGGAGGAGTACGAGCCCGG
CTCCAGCCCTGACGCAACCAAGATCTTCATCAACGGCACATGGGTTGGTGTGCACAGTG
AGCCAGCGCACTTGGTCACGCTTGTGCAGGAGCTTCGGCGCAAATGCATCATTTCTCAC
GAAGTCTCCCTCGTTCGCGACATTCGCGACCGCGAGTTCAAGATCTTCTCCGACGCTGGT
CGTGTCATGCGGCCTCTCTTCGTTATCAATCAAGAGGATAATCCCGAGACTGGAATGCA
GCAGGGCACTCTGGCTCTCACCAAGCACCACATTCGCCGTCTCGAGGAAGATGCTCAGT
ACCATCGCAAGAAGCATGATGACGACTATTTCGGCTGGGATGGTCTTCAGAACAGCGGT
TGCATCGAGTACCTGGATGCCGAGGAAGAGGAGACAGCAATGATCTGCATGAGTCCTGA
GGATCTTGAGGACTACCGCCAGCGCAAGGCTAGGGGCAAGGATGCCAAGGATGATGAA
CCGGAGGACGACGGCAGGAGTCTAAACGCTCGGGTGAAGACGAAGATCAACCCTGACA
TCCACATGTACACCCACTGCGAGATCCATCCGAGCATGCTCCTGGGAATATGCGCCAGC
ATCATTCCCTTCCCGGATCACAATCAGGTGTGTAAAATTCTCGCAGTACTAACATTACTC
CTGCTGACCACATATATCTAGTCACCTCGCATCCATCAAATCAGCATCATCACAGTCGCG
CAAACAAGGCCAAGCCAAAATGGTCGCCATCTCTTGGTCGCGGCCGTTTGCTCTGGCCC
GCGGGAGGGTGTCCTTTTTCGCCCACGCACATTTTCGCTTTGTGGTGTGCGGGGTTTTG
CGTCTGGGTCTTATCTCAGATAAACGTGACCCACCCAACCGCCACCTCGACCATCCTCCG

CCAACACACCCCTTGCGCGCCGCTCTTTTTCACCCTCAAATCTTGATAATGACAATGCTG
ACTCGGATTCTCTACTGCAGCTGCTGAGCTCGGCAAGGGTTCCTTCAAGTACGCATGGGT
CCTTGACAAGCTCAAGGCCGAGCGTGAGCGTGGTATCACCATCGACATTGCTCTGTGGA
AGTTCGAGACTCCCAAGTACTATGTCACCGTCATTGGTAAGCTCTACCCCCTGGTGATGT
GCGCAGGCCTGGCTGTCCCGCCTCAGTGCGCTGCCTCGCCATGCGCGACATCCTGGCTTC
TGATGCTTTTGAGCAATCCCATGCTAACGCAAATTCATCACAGACGCTCCCGGTCACCGT
GACTTCATCAAGAACATGATCACTGGTACCTCCCAGGCTGACTGCGCCATTCTCATCATT
GCCTCCGGTACTGGTGAGTTCGAGGCTGGTATCTCCAAGGATGGCCAGACTCGTGAGCA
CGCTCTGCTCGCCTACACCCTCGGTGTCAAGCAGCTCATTGTTGCTTGCAACAAGATGGA
CACCGCTGGCTGGAAGCAGGACCGTTTCGAGGAGATCAAGAAGGAGACCACCAACTTC
ATCAAGAAGGTCGGCTTCAACCCCAAGCAGGTCGCTTTCGTCCCCATCTCCGGCTTCCAC
GGCGACAACATGCTTGAGGCCTCCGCCAACATGCCCTGGTACAAGGGCTGGACCAAGGA
GACCCAGGACAAGAAGGAGGCCAAGGGTATGACCCTGCTCGACGCCATTGATGCCATC
GAGCCCCCCAAGCGCCCCACCGACAAGCCCCTGCGTCTGCCCCTCCAGGATGTCTACAA
GATCGGCGGTATCGGAACTGTACCTGTCGGCCGTATCGAGACTGGTATCCTGAAGCCCG
GTATGGTGGTCACCTTCGCCCCCTCTAACGTCACCACTGAGGTCAAGTCCGTGGAGATGC
ACCACGAGCAGCTCACCGAGGGCCAGCCCGGTGACAACGTTGGTTTCAACGTCAAGAAC
GTCTCCGTCAAGGAGATTCGCCGTGGCAACGTCGCTGGTGACTCCAAGAACGACCCCCC
TATGGCCGCCGCTTCCTTCAACGCCCAGGTCATCGTCCTGAACCACCCCGGCCAGGTCGG
TGCTGGATAC

>Melanconiella_meridionalis

CACACCGCCCGTCGCTACTACCGATTGAATGGTTCAGTGAGGCGTTCGGACTGGCCCAG
GGAGGTCGGCAACGACCACCCAGGGCCGGAAAGTTCTCCAAACTCGATCATTTAGAGGA
AGTAAAAGTCGTAACAAGGTCTCCGTTGGTGAACCAGCGGAGGGATCATTGATGGAAA
ACAATTCCTATACCCCTTTGTGAACTTATACCTATCTCGTTGCCTCGGCGCCAGGCCCGG
GGTTCCCCCCAGGGGGTTCCCCCCACCAGGGACCTCACGGTCCCGGGTGGAGCAGGCCC
GCCGGCGGCCCCCCTAAACTCTTGTTTTTAGAACCTATCCCCTCTGAGTTTGTTACCTAA
ATGAATCAAAACTTTCAACAACGGATCTCTTGGCTCTGGCATCGATGAAGAACGCAGCG
AAATGCGATAAGTAATGTGAATTGCAGAATTCAGTGAATCATCGAATCTTTGAACGCAC
ATTGCGCCCGCTGGAATTCCGGCGGGCATGCCTGTTCGAGCGTCATTTCAACCCCTCAAG
CCCCCGTGCTTGGTGTTGGGGCATAGCCCGTAGAAAGGCTAGCCCTCAAATTCAGTGGC
GTGCTCGCTAAGACTCCCGGGTGTAGTAATCTCCTTCTCGCCCGGGCTGGCTCAGCGGTG
CACTCGCCGTAAAACCCGCCATCGCGCACCTTCTGAAAGTTGACCTCGGATCAGGTAGG
AATACCCGCTGAACTTAAGCATATCAATAAGCGGAGGAAAAGAAACCAACAGGGATTG
CCCTAGTAACGGCGAGTGAAGCGGCAACAGCTCAAATTTGAAATCTGGCCCCCCCGGGG
CCCGAGTTGTAATTTGCAGAGGATGTCTTCTGGCGCGGTGCCTTCCGAGTTCCCTGGAAC
GGGACGCCACAGAGGGTGAGAGCCCCGTAGCGGTCGGATACCAAGCCTGTGTGAAGCT
CCTTCGACGAGTCGAGTAGTTTGGGAATGCTGCTCTAAATGGGAGGTAAATCTCTTCTAA
AGCTAAATACTGGCCAGAGACCGATAGCGCACAAGTAGAGTGATCGAAAGATGAAAAG
CACCTTGAAAAGGGGGTTAAACAGTACGTGAAATTGTTGAAAGGGAAGCGCTCATGACC
AGACTTGCGCCGGGCGGCTCAGCAGGGGTTCTGCCCCTGTGTACTCCGTCCCGGTTCAG
GCCAGCATCGGTTCCCGCTGGGGGATAAGAACGGTAGGAACGTGGCCCCCCCTCGGGGG
GGTGTTATAGCCCGCCGTCACGATACCCTGGCGGGGACCGAGGTTCGCGCACTCCGCAA
GGATGCTGGCGTAATGGTTATCAGTGACCCGTCTTGAAACACGGACCAAGGAGTCGTCC
TTTAGAGCGAGCGTCTGGGTGTCAAAACCCGCACGCGTAATGAAAGTGAAATTAGGTGA
GAGCCCTCACGGGCGCATCATCGACCGATCCTGATGTTCTCGGACGGATTTGAGTAAGA

GTTTTAACGGACGGACCCGAAAGACAGTGAACTATGCTTGTATAGGGTGAAGCCAGAGG
AAACTCTGGTGGAGGCTCGCAGCGGTTCTGACGTGCAAATCGATCGTCAAATATGAGCA
TGGGGGCGAAAGACTAATCGAACCACACCGCCCGTCGCTACTACCGATTGAATGGTTCA
GTGAGGCGTTCGGACTGGCCCAGGGAGGTCGGCAACGACCACCCAGGGCCGGAAAGTT
CTCCAAACTCGATCATTTAGAGGAAGTAAAAGTCGTAACAAGGTCTCCGTTGGTGAACC
AGCGGAGGGATCATTGATGGAAAACAATTCCTATACCCCTTTGTGAACTTATACCTATCT
CGTTGCCTCGGCGCCAGGCCCGGGGTTCCCCCAGGGGGTTCCCCCACCAGGGACCTC
ACGGTCCCGGGTGGAGCAGGCCCGCCGGCGGCCCCCCTAAACTCTTGTTTTTAGAACCT
ATCCCCTCTGAGTTTGTTACCTAAATGAATCAAAACTTTCAACAACGGATCTCTTGGCTC
TGGCATCGATGAAGAACGCAGCGAAATGCGATAAGTAATGTGAATTGCAGAATTCAGTG
AATCATCGAATCTTTGAACGCACATTGCGCCCGCTGGAATTCCGGCGGGCATGCCTGTTC
GAGCGTCATTTCAACCCCTCAAGCCCCCGTGCTTGGTGTTGGGGCATAGCCCGTAGAAA
GGCTAGCCCTCAAATTCAGTGGCGTGCTCGCTAAGACTCCCGGGTGTAGTAATCTCCTTC
TCGCCCGGGCTGGCTCAGCGGTGCACTCGCCGTAAAACCCGCCATCGCGCACCTTCTGA
AAGTTGACCTCGGATCAGGTAGGAATACCCGCTGAACTTAAGCATATCAATAAGCGGAG
GAAAAGAAACCAACAGGGATTGCCCTAGTAACGGCGAGTGAAGCGGCAACAGCTCAAA
TTTGAAATCTGGCCCCCCCGGGGCCCGAGTTGTAATTTGCAGAGGATGTCTTCTGGCGCG
GTGCCTTCCGAGTTCCCTGGAACGGGACGCCACAGAGGGTGAGAGCCCCGTAGCGGTCG
GATACCAAGCCTGTGTGAAGCTCCTTCGACGAGTCGAGTAGTTTGGGAATGCTGCTCTA
AATGGGAGGTAAATCTCTTCTAAAGCTAAATACTGGCCAGAGACCGATAGCGCACAAGT
AGAGTGATCGAAAGATGAAAAGCACCTTGAAAAGGGGGTTAAACAGTACGTGAAATTG
TTGAAAGGGAAGCGCTCATGACCAGACTTGCGCCGGGCGGCTCAGCAGGGGTTCTGCCC
CTGTGTACTCCGTCCCGGTTCAGGCCAGCATCGGTTCCCGCTGGGGGATAAGAACGGTA
GGAACGTGGCCCCCCCTCGGGGGGGTGTTATAGCCCGCCGTCACGATACCCTGGCGGGG
ACCGAGGTTCGCGCACTCCGCAAGGATGCTGGCGTAATGGTTATCAGTGACCCGTCTTG
AAACACGGACCAAGGAGTCGTCCTTTAGAGCGAGCGTCTGGGTGTCAAAACCCGCACGC
GTAATGAAAGTGAAATTAGGTGAGAGCCCTCACGGGCGCATCATCGACCGATCCTGATG
TTCTCGGACGGATTTGAGTAAGAGTTTTAACGGACGGACCCGAAAGACAGTGAACTATG
CTTGTATAGGGTGAAGCCAGAGGAAACTCTGGTGGAGGCTCGCAGCGGTTCTGACGTGC
AAATCGATCGTCAAATATGAGCATGGGGGCGAAAGACTAATCGAACGGTCCGCTCCTGG
CAAAGCTGTTCCGAAATATCGTCAAGAAGCTGACCGGCGACATGATGGCGTACATGAAG
CGTTGCGTCAGCGAGGGTAAGCATTTCGATCTGGCGTTGGGCATCCGCCACTCGACCCTC
ACAAATGGCCTCAAGTACTCTCTTGCGACTGGAAATTGGGGCGAGCAGAAGAAGGCGTC
GAGTTCCACCGCTGGTGTCTCGCAGGTGCTGAACCGGTACACGTTCTCGTCCACCCTATC
GCATTTGCGGCGCACGAACACGCCCATCGGCCGTGACGGCAAGCTGGCCAAGCCTCGTC
AGCTGCACAATACACACTGGGGTCTCGTCTGTCCAGCAGAGACTCCCGAGGGACAGGCT
TGCGGTCTCGTCAAGAACCTGTCTTTAATGTGCTACGTTAGTGTTGGTTCACCGGCTGAG
CCTATCAAGGACTTCATGGTGCAGCGTAATATGGAAGTCTTGGAGGAGTACGAGCCCGG
CTCCAGCCCTGACGCAACCAAGATCTTCATCAACGGCACATGGGTTGGTGTGCACAGTG
AGCCAGCGCACTTGGTCACGCTTGTGCAGGAGCTTCGGCGCAAATGCATCATTTCTCAC
GAAGTCTCCCTCGTTCGCGACATTCGCGACCGCGAGTTCAAGATCTTCTCCGACGCTGGT
CGTGTCATGCGGCCTCTCTTCGTTATCAATCAAGAGGATAATCCCGAGACTGGAATGCA
GCAGGGCACTCTGGCTCTCACCAAGCACCACATTCGCCGTCTCGAGGAAGATGCTCAGT
ACCATCGCAAGAAGCATGATGACGACTATTTCGGCTGGGATGGTCTTCAGAACAGCGGT
TGCATCGAGTACCTGGATGCCGAGGAAGAGGAGACAGCAATGATCTGCATGAGTCCTGA
GGATCTTGAGGACTACCGCCAGCGCAAGGCTAGGGGCAAGGATGCCAAGGATGATGAA
CCGGAGGACGACGGCAGGAGTCTAAACGCTCGGGTGAAGACGAAGATCAACCCTGACA

TCCACATGTACACCCACTGCGAGATCCATCCGAGCATGCTCCTGGGAATATGCGCCAGC
ATCATTCCCTTCCCGGATCACAATCAGGTGTGTAAAATTCTCGCAGTACTAACATTACTC
CTGCTGACCACATATATCTAGTCACCTCGCATCCATCAAATCAGCATCATCACAGTCGCG
CAAACAAGGCCAAGCCAAAATGGTCGCCATCTCTTGGTCGCGGCCGTTTGCTCTGGCCC
GCGGGAGGGTGTCCTTTTTTCGCCCACGCACATTTTCGCTTTGTGGTGTGCGGGGTTTTG
CGTCTGGGTCTTATCTCAGATAAACGTGACCCACCCAACCGCCACCTCGACCATCCTCCG
CCAACACACCCCTTGCGCGCCGCTCTTTTTCACCCTCAAATCTTGATAATGACAATGCTG
ACTCGGATTCTCTACTGCAGCTGCTGAGCTCGGCAAGGGTTCCTTCAAGTACGCATGGGT
CCTTGACAAGCTCAAGGCCGAGCGTGAGCGTGGTATCACCATCGACATTGCTCTGTGGA
AGTTCGAGACTCCCAAGTACTATGTCACCGTCATTGGTAAGCTCTACCCCCTGGTGATGT
GCGCAGGCCTGGCTGTCCCGCCTCAGTGCGCTGCCTCGCCATGCGCGACATCCTGGCTTC
TGATGCTTTTGAGCAATCCCATGCTAACGCAAATTCATCACAGACGCTCCCGGTCACCGT
GACTTCATCAAGAACATGATCACTGGTACCTCCCAGGCTGACTGCGCCATTCTCATCATT
GCCTCCGGTACTGGTGAGTTCGAGGCTGGTATCTCCAAGGATGGCCAGACTCGTGAGCA
CGCTCTGCTCGCCTACACCCTCGGTGTCAAGCAGCTCATTGTTGCTTGCAACAAGATGGA
CACCGCTGGCTGGAAGCAGGACCGTTTCGAGGAGATCAAGAAGGAGACCACCAACTTC
ATCAAGAAGGTCGGCTTCAACCCCAAGCAGGTCGCTTTCGTCCCCATCTCCGGCTTCCAC
GGCGACAACATGCTTGAGGCCTCCGCCAACATGCCCTGGTACAAGGGCTGGACCAAGGA
GACCCAGGACAAGAAGGAGGCCAAGGGTATGACCCTGCTCGACGCCATTGATGCCATC
GAGCCCCCCAAGCGCCCCACCGACAAGCCCCTGCGTCTGCCCCTCCAGGATGTCTACAA
GATCGGCGGTATCGGAACTGTACCTGTCGGCCGTATCGAGACTGGTATCCTGAAGCCCG
GTATGGTGGTCACCTTCGCCCCCTCTAACGTCACCACTGAGGTCAAGTCCGTGGAGATGC
ACCACGAGCAGCTCACCGAGGGCCAGCCCGGTGACAACGTTGGTTTCAACGTCAAGAAC
GTCTCCGTCAAGGAGATTCGCCGTGGCAACGTCGCTGGTGACTCCAAGAACGACCCCCC
TATGGCCGCCGCTTCCTTCAACGCCCAGGTCATCGTCCTGAACCACCCCGGCCAGGTCGG
TGCTGGATAC

>Melanconiella_ostryae

CACACCGCCCGTCGCTACTACCGATTGAATGGTTCAGTGAGGCGTTCGGACTGGCCCAG
GGAGGTCGGCAACGACCACCCAGGGCCGGAAAGTTCTCCAAACTCGATCATTTAGAGGA
AGTAAAAGTCGTAACAAGGTCTCCGTTGGTGAACCAGCGGAGGGATCATTGATGGAACA
CAATTCCTATACCCCTTTGTGAACTTATACCTATCTCGTTGCCTCGGCGCCAGGCCCGGG
GTTCCCCCCAGGGGGTTCCCCCAACCAGGGACCTCACGGTCCCCGGGTGGAGCAGGCC
CGCCGGCGGCCCCCCTAAACTCTTGTTTTCAGAACCTGTCTCCTCTGAGTTTGTTACCTA
AATGAATCAAAACTTTCAACAACGGATCTCTTGGCTCTGGCATCGATGAAGAACGCAGC
GAAATGCGATAAGTAATGTGAATTGCAGAATTCAGTGAATCATCGAATCTTTGAACGCA
CATTGCGCCCGCTGGAATTCCGGCGGGCATGCCTGTTCGAGCGTCATTTCAACCCCTCAA
GCCCCCGCGCTTGGTGTTGGGGCATAGCCTGTAGAAGGGCTAGCCCTCAAATTCAGTGG
CGTGCTCGCTAAGACTCCCGGGTGTAGTAATCTCCTTCTCGCCCGGGCTGGCTCAGCGGC
GCACTCGCCGTAAAACCCGCCATCGCGCACCTTCTGAAAGTTGACCTCGGATCAGGTAG
GAATACCCGCTGAACTTAAGCATATCAATAAGCGGAGGAAAAGAAACCAACAGGGATT
GCCCTAGTAACGGCGAGTGAAGCGGCAACAGCTCAAATTTGAAATCTGGCCCCCCCCGG
GGCCCGAGTTGTAATTTGCAGAGGATGTCTTCTGGCGCGGTGCCTTCCGAGTTCCCTGGA
ACGGGACGCCACAGAGGGTGAGAGCCCCGTAGCGGTCGGATACCAAGCCTGTGTGAAG
CTCCTTCGACGAGTCGAGTAGTTTGGGAATGCTGCTCTAAATGGGAGGTAAATCTCTTCT
AAAGCTAAATACTGGCCAGAGACCGATAGCGCACAAGTAGAGTGATCGAAAGATGAAA
AGCACCTTGAAAAGGGGGTTAAACAGTACGTGAAATTGTTGAAAGGGAAGCGCTCATG

ACCAGACTTGCGCCGGGCGGCTCAGCAGGGGTTCCGCCCCTGTGTATTCCGTCCCGGTTC
AGGCCAGCATCGGTTCTCGCTGGGGGATAAGAACGGTGGGAACGTGGCCCCCCCTCGGG
GGGGTGTTATAGCCCGCCGTCACGATATCCTGGCGGGGACCGAGGTTCGCGCACTCCGC
AAGGATGCTGGCGTAATGGTTATCAGTGACCCGTCTTGAAACACGGACCAAGGAGTCGT
CCTTTAGAGCGAGCGTCTGGGTGTCAAAACCCGCACGCGTAATGAAAGTGAAATTAGGT
GAGAGCCCTCGCGGGCGCATCATCGACCGATCCTGATGTTCTCGGACGGATTTGAGTAA
GAGTTCTAACGGACGGACCCGAAAGACAGTGAACTATGCTTGTATAGGGTGAAGCCAG
AGGAAACTCTGGTGGAGGCTCGCAGCGGTTCTGACGTGCAAATCGATCGTCAAATATGA
GCATGGGGGCGAAAGACTAATCGAACACACCGCCCGTCGCTACTACCGATTGAATGGTT
CAGTGAGGCGTTCGGACTGGCCCAGGGAGGTCGGCAACGACCACCCAGGGCCGGAAAG
TTCTCCAAACTCGATCATTTAGAGGAAGTAAAAGTCGTAACAAGGTCTCCGTTGGTGAA
CCAGCGGAGGGATCATTGATGGAACACAATTCCTATACCCCTTTGTGAACTTATACCTAT
CTCGTTGCCTCGGCGCCAGGCCCGGGGTTCCCCCCAGGGGGTTCCCCCCAACCAGGGAC
CTCACGGTCCCCGGGTGGAGCAGGCCCGCCGGCGGCCCCCCTAAACTCTTGTTTTCAGA
ACCTGTCTCCTCTGAGTTTGTTACCTAAATGAATCAAAACTTTCAACAACGGATCTCTTG
GCTCTGGCATCGATGAAGAACGCAGCGAAATGCGATAAGTAATGTGAATTGCAGAATTC
AGTGAATCATCGAATCTTTGAACGCACATTGCGCCCGCTGGAATTCCGGCGGGCATGCC
TGTTCGAGCGTCATTTCAACCCCTCAAGCCCCCGCGCTTGGTGTTGGGGCATAGCCTGTA
GAAGGGCTAGCCCTCAAATTCAGTGGCGTGCTCGCTAAGACTCCCGGGTGTAGTAATCT
CCTTCTCGCCCGGGCTGGCTCAGCGGCGCACTCGCCGTAAAACCCGCCATCGCGCACCTT
CTGAAAGTTGACCTCGGATCAGGTAGGAATACCCGCTGAACTTAAGCATATCAATAAGC
GGAGGAAAAGAAACCAACAGGGATTGCCCTAGTAACGGCGAGTGAAGCGGCAACAGCT
CAAATTTGAAATCTGGCCCCCCCCGGGGCCCGAGTTGTAATTTGCAGAGGATGTCTTCTG
GCGCGGTGCCTTCCGAGTTCCCTGGAACGGGACGCCACAGAGGGTGAGAGCCCCGTAGC
GGTCGGATACCAAGCCTGTGTGAAGCTCCTTCGACGAGTCGAGTAGTTTGGGAATGCTG
CTCTAAATGGGAGGTAAATCTCTTCTAAAGCTAAATACTGGCCAGAGACCGATAGCGCA
CAAGTAGAGTGATCGAAAGATGAAAAGCACCTTGAAAAGGGGGTTAAACAGTACGTGA
AATTGTTGAAAGGGAAGCGCTCATGACCAGACTTGCGCCGGGCGGCTCAGCAGGGGTTC
CGCCCCTGTGTATTCCGTCCCGGTTCAGGCCAGCATCGGTTCTCGCTGGGGGATAAGAAC
GGTGGGAACGTGGCCCCCCCTCGGGGGGGTGTTATAGCCCGCCGTCACGATATCCTGGC
GGGGACCGAGGTTCGCGCACTCCGCAAGGATGCTGGCGTAATGGTTATCAGTGACCCGT
CTTGAAACACGGACCAAGGAGTCGTCCTTTAGAGCGAGCGTCTGGGTGTCAAAACCCGC
ACGCGTAATGAAAGTGAAATTAGGTGAGAGCCCTCGCGGGCGCATCATCGACCGATCCT
GATGTTCTCGGACGGATTTGAGTAAGAGTTCTAACGGACGGACCCGAAAGACAGTGAAC
TATGCTTGTATAGGGTGAAGCCAGAGGAAACTCTGGTGGAGGCTCGCAGCGGTTCTGAC
GTGCAAATCGATCGTCAAATATGAGCATGGGGGCGAAAGACTAATCGAAGGTCCGCTCC
TGGCAAAGCTGTTCCGAAATATCGTCAAGAAGCTGACCGGCGACATGATGGCGTACATG
AAGCGTTGCGTCAGCGAGGGTAAGCATTTCGATCTGGCGTTGGGCATCCGCCACTCAAC
CCTCACAAATGGCCTCAAGTACTCTCTTGCGACTGGAAATTGGGGCGAGCAGAAGAAGG
CGATGAGTTCCACCGCTGGTGTCTCGCAGGTGTTGAACCGGTACACGTTCTCGTCCACCC
TGTCGCATTTGCGGCGCACAAACACGCCCATCGGTCGTGACGGCAAGCTGGCCAAGCCT
CGTCAGCTGCACAATACACACTGGGGTCTCGTCTGTCCAGCAGAGACTCCCGAGGGACA
GGCTTGTGGTCTCGTCAAGAACCTGTCTTTAATGTGCTACGTTAGCGTCGGTTCACCGGC
TGAGCCTATCAAGGACTTCATGGTGCAGCGTAATATGGAAGTCTTGGAGGAGTACGAGC
CCGGCTCCAGCCCTGACGCAACCAAGATCTTCATCAACGGCACATGGGTTGGCGTGCAC
AGTGAGCCAGCACACTTGGTCACGCTTGTGCAGGAGCTTCGGCGCAAATGCATCATTTC
TCACGAAGTCTCCCTCGTTCGCGACATTCGCGACCGCGAGTTCAAGATCTTCTCCGATGC

TGGTCGTGTCATGCGACCTCTCTTCGTTATCAATCAAGAGGATAATCCCGAGACTGGAAT
GCAGCAGGGCACTCTGGCTCTCACCAAGCACCACATTCGCCGTCTCGAAGAAGATGCTC
AGTACCATCGCAAGAAGCATGATGACGACTATTTCGGCTGGGATGGTCTTCAGAATAGT
GGTTGCATCGAGTACCTGGATGCCGAGGAAGAGGAGACAGCAATGATCTGCATGAGTCC
TGAGGATCTTGAGGACTACCGCCAGCGCAAGGCTAGGGGCAAGGATGCCAAGGATGAT
GAACTGGAGGACGATGGCAGGAGTCTGAACGCTCGTGTGAAGACGAAGATCAACCCTG
ACATCCACATGTACACCCACTGCGAGATCCATCCGAGCATGCTCCTGGGAATATGCGCC
AGCATCATTCCCTTCCCGGATCACAATCAGGTGTGTAAAATCCTCGCAGTACTAATATCA
CTCCTGCTGACCACATATATCTAGTCACCTCGTCAAATCAGCATCATCACAATTGCGCAA
ACATCACGGTCAAGCCAAAATGGTTGCCATCTCTGGTCGCGGCCGTTTGCCCTGGCCCGC
GGGAGGGTGTCCTTTTTTCGCCCACGCACGTTTTCGCTTTGTGGTGCGGGGTTTTGCGTCT
GGGTCTTATCTCAGATAAACGTGACCCACCCAACCGCCACCTAAATCATCCTGCGCCAA
CACCCCCTTGCGCGCCTCTCTTTTTCACCCTCAAGCTTTGACAGCGGCAATGCTGACTCG
GATTCTCTACTGCAGCTAATGAGCTCGGCAAGGGTTCCTTCAAGTACGCATGGGTTCTTG
ACAAGCTCAAGGCCGAGCGTGAGCGTGGTATCACCATCGACATTGCTCTGTGGAAGTTC
GAGACTCCCAAGTACTATGTCACCGTCATTGGTAAGCTCCACCCCCTGGTGATGTGCGCA
GGCCTGGCTGCCCCGCCTGACTGCGCTGCCTCGCCATGCGCGACACCGCGGCTTCTGGTG
CTTTTGCGCAACCCGTGCTAACGCAAATGTCACCACAGACGCTCCCGGTCACCGTGACTT
CATCAAGAACATGATCACTGGTACCTCCCAGGCTGACTGCGCCATTCTCATCATTGCCTC
CGGTACTGGTGAGTTCGAGGCTGGTATCTCCAAGGATGGCCAGACCCGTGAGCACGCCC
TGCTCGCCTACACCCTCGGTGTCAAGCAGCTCATTGTTGCTTGCAACAAGATGGACACCG
CTGGCTGGAAGCAGGACCGTTTTGAGGAGATCAAGAAGGAGACCACCAACTTCATCAA
GAAGGTCGGCTTCAACGCCAAGCAGGTCGCTTTCGTCCCCATCTCCGGCTTCCACGGCG
ACAACATGCTTGAGCCCTCCTGCAACATGCCCTGGTACAAGGGCTGGACTAAGGAGACC
CAGGACAAGAAGGAGGCCAAGGGTATTACCCTGCTCGATGCCATTGACTCCATCGAGCC
CCCCAAGCGCCCCACCGAGAAGCCCCTGCGTCTGCCCCTCCAGGATGTCTACAAGATCG
GCGGTATCGGAACTGTACCTGTCGGCCGTATCGAGACTGGTATCCTGAAGCCCGGTATG
GTCGTCACCTTCGCCCCCGCCAACGTCACCACTGAGGTCAAGTCCGTGGAGATGCACCA
CGAGCAGCTCACCGAGGGTCAGCCCGGTGACAACGTTGGTTTCAACGTCAAGAACGTCT
CCGTCAAGGAGATTCGCCGTGGCAACGTCGCTGGTGACTCCAAGAACGACCCCCCCATG
GGCGCCGCTTCCTTCAACGCCCAGGTCATCGTCCTGAACCACCCCGGCCAGGTCGGTGCT
GGATAC

>Melanconiella_carpinicola

CACACCGCCCGTCGCTACTACCGATTGAATGGTTCAGTGAGGCGTTCGGACTGGCCCAG
GGAGGTCGGCAACGACCACCCAGGGCCGGAAAGTTCTCCAAACTCGATCATTTAGAGGA
AGTAAAAGTCGTAACAAGGTCTCCGTTGGTGAACCAGCGGAGGGATCATTGCTGAATAT
CCGTATTCGCCACCCTTTGTGAATATATACCTATCTCGTTGCCTCGGCGCCAGGCCCGGG
GTTCCCCCCTAGGGGGTTCCCCCCACCTGTGGACCTCACGGTCCCGGGCGGAGCAGGCC
CGCCGGCGGCCCACCAAAACTCTTGTTTGTAGAACCTATCTCTCTGAGTCTCATACGTAA
AATGAATCAAAACTTTCAACAACGGATCTCTTGGCTCTGGCATCGATGAAGAACGCAGC
GAAATGCGATAAGTAATGTGAATTGCAGAATTCAGTGAATCATCGAATCTTTGAACGCA
CATTGCGCCCGCTGGAATTCCGGCGGGCATGCCTGTTCGAGCGTCATTTCAACCCCTCAA
GCTTCAGCGCTTGGTGTTGGGGCATAGCCCGTGAAAAGGCTAGCCCTTAAATTCAGTGG
CGTGCTCGCTGAGACCCCCGGGTGAAGTAATCTCCTTCTCGCCCGGGCCGGCTCGGCGG
TGCACTCGCCGTAAAACCCGCTATCACGCACCTTCTGAAAGTTGACCTCGGATCAGGTA
GGAATACCCGCTGAACTTAAGCATATCAATAAGCGGAGGAAAAGAAACCAACAGGGAT

TGCCCTAGTAACGGCGAGTGAAGCGGCAACAGCTCAAATTTGAAATCTGGCCCCCCGG
GGGTCCGAGTTGTAATTTGCAGAGGATGTCTTCTGGCGCGGTGCCTTCCGAGTTCCCTGG
AACGGGACGCCACAGAGGGTGAGAGCCCCGTAATGGTCGGATACCAAGCCTGTGTGAA
GCTCCTTCGACGAGTCGAGTAGTTTGGGAATGCTGCTCTAAATGGGAGGTAAATCTCTTC
TAAAGCTAAATATTGGCCAGAGACCGATAGCGCACAAGTAGAGTGATCGAAAGATGAA
AAGCACCTTGAAAAGGGGGTTAAACAGTACGTGAAATTGTTGAAAGGGAAGCGCTCAT
GACCAGACTTGCGCCGGGCGGCTCAGCAGGGGTTCCGCCCCTGTGTACTCCGTCCCGGT
TCAGGCCAGCATCGGTTCTCGCTGGGGGAAAAGAACGGCGGGAACGTGGCCCTCCCTCG
GGGGGGTGTTATAGCCCGTCGTCCGATACCCTGGCGGGGTCCGAGGTTCGCGCATTCGC
AAGGATGCTGGCGTAATGGTTATCAGTGACCCGTCTTGAAACACGGACCAAGGAGTCGT
CCTTTAGAGCGAGCGTCTGGGTGTTAAAACCCGCACGCGTAATGAAAGTGAAATTAGGT
GAGAGCCCTTACGGGCGCATCATCGACCGATCCTGATGTTCTCGGACGGATTTGAGTAA
GAGTTCTAACGGACGGACCCGAAAGACAGTGAACTATGCTTGTATAGGGTGAAGCCAG
AGGAAACTCTGGTGGAGGCTCGCAGCGGTTCTGACGTGCAAATCGATCGTCAAATATGA
GCATGGGGGCGAAAGACTAATCGAACCACACCGCCCGTCGCTACTACCGATTGAATGGT
TCAGTGAGGCGTTCGGACTGGCCCAGGGAGGTCGGCAACGACCACCCAGGGCCGGAAA
GTTCTCCAAACTCGATCATTTAGAGGAAGTAAAAGTCGTAACAAGGTCTCCGTTGGTGA
ACCAGCGGAGGGATCATTGCTGAATATCCGTATTCGCCACCCTTTGTGAATATATACCTA
TCTCGTTGCCTCGGCGCCAGGCCCGGGGTTCCCCCCTAGGGGGTTCCCCCCACCTGTGGA
CCTCACGGTCCCGGGCGGAGCAGGCCCGCCGGCGGCCCACCAAAACTCTTGTTTGTAGA
ACCTATCTCTCTGAGTCTCATACGTAAAATGAATCAAAACTTTCAACAACGGATCTCTTG
GCTCTGGCATCGATGAAGAACGCAGCGAAATGCGATAAGTAATGTGAATTGCAGAATTC
AGTGAATCATCGAATCTTTGAACGCACATTGCGCCCGCTGGAATTCCGGCGGGCATGCC
TGTTCGAGCGTCATTTCAACCCCTCAAGCTTCAGCGCTTGGTGTTGGGGCATAGCCCGTG
AAAAGGCTAGCCCTTAAATTCAGTGGCGTGCTCGCTGAGACCCCCGGGTGAAGTAATCT
CCTTCTCGCCCGGGCCGGCTCGGCGGTGCACTCGCCGTAAAACCCGCTATCACGCACCTT
CTGAAAGTTGACCTCGGATCAGGTAGGAATACCCGCTGAACTTAAGCATATCAATAAGC
GGAGGAAAAGAAACCAACAGGGATTGCCCTAGTAACGGCGAGTGAAGCGGCAACAGCT
CAAATTTGAAATCTGGCCCCCCGGGGGTCCGAGTTGTAATTTGCAGAGGATGTCTTCTG
GCGCGGTGCCTTCCGAGTTCCCTGGAACGGGACGCCACAGAGGGTGAGAGCCCCGTAAT
GGTCGGATACCAAGCCTGTGTGAAGCTCCTTCGACGAGTCGAGTAGTTTGGGAATGCTG
CTCTAAATGGGAGGTAAATCTCTTCTAAAGCTAAATATTGGCCAGAGACCGATAGCGCA
CAAGTAGAGTGATCGAAAGATGAAAAGCACCTTGAAAAGGGGGTTAAACAGTACGTGA
AATTGTTGAAAGGGAAGCGCTCATGACCAGACTTGCGCCGGGCGGCTCAGCAGGGGTTC
CGCCCCTGTGTACTCCGTCCCGGTTCAGGCCAGCATCGGTTCTCGCTGGGGGAAAAGAA
CGGCGGGAACGTGGCCCTCCCTCGGGGGGGTGTTATAGCCCGTCGTCCGATACCCTGGC
GGGGTCCGAGGTTCGCGCATTCGCAAGGATGCTGGCGTAATGGTTATCAGTGACCCGTC
TTGAAACACGGACCAAGGAGTCGTCCTTTAGAGCGAGCGTCTGGGTGTTAAAACCCGCA
CGCGTAATGAAAGTGAAATTAGGTGAGAGCCCTTACGGGCGCATCATCGACCGATCCTG
ATGTTCTCGGACGGATTTGAGTAAGAGTTCTAACGGACGGACCCGAAAGACAGTGAACT
ATGCTTGTATAGGGTGAAGCCAGAGGAAACTCTGGTGGAGGCTCGCAGCGGTTCTGACG
TGCAAATCGATCGTCAAATATGAGCATGGGGGCGAAAGACTAATCGAACGGTCCGCTCC
TGGCGAAGCTGTTCCGAAACATCGTCAAGAAGCTGACCGGCGACATGATGGCGTACATG
AAGCGTTGCGTCAGCGAGGGCAAGCATTTCGATCTAGCGTTGGGCATCCGCCACTCAAC
CCTCACAAATGGTCTCAAGTACTCCCTTGCGACTGGAAACTGGGGCGAGCAGAAGAAGG
CGATGAGTTCCACCGCTGGTGTCTCGCAGGTGCTGAACCGGTACACATTCTCGTCCACCC
TATCGCATTTGCGGCGCACGAACACGCCTATCGGTCGTGACGGCAAGCTGGCCAAGCCT

CGTCAGCTGCACAATACACACTGGGGTCTCGTCTGTCCAGCAGAGACACCCGAGGGACA
GGCTTGCGGTCTTGTCAAGAACCTGTCTTTGATGTGCTACGTTAGTGTTGGTTCACCGGC
TGAGCCTATCAAGGACTTCATGGTGCAGCGTAATATGGAGGTCTTGGAGGAATACGAGC
CCGGCTCCAGCCCTGACGCAACCAAAATCTTCATCAACGGCACGTGGGTTGGTGTGCAC
AGTGAACCAGCACACTTGGTCACACTTGTGCAGGAGCTTCGGCGCAAATGCATCATTTC
TCACGAAGTCTCCCTCGTCCGCGACATTCGCGACCGCGAGTTCAAGATCTTCTCCGACGC
TGGTCGTGTCATGCGGCCTCTCTTCGTTGTTAATCAAGATGATAATCCCGAGACTGGAGC
GCAGCAGGGCACTCTGGCTCTCACCAAGCACCACATTCGCCGTCTCGAGGAAGATGCTC
AGTACCAGCGCAAGAAGCATGATGACGACTATTTCGGCTGGGATGGTCTTCAGAACAGC
GGTTGCATCGAGTACCTGGACGCTGAGGAAGAGGAGACATCAATGATCTGCATGAGTCC
CGAGGATCTCGAGGACTACCGCCAGCGCAAGGCTAGGGGCAAGGATGCCAAGGATGAT
GAACCGGAGGACGATGGCAGGAGTCTGAACGCTCGTGTGAAGACGAAGATCAACCCCG
ACATCCACATGTACACCCACTGCGAGATCCATCCAAGCATGCTCCTGGGAATATGCGCC
AGCATTATTCCCTTCCCTGATCACAATCAGGTATGTGAAATTCTCGCCGTACTATTATCA
CTCCTGCTGACCACATATATCTAGTCACCTCGCATCAATCCAATCAGCACCATCGCATCA
CCATCGCGCAAACATCAAGATCCAGCCAAAAGAGGTCGCGATATCTTCGTCGCGGTCGT
TTGCCCTCGCCCGCTGGCGGTTGTCCTTTTTTCGCCCGCCGCACAGTTTTGTTTTGTTGTG
CGGGGTTTTGCGCCTGGGTCTTATCTCAGATAAACGTGACCCACCCAACCGCCACCTGCC
TCATCCTCCGCCAACACCCTTTTTGCGCGCGCCTCTTTCCCACCCTCAAAATCGACGATG
ACAATGCTGACTCACATTTTCTACTGCAGCTGCTGAGCTCGGCAAGGGTTCCTTCAAGTA
CGCATGGGTCCTTGACAAGCTCAAGGCCGAGCGTGAGCGTGGTATCACCATCGACATTG
CTCTGTGGAAGTTCGAGACTCCCAAGTACTATGTCACCGTCATTGGTAAGCTCTATCCCA
TGCAGATGCCGCGCAGGTCTGGCTGCCCTGTCTCCCTGCGCTGCCACGCCATGCGCGAC
ACCGCGGGTTCTGATGTTTTTTGGCAACCCGTGCTAACGCAAAATTCACACCAGACGCTC
CCGGTCACCGTGACTTCATCAAGAACATGATCACTGGTACCTCCCAGGCTGACTGCGCC
GTTCTCATCATTGCCTCCGGTACTGGTGAGTTCGAGGCTGGTATCTCCAAGGATGGCCAG
ACCCGTGAGCACGCTCTGCTCGCCTACACCCTCGGTGTCAAGCAGCTCATTGTTGCTTGC
AACAAGATGGACACCGCTGGCTGGAAGCAGGACCGTTTCGAGGAGATCAAGAAGGAGA
CCACCAACTTCATCAAGAAGGTCGGCTTCAACCCCAAGCAGGTCGCTTTCGTCCCCATCT
CCGGCTTCCACGGCGACAACATGCTTGAGGCCTCCTCCAACATGCCCTGGTACAAGGGA
TGGACCAAGGAGACCCAGGACAAGAAGGAGGCCAAGGGTATGACCTTGCTCGACGCCA
TTGACGCCATCGAGCCCCCCAAGCGCCCCACCGACAAGCCCCTGCGTCTGCCCCTCCAG
GATGTCTACAAGATCGGCGGTATCGGAACTGTACCTGTCGGCCGTATCGAGACTGGTAT
CCTGAAGCCCGGTATGGTCGTCACCTTCGCCCCCTCCAACGTCACCACTGAGGTCAAGTC
TGTGGAGATGCACCACGAGCAGCTCACCGAGGGCCAGCCCGGTGACAACGTTGGTTTCA
ACGTCAAGAACGTCTCCGTCAAGGAGATTCGCCGTGGCAACGTCGCTGGTGACTCCAAG
AACGACCCCCCCCTGGGCGCCGCTTCCTTCAACGCCCAGGTCATTGTCCTGAACCACCCC
GGCCAGGTCGGTGCTGGATAC

>Melanconiella_spodiaea

ACCGATTGAATGGTTCAGTGAGGCGTTCGGACTGGCCCAGGGAGGTCGGCAACGACCAC
CCAGGGCCGGAAAGTTCTCCAAACTCGATCATTTAGAGGAAGTAAAAGTCGTAACAAGG
TCTCCGTTGGTGAACCAGCGGAGGGATCATTGCTGGAACACGTCCCCCAGGGCGTACCC
AGATACCCTTTGTGAACTTATACCTATATCGTTGCCTCGGCGCTGGCTGGGAGCCCCCTT
TCCAGGGCTCCCCCACCAGGGGGACCTCTAACGGTCTTCCTTAGTGGAAGCAGGCCCGC
CGGCGGCCCCCTAAACTCTTGTTTTTATAACGTATCTCTTCTGAGTAACAAAATTATAAA
TGAATCAAAACTTTCAACAACGGATCTCTTGGTTCTGGCATCGATGAAGAACGCAGCGA

AATGCGATAAGTAATGTGAATTGCAGAATTCAGTGAATCATCGAATCTTTGAACGCACA
TTGCGCCCGCTGGTATTCCGGCGGGCATGCCTGTTCGAGCGTCATTTCAACCCTCAAGCT
TTACCGCTTGGTGTTGGGGCGTAGCCTGTAGAAGGGCTAGCCCTGAAATTCAGTGGCGT
GCTCGCTAAGACTCCGAGCGTAGTAATTTTTCTCGCTTAGGTTGGATTAGCGGTGCCCA
GCCGTAAAACCTGCCATCGTGCACCTCTGAAAGTTGACCTCGGATCAGGTAGGAATACC
CGCTGAACTTAAGCATATCAATAAGCGGAGGAAAAGAAACCAACAGGGATTGCCCTAG
TAACGGCGAGTGAAGCGGCAACAGCTCAAATTTGAAATCTGGCCTCGGCCCGAGTTGTA
ATTTGCAGAGGATGCTTCTGGCGCGGTGCCTTCCGAGTTCCCTGGAACGGGACGCCACA
GAGGGTGAGAGCCCCGTATGGTCGGACACCAAGCCTGTGTGAAGCTCCTTCGACGAGTC
GAGTAGTTTGGGAATGCTGCTCAAAATGGGAGGTAAATCTCTTCTAAAGCTAAATACTG
GCCAGAGACCGATAGCGCACAAGTAGAGTGATCGAAAGATGAAAAGCACCTTGAAAAG
GGGGTTAAACAGTACGTGAAATTGTTGAAAGGGAAGCGCTTGTGACCAGACTTGCGCCG
GGCGGCTCATCAGGGGTTCTCCCCTGTGTACTCCGCCCGGTTCAGGCCAGCATCGGTTCT
CGCCGGGGGATAAGAACGGTGGGAACGTGGCCCTCCCTCGGGGGGGTGTTATAGCCCGC
CGTACGATATCCTGGCGGGGACCGAGGTTCGCGCTCCGCAAGGATGCTGGCGTAATGGT
CATCAGCGACCCGTCTTGAAACACGGACCAAGGAGTCGTCCATTAGAGCGAGCGTTTGG
GTGTAAAACCCGCACGCGTAATGAAAGTGAAATTAGGTGAGAGCTTCGGCGCATCATCG
ACCGATCCTGATGTTCTCGGATGGATTTGAGTAAGAGTTTTAACGGACGGACCCGAAAG
ACAGTGAACTATGCTTGTATAGGGTGAAGCCAGAGGAAACTCTGGTGGAGGCTCGCAGC
GGTTCTGACGTGCAAATCGATCGTCAAATATGAGCATGGGGGCGAAAGACTAATCGAAC
ACCGATTGAATGGTTCAGTGAGGCGTTCGGACTGGCCCAGGGAGGTCGGCAACGACCAC
CCAGGGCCGGAAAGTTCTCCAAACTCGATCATTTAGAGGAAGTAAAAGTCGTAACAAGG
TCTCCGTTGGTGAACCAGCGGAGGGATCATTGCTGGAACACGTCCCCCAGGGCGTACCC
AGATACCCTTTGTGAACTTATACCTATATCGTTGCCTCGGCGCTGGCTGGGAGCCCCCTT
TCCAGGGCTCCCCCACCAGGGGGACCTCTAACGGTCTTCCTTAGTGGAAGCAGGCCCGC
CGGCGGCCCCCTAAACTCTTGTTTTTATAACGTATCTCTTCTGAGTAACAAAATTATAAA
TGAATCAAAACTTTCAACAACGGATCTCTTGGTTCTGGCATCGATGAAGAACGCAGCGA
AATGCGATAAGTAATGTGAATTGCAGAATTCAGTGAATCATCGAATCTTTGAACGCACA
TTGCGCCCGCTGGTATTCCGGCGGGCATGCCTGTTCGAGCGTCATTTCAACCCTCAAGCT
TTACCGCTTGGTGTTGGGGCGTAGCCTGTAGAAGGGCTAGCCCTGAAATTCAGTGGCGT
GCTCGCTAAGACTCCGAGCGTAGTAATTTTTCTCGCTTAGGTTGGATTAGCGGTGCCCA
GCCGTAAAACCTGCCATCGTGCACCTCTGAAAGTTGACCTCGGATCAGGTAGGAATACC
CGCTGAACTTAAGCATATCAATAAGCGGAGGAAAAGAAACCAACAGGGATTGCCCTAG
TAACGGCGAGTGAAGCGGCAACAGCTCAAATTTGAAATCTGGCCTCGGCCCGAGTTGTA
ATTTGCAGAGGATGCTTCTGGCGCGGTGCCTTCCGAGTTCCCTGGAACGGGACGCCACA
GAGGGTGAGAGCCCCGTATGGTCGGACACCAAGCCTGTGTGAAGCTCCTTCGACGAGTC
GAGTAGTTTGGGAATGCTGCTCAAAATGGGAGGTAAATCTCTTCTAAAGCTAAATACTG
GCCAGAGACCGATAGCGCACAAGTAGAGTGATCGAAAGATGAAAAGCACCTTGAAAAG
GGGGTTAAACAGTACGTGAAATTGTTGAAAGGGAAGCGCTTGTGACCAGACTTGCGCCG
GGCGGCTCATCAGGGGTTCTCCCCTGTGTACTCCGCCCGGTTCAGGCCAGCATCGGTTCT
CGCCGGGGGATAAGAACGGTGGGAACGTGGCCCTCCCTCGGGGGGGTGTTATAGCCCGC
CGTACGATATCCTGGCGGGGACCGAGGTTCGCGCTCCGCAAGGATGCTGGCGTAATGGT
CATCAGCGACCCGTCTTGAAACACGGACCAAGGAGTCGTCCATTAGAGCGAGCGTTTGG
GTGTAAAACCCGCACGCGTAATGAAAGTGAAATTAGGTGAGAGCTTCGGCGCATCATCG
ACCGATCCTGATGTTCTCGGATGGATTTGAGTAAGAGTTTTAACGGACGGACCCGAAAG
ACAGTGAACTATGCTTGTATAGGGTGAAGCCAGAGGAAACTCTGGTGGAGGCTCGCAGC
GGTTCTGACGTGCAAATCGATCGTCAAATATGAGCATGGGGGCGAAAGACTAATCGAAC

GGTCCGCTCTTGGCAAAGCTGTTCCGAAACATCGTCAAGAAGCTGACAGGTGACATGAT
GGCGTACATGAAGCGTTGTGTCAGCGAGGGCAAGCATTTCGATCTGGCGTTGGGCATCC
GCCATTCCACCCTCACAAATGGTCTCAAGTACTCTCTTGCGACTGGAAACTGGGGTGAG
CAGAAGAAGGCGATGAGTTCCACTGCTGGTGTCTCGCAGGTGCTGAACCGGTACACGTT
TTCGTCTACCCTTTCGCATTTGCGGCGCACGAACACGCCCATTGGTCGTGACGGAAAGCT
GGCCAAGCCTCGTCAGCTGCACAACACTCACTGGGGCCTCGTCTGTCCGGCAGAGACTC
CTGAGGGACAGGCCTGCGGTCTTGTCAAGAACCTGTCTTTAATGTGCTACGTCAGCGTTG
GCTCACCGGCCGAGCCTATCAAGGACTTCATGGTGCAGAGAAACATGGAGGTTTTGGAG
GAATACGAACCCGGCTCCAGCCCTGACGCGACCAAAATATTCATCAACGGCACGTGGGT
TGGTGTCCACAACGAGCCAGCTCACTTGGTCACGCTTGTGCAAGAGCTTCGACGCAAAT
GCATAATTTCTCACGAAGTCTCCCTCGTTCGCGACATTCGCGATCGTGAGTTCAAGATTT
TCTCCGACGCTGGTCGTGTGATGCGGCCTCTCTTCGTTGTCAACCAAGAGGACAACCCCG
AGACTGGAGCGCAGCAGGGCACTCTGGCCCTCACCAAGCACCACATTCGCCGTCTCGAG
GAAGATGCTCAGTACCAGCGCAAGAAGCATGATGAAGACTATTTTGGCTGGGATGGTCT
TCAGAACAGCGGTTGCATCGAGTACCTGGATGCCGAGGAAGAGGAGACAGCAATGATC
TGCATGAGTCCTGAGGATCTTGAGGACTACCGACAACGCAAGGCTAGGGGCAAGGATG
CCAAGGATGATGAGCCTGAGGATGATGGCAGGAGTCTGAACGCTCGTGTGAAGACGAA
GATCAATACCGACATCCACATGTACACCCACTGCGAGATCCATCCGAGCATGCTCCTGG
GAATCTGCGCCAGCATCATTCCCTTCCCAGACCACAATCAGGTACGTAAAAGCTTTGCA
ATACTACCATCACTCCAGCTGACCACATGTATCTAGTCGCCTCGATCACCCATGTCATTA
TCATCACAATCGCAGAACCACAAAGGCAAACGGTCGCGATTTCTTCATCGCGTCGCGAC
TGTTGGCCTTGGCTGGCTATGCTTTTTCGCCCATCGCACATTTTCGCTTTGTGGTGCGGG
GTTTTGCGTCTGGGTCTTATCTCAGATAAACGTGACCCACCCGACCCGCCATCTCAAATC
ATCCTCTGCCAACCCACCCCCTCGTGCACCACTGCTCCTCACACCATGACAACGACGATG
CTGACTGAATTCTTCTATCACAGCTGCTGAGCTCGGCAAGGGTTCCTTCAAGTACGCATG
GGTCCTTGACAAGCTCAAGGCCGAGCGTGAGCGTGGTATCACCATCGACATTGCTCTGT
GGAAGTTCGAGACTCCCAAGTACTATGTCACCGTCATTGGTAAGCTTGGCCCTCTTGACG
ATGTCGCGCAGGGCTGGCAGCCCCGCCTATTTGCGCTGCCCCGCCATGGGCGACACACT
GCGCACTGTCACAATTTTCGCAACCCGTACTAATAAACGTCGCCACAGACGCTCCCGGT
CACCGTGACTTCATCAAGAACATGATCACTGGTACCTCCCAGGCTGACTGCGCCGTTCTC
ATCATCGCCTCCGGTACTGGTGAGTTCGAGGCCGGTATCTCCAAGGACGGCCAGACCCG
TGAGCACGCTCTGCTTGCCTACACCCTCGGTGTTAAGCAGCTCATTGTTGCTTGCAACAA
GATGGACACCGCTGGCTGGAAGCAGGACCGTTTTGAGGAAATCAAGAAGGAGACCACC
AACTTCATTAAGAAGGTCGGCTTCAACCCCAAGCAGGTCGCTTTCGTCCCCATCTCTGGC
TTCCACGGCGACAACATGCTTGAGGCCTCCGCCAACATGCCCTGGTACAAGGGCTGGAC
CAAGGAGACCCAGGACAAGAAGGAGGCCAAGGGTATGACCCTGCTCGACGCGATTGAC
GCCATCGAGCCCCCCAAGCGTCCCACCGAGAAGCCCCTGCGTCTGCCCCTCCAGGATGT
CTACAAGATCGGCGGTATTGGAACTGTACCTGTCGGCCGTATCGAGACTGGTGTCCTGA
AGCCCGGTATGGTCGTCACCTTCGCTCCCTCCAACGTCACCACTGAGGTCAAGTCCGTGG
AGATGCACCACGAGCAGCTGACCGAGGGTCAGCCCGGTGACAACGTTGGTTTCAACGTC
AAGAACGTCTCCGTCAAGGAGATTCGCCGTGGCAACGTCGCTGGTGACTCCAAGAACGA
TCCCCCTATGGCCGCCGCTTCCTTCAACGCCCAGGTCATCGTCCTGAACCACCCCGGCCA
GGTCGGTGCTGGATAC

>Melanconiella_spodiaea

TACCGATTGAATGGTTCAGTGAGGCGTTCGGACTGGCCCAGGGAGGTCGGCAACGACCA
CCCAGGGCCGGAAAGTTCTCCAAACTCGATCATTTAGAGGAAGTAAAAGTCGTAACAAG

GTCTCCGTTGGTGAACCAGCGGAGGGATCATTGCTGGAACACGTCCCCCAGGGCGTACC
CAGATACCCTTTGTGAACTTATACCTATATCGTTGCCTCGGCGCTGGCTGGGAGCCCCCT
TTCCAGGGCTCCCCCACCAGGGGGACCTCTAACGGTCTTCCTTAGTGGAAGCAGGCCCG
CCGGCGGCCCCCTAAACTCTTGTTTTTATAACGTATCTCTTCTGAGTAACAAAATTATAA
ATGAATCAAAACTTTCAACAACGGATCTCTTGGTTCTGGCATCGATGAAGAACGCAGCG
AAATGCGATAAGTAATGTGAATTGCAGAATTCAGTGAATCATCGAATCTTTGAACGCAC
ATTGCGCCCGCTGGTATTCCGGCGGGCATGCCTGTTCGAGCGTCATTTCAACCCTCAAGC
TTTACCGCTTGGTGTTGGGGCGTAGCCTGTAGAAGGGCTAGCCCCGAAATTCAGTGGCG
TGCTCGCTAAGACTCCGAGCGTAGTAATTTTTCTCGCTTAGGTTGGATTAGCGGTGCCC
AGCCGTAAAACCTGCCATCGTGCACCTCTGAAAGTTGACCTCGGATCAGGTAGGAATAC
CCGCTGAACTTAAGCATATCAATAAGCGGAGGAAAAGAAACCAACAGGGATTGCCCTA
GTAACGGCGAGTGAAGCGGCAACAGCTCAAATTTGAAATCTGGCCTCGGCCCGAGTTGT
AATTTGCAGAGGATGCTTCTGGCGCGGTGCCTTCCGAGTTCCCTGGAACGGGACGCCAC
AGAGGGTGAGAGCCCCGTATGGTCGGACACCAAGCCTGTGTGAAGCTCCTTCGACGAGT
CGAGTAGTTTGGGAATGCTGCTCAAAATGGGAGGTAAATCTCTTCTAAAGCTAAATACT
GGCCAGAGACCGATAGCGCACAAGTAGAGTGATCGAAAGATGAAAAGCACCTTGAAAA
GGGGGTTAAACAGTACGTGAAATTGTTGAAAGGGAAGCGCTTGTGACCAGACTTGCGCC
GGGCGGCTCATCAGGGGTTCTCCCCTGTGTACTCCGCCCGGTTCAGGCCAGCATCGGTTC
TCGCCGGGGGATAACAACGGTGGGAACGTGGCCCTCCCTCGGGGGGGTGTTATAGCCCG
CCGTACGATATCCTGGCGGGGACCGAGGTTCGCGCTCCGCAAGGATGCTGGCGTAATGG
TCATCAGCGACCCGTCTTGAAACACGGACCAAGGAGTCGTCCATTAGAGCGAGCGTTTG
GGTGTAAAACCCGCACGCGTAATGAAAGTGAAATTAGGTGAGAGCTTCGGCGCATCATC
GACCGATCCTGATGTTCTCGGATGGATTTGAGTAAGAGTTTTAACGGACGGACCCGAAA
GACAGTGAACTATGCTTGTATAGGGTGAAGCCAGAGGAAACTCTGGTGGAGGCTCGCAG
CGGTTCTGACGTGCAAATCGATCGTCAAATATGAGCATGGGGGCGAAAGACTAATCGAA
CTACCGATTGAATGGTTCAGTGAGGCGTTCGGACTGGCCCAGGGAGGTCGGCAACGACC
ACCCAGGGCCGGAAAGTTCTCCAAACTCGATCATTTAGAGGAAGTAAAAGTCGTAACAA
GGTCTCCGTTGGTGAACCAGCGGAGGGATCATTGCTGGAACACGTCCCCCAGGGCGTAC
CCAGATACCCTTTGTGAACTTATACCTATATCGTTGCCTCGGCGCTGGCTGGGAGCCCCC
TTTCCAGGGCTCCCCCACCAGGGGGACCTCTAACGGTCTTCCTTAGTGGAAGCAGGCCC
GCCGGCGGCCCCCTAAACTCTTGTTTTTATAACGTATCTCTTCTGAGTAACAAAATTATA
AATGAATCAAAACTTTCAACAACGGATCTCTTGGTTCTGGCATCGATGAAGAACGCAGC
GAAATGCGATAAGTAATGTGAATTGCAGAATTCAGTGAATCATCGAATCTTTGAACGCA
CATTGCGCCCGCTGGTATTCCGGCGGGCATGCCTGTTCGAGCGTCATTTCAACCCTCAAG
CTTTACCGCTTGGTGTTGGGGCGTAGCCTGTAGAAGGGCTAGCCCCGAAATTCAGTGGC
GTGCTCGCTAAGACTCCGAGCGTAGTAATTTTTCTCGCTTAGGTTGGATTAGCGGTGCC
CAGCCGTAAAACCTGCCATCGTGCACCTCTGAAAGTTGACCTCGGATCAGGTAGGAATA
CCCGCTGAACTTAAGCATATCAATAAGCGGAGGAAAAGAAACCAACAGGGATTGCCCT
AGTAACGGCGAGTGAAGCGGCAACAGCTCAAATTTGAAATCTGGCCTCGGCCCGAGTTG
TAATTTGCAGAGGATGCTTCTGGCGCGGTGCCTTCCGAGTTCCCTGGAACGGGACGCCA
CAGAGGGTGAGAGCCCCGTATGGTCGGACACCAAGCCTGTGTGAAGCTCCTTCGACGAG
TCGAGTAGTTTGGGAATGCTGCTCAAAATGGGAGGTAAATCTCTTCTAAAGCTAAATAC
TGGCCAGAGACCGATAGCGCACAAGTAGAGTGATCGAAAGATGAAAAGCACCTTGAAA
AGGGGGTTAAACAGTACGTGAAATTGTTGAAAGGGAAGCGCTTGTGACCAGACTTGCGC
CGGGCGGCTCATCAGGGGTTCTCCCCTGTGTACTCCGCCCGGTTCAGGCCAGCATCGGTT
CTCGCCGGGGGATAACAACGGTGGGAACGTGGCCCTCCCTCGGGGGGGTGTTATAGCCC
GCCGTACGATATCCTGGCGGGGACCGAGGTTCGCGCTCCGCAAGGATGCTGGCGTAATG

GTCATCAGCGACCCGTCTTGAAACACGGACCAAGGAGTCGTCCATTAGAGCGAGCGTTT
GGGTGTAAAACCCGCACGCGTAATGAAAGTGAAATTAGGTGAGAGCTTCGGCGCATCAT
CGACCGATCCTGATGTTCTCGGATGGATTTGAGTAAGAGTTTTAACGGACGGACCCGAA
AGACAGTGAACTATGCTTGTATAGGGTGAAGCCAGAGGAAACTCTGGTGGAGGCTCGCA
GCGGTTCTGACGTGCAAATCGATCGTCAAATATGAGCATGGGGGCGAAAGACTAATCGA
ACGGTCCGCTCTTGGCAAAGCTGTTCCGAAACATCGTCAAGAAGCTGACAGGTGACATG
ATGGCGTACATGAAGCGTTGTGTCAGCGAGGGCAAGCATTTCGATCTGGCGTTGGGCAT
CCGCCATTCCACCCTCACAAATGGTCTCAAGTACTCTCTTGCGACTGGAAACTGGGGTGA
GCAGAAGAAGGCGATGAGTTCCACTGCTGGTGTCTCGCAGGTGCTGAACCGGTACACGT
TTTCGTCTACCCTTTCGCATTTGCGGCGCACGAACACGCCCATTGGTCGTGACGGAAAGC
TGGCCAAGCCTCGTCAGCTGCACAACACTCACTGGGGCCTCGTCTGTCCGGCAGAGACT
CCTGAGGGACAGGCCTGCGGTCTTGTCAAGAACCTGTCTTTAATGTGCTACGTCAGCGTT
GGCTCACCGGCCGAGCCTATCAAGGACTTCATGGTGCAGAGAAACATGGAGGTTTTGGA
GGAATACGAACCCGGCTCCAGCCCTGACGCGACCAAAATATTCATCAACGGCACGTGGG
TTGGTGTCCACAACGAGCCAGCTCACTTGGTCACGCTTGTGCAAGAGCTTCGACGCAAA
TGCATCATTTCTCACGAAGTCTCCCTCGTTCGCGACATTCGCGATCGCGAGTTCAAGATC
TTCTCCGACGCTGGTCGTGTGATGCGGCCTCTCTTCGTCGTCAACCAAGAGGACAACCCC
GAGACTGGAGCGCAGCAGGGCACTCTGGCCCTCACCAAGCACCACATTCGCCGTCTCGA
GGAAGATGCTCAGTATCAGCGCAAGAAGCATGATGAAGACTATTTTGGCTGGGATGGTC
TTCAGAATAGCGGTTGCATCGAGTACCTGGATGCCGAGGAAGAGGAGACAGCAATGAT
CTGCATGAGTCCTGAGGATCTTGAGGACTACCGACAACGCAAGGCTAGGGGCAAGGAT
GCCAAGGATGATGAGCCTGAGGATGATGGCAGGAGTCTGAACGCTCGTGTGAAGACGA
AGATCAATACCGACATCCACATGTACACCCACTGCGAGATCCATCCGAGCATGCTCCTG
GGAATCTGCGCCAGCATCATTCCCTTCCCAGACCACAATCAGGTATGTAAAAGCTTTGC
AATACTACCATCACTCCAGCTGACCACATGTATCTAGTCGCCTCGCATCACCCATGTCAT
TATCATCACAATCGCAGAACCACAAAGGCAAACGGTCGCGATTTCTTCATCGCGTCGCG
ACTGTTGGCCTTGGCTGGCTATGCTTTTTTCGCCCATCGCACATTTTCGCTTTGTGGTGCG
GGGTTTTGCGTCTGGGTCTTATCTCAGATAAACGTGACCCACCCGACCCGCCATCTCAAA
TCATCCTCTGCCAACCCACCCCCTCGTGCACCACTGCTCCTCACACCATGACAACGACGA
TGCTGACTGAATTCTTCTATCACAGCTGCTGAGCTCGGCAAGGGTTCCTTCAAGTACGCA
TGGGTCCTTGACAAGCTCAAGGCCGAGCGTGAGCGTGGTATCACCATCGACATTGCTCT
GTGGAAGTTCGAGACTCCCAAGTACTATGTCACCGTCATTGGTAAGCTTGGCCCTCTTGA
CGATGTCGCGCAGGGCTGGCAGCCCCGCCTATTTGCGCTGCCCCGCCATGGGCGACACA
CTGCGCACTGTCACAATTTTCGCAACCCGTACTAATAAACGTCGCCACAGACGCTCCCG
GTCACCGTGACTTCATCAAGAACATGATCACTGGTACCTCCCAGGCTGACTGCGCCGTTC
TCATCATCGCCTCCGGTACTGGTGAGTTCGAGGCCGGTATCTCCAAGGACGGCCAGACC
CGTGAGCACGCTCTGCTTGCCTACACCCTCGGTGTTAAGCAGCTCATTGTTGCTTGCAAC
AAGATGGACACCGCTGGCTGGAAGCAGGACCGTTTTGAGGAAATCAAGAAGGAGACCA
CCAACTTCATTAAGAAGGTCGGCTTCAACCCCAAGCAGGTCGCTTTCGTCCCCATCTCTG
GCTTCCACGGCGACAACATGCTTGAGGCCTCCGCCAACATGCCCTGGTACAAGGGCTGG
ACCAAGGAGACCCAGGACAAGAAGGAGGCCAAGGGTATGACCCTGCTCGACGCAATTG
ACGCCATCGAGCCCCCCAAGCGTCCCACCGAGAAGCCCCTGCGTCTGCCCCTCCAGGAT
GTCTACAAGATCGGCGGTATTGGAACTGTACCTGTCGGCCGTATCGAGACTGGTGTCCT
GAAGCCCGGTATGGTCGTCACCTTCGCTCCCTCCAACGTCACCACTGAGGTCAAGTCCGT
GGAGATGCACCACGAGCAGCTGACCGAGGGTCAGCCCGGTGACAACGTTGGTTTCAACG
TCAAGAACGTCTCCGTCAAGGAGATTCGCCGTGGCAACGTCGCTGGTGACTCCAAGAAC
GATCCCCCTATGGCCGCCGCTTCCTTCAACGCCCAGGTCATCGTCCTGAACCACCCCGGC
CAGGTCGGTGCTGGATAC

>Melanconiella_spodiaea

GAACCAGCGGAGGGATCATTGCTGGAACACGTCCCCCAGGGCGTACCCAGATACCCTTT
GTGAACTTATACCTATATCGTTGCCTCGGCGCTGGCTGGGAGCCCCCTTTCCAGGGCTCC
CCCACCAGGGGGACCTCTAACGGTCTTCCTTAGTGGAAGCAGGCCCGCCGGCGGCCCCC
TAAACTCTTGTTTTTAATAACGTATCTCTTCTGAGTAACAAAATTATAAATGAATCAAAA
CTTTCAACAACGGATCTCTTGGTTCTGGCATCGATGAAGAACGCAGCGAAATGCGATAA
GTAATGTGAATTGCAGAATTCAGTGAATCATCGAATCTTTGAACGCACATTGCGCCCGCT
GGTATTCCGGCGGGCATGCCTGTTCGAGCGTCATTTCAACCCTCAAGCTTTACCGCTTGG
TGTTGGGGCGTAGCCTGTAGAAGGGCTAGCCCTGAAATTCAGTGGCGTGCTCGCTAAGA
CTCCGAGCGTAGTAATTTTTTCTCGCTTAGGTTGGATTAGCGGTGCCCAGCCGTAAAACC
TGCCATCGTGCACCTCTGAAAGTTGACCTCGGATCAGGTAGGAATACCCGCTGAACTTA
AGCATATCAATAAGCGGAGGAAAAGAAACCAACAGGGATTGCCCTAGTAACGGCGAGT
GAAGCGGCAACAGCTCAAATTTGAAATCTGGCCTCGGCCCGAGTTGTAATTTGCAGAGG
ATGCTTCTGGCGCGGTGCCTTCCGAGTTCCCTGGAACGGGACGCCACAGAGGGTGAGAG
CCCCGTATGGTCGGACACCAAGCCTGTGTGAAGCTCCTTCGACGAGTCGAGTAGTTTGG
GAATGCTGCTCAAAATGGGAGGTAAATCTCTTCTAAAGCTAAATACTGGCCAGAGACCG
ATAGCGCACAAGTAGAGTGATCGAAAGATGAAAAGCACCTTGAAAAGGGGGTTAAACA
GTACGTGAAATTGTTGAAAGGGAAGCGCTTGTGACCAGACTTGCGCCGGGCGGCTCATC
AGGGGTTCTCCCCTGTGTACTCCGCCCGGTTCAGGCCAGCATCGGTTCTCGCCGGGGGAT
AAGAACGGTGGGAACGTGGCCCTCCCTCGGGGGGGTGTTATAGCCCGCCGTACGATATC
CTGGCGGGGACCGAGGTTCGCGCTCCGCAAGGATGCTGGCGTAATGGTCATCAGCGACC
CGTCTTGAAACACGGACCAAGGAGTCGTCCATTAGAGCGAGCGTTTGGGTGTAAAACCC
GCACGCGTAATGAAAGTGAAATTAGGTGAGAGCTTCGGCGCATCATCGACCGATCCTGA
TGTTCTCGGATGGATTTGAGTAAGAGTTTTAACGGACGGACCCGAAAGACAGTGAACTA
TGCTTGTATAGGGTGAAGCCAGAGGAAACTCTGGTGGAGGCTCGCAGCGGTTCTGACGT
GCAAATCGATCGTCAAATATGAGCATGGGGGCGAAAGACTAATCGAACGAACCAGCGG
AGGGATCATTGCTGGAACACGTCCCCCAGGGCGTACCCAGATACCCTTTGTGAACTTAT
ACCTATATCGTTGCCTCGGCGCTGGCTGGGAGCCCCCTTTCCAGGGCTCCCCCACCAGGG
GGACCTCTAACGGTCTTCCTTAGTGGAAGCAGGCCCGCCGGCGGCCCCCTAAACTCTTGT
TTTTAATAACGTATCTCTTCTGAGTAACAAAATTATAAATGAATCAAAACTTTCAACAAC
GGATCTCTTGGTTCTGGCATCGATGAAGAACGCAGCGAAATGCGATAAGTAATGTGAAT
TGCAGAATTCAGTGAATCATCGAATCTTTGAACGCACATTGCGCCCGCTGGTATTCCGGC
GGGCATGCCTGTTCGAGCGTCATTTCAACCCTCAAGCTTTACCGCTTGGTGTTGGGGCGT
AGCCTGTAGAAGGGCTAGCCCTGAAATTCAGTGGCGTGCTCGCTAAGACTCCGAGCGTA
GTAATTTTTTCTCGCTTAGGTTGGATTAGCGGTGCCCAGCCGTAAAACCTGCCATCGTGC
ACCTCTGAAAGTTGACCTCGGATCAGGTAGGAATACCCGCTGAACTTAAGCATATCAAT
AAGCGGAGGAAAAGAAACCAACAGGGATTGCCCTAGTAACGGCGAGTGAAGCGGCAAC
AGCTCAAATTTGAAATCTGGCCTCGGCCCGAGTTGTAATTTGCAGAGGATGCTTCTGGCG
CGGTGCCTTCCGAGTTCCCTGGAACGGGACGCCACAGAGGGTGAGAGCCCCGTATGGTC
GGACACCAAGCCTGTGTGAAGCTCCTTCGACGAGTCGAGTAGTTTGGGAATGCTGCTCA
AAATGGGAGGTAAATCTCTTCTAAAGCTAAATACTGGCCAGAGACCGATAGCGCACAAG
TAGAGTGATCGAAAGATGAAAAGCACCTTGAAAAGGGGGTTAAACAGTACGTGAAATT
GTTGAAAGGGAAGCGCTTGTGACCAGACTTGCGCCGGGCGGCTCATCAGGGGTTCTCCC
CTGTGTACTCCGCCCGGTTCAGGCCAGCATCGGTTCTCGCCGGGGGATAAGAACGGTGG
GAACGTGGCCCTCCCTCGGGGGGGTGTTATAGCCCGCCGTACGATATCCTGGCGGGGAC
CGAGGTTCGCGCTCCGCAAGGATGCTGGCGTAATGGTCATCAGCGACCCGTCTTGAAAC

ACGGACCAAGGAGTCGTCCATTAGAGCGAGCGTTTGGGTGTAAAACCCGCACGCGTAAT
GAAAGTGAAATTAGGTGAGAGCTTCGGCGCATCATCGACCGATCCTGATGTTCTCGGAT
GGATTTGAGTAAGAGTTTTAACGGACGGACCCGAAAGACAGTGAACTATGCTTGTATAG
GGTGAAGCCAGAGGAAACTCTGGTGGAGGCTCGCAGCGGTTCTGACGTGCAAATCGATC
GTCAAATATGAGCATGGGGGCGAAAGACTAATCGAACGGTCCGCTCTTGGCAAAGCTGT
TCCGAAACATCGTCAAGAAGCTGACAGGTGACATGATGGCGTACATGAAGCGTTGTGTC
AGCGAGGGCAAGCATTTCGATCTGGCGTTGGGCATCCGCCACTCCACCCTCACAAATGG
TCTCAAGTACTCTCTTGCGACTGGAAACTGGGGTGAGCAGAAGAAGGCGATGAGTTCCA
CTGCTGGTGTCTCGCAGGTGCTGAACCGGTACACGTTTTCGTCTACCCTTTCGCATTTGC
GGCGCACGAACACGCCCATTGGTCGTGACGGAAAGCTGGCCAAGCCTCGTCAGCTGCAC
AACACTCACTGGGGCCTCGTCTGTCCGGCAGAGACTCCTGAGGGACAGGCCTGCGGTCT
TGTCAAGAACCTGTCTTTAATGTGCTACGTCAGCGTTGGCTCACCGGCCGAGCCTATCAA
GGACTTCATGGTGCAGAGAAACATGGAGGTTTTGGAGGAATACGAACCCGGCTCCAGCC
CTGACGCGACCAAAATATTCATCAACGGCACGTGGGTTGGTGTCCACAACGAGCCAGCT
CACTTGGTCACGCTTGTGCAAGAGCTTCGACGCAAATGCATCATTTCTCACGAAGTCTCC
CTCGTTCGCGACATTCGCGATCGCGAGTTCAAGATCTTCTCCGACGCTGGTCGTGTGATG
CGGCCTCTCTTCGTCGTCAACCAAGAGGACAACCCCGAGACTGGAGCGCAGCAGGGCAC
TCTGGCCCTCACCAAGCACCACATTCGCCGTCTCGAGGAAGATGCTCAGTATCAGCGCA
AGAAGCATGATGAAGACTATTTTGGCTGGGATGGTCTTCAGAACAGCGGTTGCATCGAG
TACCTGGATGCCGAGGAAGAGGAGACAGCAATGATCTGCATGAGTCCTGAGGATCTTGA
GGACTACCGACAACGCAAGGCTAGGGGCAAGGATGCCAAGGATGATGAGCCTGAGGAT
GATGGCAGGAGTCTGAACGCTCGTGTGAAGACGAAGATCAATACCGACATCCACATGTA
CACCCACTGCGAGATCCATCCGAGCATGCTCCTGGGAATCTGCGCCAGCATCATTCCCTT
CCCAGACCACAATCAGGTATGTAAAAGCTTTGCAATACTACCATCACTCCAGCTGACCA
CATGTATCTAGTCGCCTCGCATCACCCATGTCATTATCATCACAATCGCAGAACCACAAA
GGCAAACGGTCGCGATTTCTTCATCGCGTCGCGACTGTTGGCCTTGGCTGGCTATGCTTT
TTTCGCCCATCGCACATTTTCGCTTTGTGGTGCGGGGTTTTGCGTCTGGGTCTTATCTCAG
ATAAACGTGACCCACCCGACCCGCCATCTCAAATCATCCTCTGCCAACCCACCCCCTCGT
GCACCACTGCTCCTCACACCATGACAACGACGATGCTGACTGAATTCTTCTATCACAGCT
GCTGAGCTCGGCAAGGGTTCCTTCAAGTACGCATGGGTCCTTGACAAGCTCAAGGCCGA
GCGTGAGCGTGGTATCACCATCGACATTGCTCTGTGGAAGTTCGAGACTCCCAAGTACT
ATGTCACCGTCATTGGTAAGCTTGGCCCTCTTGACGATGTCGCGCAGGGCTGGCAGCCCC
GCCTATTTGCGCTGCCCCGCCATGGGCGACACACTGCGCACTGTCACAATTTTCGCAACC
CGTACTAATAAACGTCGCCACAGACGCTCCCGGTCACCGTGACTTCATCAAGAACATGA
TCACTGGTACCTCCCAGGCTGACTGCGCCGTTCTCATCATCGCCTCCGGTACTGGTGAGT
TCGAGGCCGGTATCTCCAAGGACGGCCAGACCCGTGAGCACGCTCTGCTTGCCTACACC
CTCGGTGTTAAGCAGCTCATTGTTGCTTGCAACAAGATGGACACCGCTGGCTGGAAGCA
GGACCGTTTTGAGGAAATCAAGAAGGAGACCACCAACTTCATTAAGAAGGTCGGCTTCA
ACCCCAAGCAGGTCGCTTTCGTCCCCATCTCTGGCTTCCACGGCGACAACATGCTTGAGG
CCTCCGCCAACATGCCCTGGTACAAGGGCTGGACCAAGGAGACCCAGGACAAGAAGGA
GGCCAAGGGTATGACCCTGCTCGACGCGATTGACGCCATCGAGCCCCCCAAGCGTCCCA
CCGAGAAGCCCCTGCGTCTGCCCCTCCAGGATGTCTACAAGATCGGCGGTATTGGAACT
GTACCTGTCGGCCGTATCGAGACTGGTGTCCTGAAGCCCGGTATGGTCGTCACCTTCGCT
CCCTCCAACGTCACCACTGAGGTCAAGTCCGTGGAGATGCACCACGAGCAGCTGACCGA
GGGTCAGCCCGGTGACAACGTTGGTTTCAACGTCAAGAACGTCTCCGTCAAGGAGATTC
GCCGTGGCAACGTCGCTGGTGACTCCAAGAACGATCCCCCTATGGCCGCCGCTTCCTTCA
ACGCCCAGGTCATCGTCCTGAACCACCCCGGCCAGGTCGGTGCTGGATAC

>Melanconiellasyzygii

TAACAAGGTCTCCGTTGGTGAACCAGCGGAGGGATCATTGCTGGAACACGCCCTCCGGG
CGTCCCCAGATACCCTGTGTGAACTTATACCTGTCGTTGCCTCGGCGCTGGCTGGTGGGC
TCCCCGCCCACCCCACTGGACCGTCACGGTCCGGCGGAGCAGGCCCGCCGGCGGCCTTT
CCAACTCTTGTTTATATCACGTATCTCTTCTGAGCAGACAAAACAAAATGAATCAAAACT
TTCAACAACGGATCTCTTGGTTCTGGCATCGATGAAGAACGCAGCGAAATGCGATAAGT
AATGTGAATTGCAGAATTCAGTGAATCATCGAATCTTTGAACGCACATTGCGCCCGCTG
GTATTCCAGCGGGCATGCCTGTTCGAGCGTCATTTCAACCCTCAAGCTGTCCTCAGCTTG
GTGTTGGGGCGTAGCCTGTGCCCACGGGCTAGCCCTGAAACCTACCGGCGTGCTCACTA
AGACTCCGAGCGTAGTAATCTCTTCTCGCTTAGGCTGGCTTAGCGGTGCGTAGCCGTGAA
AACCCCCCAATTTTCTGAAAGTTGACCTCGGATCAGGTAGGAATACCCGCTGAACTTAA
GCATATCAATAACGGCGAGTGAAGCGGCAACAGCTCAAATTTGAAATCTGGCCCTCGGG
CCCGAGTTGTAATTTGCAGAGGATGCTTCTGGCGCGGTGCCTTCCGAGTTCCCTGGAACG
GGACGCCACAGAGGGTGAGAGCCCCGTATGGTCGGACACCAAGCCTGTGTGAAGCTCCT
TCGACGAGTCGAGTAGTTTGGGAATGCTGCTCAAAATGGGAGGTAAATCTCTTCTAAAG
CTAAATACCGGCCAGAGACCGATAGCGCACAAGTAGAGTGATCGAAAGATGAAAAGCA
CCTTGAAAAGGGGGTTAAACAGTACGTGAAATTGTTGAAAGGGAAGCGCTTGTGACCAG
ACTTGTGCCGGGCGGCTCATCAGGGGTTCTCCCCTGTGTATTCCGCCCGGTTCAGGCCAG
CATCGGTTCTCACCGGGGGATAAGAACGGTAGGAACGTGGCTCCCCCTCGGGGGAGTGT
TATAGCCTGCCGTACGATACCCTGGCGGGGACCGAGGTTCGCGCTCCGCAAGGATGCTG
GCGTAATGGTCATCAGCGACCCGTCTTGAAACACGGACCAAGGAGTCGTCCATTAGAGC
GAGCGTTTGGGTGTAAAACCCGCACGCGTAATGAAAGTGAAATTAGGTGAGAGCTTCGG
CGCATCATCGACCGATCCTGATGTTCTCGGATGGATTTGAGTAAGAGTTTTAACGGACG
GACCCGAAAGACAGTGAACTATGCTTGTATAGGGTGAAGCCAGAGGAAACTCTGGTGG
AGGCTCGCAGCGGTTCTGACGTGCAAATCGATCGTCAAATATGAGCATGGGGGCGAAAG
??
??
??
??
??
??
??
??
??
??
??
??
??
??
??
??
??
??
??
??
??

??
??
??
??
??
??
??
??
?????????????

>Melanconis_stilbostoma

CACACCGCCCGTCGCTACTACCGATTGAATGGTTCAGTGAGGCGTTGGGACTGGCCTAG
GGAGGTCGGCAACGACCACCCAGGGCCGGAAACTTCTCCAAACTCGATCATTTAGAGGA
AGTAAAAGTCGTAACAAGGTCTCCGTTGGTGAACCAGCGGAGGGATCATTGCTGGAATA
AACGCCCTCACGGGTGCTACCCAGAAACCCTTTGTGAATTATACCAAACCGTTGCCTCG
GCATTAGCTGAATCTTTTCAAGGGATTCCCTTTTTCTTCGGAGAAAGGAGCAGGCTGGCC
GGTGGCCCTATAAACTCTTATTGTTAAAATAGTACTCTGAGTAAAACAACTATAAATGA
ATCAAAACTTTCAACAACGGATCTCTTGGTTCTGGCATCGATGAAGAACGCAGCGAAAT
GCGATAAGTAATGTGAATTGCAGAATTCAGTGAATCATCGAATCTTTGAACGCACATTG
CGCCCGCTGGTATTCCAGCGGGCATGCCTGTTCGAGCGTCATTTCAACCCTCAAAGCTTC
GTCTTTGGTGTTGGGGCTTTACCCGTCAAAGGGTATGCCCTGAAATACAGTGGCGGGCTC
GCTAGAATTTTGAGCGTAGTAATTTATACCTCGTTTGTAAAGACTAGTGGGACTTCTAGC
CAGAAAACTCCCCCCATTTTTTGAAAAATTGACCTCGGATCAGGTAGGAATACCCGCTG
AACTTAAGCATATCAATAAGCGGAGGAAAAGAAACCAACAGGGATTGCCCCAGTAACG
GCGAGTGAAGCGGCAACAGCTCAAATTTGAAATCTGGCTTCGGCCCGAGTTGTAATTTG
CAGAGGAAGTATTTGGCGCGGTGCCTTCCGAGTTCCCTGGAACGGGACGCCATAGAGGG
TGAGAGCCCCGTCTGGTTGGACACCAAGCCTGTGTAATACTCCTTCGACGAGTCGAGTA
GTTTGGGAATGCTGCTCTAAATGGGAGGTAAATCTCTTCTAAAGCTAAATACCGGCCAG
AGACCGATAGCGCACAAGTAGAGTGATCGAAAGATGAAAAGCACCTTGAAAAGGGGGT
TAAACAGTACGTGAAATTGTTGAAAGGGAAGCGTTTATGACCAGACTTGTGCCGTGTGG
CTCATCCGGGGTTCTCCCCGGTGCACTCCACACGGTTCAGGCCAACATCGGTTCTCGTTG
GGGGATAAGAACAGTAGGAACGTGGCCCTCTTCGGAGGGTGTTATAGCCTATTGTACGA
TACCTTGATGGGGACCGAGGACCGCGCTTCGGCTAGGATGTTGGCGTAATGGTCATTAG
CGACCCGTCTTGAAACACGGACCAAGGAGTCGTCCATTAGAGCGAGCGTTTGGGTGTAA
AACCCGCACGCGTAATGAAAGTGAAATTAGGTGAGAGCTTCGGCGCATCATCGACCGAT
CCTGAAGTTTACGGATGGATTTGAGTAAGAGTTTTAACGGACGGACCCGAAAGACAGTG
AACTATACTTGAATAGGGTGAAGCCAGAGGAAACTCTGGTGGAGGCTCGCAGCGGTTCT
GACGTGCAAATCGATCGTCAAATTTGAGTATGGGGGCGAAAGACTAATCGAACCACACC
GCCCGTCGCTACTACCGATTGAATGGTTCAGTGAGGCGTTGGGACTGGCCTAGGGAGGT
CGGCAACGACCACCCAGGGCCGGAAACTTCTCCAAACTCGATCATTTAGAGGAAGTAAA
AGTCGTAACAAGGTCTCCGTTGGTGAACCAGCGGAGGGATCATTGCTGGAATAAACGCC
CTCACGGGTGCTACCCAGAAACCCTTTGTGAATTATACCAAACCGTTGCCTCGGCATTAG
CTGAATCTTTTCAAGGGATTCCCTTTTTCTTCGGAGAAAGGAGCAGGCTGGCCGGTGGCC
CTATAAACTCTTATTGTTAAAATAGTACTCTGAGTAAAACAACTATAAATGAATCAAAA
CTTTCAACAACGGATCTCTTGGTTCTGGCATCGATGAAGAACGCAGCGAAATGCGATAA
GTAATGTGAATTGCAGAATTCAGTGAATCATCGAATCTTTGAACGCACATTGCGCCCGCT
GGTATTCCAGCGGGCATGCCTGTTCGAGCGTCATTTCAACCCTCAAAGCTTCGTCTTTGG

TGTTGGGGCTTTACCCGTCAAAGGGTATGCCCTGAAATACAGTGGCGGGCTCGCTAGAA
TTTTGAGCGTAGTAATTTATACCTCGTTTGTAAAGACTAGTGGGACTTCTAGCCAGAAAA
CTCCCCCCATTTTTTGAAAAATTGACCTCGGATCAGGTAGGAATACCCGCTGAACTTAAG
CATATCAATAAGCGGAGGAAAAGAAACCAACAGGGATTGCCCCAGTAACGGCGAGTGA
AGCGGCAACAGCTCAAATTTGAAATCTGGCTTCGGCCCGAGTTGTAATTTGCAGAGGAA
GTATTTGGCGCGGTGCCTTCCGAGTTCCCTGGAACGGGACGCCATAGAGGGTGAGAGCC
CCGTCTGGTTGGACACCAAGCCTGTGTAATACTCCTTCGACGAGTCGAGTAGTTTGGGA
ATGCTGCTCTAAATGGGAGGTAAATCTCTTCTAAAGCTAAATACCGGCCAGAGACCGAT
AGCGCACAAGTAGAGTGATCGAAAGATGAAAAGCACCTTGAAAAGGGGGTTAAACAGT
ACGTGAAATTGTTGAAAGGGAAGCGTTTATGACCAGACTTGTGCCGTGTGGCTCATCCG
GGGTTCTCCCCGGTGCACTCCACACGGTTCAGGCCAACATCGGTTCTCGTTGGGGGATA
AGAACAGTAGGAACGTGGCCCTCTTCGGAGGGTGTTATAGCCTATTGTACGATACCTTG
ATGGGGACCGAGGACCGCGCTTCGGCTAGGATGTTGGCGTAATGGTCATTAGCGACCCG
TCTTGAAACACGGACCAAGGAGTCGTCCATTAGAGCGAGCGTTTGGGTGTAAAACCCGC
ACGCGTAATGAAAGTGAAATTAGGTGAGAGCTTCGGCGCATCATCGACCGATCCTGAAG
TTTACGGATGGATTTGAGTAAGAGTTTTAACGGACGGACCCGAAAGACAGTGAACTATA
CTTGAATAGGGTGAAGCCAGAGGAAACTCTGGTGGAGGCTCGCAGCGGTTCTGACGTGC
AAATCGATCGTCAAATTTGAGTATGGGGGCGAAAGACTAATCGAACGGTCCTCTGCTGG
CCAAGCTTTTCCGGAACATTGTCAAGAAGCTGACGGGTGACATGATGGCATACATGAAG
CGATGTGTCAGCGAGGGCAAGCACTTCGATCTCGCCTTGGGTATTCGCCACTCTACCCTC
ACAAACGGTCTCAAGTACTCCCTTGCAACTGGAAACTGGGGCGAGCAAAAGAAGGCTA
GCAGCTCTACGGCAGGTGTGTCTCAGGTGCTCAACCGATACACATTCTCGTCCACTCTTT
CTCATTTGCGCCGCACCAACACTCCGATTGGACGTGATGGCAAATTGGCCAAGCCTCGC
CAGCTGCACAACACGCATTGGGGTCTCGTCTGCCCCGCAGAAACCCCCGAAGGTCAAGC
TTGTGGTCTCGTCAAGAACCTGTCGCTCATGTGTTATGTCAGTGTCGGTTCCCCTGCCGA
GCCCATCAAGGAGTTCATGGTGCAGCGCAACATGGAGCTGCTCGAGGAATACGAGCCTG
GCACCAGCCCCGACTCCACCAAGGTTTTCATCAACGGTACCTGGGTTGGAGTGCACAAC
GAGCCGGCACATTTGGTGCAGTTGGTACAAGAGCTTCGACGTCGATGTATCATCTCGCA
CGAAGTTTCCCTTGTCCGCGAGATCCGCGACCGCGAGTTCAAGATCTTCTCCGACGCAG
GGCGTGTGATGCGACCCTTGTTCGTGATCGATCAGCACGACAACCCCGAAACCGGTGCG
CAACAGGGTACTTTGGCTCTCACCAAGAACCACATCCGCCGTCTGGAAGAGGACGCCCA
GTACCACCGCAAGAAGGACGACGAGGACTACTTCGGTTGGGACGGACTCCAGAACAGC
GGTGTGATCGAGTACTTAGACGCTGAGGAAGAGGAGTCGGCCATGATTTGCATGAGTCC
AGAGGATCTTGAGGACTACCGGCAGCGGAAGGCCCGAGGAAAGGTCGACCCAGACCAG
GAAACCGCACCAGAGGATGACGGCAGAAGCCTGAACGCTCGTGTCAAGACCAAGATCA
ATCTTGACATCCACCAGTACACTCACTGTGAGATTCATCCCAGCATGCTTTTGGGAATTT
GCGCCAGCATCATTCCCTTCCCAGACCACAACCAGGTATGTACAAGTTTTCGAGTATCTG
ACATCGCATTTGCTGACCAAGAGCACAGTCGCCTCGGATCCATTCTACCACCGACATCG
CGCTCCAAACGCCACGGGCGAGCAGCTGCGATCGCGAGATTGCGGCCGTTTGCCTTGGC
TTGGCTGGTCTTTTTTTGCCCAACGCACATTTTTCGCTGTGGTGCGGGGTTTGCGTTTGTC
CTTCTTATCTGAGATAAACAAACGTGACCCACCCAAACCCAACCGCCACCTCAGCCATC
CCGCACCGACACATCAATACCCATGAGCACACCCTCCTCCCTTACTTGTTTCAATTGACA
CCACTATGATGCTAATTCGATTTTCACTGCAGCTGCTGAGCTCGGAAAGGGTTCCTTCAA
GTACGCCTGGGTTCTTGACAAGCTGAAGGCTGAGCGTGAGCGTGGAATCACCATCGACA
TCGCTCTCTGGAAGTTCGAGACCCCCAAGTACTATGTCACCGTCATTGGTATGTTCACAT
TCTCTGATATCCCTACTGGCTGTGTCGTGCAGGCTCGGCAGTTTTGCTGCCCCACCACGC
GCGGCCCCTCGCTAGGCAATCTGCTCCGCGACTGTTGCTAACGATGTGTTCCTCCAGACG

CTCCCGGTCACCGTGACTTCATCAAGAACATGATCACTGGTACTTCCCAGGCCGACTGCG
CTGTTCTCATCATTGCCTCCGGCACTGGTGAGTTCGAGGCTGGTATCTCCAAGGATGGCC
AGACTCGTGAGCACGCCCTGCTCGCCTACACCCTCGGTGTCAAGCAGCTCATCGTTGCTT
GCAACAAGATGGACACTGCCGAGTGGAAGCAGGCCCGTTTCGAGGAGATCCAGAAGGA
GACCTCCACCTTCATCAAGAAGGTCGGCTACAACCCCAAGACCGTCGCCTTCGTCCCCAT
CTCCGGCTTCAACGGTGACAACATGATCGAGGGTGAGACTCTCGACCCCCGTGGCAAGG
CCTGGTACAAGGGCTGGAAGAAGCTCGGTTCCGACGGCAAGGAGGTCTCCGGAAAGAC
CCTGCTCGACGCCATTGATGCCATCGAGCCCCCCAAGCGTCCTACCGACAAGCCCCTGC
GTCTGCCCCTCCAGGACGTGTACAAGATCGGCGGTATTGGCACAGTACCCGTCGGCCGT
ATCGAGACTGGTATCCTCAAGCCCGGTATGGTCGTTACCTTCGCTCCCGCCAACGTGACC
ACTGAAGTCAAGTCCGTTGAGATGCACCACGAGCAGCTCACCGAGGGTGTTCCCGGTGA
CAACGTCGGCTTCAACGTCAAGAACGTCTCCGTCAAGGAGATTCGTCGTGGCAACGTTG
CCGGTGACTCCAAGAACGACCCCCCCGTCGGCGCCGCCTCCTTCAACGCCCAGGTCATC
GTCCTCAACCACCCCGGTCAGGTCGGTGCTGGTTAC

>Melanconiella_carpinicola

CACACCGCCCGTCGCTACTACCGATTGAATGGTTCAGTGAGGCGTTCGGACTGGCCCAG
GGAGGTCGGCAACGACCACCCAGGGCCGGAAAGTTCTCCAAACTCGATCATTTAGAGGA
AGTAAAAGTCGTAACAAGGTCTCCGTTGGTGAACCAGCGGAGGGATCATTGCTGAATAT
CCGTATTCGCCACCCTTTGTGAATATATACCTATCTCGTTGCCTCGGCGCCAGGCCCGGG
GTTCCCCCCTAGGGGGTTCCCCCCACCTGTGGACCTCACGGTCCCGGGCGGAGCAGGCC
CGCCGGCGGCCCACCAAAACTCTTGTTTGTAGAACCTATCTCTCTGAGTCTCATACGTAA
AATGAATCAAAACTTTCAACAACGGATCTCTTGGCTCTGGCATCGATGAAGAACGCAGC
GAAATGCGATAAGTAATGTGAATTGCAGAATTCAGTGAATCATCGAATCTTTGAACGCA
CATTGCGCCCGCTGGAATTCCGGCGGGCATGCCTGTTCGAGCGTCATTTCAACCCCTCAA
GCTTCAGCGCTTGGTGTTGGGGCATAGCCCGTGAAAAGGCTAGCCCTTAAATTCAGTGG
CGTGCTCGCTGAGACCCCCGGGTGAAGTAATCTCCTTCTCGCCCGGGCCGGCTCGGCGG
TGCACTCGCCGTAAAACCCGCTATCACGCACCTTCTGAAAGTTGACCTCGGATCAGGTA
GGAATACCCGCTGAACTTAAGCATATCAATAAGCGGAGGAAAAGAAACCAACAGGGAT
TGCCCTAGTAACGGCGAGTGAAGCGGCAACAGCTCAAATTTGAAATCTGGCCCCCCCGG
GGGTCCGAGTTGTAATTTGCAGAGGATGTCTTCTGGCGCGGTGCCTTCCGAGTTCCCTGG
AACGGGACGCCACAGAGGGTGAGAGCCCCGTAATGGTCGGATACCAAGCCTGTGTGAA
GCTCCTTCGACGAGTCGAGTAGTTTGGGAATGCTGCTCTAAATGGGAGGTAAATCTCTTC
TAAAGCTAAATATTGGCCAGAGACCGATAGCGCACAAGTAGAGTGATCGAAAGATGAA
AAGCACCTTGAAAAGGGGGTTAAACAGTACGTGAAATTGTTGAAAGGGAAGCGCTCAT
GACCAGACTTGCGCCGGGCGGCTCAGCAGGGGTTCCGCCCCTGTGTACTCCGTCCCGGT
TCAGGCCAGCATCGGTTCTCGCTGGGGGAAAAGAACGGCGGGAACGTGGCCCTCCCTCG
GGGGGGTGTTATAGCCCGTCGTCCGATACCCTGGCGGGGTCCGAGGTTCGCGCATTCGC
AAGGATGCTGGCGTAATGGTTATCAGTGACCCGTCTTGAAACACGGACCAAGGAGTCGT
CCTTTAGAGCGAGCGTCTGGGTGTTAAAACCCGCACGCGTAATGAAAGTGAAATTAGGT
GAGAGCCCTTACGGGCGCATCATCGACCGATCCTGATGTTCTCGGACGGATTTGAGTAA
GAGTTCTAACGGACGGACCCGAAAGACAGTGAACTATGCTTGTATAGGGTGAAGCCAG
AGGAAACTCTGGTGGAGGCTCGCAGCGGTTCTGACGTGCAAATCGATCGTCAAATATGA
GCATGGGGGCGAAAGACTAATCGAACCACACCGCCCGTCGCTACTACCGATTGAATGGT
TCAGTGAGGCGTTCGGACTGGCCCAGGGAGGTCGGCAACGACCACCCAGGGCCGGAAA
GTTCTCCAAACTCGATCATTTAGAGGAAGTAAAAGTCGTAACAAGGTCTCCGTTGGTGA
ACCAGCGGAGGGATCATTGCTGAATATCCGTATTCGCCACCCTTTGTGAATATATACCTA

TCTCGTTGCCTCGGCGCCAGGCCCGGGGTTCCCCCCTAGGGGGTTCCCCCCACCTGTGGA
CCTCACGGTCCCGGGCGGAGCAGGCCCGCCGGCGGCCCACCAAAACTCTTGTTTGTAGA
ACCTATCTCTCTGAGTCTCATACGTAAAATGAATCAAAACTTTCAACAACGGATCTCTTG
GCTCTGGCATCGATGAAGAACGCAGCGAAATGCGATAAGTAATGTGAATTGCAGAATTC
AGTGAATCATCGAATCTTTGAACGCACATTGCGCCCGCTGGAATTCCGGCGGGCATGCC
TGTTCGAGCGTCATTTCAACCCCTCAAGCTTCAGCGCTTGGTGTTGGGGCATAGCCCGTG
AAAAGGCTAGCCCTTAAATTCAGTGGCGTGCTCGCTGAGACCCCCGGGTGAAGTAATCT
CCTTCTCGCCCGGGCCGGCTCGGCGGTGCACTCGCCGTAAAACCCGCTATCACGCACCTT
CTGAAAGTTGACCTCGGATCAGGTAGGAATACCCGCTGAACTTAAGCATATCAATAAGC
GGAGGAAAAGAAACCAACAGGGATTGCCCTAGTAACGGCGAGTGAAGCGGCAACAGCT
CAAATTTGAAATCTGGCCCCCCCGGGGGTCCGAGTTGTAATTTGCAGAGGATGTCTTCTG
GCGCGGTGCCTTCCGAGTTCCCTGGAACGGGACGCCACAGAGGGTGAGAGCCCCGTAAT
GGTCGGATACCAAGCCTGTGTGAAGCTCCTTCGACGAGTCGAGTAGTTTGGGAATGCTG
CTCTAAATGGGAGGTAAATCTCTTCTAAAGCTAAATATTGGCCAGAGACCGATAGCGCA
CAAGTAGAGTGATCGAAAGATGAAAAGCACCTTGAAAAGGGGGTTAAACAGTACGTGA
AATTGTTGAAAGGGAAGCGCTCATGACCAGACTTGCGCCGGGCGGCTCAGCAGGGGTTC
CGCCCCTGTGTACTCCGTCCCGGTTCAGGCCAGCATCGGTTCTCGCTGGGGGAAAAGAA
CGGCGGGAACGTGGCCCTCCCTCGGGGGGGTGTTATAGCCCGTCGTCCGATACCCTGGC
GGGGTCCGAGGTTCGCGCATTCGCAAGGATGCTGGCGTAATGGTTATCAGTGACCCGTC
TTGAAACACGGACCAAGGAGTCGTCCTTTAGAGCGAGCGTCTGGGTGTTAAAACCCGCA
CGCGTAATGAAAGTGAAATTAGGTGAGAGCCCTTACGGGCGCATCATCGACCGATCCTG
ATGTTCTCGGACGGATTTGAGTAAGAGTTCTAACGGACGGACCCGAAAGACAGTGAACT
ATGCTTGTATAGGGTGAAGCCAGAGGAAACTCTGGTGGAGGCTCGCAGCGGTTCTGACG
TGCAAATCGATCGTCAAATATGAGCATGGGGGCGAAAGACTAATCGAACGGTCCGCTCC
TGGCGAAGCTGTTCCGAAACATCGTCAAGAAGCTGACCGGCGACATGATGGCGTACATG
AAGCGTTGCGTCAGCGAGGGCAAGCATTTCGATCTAGCGTTGGGCATCCGCCACTCAAC
CCTCACAAATGGTCTCAAGTACTCCCTTGCGACTGGAAACTGGGGCGAGCAGAAGAAGG
CGATGAGTTCCACCGCTGGTGTCTCGCAGGTGCTGAACCGGTACACATTCTCGTCCACCC
TATCGCATTTGCGGCGCACGAACACGCCTATCGGTCGTGACGGCAAGCTGGCCAAGCCT
CGTCAGCTGCACAATACACACTGGGGTCTCGTCTGTCCAGCAGAGACACCCGAGGGACA
GGCTTGCGGTCTTGTCAAGAACCTGTCTTTGATGTGCTACGTTAGTGTTGGTTCACCGGC
TGAGCCTATCAAGGACTTCATGGTGCAGCGTAATATGGAGGTCTTGGAGGAATACGAGC
CCGGCTCCAGCCCTGACGCAACCAAAATCTTCATCAACGGCACGTGGGTTGGTGTGCAC
AGTGAACCAGCACACTTGGTCACACTTGTGCAGGAGCTTCGGCGCAAATGCATCATTTC
TCACGAAGTCTCCCTCGTCCGCGACATTCGCGACCGCGAGTTCAAGATCTTCTCCGACGC
TGGTCGTGTCATGCGGCCTCTCTTCGTTGTTAATCAAGATGATAATCCCGAGACTGGAGC
GCAGCAGGGCACTCTGGCTCTCACCAAGCACCACATTCGCCGTCTCGAGGAAGATGCTC
AGTACCAGCGCAAGAAGCATGATGACGACTATTTCGGCTGGGATGGTCTTCAGAACAGC
GGTTGCATCGAGTACCTGGACGCTGAGGAAGAGGAGACATCAATGATCTGCATGAGTCC
CGAGGATCTCGAGGACTACCGCCAGCGCAAGGCTAGGGGCAAGGATGCCAAGGATGAT
GAACCGGAGGACGATGGCAGGAGTCTGAACGCTCGTGTGAAGACGAAGATCAACCCCG
ACATCCACATGTACACCCACTGCGAGATCCATCCAAGCATGCTCCTGGGAATATGCGCC
AGCATTATTCCCTTCCCTGATCACAATCAGGTATGTGAAATTCTCGCCGTACTATTATCA
CTCCTGCTGACCACATATATCTAGTCACCTCGCATCAATCCAATCAGCACCATCGCATCA
CCATCGCGCAAACATCAAGATCCAGCCAAAAGAGGTCGCGATATCTTCGTCGCGGTCGT
TTGCCCTCGCCCGCTGGCGGTTGTCCTTTTTTCGCCCGCCGCACAGTTTTGTTTTGTTGTG
CGGGGTTTTGCGCCTGGGTCTTATCTCAGATAAACGTGACCCACCCAACCGCCACCTGCC

TCATCCTCCGCCAACACCCTTTTGCGCGCGCCTCTTTCCCACCCTCAAAATCGACGATG
ACAATGCTGACTCACATTTCTACTGCAGCTGCTGAGCTCGGCAAGGGTTCCTTCAAGTA
CGCATGGGTCCTTGACAAGCTCAAGGCCGAGCGTGAGCGTGGTATCACCATCGACATTG
CTCTGTGGAAGTTCGAGACTCCCAAGTACTATGTCACCGTCATTGGTAAGCTCTATCCCA
TGCAGATGCCGCGCAGGTCTGGCTGCCCTGTCTCCCTGCGCTGCCACGCCATGCGCGAC
ACCGCGGGTTCTGATGTTTTTGGCAACCCGTGCTAACGCAAAATTCACACCAGACGCTC
CCGGTCACCGTGACTTCATCAAGAACATGATCACTGGTACCTCCCAGGCTGACTGCGCC
GTTCTCATCATTGCCTCCGGTACTGGTGAGTTCGAGGCTGGTATCTCCAAGGATGGCCAG
ACCCGTGAGCACGCTCTGCTCGCCTACACCCTCGGTGTCAAGCAGCTCATTGTTGCTTGC
AACAAGATGGACACCGCTGGCTGGAAGCAGGACCGTTTCGAGGAGATCAAGAAGGAGA
CCACCAACTTCATCAAGAAGGTCGGCTTCAACCCCAAGCAGGTCGCTTTCGTCCCCATCT
CCGGCTTCCACGGCGACAACATGCTTGAGGCCTCCTCCAACATGCCCTGGTACAAGGGA
TGGACCAAGGAGACCCAGGACAAGAAGGAGGCCAAGGGTATGACCTTGCTCGACGCCA
TTGACGCCATCGAGCCCCCCAAGCGCCCCACCGACAAGCCCCTGCGTCTGCCCCTCCAG
GATGTCTACAAGATCGGCGGTATCGGAACTGTACCTGTCGGCCGTATCGAGACTGGTAT
CCTGAAGCCCGGTATGGTCGTCACCTTCGCCCCCTCCAACGTCACCACTGAGGTCAAGTC
TGTGGAGATGCACCACGAGCAGCTCACCGAGGGCCAGCCCGGTGACAACGTTGGTTTCA
ACGTCAAGAACGTCTCCGTCAAGGAGATTCGCCGTGGCAACGTCGCTGGTGACTCCAAG
AACGACCCCCCCCTGGGCGCCGCTTCCTTCAACGCCCAGGTCATTGTCCTGAACCACCCC
GGCCAGGTCGGTGCTGGATAC

>Melanconiella_carpinicola

CACACCGCCCGTCGCTACTACCGATTGAATGGTTCAGTGAGGCGTTCGGACTGGCCCAG
GGAGGTCGGCAACGACCACCCAGGGCCGGAAAGTTCTCCAAACTCGATCATTTAGAGGA
AGTAAAAGTCGTAACAAGGTCTCCGTTGGTGAACCAGCGGAGGGATCATTGCTGAATAT
CCGTATTCGCCACCCTTTGTGAATATATACCTATCTCGTTGCCTCGGCGCCAGGCCCGGG
GTTCCCCCCTAGGGGGTTCCCCCCACCTGTGGACCTCACGGTCCCGGGCGGAGCAGGCC
CGCCGGCGGCCCACCAAAACTCTTGTTTGTAGAACCTATCTCTCTGAGTCTCATACGTAA
AATGAATCAAAACTTTCAACAACGGATCTCTTGGCTCTGGCATCGATGAAGAACGCAGC
GAAATGCGATAAGTAATGTGAATTGCAGAATTCAGTGAATCATCGAATCTTTGAACGCA
CATTGCGCCCGCTGGAATTCCGGCGGGCATGCCTGTTCGAGCGTCATTTCAACCCCTCAA
GCTTCAGCGCTTGGTGTTGGGGCATAGCCCGTGAAAAGGCTAGCCCTTAAATTCAGTGG
CGTGCTCGCTGAGACCCCCGGGTGAAGTAATCTCCTTCTCGCCCGGGCCGGCTCGGCGG
TGCACTCGCCGTAAAACCCGCTATCACGCACCTTCTGAAAGTTGACCTCGGATCAGGTA
GGAATACCCGCTGAACTTAAGCATATCAATAAGCGGAGGAAAAGAAACCAACAGGGAT
TGCCCTAGTAACGGCGAGTGAAGCGGCAACAGCTCAAATTTGAAATCTGGCCCCCCCGG
GGGTCCGAGTTGTAATTTGCAGAGGATGTCTTCTGGCGCGGTGCCTTCCGAGTTCCCTGG
AACGGGACGCCACAGAGGGTGAGAGCCCCGTAATGGTCGGATACCAAGCCTGTGTGAA
GCTCCTTCGACGAGTCGAGTAGTTTGGGAATGCTGCTCTAAATGGGAGGTAAATCTCTTC
TAAAGCTAAATATTGGCCAGAGACCGATAGCGCACAAGTAGAGTGATCGAAAGATGAA
AAGCACCTTGAAAAGGGGGTTAAACAGTACGTGAAATTGTTGAAAGGGAAGCGCTCAT
GACCAGACTTGCGCCGGGCGGCTCAGCAGGGGTTCCGCCCCTGTGTACTCCGTCCCGGT
TCAGGCCAGCATCGGTTCTCGCTGGGGGAAAAGAACGGCGGGAACGTGGCCCTCCCTCG
GGGGGGTGTTATAGCCCGTCGTCCGATACCCTGGCGGGGTCCGAGGTTCGCGCATTCGC
AAGGATGCTGGCGTAATGGTTATCAGTGACCCGTCTTGAAACACGGACCAAGGAGTCGT
CCTTTAGAGCGAGCGTCTGGGTGTTAAAACCCGCACGCGTAATGAAAGTGAAATTAGGT
GAGAGCCCTTACGGGCGCATCATCGACCGATCCTGATGTTCTCGGACGGATTTGAGTAA

GAGTTCTAACGGACGGACCCGAAAGACAGTGAACTATGCTTGTATAGGGTGAAGCCAG
AGGAAACTCTGGTGGAGGCTCGCAGCGGTTCTGACGTGCAAATCGATCGTCAAATATGA
GCATGGGGGCGAAAGACTAATCGAACCACACCGCCCGTCGCTACTACCGATTGAATGGT
TCAGTGAGGCGTTCGGACTGGCCCAGGGAGGTCGGCAACGACCACCCAGGGCCGGAAA
GTTCTCCAAACTCGATCATTTAGAGGAAGTAAAAGTCGTAACAAGGTCTCCGTTGGTGA
ACCAGCGGAGGGATCATTGCTGAATATCCGTATTCGCCACCCTTTGTGAATATATACCTA
TCTCGTTGCCTCGGCGCCAGGCCCGGGGTTCCCCCCTAGGGGGTTCCCCCCACCTGTGGA
CCTCACGGTCCCGGGCGGAGCAGGCCCGCCGGCGGCCCACCAAAACTCTTGTTTGTAGA
ACCTATCTCTCTGAGTCTCATACGTAAAATGAATCAAAACTTTCAACAACGGATCTCTTG
GCTCTGGCATCGATGAAGAACGCAGCGAAATGCGATAAGTAATGTGAATTGCAGAATTC
AGTGAATCATCGAATCTTTGAACGCACATTGCGCCCGCTGGAATTCCGGCGGGCATGCC
TGTTCGAGCGTCATTTCAACCCCTCAAGCTTCAGCGCTTGGTGTTGGGGCATAGCCCGTG
AAAAGGCTAGCCCTTAAATTCAGTGGCGTGCTCGCTGAGACCCCCGGGTGAAGTAATCT
CCTTCTCGCCCGGGCCGGCTCGGCGGTGCACTCGCCGTAAAACCCGCTATCACGCACCTT
CTGAAAGTTGACCTCGGATCAGGTAGGAATACCCGCTGAACTTAAGCATATCAATAAGC
GGAGGAAAAGAAACCAACAGGGATTGCCCTAGTAACGGCGAGTGAAGCGGCAACAGCT
CAAATTTGAAATCTGGCCCCCCCGGGGGTCCGAGTTGTAATTTGCAGAGGATGTCTTCTG
GCGCGGTGCCTTCCGAGTTCCCTGGAACGGGACGCCACAGAGGGTGAGAGCCCCGTAAT
GGTCGGATACCAAGCCTGTGTGAAGCTCCTTCGACGAGTCGAGTAGTTTGGGAATGCTG
CTCTAAATGGGAGGTAAATCTCTTCTAAAGCTAAATATTGGCCAGAGACCGATAGCGCA
CAAGTAGAGTGATCGAAAGATGAAAAGCACCTTGAAAAGGGGGTTAAACAGTACGTGA
AATTGTTGAAAGGGAAGCGCTCATGACCAGACTTGCGCCGGGCGGCTCAGCAGGGGTTC
CGCCCCTGTGTACTCCGTCCCGGTTCAGGCCAGCATCGGTTCTCGCTGGGGGAAAAGAA
CGGCGGGAACGTGGCCCTCCCTCGGGGGGGTGTTATAGCCCGTCGTCCGATACCCTGGC
GGGGTCCGAGGTTCGCGCATTCGCAAGGATGCTGGCGTAATGGTTATCAGTGACCCGTC
TTGAAACACGGACCAAGGAGTCGTCCTTTAGAGCGAGCGTCTGGGTGTTAAAACCCGCA
CGCGTAATGAAAGTGAAATTAGGTGAGAGCCCTTACGGGCGCATCATCGACCGATCCTG
ATGTTCTCGGACGGATTTGAGTAAGAGTTCTAACGGACGGACCCGAAAGACAGTGAACT
ATGCTTGTATAGGGTGAAGCCAGAGGAAACTCTGGTGGAGGCTCGCAGCGGTTCTGACG
TGCAAATCGATCGTCAAATATGAGCATGGGGGCGAAAGACTAATCGAACGGTCCGCTCC
TGGCGAAGCTGTTCCGAAACATCGTCAAGAAGCTGACCGGCGACATGATGGCGTACATG
AAGCGTTGCGTCAGCGAGGGCAAGCATTTCGATCTAGCGTTGGGCATCCGCCACTCAAC
CCTCACAAATGGTCTCAAGTACTCCCTTGCGACTGGAAACTGGGGCGAGCAGAAGAAGG
CGATGAGTTCCACCGCTGGTGTCTCGCAGGTGCTGAACCGGTACACATTCTCGTCCACCC
TATCGCATTTGCGGCGCACGAACACGCCTATCGGTCGTGACGGCAAGCTGGCCAAGCCT
CGTCAGCTGCACAATACACACTGGGGTCTCGTCTGTCCAGCAGAGACACCCGAGGGACA
GGCTTGCGGTCTTGTCAAGAACCTGTCTTTGATGTGCTACGTTAGTGTTGGTTCACCGGC
TGAGCCTATCAAGGACTTCATGGTGCAGCGTAATATGGAGGTCTTGGAGGAATACGAGC
CCGGCTCCAGCCCTGACGCAACCAAAATCTTCATCAACGGCACGTGGGTTGGTGTGCAC
AGTGAACCAGCACACTTGGTCACACTTGTGCAGGAGCTTCGGCGCAAATGCATCATTTC
TCACGAAGTCTCCCTCGTCCGCGACATTCGCGACCGCGAGTTCAAGATCTTCTCCGACGC
TGGTCGTGTCATGCGGCCTCTCTTCGTTGTTAATCAAGATGATAATCCCGAGACTGGAGC
GCAGCAGGGCACTCTGGCTCTCACCAAGCACCACATTCGCCGTCTCGAGGAAGATGCTC
AGTACCAGCGCAAGAAGCATGATGACGACTATTTCGGCTGGGATGGTCTTCAGAACAGC
GGTTGCATCGAGTACCTGGACGCTGAGGAAGAGGAGACATCAATGATCTGCATGAGTCC
CGAGGATCTCGAGGACTACCGCCAGCGCAAGGCTAGGGGCAAGGATGCCAAGGATGAT
GAACCGGAGGACGATGGCAGGAGTCTGAACGCTCGTGTGAAGACGAAGATCAACCCCG

ACATCCACATGTACACCCACTGCGAGATCCATCCAAGCATGCTCCTGGGAATATGCGCC
AGCATTATTCCCTTCCCTGATCACAATCAGGTATGTGAAATTCTCGCCGTACTATTATCA
CTCCTGCTGACCACATATATCTAGTCACCTCGCATCAATCCAATCAGCACCATCGCATCA
CCATCGCGCAAACATCAAGATCCAGCCAAAAGAGGTCGCGATATCTTCGTCGCGGTCGT
TTGCCCTCGCCCGCTGGCGGTTGTCCTTTTTCGCCCGCCGCACAGTTTTGTTTTGTTGTG
CGGGGTTTTGCGCCTGGGTCTTATCTCAGATAAACGTGACCCACCCAACCGCCACCTGCC
TCATCCTCCGCCAACACCCTTTTTGCGCGCGCCTCTTTCCCACCCTCAAAATCGACGATG
ACAATGCTGACTCACATTTTCTACTGCAGCTGCTGAGCTCGGCAAGGGTTCCTTCAAGTA
CGCATGGGTCCTTGACAAGCTCAAGGCCGAGCGTGAGCGTGGTATCACCATCGACATTG
CTCTGTGGAAGTTCGAGACTCCCAAGTACTATGTCACCGTCATTGGTAAGCTCTATCCCA
TGCAGATGCCGCGCAGGTCTGGCTGCCCTGTCTCCCTGCGCTGCCACGCCATGCGCGAC
ACCGCGGGTTCTGATGTTTTTTGGCAACCCGTGCTAACGCAAAATTCACACCAGACGCTC
CCGGTCACCGTGACTTCATCAAGAACATGATCACTGGTACCTCCCAGGCTGACTGCGCC
GTTCTCATCATTGCCTCCGGTACTGGTGAGTTCGAGGCTGGTATCTCCAAGGATGGCCAG
ACCCGTGAGCACGCTCTGCTCGCCTACACCCTCGGTGTCAAGCAGCTCATTGTTGCTTGC
AACAAGATGGACACCGCTGGCTGGAAGCAGGACCGTTTCGAGGAGATCAAGAAGGAGA
CCACCAACTTCATCAAGAAGGTCGGCTTCAACCCCAAGCAGGTCGCTTTCGTCCCCATCT
CCGGCTTCCACGGCGACAACATGCTTGAGGCCTCCTCCAACATGCCCTGGTACAAGGGA
TGGACCAAGGAGACCCAGGACAAGAAGGAGGCCAAGGGTATGACCTTGCTCGACGCCA
TTGACGCCATCGAGCCCCCCAAGCGCCCCACCGACAAGCCCCTGCGTCTGCCCCTCCAG
GATGTCTACAAGATCGGCGGTATCGGAACTGTACCTGTCGGCCGTATCGAGACTGGTAT
CCTGAAGCCCGGTATGGTCGTCACCTTCGCCCCCTCCAACGTCACCACTGAGGTCAAGTC
TGTGGAGATGCACCACGAGCAGCTCACCGAGGGCCAGCCCGGTGACAACGTTGGTTTCA
ACGTCAAGAACGTCTCCGTCAAGGAGATTCGCCGTGGCAACGTCGCTGGTGACTCCAAG
AACGACCCCCCCCTGGGCGCCGCTTCCTTCAACGCCCAGGTCATTGTCCTGAACCACCCC
GGCCAGGTCGGTGCTGGATAC

>Melanconiella_chrysodiscosporina

GAACCAGCGGAGGGATCATTGCTGGAAAACACGTCCCATACCCCTTTGTGAACATATAC
CTATCTCGTTGCCTCGGCGCCAGGCCCGGGGTGCCCCCCAGGGGGTACCCCCCACCTAT
GGACCTCACGGTCCCGGGCGGAGCAGGCTCGCCGGCGGCCCCTTCCAACTCTTGTTTTTA
GAACCTATCTCTTCTGAGTCTTGTTACCTAGAAATGAATCAAAACTTTCAACAACGGATC
TCTTGGCTCTGGCATCGATGAAGAACGCAGCGAAATGCGATAAGTAATGTGAATTGCAG
AATTCAGTGAACCATCGAATCTTTGAACGCACATTGCGCCCGCTGGGATTCCGGCGGGC
ATGCCTGTTCGAGCGTCATTTCAAACCCTCAGGCGTCCGCGCCTGGTGTTGGGGCATAGC
TTGTAAAAAGGCTAGCTCTTAAATTCAGTGGCGTGCTCGCTGAGACTCCCGGGTGTAGT
AATCTCCTTCTCGCCCGGGCAGGCTCGGCGGCGCACTTGCCGTAAAACCCGCTATCACG
CACTGCTTCTGAAAATTGACCTCGGATCAGGTAGGAATACCCGCTGAACTTAAGCATAT
CAATAAGCGGAGGAAAAGAAACCAACAGGGATTGCCCTAGTAACGGCGAGTGAAGCGG
CAACAGCTCAAATTTGAAATCTGGTCCCTCACGGGGCCCGAGTTGTAATTTGCAGAGGA
TGTCTTCTGGCGCGGTGCCTTCCGAGTTCCCTGGAACGGACGCCACAGAGGGTGAGAG
CCCCGTAATGGTCGGATACCAAGCCTGTGTGAAGCTCCTTCGACGAGTCGAGTAGTTTG
GGAATGCTGCTCTAAATGGGAGGTAAATCTCTTCTAAAGCTAAATACTGGCCAGAGACC
GATAGCGCACAAGTAGAGTGATCGAAAGATGAAAAGCACCTTGAAAAGGGGGTTAAAC
AGTACGTGAAATTGTTGAAAGGGAAGCGCTCATGACCAGACTTGCGCCGGGCGGCTCAT
CAGGTGGTTCTGCCCCTGTGTACTCCGTCCCGGTTCAGGCCAGCATCGGTTCGCGCCGGG
GGACAAGAACGGTGGGAACGTGGCCCCCCCTCGGGGGGGTGTTATAGCCCGCCGTGAC

GATACCCTGGTGCGGACCGAGGTTCGCGCACTCCGCAAGGATGCTGGCGTAATGGTTAT
CAGTGACCCGTCTTGAAACACGGACCAAGGAGTCGTCCTTTAGAGCGAGCGTCTGGGTG
TCAAAACCCGCACGCGTAATGAAAGTGAAATCAGGTGAGAGCCCCTCGTGGGCGCATCA
TCGACCGATCCTGATGTTCTCGGATGGATTTGAGTAAGAGTTTTAACGGACGGACCCGA
AAGACAGTGAACTATGCTTGTATAGGGTGAAGCCAGAGGAAACTCTGGTGGAGGCTCGC
AGCGGTTCTGACGTGCAAATCGATCGTCAAATATGAGCATGGGGGCGAAAGACTAATCG
AACGAACCAGCGGAGGGATCATTGCTGGAAAACACGTCCCATACCCCTTTGTGAACATA
TACCTATCTCGTTGCCTCGGCGCCAGGCCCGGGGTGCCCCCCAGGGGGTACCCCCCACCT
ATGGACCTCACGGTCCCGGGCGGAGCAGGCTCGCCGGCGGCCCCTTCCAACTCTTGTTTT
TAGAACCTATCTCTTCTGAGTCTTGTTACCTAGAAATGAATCAAAACTTTCAACAACGGA
TCTCTTGGCTCTGGCATCGATGAAGAACGCAGCGAAATGCGATAAGTAATGTGAATTGC
AGAATTCAGTGAACCATCGAATCTTTGAACGCACATTGCGCCCGCTGGGATTCCGGCGG
GCATGCCTGTTCGAGCGTCATTTCAAACCCTCAGGCGTCCGCGCCTGGTGTTGGGGCATA
GCTTGTAAAAAGGCTAGCTCTTAAATTCAGTGGCGTGCTCGCTGAGACTCCCGGGTGTA
GTAATCTCCTTCTCGCCCGGGCAGGCTCGGCGGCGCACTTGCCGTAAAACCCGCTATCAC
GCACTGCTTCTGAAAATTGACCTCGGATCAGGTAGGAATACCCGCTGAACTTAAGCATA
TCAATAAGCGGAGGAAAAGAAACCAACAGGGATTGCCCTAGTAACGGCGAGTGAAGCG
GCAACAGCTCAAATTTGAAATCTGGTCCCTCACGGGGCCCGAGTTGTAATTTGCAGAGG
ATGTCTTCTGGCGCGGTGCCTTCCGAGTTCCCTGGAACGGGACGCCACAGAGGGTGAGA
GCCCCGTAATGGTCGGATACCAAGCCTGTGTGAAGCTCCTTCGACGAGTCGAGTAGTTT
GGGAATGCTGCTCTAAATGGGAGGTAAATCTCTTCTAAAGCTAAATACTGGCCAGAGAC
CGATAGCGCACAAGTAGAGTGATCGAAAGATGAAAAGCACCTTGAAAAGGGGGTTAAA
CAGTACGTGAAATTGTTGAAAGGGAAGCGCTCATGACCAGACTTGCGCCGGGCGGCTCA
TCAGGTGGTTCTGCCCCTGTGTACTCCGTCCCGGTTCAGGCCAGCATCGGTTCGCGCCGG
GGGACAAGAACGGTGGGAACGTGGCCCCCCCTCGGGGGGGTGTTATAGCCCGCCGTGA
CGATACCCTGGTGCGGACCGAGGTTCGCGCACTCCGCAAGGATGCTGGCGTAATGGTTA
TCAGTGACCCGTCTTGAAACACGGACCAAGGAGTCGTCCTTTAGAGCGAGCGTCTGGGT
GTCAAAACCCGCACGCGTAATGAAAGTGAAATCAGGTGAGAGCCCCTCGTGGGCGCATC
ATCGACCGATCCTGATGTTCTCGGATGGATTTGAGTAAGAGTTTTAACGGACGGACCCG
AAAGACAGTGAACTATGCTTGTATAGGGTGAAGCCAGAGGAAACTCTGGTGGAGGCTC
GCAGCGGTTCTGACGTGCAAATCGATCGTCAAATATGAGCATGGGGGCGAAAGACTAAT
CGAACGGTCCGCTCCTGGCAAAGCTGTTCCGAAACATCGTCAAGAAGCTGACCGGCGAC
ATGATGGCCTACATGAAGCGTTGTGTCAGCGAGGGTAAGCATTTTGATCTAGCGTTGGG
CATCCGTCACTCGACCCTCACGAATGGCCTCAAGTATTCTCTTGCGACTGGAAACTGGGG
CGAGCAGAAGAAGGCGATGAGTTCCACCGCTGGTGTCTCGCAGGTGCTGAACCGCTACA
CGTTCTCTTCCACCCTGTCGCATTTGCGGCGCACGAACACGCCCATCGGTCGTGACGGCA
AGCTGGCCAAGCCTCGTCAGCTGCACAATACACACTGGGGCCTCGTCTGTCCAGCAGAG
ACTCCCGAGGGACAGGCTTGCGGTCTCGTCAAGAACCTCTCTTTAATGTGCTACGTTAGC
GTTGGTTCACCAGCTGAGCCTATCAAGGACTTCATGGTGCAGCGTAATATGGAAGTCTT
GGAGGAATACGAGCCCGGCTCCAGCCCTGACGCAACCAAGATATTCATCAACGGCACCT
GGGTTGGTGTACACAGTGAACCAGCACACTTGGTGACGCTTGTGCAGGAGCTTCGGCGC
AAATGCATCATTTCTCACGAAGTCTCCCTCGTTCGCGACATTCGCGACCGCGAGTTCAAG
ATCTTCTCTGACGCTGGTCGTGTCATGCGGCCACTCTTCGTTGTCAATCAAGACGATAAC
CCCGAGACTGGAGCGCAGCAGGGCACTCTAGCTCTCACCAAGCACCACATTCGCCGTCT
CGAGGAAGATGCTCAGTACCAACGCAAGAAGCATGACGACGACTATTTTGGCTGGGATG
GTCTTCAGAACAGCGGTTGTATCGAGTATCTGGATGCCGAGGAAGAGGAGACAGCGATG
ATCTGCATGAGTCCTGAGGATCTTGAGGACTACCGCCAGCGCAAGGCTAGAGGCAAGGA

TGCCAAGGATGATGAACCCGAGGACGATGGCAGAAGTCTGAACGCTCGTGTGAAGACG
AAGATTAACCCCGACATCCACATGTACACCCACTGCGAGATCCATCCGAGCATGCTCCT
GGGAATATGCGCCAGCATCATTCCCTTCCCGGATCACAATCAGGTGTGTAAAATTCTCGC
AGTACTACTATCACTCCTGCTGACCACATATATCTAGTCACCTCGCATCATCCAATCCAG
CATCACCACATCACCATCGCACAAACATCAAGATCAAGTCCAAACGGTCGCCCTTTCTTC
CTCCCGGCCGCCTGCCCTGGCCTGCTTGGGGGGAGGGGGGGTTGCCCTTTTTTCGCCCAC
CACACATTTTCGCTTTGTTGTGCGGGGTTTTGCGTCTGGGTCTTATCAGATAAACGTGAC
CCACCCAACCGCCACCTCAATCATCCTCCGCTAACACACCCTTGCGCGCGCCTCTTTTTT
GCCCTCTAATTTGACAACGACAATACTGACCCGGGTTTTTTACTGCAGCTGCTGAGCTCG
GCAAGGGTTCCTTCAAGTACGCATGGGTCCTTGACAAGCTCAAGGCCGAGCGTGAGCGT
GGTATCACCATCGACATTGCTCTGTGGAAGTTCGAGACTCCCAAGTACTATGTCACCGTC
ATTGGTAAGCTCTACGTACCCCCTGGCGGTTTTGCGCAGGCCTGCCTGCCCCGCCTCTGC
GCTGCCTCGCCTCTGCGCTGCCTCGCCTCTGCGCTGCCCCGCCACGCGCAACGCCACGGC
TCCTGGTGCTTTTGCGCAACCCGTGCTAACGCAAATGTCAACACAGACGCTCCCGGTCAC
CGTGACTTCATCAAGAACATGATCACTGGTACCTCCCAGGCTGACTGCGCCGTTCTCATC
ATTGCCTCCGGTACTGGTGAGTTCGAGGCTGGTATCTCCAAGGATGGCCAGACTCGTGA
GCACGCTCTGCTCGCCTACACCCTCGGTGTCAAGCAGCTCATTGTTGCTTGCAACAAGAT
GGACACCGCTGGCTGGAAGCAGGACCGTTTCGAGGAGATCAAGAAGGAGACCACCAAC
TTCATCAAGAAGGTTGGCTTCAACCCCAAGCAGGTTGCTTTCGTCCCCATCTCCGGTTTC
CACGGCGACAACATGCTTGAGGCTTCCGTCAACATGCCCTGGTACAAGGGCTGGACCAA
GGAGACCCAGGACAAGAAGGAGGCCAAGGGTATGACCCTGCTCGACGCCATTGACTCC
ATCGAGCCCCCCAAGCGTCCCACCGAGAAGCCCCTGCGTCTGCCCCTCCAGGATGTCTA
CAAGATCGGCGGTATCGGAACTGTACCGGTCGGCCGTATCGAGACTGGTGTCCTGAAGC
CCGGTATGGTCGTCACCTTCGCCCCCTCCAACGTCACCACTGAGGTCAAGTCCGTGGAG
ATGCACCACGAGCAGCTCACCGAGGGTCAGCCCGGTGACAACGTTGGTTTCAACGTCAA
GAACGTCTCCGTCAAGGAGATTCGCCGTGGCAACGTCGCTGGTGACTCCAAGAACGACC
CCCCCATGGCCGCCGCTTCCTTCAACGCTCAGGTCATCGTCCTGAACCACCCTGGCCAGG
TCGGTGCTGGATAC

>Melanconiella_chrysodiscosporina

CACACCGCCCGTCGCTACTACCGATTGAATGGTTCAGTGAGGCGTCCGGACTGGCCCAG
GGAGGTCGGCAACGACCACCCAGGGCCGGAAAGTTCTCCAAACTCGATCATTTAGAGGA
AGTAAAAGTCGTAACAAGGTCTCCGTTGGTGAACCAGCGGAGGGATCATTGCTGGAAAA
CACGTCCCATACCCCTTTGTGAACATATACCTATCTCGTTGCCTCGGCGCCAGGCCCGGG
GTGCCCCCCAGGGGGTACCCCCCACCTATGGACCTCACGGTCCCGGGCGGAGCAGGCTC
GCCGGCGGCCCCTTCCAACTCTTGTTTTAGAACCTATCTCTTCTGAGTCTTGTTACCTAG
AAATGAATCAAAACTTTCAACAACGGATCTCTTGGCTCTGGCATCGATGAAGAACGCAG
CGAAATGCGATAAGTAATGTGAATTGCAGAATTCAGTGAACCATCGAATCTTTGAACGC
ACATTGCGCCCGCTGGGATTCCGGCGGGCATGCCTGTTCGAGCGTCATTTCAAACCCTCA
GGCGTCCGCGCCTGGTGTTGGGGCATAGCTTGTAAAAAGGCTAGCTCTTAAATTCAGTG
GCGTGCTCGCTGAGACTCCCGGGTGTAGTAATCTCCTTCTCGCCCGGGCAGGCTCGGCG
GCGCACTTGCCGTAAAACCCGCTATCACGCACTGCTTCTGAAAATTGACCTCGGATCAG
GTAGGAATACCCGCTGAACTTAAGCATATCAATAAGCGGAGGAAAAGAAACCAACAGG
GATTGCCCTAGTAACGGCGAGTGAAGCGGCAACAGCTCAAATTTGAAATCTGGTCCCTC
ACGGGGCCCGAGTTGTAATTTGCAGAGGATGTCTTCTGGCGCGGTGCCTTCCGAGTTCCC
TGGAACGGGACGCCACAGAGGGTGAGAGCCCCGTAATGGTCGGATACCAAGCCTGTGT
GAAGCTCCTTCGACGAGTCGAGTAGTTTGGGAATGCTGCTCTAAATGGGAGGTAAATCT

CTTCTAAAGCTAAATACCGGCCAGAGACCGATAGCGCACAAGTAGAGTGATCGAAAGA
TGAAAAGCACCTTGAAAAGGGGGTTAAACAGTACGTGAAATTGTTGAAAGGGAAGCGC
TCATGACCAGACTTGCGCCGGGCGGCTCATCAGGTGGTTCTGCCCCTGTGTACTCCGTCC
CGGTTCAGGCCAGCATCGGTTCGCGCCGGGGGACAAGAACGGTGGGAACGTGGCCCCC
CCTCGGGGGGGTGTTATAGCCCGCCGTGACGATACCCTGGTGCGGACCGAGGTTCGCGC
ACTCCGCAAGGATGCTGGCGTAATGGTTATCAGTGACCCGTCTTGAAACACGGACCAAG
GAGTCGTCCTTTAGAGCGAGCGTCTGGGTGTCAAAACCCGCACGCGTAATGAAAGTGAA
ATCAGGTGAGAGCCCCTCGTGGGCGCATCATCGACCGATCCTGATGTTCTCGGATGGAT
TTGAGTAAGAGTTTTAACGGACGGACCCGAAAGACAGTGAACTATGCTTGTATAGGGTG
AAGCCAGAGGAAACTCTGGTGGAGGCTCGCAGCGGTTCTGACGTGCAAATCGATCGTCA
AATATGAGCATGGGGGCGAAAGACTAATCGAACCACACCGCCCGTCGCTACTACCGATT
GAATGGTTCAGTGAGGCGTCCGGACTGGCCCAGGGAGGTCGGCAACGACCACCCAGGG
CCGGAAAGTTCTCCAAACTCGATCATTTAGAGGAAGTAAAAGTCGTAACAAGGTCTCCG
TTGGTGAACCAGCGGAGGGATCATTGCTGGAAAACACGTCCCATACCCCTTTGTGAACA
TATACCTATCTCGTTGCCTCGGCGCCAGGCCCGGGGTGCCCCCCAGGGGGTACCCCCCA
CCTATGGACCTCACGGTCCCGGGCGGAGCAGGCTCGCCGGCGGCCCCTTCCAACTCTTG
TTTTTAGAACCTATCTCTTCTGAGTCTTGTTACCTAGAAATGAATCAAAACTTTCAACAA
CGGATCTCTTGGCTCTGGCATCGATGAAGAACGCAGCGAAATGCGATAAGTAATGTGAA
TTGCAGAATTCAGTGAACCATCGAATCTTTGAACGCACATTGCGCCCGCTGGGATTCCG
GCGGGCATGCCTGTTCGAGCGTCATTTCAAACCCTCAGGCGTCCGCGCCTGGTGTTGGG
GCATAGCTTGTAAAAAGGCTAGCTCTTAAATTCAGTGGCGTGCTCGCTGAGACTCCCGG
GTGTAGTAATCTCCTTCTCGCCCGGGCAGGCTCGGCGGCGCACTTGCCGTAAAACCCGCT
ATCACGCACTGCTTCTGAAAATTGACCTCGGATCAGGTAGGAATACCCGCTGAACTTAA
GCATATCAATAAGCGGAGGAAAAGAAACCAACAGGGATTGCCCTAGTAACGGCGAGTG
AAGCGGCAACAGCTCAAATTTGAAATCTGGTCCCTCACGGGGCCCGAGTTGTAATTTGC
AGAGGATGTCTTCTGGCGCGGTGCCTTCCGAGTTCCCTGGAACGGGACGCCACAGAGGG
TGAGAGCCCCGTAATGGTCGGATACCAAGCCTGTGTGAAGCTCCTTCGACGAGTCGAGT
AGTTTGGGAATGCTGCTCTAAATGGGAGGTAAATCTCTTCTAAAGCTAAATACCGGCCA
GAGACCGATAGCGCACAAGTAGAGTGATCGAAAGATGAAAAGCACCTTGAAAAGGGGG
TTAAACAGTACGTGAAATTGTTGAAAGGGAAGCGCTCATGACCAGACTTGCGCCGGGCG
GCTCATCAGGTGGTTCTGCCCCTGTGTACTCCGTCCCGGTTCAGGCCAGCATCGGTTCGC
GCCGGGGGACAAGAACGGTGGGAACGTGGCCCCCCCTCGGGGGGGTGTTATAGCCCGC
CGTGACGATACCCTGGTGCGGACCGAGGTTCGCGCACTCCGCAAGGATGCTGGCGTAAT
GGTTATCAGTGACCCGTCTTGAAACACGGACCAAGGAGTCGTCCTTTAGAGCGAGCGTC
TGGGTGTCAAAACCCGCACGCGTAATGAAAGTGAAATCAGGTGAGAGCCCCTCGTGGGC
GCATCATCGACCGATCCTGATGTTCTCGGATGGATTTGAGTAAGAGTTTTAACGGACGG
ACCCGAAAGACAGTGAACTATGCTTGTATAGGGTGAAGCCAGAGGAAACTCTGGTGGA
GGCTCGCAGCGGTTCTGACGTGCAAATCGATCGTCAAATATGAGCATGGGGGCGAAAGA
CTAATCGAACGGTCCGCTCCTGGCAAAGCTGTTCCGAAACATCGTCAAGAAGCTGACCG
GCGACATGATGGCCTACATGAAGCGTTGTGTCAGCGAGGGTAAGCATTTTGATCTAGCG
TTGGGCATCCGTCACTCGACCCTCACGAATGGCCTCAAGTATTCTCTTGCGACTGGAAAC
TGGGGCGAGCAGAAGAAGGCGATGAGTTCCACCGCTGGTGTCTCGCAGGTGCTGAACCG
CTACACGTTCTCTTCCACCCTGTCGCATTTGCGGCGCACGAACACGCCCATCGGTCGTGA
CGGCAAGCTGGCCAAGCCTCGTCAGCTGCACAATACACACTGGGGCCTCGTCTGTCCAG
CAGAGACTCCCGAGGGACAGGCTTGCGGTCTCGTCAAGAACCTCTCTTTAATGTGCTAC
GTTAGCGTTGGTTCACCAGCTGAGCCTATCAAGGACTTCATGGTGCAGCGTAATATGGA
AGTCTTGGAGGAATACGAGCCCGGCTCCAGCCCTGACGCAACCAAGATATTCATCAACG

GCACCTGGGTTGGTGTACACAGTGAACCAGCACACTTGGTGACGCTTGTGCAGGAGCTT
CGGCGCAAATGCATCATTTCTCACGAAGTCTCCCTCGTTCGCGACATTCGCGACCGCGAG
TTCAAGATCTTCTCTGACGCTGGTCGTGTCATGCGGCCACTCTTCGTTGTCAATCAAGAC
GATAACCCCGAGACTGGAGCGCAGCAGGGCACTCTAGCTCTCACCAAGCACCACATTCG
CCGTCTCGAGGAAGATGCTCAGTACCAACGCAAGAAGCATGACGACGACTATTTTGGCT
GGGATGGTCTTCAGAACAGCGGTTGTATCGAGTATCTGGATGCCGAGGAAGAGGAGAC
AGCGATGATCTGCATGAGTCCTGAGGATCTTGAGGACTACCGCCAGCGCAAGGCTAGAG
GCAAGGATGCCAAGGATGATGAACCCGAGGACGATGGCAGAAGTCTGAACGCTCGTGT
GAAGACGAAGATTAACCCCGACATCCACATGTACACCCACTGCGAGATCCATCCGAGCA
TGCTCCTGGGAATATGCGCCAGCATCATTCCCTTCCCGGATCACAATCAGGTGTGTAAAA
TTCTCGCAGTACTACTATCACTCCTGCTGACCACATATATCTAGTCACCTCGGCATCACC
ACATCACCATCGCACAAACATCAAGATCAAGTCCAAACGGTCGCCCTTTCTTCCTCCCGG
CCGCCTGCCCTGGCCTGCTTGGGGGGAGGGGGGGTTGCCCTTTTTTCGCCCACCACACAT
TTTCGCTTTGTTGTGCGGGGTTTTGCGTCTGGGTCTTATCAGATAAACGTGACCCACCCA
ACCGCCACCTCAATCATCCTCCGCTAACACACCCTTGCGCGCGCCTCTTTTTTGCCCTCTA
ATTTGACAACGACAATACTGACCCGGGTTTTTTACTGCAGCTGCTGAGCTCGGCAAGGG
TTCCTTCAAGTACGCATGGGTCCTTGACAAGCTCAAGGCCGAGCGTGAGCGTGGTATCA
CCATCGACATTGCTCTGTGGAAGTTCGAGACTCCCAAGTACTATGTCACCGTCATTGGTA
AGCTCTACGTACCCCCTGGCGGTTTTGCGCAGGCCTGCCTGCCCCGCCTCTGCGCTGCCT
CGCCTCTGCGCTGCCTCGCCTCTGCGCTGCCCCGCCACGCGCAACGCCACGGCTCCTGGT
GCTTTTGCGCAACCCGTGCTAACGCAAATGTCAACACAGACGCTCCCGGTCACCGTGAC
TTCATCAAGAACATGATCACTGGTACCTCCCAGGCTGACTGCGCCGTTCTCATCATTGCC
TCCGGTACTGGTGAGTTCGAGGCTGGTATCTCCAAGGATGGCCAGACTCGTGAGCACGC
TCTGCTCGCCTACACCCTCGGTGTCAAGCAGCTCATTGTTGCTTGCAACAAGATGGACAC
CGCTGGCTGGAAGCAGGACCGTTTCGAGGAGATCAAGAAGGAGACCACCAACTTCATC
AAGAAGGTTGGCTTCAACCCCAAGCAGGTTGCTTTCGTCCCCATCTCCGGTTTCCACGGC
GACAACATGCTTGAGGCTTCCGTCAACATGCCCTGGTACAAGGGCTGGACCAAGGAGAC
CCAGGACAAGAAGGAGGCCAAGGGTATGACCCTGCTCGACGCCATTGACTCCATCGAGC
CCCCCAAGCGTCCCACCGAGAAGCCCCTGCGTCTGCCCCTCCAGGATGTCTACAAGATC
GGCGGTATCGGAACTGTACCGGTCGGCCGTATCGAGACTGGTGTCCTGAAGCCCGGTAT
GGTCGTCACCTTCGCCCCCTCCAACGTCACCACTGAGGTCAAGTCCGTGGAGATGCACC
ACGAGCAGCTCACCGAGGGTCAGCCCGGTGACAACGTTGGTTTCAACGTCAAGAACGTC
TCCGTCAAGGAGATTCGCCGTGGCAACGTCGCTGGTGACTCCAAGAACGACCCCCCCAT
GGCCGCCGCTTCCTTCAACGCTCAGGTCATCGTCCTGAACCACCCTGGCCAGGTCGGTGC
TGGATAC

>Melanconiella_chrysodiscosporina

CACACCGCCCGTCGCTACTACCGATTGAATGGTTCAGTGAGGCGTCCGGACTGGCCCAG
GGAGGTCGGCAACGACCACCCAGGGCCGGAAAGTTCTCCAAACTCGATCATTTAGAGGA
AGTAAAAGTCGTAACAAGGTCTCCGTTGGTGAACCAGCGGAGGGATCATTGCTGGAAAA
CACGTCCCATACCCCTTTGTGAACATATACCTATCTCGTTGCCTCGGCGCCAGGCCCGGG
GTGCCCCCCAGGGGGTACCCCCCACCTATGGACCTCACGGTCCCGGGCGGAGCAGGCTC
GCCGGCGGCCCCTTCCAACTCTTGTTTTTAGAACCTATCTCTTCTGAGTCTTGTTACCTAG
AAATGAATCAAAACTTTCAACAACGGATCTCTTGGCTCTGGCATCGATGAAGAACGCAG
CGAAATGCGATAAGTAATGTGAATTGCAGAATTCAGTGAACCATCGAATCTTTGAACGC
ACATTGCGCCCGCTGGGATTCCGGCGGGCATGCCTGTTCGAGCGTCATTTCAAACCCTCA
GGCGTCCGCGCCTGGTGTTGGGGCATAGCTTGTAAAAAGGCTAGCTCTTAAATTCAGTG

GCGTGCTCGCTGAGACTCCCGGGTGTAGTAATCTCCTTCTCGCCCGGGCAGGCTCGGCG
GCGCACTTGCCGTAAAACCCGCTATCACGCACTGCTTCTGAAAATTGACCTCGGATCAG
GTAGGAATACCCGCTGAACTTAAGCATATCAATAAGCGGAGGAAAAGAAACCAACAGG
GATTGCCCTAGTAACGGCGAGTGAAGCGGCAACAGCTCAAATTTGAAATCTGGTCCCTC
ACGGGGCCCGAGTTGTAATTTGCAGAGGATGTCTTCTGGCGCGGTGCCTTCCGAGTTCCC
TGGAACGGGACGCCACAGAGGGTGAGAGCCCCGTAATGGTCGGATACCAAGCCTGTGT
GAAGCTCCTTCGACGAGTCGAGTAGTTTGGGAATGCTGCTCTAAATGGGAGGTAAATCT
CTTCTAAAGCTAAATACYGGCCAGAGACCGATAGCGCACAAGTAGAGTGATCGAAAGA
TGAAAAGCACCTTGAAAAGGGGGTTAAACAGTACGTGAAATTGTTGAAAGGGAAGCGC
TCATGACCAGACTTGCGCCGGGCGGCTCATCAGGTGGTTCTGCCCCTGTGTACTCCGTCC
CGGTTCAGGCCAGCATCGGTTCGCGCCGGGGGACAAGAACGGTGGGAACGTGGCCCCC
CCTCGGGGGGGTGTTATAGCCCGCCGTGACGATACCCTGGTGCGGACCGAGGTTCGCGC
ACTCCGCAAGGATGCTGGCGTAATGGTTATCAGTGACCCGTCTTGAAACACGGACCAAG
GAGTCGTCCTTTAGAGCGAGCGTCTGGGTGTCAAAACCCGCACGCGTAATGAAAGTGAA
ATCAGGTGAGAGCCCCTCGTGGGCGCATCATCGACCGATCCTGATGTTCTCGGATGGAT
TTGAGTAAGAGTTTTAACGGACGGACCCGAAAGACAGTGAACTATGCTTGTATAGGGTG
AAGCCAGAGGAAACTCTGGTGGAGGCTCGCAGCGGTTCTGACGTGCAAATCGATCGTCA
AATATGAGCATGGGGGCGAAAGACTAATCGAACCACACCGCCCGTCGCTACTACCGATT
GAATGGTTCAGTGAGGCGTCCGGACTGGCCCAGGGAGGTCGGCAACGACCACCCAGGG
CCGGAAAGTTCTCCAAACTCGATCATTTAGAGGAAGTAAAAGTCGTAACAAGGTCTCCG
TTGGTGAACCAGCGGAGGGATCATTGCTGGAAAACACGTCCCATACCCCTTTGTGAACA
TATACCTATCTCGTTGCCTCGGCGCCAGGCCCGGGGTGCCCCCCAGGGGGTACCCCCCA
CCTATGGACCTCACGGTCCCGGGCGGAGCAGGCTCGCCGGCGGCCCCTTCCAACTCTTG
TTTTTAGAACCTATCTCTTCTGAGTCTTGTTACCTAGAAATGAATCAAAACTTTCAACAA
CGGATCTCTTGGCTCTGGCATCGATGAAGAACGCAGCGAAATGCGATAAGTAATGTGAA
TTGCAGAATTCAGTGAACCATCGAATCTTTGAACGCACATTGCGCCCGCTGGGATTCCG
GCGGGCATGCCTGTTCGAGCGTCATTTCAAACCCTCAGGCGTCCGCGCCTGGTGTTGGG
GCATAGCTTGTAAAAAGGCTAGCTCTTAAATTCAGTGGCGTGCTCGCTGAGACTCCCGG
GTGTAGTAATCTCCTTCTCGCCCGGGCAGGCTCGGCGGCGCACTTGCCGTAAAACCCGCT
ATCACGCACTGCTTCTGAAAATTGACCTCGGATCAGGTAGGAATACCCGCTGAACTTAA
GCATATCAATAAGCGGAGGAAAAGAAACCAACAGGGATTGCCCTAGTAACGGCGAGTG
AAGCGGCAACAGCTCAAATTTGAAATCTGGTCCCTCACGGGGCCCGAGTTGTAATTTGC
AGAGGATGTCTTCTGGCGCGGTGCCTTCCGAGTTCCCTGGAACGGGACGCCACAGAGGG
TGAGAGCCCCGTAATGGTCGGATACCAAGCCTGTGTGAAGCTCCTTCGACGAGTCGAGT
AGTTTGGGAATGCTGCTCTAAATGGGAGGTAAATCTCTTCTAAAGCTAAATACYGGCCA
GAGACCGATAGCGCACAAGTAGAGTGATCGAAAGATGAAAAGCACCTTGAAAAGGGGG
TTAAACAGTACGTGAAATTGTTGAAAGGGAAGCGCTCATGACCAGACTTGCGCCGGGCG
GCTCATCAGGTGGTTCTGCCCCTGTGTACTCCGTCCCGGTTCAGGCCAGCATCGGTTCGC
GCCGGGGGACAAGAACGGTGGGAACGTGGCCCCCCCTCGGGGGGGTGTTATAGCCCGC
CGTGACGATACCCTGGTGCGGACCGAGGTTCGCGCACTCCGCAAGGATGCTGGCGTAAT
GGTTATCAGTGACCCGTCTTGAAACACGGACCAAGGAGTCGTCCTTTAGAGCGAGCGTC
TGGGTGTCAAAACCCGCACGCGTAATGAAAGTGAAATCAGGTGAGAGCCCCTCGTGGGC
GCATCATCGACCGATCCTGATGTTCTCGGATGGATTTGAGTAAGAGTTTTAACGGACGG
ACCCGAAAGACAGTGAACTATGCTTGTATAGGGTGAAGCCAGAGGAAACTCTGGTGGA
GGCTCGCAGCGGTTCTGACGTGCAAATCGATCGTCAAATATGAGCATGGGGGCGAAAGA
CTAATCGAACGGTCCGCTCCTGGCAAAGCTGTTCCGAAACATCGTCAAGAAGCTGACCG
GCGACATGATGGCCTACATGAAGCGTTGTGTCAGCGAGGGTAAGCATTTTGATCTAGCG

TTGGGCATCCGTCACTCGACCCTCACGAATGGCCTCAAGTATTCTCTTGCGACTGGAAAC
TGGGGCGAGCAGAAGAAGGCGATGAGTTCCACCGCTGGTGTCTCGCAGGTGCTGAACCG
CTACACGTTCTCTTCCACCCTGTCGCATTTGCGGCGCACGAACACGCCCATCGGTCGTGA
CGGCAAGCTGGCCAAGCCTCGTCAGCTGCACAATACACACTGGGGCCTCGTCTGTCCAG
CAGAGACTCCCGAGGGACAGGCTTGCGGTCTCGTCAAGAACCTCTCTTAATGTGCTAC
GTTAGCGTTGGTTCACCAGCTGAGCCTATCAAGGACTTCATGGTGCAGCGTAATATGGA
AGTCTTGGAGGAATACGAGCCCGGCTCCAGCCCTGACGCAACCAAGATATTCATCAACG
GCACCTGGGTTGGTGTACACAGTGAACCAGCACACTTGGTGACGCTTGTGCAGGAGCTT
CGGCGCAAATGCATCATTTCTCACGAAGTCTCCCTCGTTCGCGACATTCGCGACCGCGAG
TTCAAGATCTTCTCTGACGCTGGTCGTGTCATGCGGCCACTCTTCGTTGTCAATCAAGAC
GATAACCCCGAGACTGGAGCGCAGCAGGGCACTCTAGCTCTCACCAAGCACCACATTCG
CCGTCTCGAGGAAGATGCTCAGTACCAACGCAAGAAGCATGACGACGACTATTTTGGCT
GGGATGGTCTTCAGAACAGCGGTTGTATCGAGTATCTGGATGCCGAGGAAGAGGAGAC
AGCGATGATCTGCATGAGTCCTGAGGATCTTGAGGACTACCGCCAGCGCAAGGCTAGAG
GCAAGGATGCCAAGGATGATGAACCCGAGGACGATGGCAGAAGTCTGAACGCTCGTGT
GAAGACGAAGATTAACCCCGACATCCACATGTACACCCACTGCGAGATCCATCCGAGCA
TGCTCCTGGGAATATGCGCCAGCATCATTCCCTTCCCGGATCACAATCAGGTGTGTAAAA
TTCTCGCAGTACTACTATCACTCCTGCTGACCACATATATCTAGTCACCTCGTCATCCAA
TCCAGCATCACCACATCACCATCGCACAAACATCAAGATCAAGTCCAAACGGTCGCCCT
TTCTTCCTCCCGGCCGCCTGCCCTGGCCTGCTTGGGGGGAGGGGGGGTTGCCCTTTTTC
GCCCACCACACATTTTCGCTTTGTTGTGCGGGGTTTTGCGTCTGGGTCTTATCAGATAAA
CGTGACCCACCCAACCGCCACCTCAATCATCCTCCGCTAACACACCCTTGCGCGCGCCTC
TTTTTTGCCCTCTAATTTGACAACGACAATACTGACCCGGGTTTTTACTGCAGCTGCTG
AGCTCGGCAAGGGTTCCTTCAAGTACGCATGGGTCCTTGACAAGCTCAAGGCCGAGCGT
GAGCGTGGTATCACCATCGACATTGCTCTGTGGAAGTTCGAGACTCCCAAGTACTATGTC
ACCGTCATTGGTAAGCTCTACGTACCCCCTGGCGGTTTTGCGCAGGCCTGCCTGCCCCGC
CTCTGCGCTGCCTCGCCTCTGCGCTGCCTCGCCTCTGCGCTGCCCCGCCACGCGCAACGC
CACGGCTCCTGGTGCTTTTGCGCAACCCGTGCTAACGCAAATGTCAACACAGACGCTCC
CGGTCACCGTGACTTCATCAAGAACATGATCACTGGTACCTCCCAGGCTGACTGCGCCG
TTCTCATCATTGCCTCCGGTACTGGTGAGTTCGAGGCTGGTATCTCCAAGGATGGCCAGA
CTCGTGAGCACGCTCTGCTCGCCTACACCCTCGGTGTCAAGCAGCTCATTGTTGCTTGCA
ACAAGATGGACACCGCTGGCTGGAAGCAGGACCGTTTCGAGGAGATCAAGAAGGAGAC
CACCAACTTCATCAAGAAGGTTGGCTTCAACCCCAAGCAGGTTGCTTTCGTCCCCATCTC
CGGTTTCCACGGCGACAACATGCTTGAGGCTTCCGTCAACATGCCCTGGTACAAGGGCT
GGACCAAGGAGACCCAGGACAAGAAGGAGGCCAAGGGTATGACCCTGCTCGACGCCAT
TGACTCCATCGAGCCCCCCAAGCGTCCCACCGAGAAGCCCCTGCGTCTGCCCCTCCAGG
ATGTCTACAAGATCGGCGGTATCGGAACTGTACCGGTCGGCCGTATCGAGACTGGTGTC
CTGAAGCCCGGTATGGTCGTCACCTTCGCCCCCTCCAACGTCACCACTGAGGTCAAGTCC
GTGGAGATGCACCACGAGCAGCTCACCGAGGGTCAGCCCGGTGACAACGTTGGTTTCAA
CGTCAAGAACGTCTCCGTCAAGGAGATTCGCCGTGGCAACGTCGCTGGTGACTCCAAGA
ACGACCCCCCCATGGCCGCCGCTTCCTTCAACGCTCAGGTCATCGTCCTGAACCACCCTG
GCCAGGTCGGTGCTGGATAC

>Melanconiella_chrysomelanconium

CACACCGCCCGTCGCTACTACCGATTGAATGGTTCAGTGAGGCGTCCGGACTGGCCCAG
GGAGGTCGGCAACGACCACCCAGGGCCGGAAAGCTCTCCAAACTCGATCATTTAGAGG
AAGTAAAAGTCGTAACAAGGTCTCCGTTGGTGAACCAGCGGAGGGATCATTGATGGAAT

ACACGTCCTATACCCCTTTGTGAACATATACCTATCTCGTTGCCTCGGCGCCAGGCCCGG
GGTGCCCCCCAGGGGGTACCCCCCACCTATGGACCTCACGGTCCCGGGCGGAGCAGGCC
CGCCGGCGGCCCCTTTAAACTCTTGTTTTTAGAACCTATCTCTTCTGAGTGATACAAAAA
ATGAATCAAAACTTTCAACAACGGATCTCTTGGCTCTGGCATCGATGAAGAACGCAGCG
AAATGCGATAAGTAATGTGAATTGCAGAATTCAGTGAATCATCGAATCTTTGAACGCAC
ATTGCGCCCGCTGGGATTCCGGCGGGCATGCCTGTTCGAGCGTCATTTCAAACCCCTCAG
GCGTCCGCGCCTGGTGTTGGGGCATAGCCTGTAAAAAGGCTAGCCCTTAAATTCAGTGG
CGTGCTCGCTGAGACTCCCGGGTGTAGTAATCTCCTTCTCGCCCGGGCAGGCTCAGCGGC
GCACTTGCCGTAAAACCCGCTATCACGCACCTTCTGAAAGTTGACCTCGGATCAGGTAG
GAATACCCGCTGAACTTAAGCATATCAATAAGCGGAGGAAAAGAAACCAACAGGGATT
GCCCTAGTAACGGCGAGTGAAGCGGCAACAGCTCAAATTTGAAATCTGGCCCCCCACGG
GGCCCGAGTTGTAATTTGCAGAGGATGTCTTCTGGCGCGGTGCCTTCCGAGTTCCCTGGA
ACGGGACGCCATAGAGGGTGAGAGCCCCGTAATGGTCGGATACCAAGCCTGTGTGAAG
CTCCTTCGACGAGTCGAGTAGTTTGGGAATGCTGCTCTAAATGGGAGGTAAATCTCTTCT
AAAGCTAAATACTGGCCAGAGACCGATAGCGCACAAGTAGAGTGATCGAAAGATGAAA
AGCACCTTGAAAAGGGGGTTAAACAGTACGTGAAATTGTTGAAAGGGAAGCGCTCATG
ACCAGACTTGCGCCGGGCGGCTCATCAGGTGGTTCTGCCCCTGTGTACTCCGTCCCGGTT
CAGGCCAGCATCGGTTCGCGCCGGGGGACAAGAACGGTGGGAACGTGGCCCTCCCTCG
GGGGGGTGTTATAGCCCGCCGTAACGATATCCTGGTGCGGACCGAGGTTCGCGCACTCC
GCAAGGATGCTGGCGTAATGGTTATCAGTGACCCGTCTTGAAACACGGACCAAGGAGTC
GTCCTTTAGAGCGAGCGTCTGGGTGTCAAAACCCGCACGCGTAATGAAAGTGAAATCAG
GTGAGAGCCCCTTGTGGGCGCATCATCGACCGATCCTGATGTTCTCGGATGGATTTGAGT
AAGAGTTTTAACGGACGGACCCGAAAGACAGTGAACTATGCTTGTATAGGGTGAAGCCA
GAGGAAACTCTGGTGGAGGCTCGCAGCGGTTCTGACGTGCAAATCGATCGTCAAATATG
AGCATGGGGGCGAAAGACTAATCGAACACACCGCCCGTCGCTACTACCGATTGAATGGT
TCAGTGAGGCGTCCGGACTGGCCCAGGGAGGTCGGCAACGACCACCCAGGGCCGGAAA
GCTCTCCAAACTCGATCATTTAGAGGAAGTAAAAGTCGTAACAAGGTCTCCGTTGGTGA
ACCAGCGGAGGGATCATTGATGGAATACACGTCCTATACCCCTTTGTGAACATATACCT
ATCTCGTTGCCTCGGCGCCAGGCCCGGGGTGCCCCCCAGGGGGTACCCCCCACCTATGG
ACCTCACGGTCCCGGGCGGAGCAGGCCCGCCGGCGGCCCCTTTAAACTCTTGTTTTTAGA
ACCTATCTCTTCTGAGTGATACAAAAAATGAATCAAAACTTTCAACAACGGATCTCTTGG
CTCTGGCATCGATGAAGAACGCAGCGAAATGCGATAAGTAATGTGAATTGCAGAATTCA
GTGAATCATCGAATCTTTGAACGCACATTGCGCCCGCTGGGATTCCGGCGGGCATGCCT
GTTCGAGCGTCATTTCAAACCCCTCAGGCGTCCGCGCCTGGTGTTGGGGCATAGCCTGTA
AAAAGGCTAGCCCTTAAATTCAGTGGCGTGCTCGCTGAGACTCCCGGGTGTAGTAATCT
CCTTCTCGCCCGGGCAGGCTCAGCGGCGCACTTGCCGTAAAACCCGCTATCACGCACCTT
CTGAAAGTTGACCTCGGATCAGGTAGGAATACCCGCTGAACTTAAGCATATCAATAAGC
GGAGGAAAAGAAACCAACAGGGATTGCCCTAGTAACGGCGAGTGAAGCGGCAACAGCT
CAAATTTGAAATCTGGCCCCCCACGGGGCCCGAGTTGTAATTTGCAGAGGATGTCTTCTG
GCGCGGTGCCTTCCGAGTTCCCTGGAACGGGACGCCATAGAGGGTGAGAGCCCCGTAAT
GGTCGGATACCAAGCCTGTGTGAAGCTCCTTCGACGAGTCGAGTAGTTTGGGAATGCTG
CTCTAAATGGGAGGTAAATCTCTTCTAAAGCTAAATACTGGCCAGAGACCGATAGCGCA
CAAGTAGAGTGATCGAAAGATGAAAAGCACCTTGAAAAGGGGGTTAAACAGTACGTGA
AATTGTTGAAAGGGAAGCGCTCATGACCAGACTTGCGCCGGGCGGCTCATCAGGTGGTT
CTGCCCCTGTGTACTCCGTCCCGGTTCAGGCCAGCATCGGTTCGCGCCGGGGGACAAGA
ACGGTGGGAACGTGGCCCTCCCTCGGGGGGGTGTTATAGCCCGCCGTAACGATATCCTG
GTGCGGACCGAGGTTCGCGCACTCCGCAAGGATGCTGGCGTAATGGTTATCAGTGACCC

GTCTTGAAACACGGACCAAGGAGTCGTCCTTTAGAGCGAGCGTCTGGGTGTCAAAACCC
GCACGCGTAATGAAAGTGAAATCAGGTGAGAGCCCCTTGTGGGCGCATCATCGACCGAT
CCTGATGTTCTCGGATGGATTTGAGTAAGAGTTTTAACGGACGGACCCGAAAGACAGTG
AACTATGCTTGTATAGGGTGAAGCCAGAGGAAACTCTGGTGGAGGCTCGCAGCGGTTCT
GACGTGCAAATCGATCGTCAAATATGAGCATGGGGGCGAAAGACTAATCGAAGGTCCG
CTCCTGGCAAAGCTGTTCCGAAACATCGTCAAGAAGCTGACCGGCGACATGATGGCCTA
CATGAAGCGTTGTGTCAGCGAGGGTAAGCATTTTGATCTAGCGTTGGGCATCCGTCACTC
GACCCTCACGAATGGCCTCAAGTATTCTCTTGCGACTGGAAACTGGGGCGAGCAGAAGA
AGGCGTCGAGTTCCACCGCTGGTGTCTCGCAGGTGTTGAACCGGTACACCTTCTCCTCCA
CCCTGTCGCATTTGCGGCGCACGAACACGCCCATCGGTCGTGACGGCAAGCTGGCCAAG
CCTCGTCAGCTGCACAATACACACTGGGGTCTCGTCTGTCCAGCAGAGACTCCCGAGGG
ACAGGCTTGCGGTCTCGTCAAGAACCTCTCTTTAATGTGCTACGTTAGCGTTGGTTCACC
AGCTGAGCCTATCAAGGACTTCATGGTGCAACGTAATATGGAAGTCTTGGAGGAATACG
AGCCCGGCTCCAGCCCTGACGCAACCAAGATCTTCATCAACGGCACCTGGGTTGGTGTA
CACAGTGAACCAGCACACCTGGTGACGCTTGTGCAGGAGCTTCGGCGCAAATGCATCAT
TTCTCATGAAGTCTCCCTCGTTCGCGACATTCGCGACCGCGAGTTCAAGATCTTCTCTGA
CGCTGGTCGTGTCATGCGGCCACTCTTCGTTATCAATCAAGAGGACAATCCCGAGACTG
GAGCGCAGCAGGGCACTCTAGCTCTCACCAAGCACCACATTCGCCGTCTCGAGGAAGAT
GCTCAGTACCAACGCAAGAAGCATGACGACGACTATTTTGGCTGGGATGGTCTTCAGAA
CAGCGGTTGTATCGAGTATCTGGATGCCGAGGAAGAGGAGACATCGATGATCTGCATGA
GTCCTGAGGATCTTGAGGACTACCGCCAGCGCAAGGCTAGGGGCAAGGATGCCAAGGA
TGATGAACCGGAGGACGATGGCAGAAGTCTGAACGCTCGTGTGAAGACGAAGATTAAC
CCCGACATCCACATGTACACCCACTGCGAGATCCATCCGAGCATGCTCCTGGGAATATG
CGCCAGCATCATTCCCTTCCCGGATCACAATCAGGTGTGTAAAATTCTCGCAGTACTACT
ATCACTCCTGCTGACCACATATATCTAGTCACCTCGATCATCCAATCCAGCATCACCACA
TTACCATCGCATAAGCTTCAAGATCAAGTCAAAATGGTCGCCCTCTCTTCGTCCCGGCCG
CCTGCCCTGGCCTGCTTGGGGGGACGGGGGAGGTTGCCCTTTTTTTGCCCACCACACATT
TTCGCTTTGTTGTGCGGGGTTTTGCGTCTGGGTCTTATCAGATAAACGTGACCCACCCAA
CCGCCACCTCAATCACCCTCCGCTAAGACACCCTGGCGCGCGCCTCTTTTCTACCCTCCA
ATTTGACAACGGCAATACTGACCCGGGTTTTCTACTGCAGCTGCTGAGCTCGGCAAGGG
TTCCTTCAAGTACGCATGGGTCCTTGACAAGCTCAAGGCCGAGCGTGAGCGTGGTATCA
CCATCGACATTGCTCTGTGGAAGTTCGAGACTCCCAAGTTCTATGTCACCGTCATCGGTA
AGCTCACCCCTTGGTGGTGTCGCGCAGGCCTGCCTGCCCCACCTCTCTGCGCTGCCCCGC
CACGCGCGACGCCGCGGCTCCTGGTGCTTTTATGCAACCCGTGCTGACGCAAATATCAC
CACAGACGCTCCCGGTCACCGTGACTTCATCAAGAACATGATCACTGGTACCTCTCAGG
CTGACTGCGCCATTCTCATCATTGCCTCCGGTACTGGTGAGTTCGAGGCTGGTATCTCCA
AGGATGGCCAGACCCGTGAGCACGCTCTGCTCGCCTACACCCTCGGTGTCAAGCAGCTC
ATTGTTGCTTGCAACAAGATGGACACCGCTGGCTGGAAGCAGGACCGTTTTGAGGAGAT
CAAGAAGGAGACCACCAACTTCATCAAGAAGGTCGGCTTCAACCCCAAGCAGGTCGCTT
TCGTCCCCATCTCCGGTTTCCACGGCGACAACATGCTTGAGGCCTCCCCCAACATGCCCT
GGTACAAGGGCTGGACCAAGGAGACCCAGGACAAGAAGGAGGCCAAGGGCATGACCCT
GCTCGACGCCATTGACTCCATCGAGCCCCCCAAGCGCCCCACCGAGAAGCCCCTGCGTC
TGCCCCTCCAGGATGTTTACAAGATCGGCGGTATCGGAACTGTACCGGTCGGCCGTATC
GAGACTGGTGTCCTGAAGCCCGGTATGGTCGTCACCTTCGCCCCCTCCAACGTCACCACT
GAGGTCAAGTCCGTGGAGATGCACCACGAGCAGCTCACCGAGGGTCAGCCCGGTGACA
ACGTTGGTTTCAACATCAAGAACGTCTCCGTGAAGGAGATTCGCCGTGGCAACGTCGCT
GGTGACTCCAAGAACGACCCCCCCTTGGGCGCCGCTTCCTTCAACGCTCAGGTCATTGTC
CTGAACCACCCCGGCCAGGTCGGTGCTGGATAC

>Melanconiella loropetali

GGTCTCCGTTGGTGAACCAGCGGAGGGATCATTGCTGGAACACGTCCCCCAGGGCGTAC
CCAGATACCCTTTGTGAACTTATACCTATTATCGTTGCCTCGGCGCTGGCTGGTGGGCTC
TCATGCCCACCCCTCTAGACCCTCACCGGTATAGCGGAGCAGGCCCGCCGGCGGCCCCC
TAAACTCTTGTTTTTATAACGTATCTCCTCTGAGTAACAAACAAACAAATGAATCAAAAC
TTTCAACAACGGATCTCTTGGTTCTGGCATCGATGAAGAACGCAGCGAAATGCGATAAG
TAATGTGAATTGCAGAATTCAGTGAATCATCGAATCTTTGAACGCACATTGCGCCCGCTG
GTATTCCGGCGGGCATGCCTGTTCGAGCGTCATTTCAACCCTCAAGCAGTATTCTGCTTG
GTGTTGGGGCATAGCCTGGTCAAAAGGGCTAGCCCTCAAATCTAGTGGCGTGCTCGCTA
AGACTCCGAGCGTAGTAATTTATTCTCGCTTAGGTTGGATTAGCGGTGCGTAGCCGTAAA
ACCCCCTATATTTCTGAAAGTTGACCTCGGATCAGGTAGGAATACCCGCTGAACTTAAG
CATATCCCTAGTAACGGCGAGTGAAGCGGCAACAGCTCAAATTTGAAATCTGGCCTCTT
TTGGTCCGAGTTGTAATTTGCAGAGGATGCTTCTGGCGCGGTGCCTTCCGAGTTCCCTGG
AACGGGACGCCACAGAGGGTGAGAGCCCCGTATGGTCGGACACCAAGCCTGTGTGAAG
CTCCTTCGACGAGTCGAGTAGTTTGGGAATGCTGCTCTAAATGGGAGGTAAATCTCTTCT
AAAGCTAAATACCGGCCAGAGACCGATAGCGCACAAGTAGAGTGATCGAAAGATGAAA
AGCACCTTGAAAAGGGGGTTAAACAGTACGTGAAATTGTTGAAAGGGAAGCGCTTGTG
ACCAGACTTGCGCCGGGCGGCTCATCAGGGGTTCTCCCCTGTGTATTCCGCCCGGTTCAG
GCCAGCATCGGTTCTCGCTGGGGGATAAGAACGGTAGGAACGTGGCTCCCCTCGGGGAG
TGTTATAGCCTGCCGTACGATACCCTGGCGGGGACCGAGGTTCGCGCTCCGCAAGGATG
CTGGCGTAATGGTCATCAGCGACCCGTCTTGAAACACGGACCAAGGAGTCGTCCATTAG
AGCGAGCGTTTGGGTGTAAAACCCGCACGCGTAATGAAAGTGAAATTAGGTGAGAGCTT
CGGCGCATCATCGACCGATCCTGATGTTCTCGGATGGATTTGAGTAAGAGTTTTAACGG
ACGGACCCGAAAGACAGTGAACTATGCTTGTATAGGGTGAAGCCAGAGGAAACTCTGG
TGGAGGCTCGCAGCGGTTCTGACGTGCAAATCGATCGTCAAATATGAGCATGGGGGCGA
AAGACTAATCGAACATCGTCAAGAAGTTGACAGGTGACATGATGGCATACATGAAGCG
GTGTGTCAGTGAGGGCAAGCATTTCGATCTTGCGCTGGGCATCCGTCACTCAACCCTCAC
AAATGGACTCAAGTATTCTCTCGCAACTGGAAACTGGGGTGAACAGAAGAAGGCTATGA
GCTCTACTGCTGGTGTCTCTCAGGTGTTGAACCGATACACATTCTCGTCTACTCTGTCGC
ATTTGCGGCGTACGAACACGCCCATCGGTCGTGACGGTAAGCTGGCCAAGCCTCGTCAG
CTGCACAACACGCACTGGGGCCTCGTCTGTCCAGCAGAAACGCCCGAGGGCCAGGCTTG
CGGTCTTGTCAAGAACCTGTCCCTGATGTGCTACGTCAGTGTTGGCTCACCGGCTGAGCC
CATCAAGGACTTTATGGTGCAGCGTAATATGGAGGTGTTGGAGGAATATGAGCCCGGCT
CCAGCCCCGATGCCACCAAGATCTTCATCAACGGCACGTGGGTCGGTGTCCACAACGAG
CCAGCGCACCTGGTTGGCCTGGTCCAGGAGCTTCGGCGGAAGTGCATCATCTCCCACGA
AGTCTCCCTTGTCCGCGACATTCGCGACCGCGAATTCAAGATCTTCTCCGATGCTGGGCG
TGTGATGCGCCCTTTGTTTGTTGTCAACCAAGACGACAATCCTGAGACGGGAGCGCAGC
AGGGCACCCTAGCTCTGACCAAGCATCACATCAGACGTCTCGAAGAGGATGCCCAGTAC
CATCGCAAGAAAGATGATGAGGATTATTTCGGCTGGGATGGTCTTCAGAATAGCGGCTG
CATCGAGTATCTGGATGCCGAGGAGGAGGAGACGGTGATGATCTGTATGAGCCCCGAG
GATCTTGAAGACTACCGGCAGCGCAAGGCGAGAGGCAAGGACGCCAAGGATGAAGAGC
ATGAGGATGATGGCAGGAGCCTGAACGCCCGTGTCAAGACGAAGATTAACCCTGACATT
CACATGTACACTCACTGCGAGATCCACCCGAGCATGCTCCTGGGCATTTGCGCCAGCAT
CATTCCCTTCCCGGACCACAATCAGGTATGTACAATCGCATCAGCCCCAGGCAAAGTGG
CTGCTATCTCGATGGCGGCCGTTTGCCTGGCCTGTTTGCCTGTCTTTTTCCGCCCACTGC
ACATTTTCGCTTTGTGGTGCGGGGTTTGCGTCTCGGTCTTATCTCAGATAAACGTGACCC

ACCCAACCCGCCACCTAGATCATCCTTCATCCCCGCCCCCATTCTCTCCTGACCAGAACC
GCATTCCCGCGACGACAACGGTGCTAATTCATTTCCCATCACAGCTGCTGAGCTCGGCA
AGGGTTCCTTCAAGTACGCATGGGTCCTTGACAAGCTCAAGGCCGAGCGTGAGCGTGGT
ATCACCATCGACATTGCTCTGTGGAAGTTCGAGACTCCCAAGTACTATGTCACCGTCATT
GGTAAGCTCGCTCACTCATCTCCATCTACACGCTGTGCAGACACGGCAGGCCTCGCTTAG
CGCGATACCCCGCTATGCGCGACCCCGCGGCCACAGTTATCCTGCTTGCGACACACGGC
TAACACGAAGTCCCTCCGCAGACGCTCCCGGTCACCGTGACTTCATCAAGAACATGATC
ACTGGTACCTCCCAGGCCGACTGCGCCATTCTCATCATTGCCTCCGGTACTGGTGAGTTC
GAGGCTGGTATCTCCAAGGATGGCCAGACTCGTGAGCACGCTCTGCTCGCCTACACCCT
CGGTGTCAAGCAGCTCATTGTTGCTTGCAACAAGATGGACACTGCTGGCTGGAAGCAGG
AGCGTTTCGAGGAGATCAAGAAGGAGACCACCAACTTCATCAAGAAGGTCGGCTTCAAC
CCCAAGCAGGTTGCTTTCGTCCCCATCTCCGGCTTCCACGGCGACAACATGCTGGAGGCC
TCCTCCAACATGCCCTGGTACAAGGGATGGTCCAAGGAGACCCAGGACAAGAAGGAGG
CTAAGGGTATGACCCTGCTCGACGCCATTGACGCTATCGAGCCCCCCAAGCGTCCCACC
GAGAAGCCCCTGCGTCTGCCCCTCCAGGACGTCTACAAGATCGGTGGTATTGGAACTGT
GCCCGTCGGCCGTATCGAGACTGGTGTCCTGAAGCCCGGTATGGTCGTCACCTTCGCTCC
CTCCAACGTCACCACTGAGGTCAAGTCCGTCGAGATGCACCACGAGCAGCTCACCGAGG
GTCTCCCCGGTGACAACGTTGGTTTCAACGTCAAGAACGTCTCCGTCAAGGAGATTCGC
CGTGGAAACGTCGCTGGTGACTCCAAGAACGACCCCCCTCTGGCCGCTGCCTCTTTCAAC
GCCCAGGTCATCGTCCTGAACCACCCCGGCCAGGTCG

>Melanconiella loropetali

GGTCTCCGTTGGTGAACCAGCGGAGGGATCATTGCTGGAACACGTCCCCCAGGGCGTAC
CCAGATACCCTTTGTGAACTTATACCTATTATCGTTGCCTCGGCGCTGGCTGGTGGGCTC
TCATGCCCACCCCTCTAGACCCTCACCGGTATAGCGGAGCAGGCCCGCCGGCGGCCCCC
TAAACTCTTGTTTTTATAACGTATCTCCTCTGAGTAACAAACAAACAAATGAATCAAAAC
TTTCAACAACGGATCTCTTGGTTCTGGCATCGATGAAGAACGCAGCGAAATGCGATAAG
TAATGTGAATTGCAGAATTCAGTGAATCATCGAATCTTTGAACGCACATTGCGCCCGCTG
GTATTCCGGCGGGCATGCCTGTTCGAGCGTCATTTCAACCCTCAAGCAGTATTCTGCTTG
GTGTTGGGGCATAGCCTGGTCAAAAGGGCTAGCCCTCAAATCTAGTGGCGTGCTCGCTA
AGACTCCGAGCGTAGTAATTTATTCTCGCTTAGGTTGGATTAGCGGTGCGTAGCCGTAAA
ACCCCCTATATTTCTGAAAGTTGACCTCGGATCAGGTAGGAATACCCGCTGAACTTAAG
CATATCCCTAGTAACGGCGAGTGAAGCGGCAACAGCTCAAATTTGAAATCTGGCCTCTT
TTGGTCCGAGTTGTAATTTGCAGAGGATGCTTCTGGCGCGGTGCCTTCCGAGTTCCCTGG
AACGGGACGCCACAGAGGGTGAGAGCCCCGTATGGTCGGACACCAAGCCTGTGTGAAG
CTCCTTCGACGAGTCGAGTAGTTTGGGAATGCTGCTCTAAATGGGAGGTAAATCTCTTCT
AAAGCTAAATACCGGCCAGAGACCGATAGCGCACAAGTAGAGTGATCGAAAGATGAAA
AGCACCTTGAAAAGGGGGTTAAACAGTACGTGAAATTGTTGAAAGGGAAGCGCTTGTG
ACCAGACTTGCGCCGGGCGGCTCATCAGGGGTTCTCCCCTGTGTATTCCGCCCGGTTCAG
GCCAGCATCGGTTCTCGCTGGGGGATAAGAACGGTAGGAACGTGGCTCCCCTCGGGGAG
TGTTATAGCCTGCCGTACGATACCCTGGCGGGGACCGAGGTTCGCGCTCCGCAAGGATG
CTGGCGTAATGGTCATCAGCGACCCGTCTTGAAACACGGACCAAGGAGTCGTCCATTAG
AGCGAGCGTTTGGGTGTAAAACCCGCACGCGTAATGAAAGTGAAATTAGGTGAGAGCTT
CGGCGCATCATCGACCGATCCTGATGTTCTCGGATGGATTTGAGTAAGAGTTTTAACGG
ACGGACCCGAAAGACAGTGAACTATGCTTGTATAGGGTGAAGCCAGAGGAAACTCTGG
TGGAGGCTCGCAGCGGTTCTGACGTGCAAATCGATCGTCAAATATGAGCATGGGGGCGA
AAGACTAATCGACAAGAAGTTGACAGGTGACATGATGGCATACATGAAGCGGTGTGTC

AGTGAGGGCAAGCATTTCGATCTTGCGCTGGGCATCCGTCACTCAACCCTCACAAATGG
ACTCAAGTATTCTCTCGCAACTGGAAACTGGGGTGAACAGAAGAAGGCTATGAGCTCTA
CTGCTGGTGTCTCTCAGGTGTTGAACCGATACACATTCTCGTCTACTCTGTCGCATTTGC
GGCGTACGAACACGCCCATCGGTCGTGACGGTAAGCTGGCCAAGCCTCGTCAGCTGCAC
AACACGCACTGGGGCCTCGTCTGTCCAGCAGAAACGCCCGAGGGCCAGGCTTGCGGTCT
TGTCAAGAACCTGTCCCTGATGTGCTACGTCAGTGTTGGCTCACCGGCTGAGCCCATCAA
GGACTTTATGGTGCAGCGTAATATGGAGGTGTTGGAGGAATATGAGCCCGGCTCCAGCC
CCGATGCCACCAAGATCTTCATCAACGGCACGTGGGTCGGTGTCCACAACGAGCCAGCG
CACCTGGTTGGCCTGGTCCAGGAGCTTCGGCGGAAGTGCATCATCTCCCACGAAGTCTC
CCTTGTCCGCGACATTCGCGACCGCGAATTCAAGATCTTCTCCGATGCTGGGCGTGTGAT
GCGCCCTTTGTTTGTTGTCAACCAAGACGACAATCCTGAGACGGGAGCGCAGCAGGGCA
CCCTAGCTCTGACCAAGCATCACATCAGACGTCTCGAAGAGGATGCCCAGTACCATCGC
AAGAAAGATGATGAGGATTATTTCGGCTGGGATGGTCTTCAGAATAGCGGCTGCATCGA
GTATCTGGATGCCGAGGAGGAGGAGACGGTGATGATCTGTATGAGCCCCGAGGATCTTG
AAGACTACCGGCAGCGCAAGGCGAGAGGCAAGGACGCCAAGGATGAAGAGCATGAGG
ATGATGGCAGGAGCCTGAACGCCCGTGTCAAGACGAAGATTAACCCTGACATTCACATG
TACACTCACTGCGAGATCCACCCGAGCATGCTCCTGGGCATTTGCGCCAGCATCATTCCC
TTCCCGGACCACAATCAGGTATGTACAGCCCCAGGCAAAGTGGCTGCTATCTCGATGGC
GGCCGTTTGCCTGGCCTGTTTGCCTGTCTTTTTCCGCCCACTGCACATTTTCGCTTTGTG
GTGCGGGGTTTGCGTCTCGGTCTTATCTCAGATAAACGTGACCCACCCAACCCGCCACCT
AGATCATCCTTCATCCCCGCCCCCATTCTCTCCTGACCAGAACCGCATTCCCGCGACGAC
AACGGTGCTAATTCATTTCCCATCACAGCTGCTGAGCTCGGCAAGGGTTCCTTCAAGTAC
GCATGGGTCCTTGACAAGCTCAAGGCCGAGCGTGAGCGTGGTATCACCATCGACATTGC
TCTGTGGAAGTTCGAGACTCCCAAGTACTATGTCACCGTCATTGGTAAGCTCGCTCACTC
ATCTCCATCTACACGCTGTGCAGACACGGCAGGCCTCGCTTAGCGCGATACCCCGCTAT
GCGCGACCCCGCGGCCACAGTTATCCTGCTTGCGACACACGGCTAACACGAAGTCCCTC
CGCAGACGCTCCCGGTCACCGTGACTTCATCAAGAACATGATCACTGGTACCTCCCAGG
CCGACTGCGCCATTCTCATCATTGCCTCCGGTACTGGTGAGTTCGAGGCTGGTATCTCCA
AGGATGGCCAGACTCGTGAGCACGCTCTGCTCGCCTACACCCTCGGTGTCAAGCAGCTC
ATTGTTGCTTGCAACAAGATGGACACTGCTGGCTGGAAGCAGGAGCGTTTCGAGGAGAT
CAAGAAGGAGACCACCAACTTCATCAAGAAGGTCGGCTTCAACCCCAAGCAGGTTGCTT
TCGTCCCCATCTCCGGCTTCCACGGCGACAACATGCTGGAGGCCTCCTCCAACATGCCCT
GGTACAAGGGATGGTCCAAGGAGACCCAGGACAAGAAGGAGGCTAAGGGTATGACCCT
GCTCGACGCCATTGACGCTATCGAGCCCCCCAAGCGTCCCACCGAGAAGCCCCTGCGTC
TGCCCCTCCAGGACGTCTACAAGATCGGTGGTATTGGAACTGTGCCCGTCGGCCGTATC
GAGACTGGTGTCCTGAAGCCCGGTATGGTCGTCACCTTCGCTCCCTCCAACGTCACCACT
GAGGTCAAGTCCGTCGAGATGCACCACGAGCAGCTCACCGAGGGTCTCCCCGGTGACAA
CGTTGGTTTCAACGTCAAGAACGTCTCCGTCAAGGAGATTCGCCGTGGAAACGTCGCTG
GTGACTCCAAGAACGACCCCCCTCTGGCCGCTGCCTCTTTCAACGCCCAGGTCATCGTCC
TGAACCACCCCGGCC

>Melanconiella camelliae

AGGTCTCCGTTGGTGAACCAGCGGAGGGATCATTGCTGGAAGATACCCAGAAACCCTGT
GAACATACCTGTCGTTGCCTCGGCGTTGGCTGGAGAGCCCTAAGCTCTCCCCACTGGAG
ATTTGTTTCTACAGTGGAGCAGGCTCGCCGGTGGCCCCTATAAACTCGTATTACTGTTAC
CGAAATCTTCTGAGTCCTAAAATTAAATGAATTAAAACTTTCAACAATGGATCTCTTGGC

TCTGGCATCGATGAAGAACGCAGCGAAATGCGATACGTAATGCGAATTGCAGAATTTAG
TGAATCATCGAATTTTTGAACGCACATTGCGCCCGCTGGCATTCCAGCGGGCATGCCTGT
TCGAGCGTCATTTCTCCCCTCAAATCCCCCTGTGGGCTTTGGTGTTGGGGCATAGCCTGT
TCAATGGCTAGCCCTTAAATCCAATGGCGTGCACACTGTATCACCGGGCGTAGTAATTTG
TTTCTCGCTTTGGGGGTTGTAGTGCTGCGTAGCCGTAAATTCCGGGTGTCCTCCCTTCTGT
AAAAGCAGGGGGACGCTTCTGAAATTGACCTCGGATCAGGTAGGGATACCCGCTGAACT
TAAACAGGGATTGCCTTAGTAACGGCGAGTGAAGCGGCAACAGCTCAAATTTGAAATCT
GGCCCCTAGGGTCCGAGTTGTAATTTGTAGAGGATGTTTTTGGCACGGTGCCTTCCGAGT
TCCCTGGAACGGGACGCCACAGAGGGTGAGAGCCCCGTATGGTTGGACACTAAGCCTCT
GTAAAGCTCCTTCAACGAGTCGAGTAGTTTGGGAATGCTGCTCTAAATGGGAGGTAAAT
CTCTTCTAAAGCTAAATACTGGCCAGAGACCGATAGCGCACAAGTAGAGTGATCGAAAG
ATGAAAAGCACCTTGAAAAGGGGGTTAAACAGTACGTGAAATTGTTGAAAGGGAAGCA
CTTGTCACCAGACGTGCGCCGGGCGGTTCATCAGAGGCTTTGCCCCTGTGTATGCCGTCC
GGTTCAGGCTAGCATCGGTTTTCGCTGGGGGAGAAGAACAGTTGGAACGTGGCTCGCCT
TCGGGGGAGTGTTATAGCCTTCTGTACGATGCCCTGGCGGGGACTGAGGTCCGCGCATT
TTGCAAGGATGCTGGCGTAATGGTTACCAGTGACCCGTCTTGAAACACGGACCAAGGAG
TCGTCCATTAAAGCGAGCGTCTGGGTGTGAAAACCCGTTACGCGTAATGAAAGTGAAAC
TAGGTGAGAGCTTCGGCGCATCATCGACCGATCCTGATGTTCTCGGATGGATTTGAGTA
AGAGTTTTAACGGACGGACCCGAAAGACAGTGAACTATGCTTGTATAGGGTGAAGCCAG
AGGAAACTCTGGTGGAGGCTCGCAGCGGTTCTGACGTGCAAATCGATCGTCAAATATGA
GCATGGGGGCGAAAGAGGTGACATGATGGCATACATGAAGCGGTGTGTCAGCGAGGGG
AAGCACTTCGATCTCGCACTGGGCATCCGCCACTCTACCCTTACAAATGGTCTCAAGTAC
TCACTTGCGACTGGAAACTGGGGCGAACAGAAGAAGGCGATGAGCTCTACAGCTGGCG
TCTCTCAGGTGCTGAATCGATACACTTTTGCGTCTACCCTGTCGCATTTGCGGCGCACAA
ATACGCCAATCGGCCGTGACGGGAAGCTCGCTAAGCCGCGTCAGTTGCACAACACTCAT
TGGGGTCTGGTCTGTCCAGCAGAAACTCCCGAAGGCCAGGCTTGTGGTCTTGTCAAGAA
CCTGTCTCTGATGTGCTACGTCAGTGTTGGTTCACCGGCGGAGCCCATCAAGGATTTCTT
GGTGCAGCGGAACATGGAAGTGCTGGAGGAATATGAGCCCGGCTCCAGTCCCGATGCA
ACCAAGATCTTCATCAACGGCACGTGGGTCGGTGTTCACAACGAGCCAGCGCATTTGGT
CGGCCTTATCCAAGACCTTCGACGAAAATGCATCATTTCTCACGAAGTCTCCCTTGTCCG
CGATATTCGCGATCGCGAGTTCAAGATATTCTCCGATGCTGGTCGCGTGATGCGACCCTT
GTTCGTTGTCAACCAAGATGACAATCCTCAGACGGGAGCGCAGCAAGGCACACTGGCTC
TTACCAAGCATCACATTCGGCGTCTCGAGGAAGATGCTCAGTATCATCGCAAGAAGGAC
GATGATGACTATTTCGGCTGGGATGGTCTTCAGACTAACGGCTGTATCGAGTACCTGGAT
GCCGAGGAAGAGGAGACGGTAATGATCTGTATGAGCCCGGAGGATCTCGAGGACTACC
GACAGCGGAAGGCTAGAGGCAAGGATGCAAAGGATGAAGAGCCTGAAGACGACGGCA
GGAGCCTTAACGCTCGTGTCAAAACCAAGATCAACCCTGACATTCACATGTACACCCAC
TGCGAGATCCATCCGAGCATGCTCCTGGGCATCTGCGCCAGCATCATTCCCTTCCCAGAT
CACAACCAGGTATGTACATGTCTTGCAGTTCGCACAAACACCAATCTCAAATGGCCGCG
ATAAAAACATCGCGACCGTTTGCCTGGTTCTTCCTGTCTGTCCTTTTTTCCACCCACCACA
CATTTTCGCTTTGTGGTGCGGGGTTTTGCGTCTCGGTCTTATCCAGATAAACGTGACCCA
CCCGACCGCCACCTAAGTCATCCTACGCAAACACCCATGCTCTCCTCCCTAAGAACAAA
ATTATCAGCGACGATGATACTAATTCAATTCCCATCACAGCTGCTGAGCTCGGCAAGGG
TTCCTTCAAGTACGCATGGGTCCTTGACAAGCTCAAGGCCGAGCGTGAGCGTGGTATCA
CCATCGACATTGCTCTGTGGAAGTTCGAGACTCCCAAGTACTATGTCACCGTCATTGGTA
AGTCCTCACATCCACCGCCGTTGTCGCGCAGGCCCGGTCGCCCCGCTTAGCGTGTGCTGC
CCCGCCACGTTCGACCCCGCGGCAAAATCGAGACCTCTCCGCGACCCATTGCTAACATG
CACATCACCACAGACGCTCCTGGTCACCGTGATTTCATCAAGAACATGATCACTGGTAC

CTCCCAGGCTGACTGCGCCGTTCTCATCATTGCCTCCGGTACTGGTGAGTTCGAGGCTGG
TATCTCCAAGGATGGCCAGACCCGTGAGCACGCTCTCCTCGCCTACACCCTCGGTGTCAA
GCAGCTCATTGTTGCTTGCAACAAGATGGACACCGCTGGCTGGAAGCAGGAGCGTTTCG
AGGAGATCAAGAAGGAGACGACCAACTTCATCAAGAAGGTCGGCTTCAACCCCAAGCA
GGTTGCTTTTGTCCCCATCTCTGGTTTCCACGGCGACAACATGCTTGAGGCCTCCGCCAA
CATGCCCTGGTACAAGGGCTGGACCAAGGAGACTCAGGACAAGAAGGAGGCCAAGGGC
ATGACCCTGCTCGACGCCATTGACGCCATCGAGCCCCCCAAGCGTCCCACCGAGAAGCC
CCTGCGTCTGCCCCTCCAGGACGTCTACAAGATCGGTGGTATTGGAACTGTGCCCGTCGG
CCGTATCGAGACTGGTGTCCTCAAGCCCGGTATGGTCGTTACCTTCGCTCCCTCCAACGT
CACCACTGAGGTCAAGTCCGTCGAGATGCACCACGAGCAGCTTACCGAGGGTCTCCCCG
GTGACAACGTTGGTTTCAACGTCAAGAACGTCTCCGTCAAGGAGATTCGCCGTGGCAAC
GTCGCTGGTGACTCCAAGAACGACCCCCCACTGGGTGCCGCTTCTTTCAACGCCCAGGTC
ATCGTC

>Melanconiella camelliae

CTGTGAACATACCTGTCGTTGCCTCGGCGTTGGCTGGAGAGCCCTAAGCTCTCCCCACTG
GAGATTTGTTTCTACAGTGGAGCAGGCTCGCCGGTGGCCCCTATAAACTCGTATTACTGT
TACCGAAATCTTCTGAGTCCTAAAATTAAATGAATTAAAACTTTCAACAATGGATCTCTT
GGCTCTGGCATCGATGAAGAACGCAGCGAAATGCGATACGTAATGCGAATTGCAGAATT
TAGTGAATCATCGAATTTTTGAACGCACATTGCGCCCGCTGGCATTCCAGCGGGCATGCC
TGTTCGAGCGTCATTTCTCCCCTCAAATCCCCCTGTGGGCTTTGGTGTTGGGGCATAGCC
TGTTCAATGGCTAGCCCTTAAATCCAATGGCGTGCACACTGTATCACCGGGCGTAGTAAT
TTGTTTCTCGCTTTGGGGGTTGTAGTGCTGCGTAGCCGTAAATTCCGGGTGTCCTCCCTTC
TGTAGGGATTGCCTTAGTAACGGCGAGTGAAGCGGCAACAGCTCAAATTTGAAATCTGG
CCCCTAGGGTCCGAGTTGTAATTTGTAGAGGATGTTTTTGGCACGGTGCCTTCCGAGTTC
CCTGGAACGGGACGCCACAGAGGGTGAGAGCCCCGTATGGTTGGACACTAAGCCTCTGT
AAAGCTCCTTCAACGAGTCGAGTAGTTTGGGAATGCTGCTCTAAATGGGAGGTAAATCT
CTTCTAAAGCTAAATACTGGCCAGAGACCGATAGCGCACAAGTAGAGTGATCGAAAGAT
GAAAAGCACCTTGAAAAGGGGGTTAAACAGTACGTGAAATTGTTGAAAGGGAAGCACT
TGTCACCAGACGTGCGCCGGGCGGTTCATCAGAGGCTTTGCCCCTGTGTATGCCGTCCGG
TTCAGGCTAGCATCGGTTTTCGCTGGGGGAGAAGAACAGTTGGAACGTGGCTCGCCTTC
GGGGGAGTGTTATAGCCTTCTGTACGATGCCCTGGCGGGGACTGAGGTCCGCGCATTTT
GCAAGGATGCTGGCGTAATGGTTACCAGTGACCCGTCTTGAAACACGGACCAAGGAGTC
GTCCATTAAAGCGAGCGTCTGGGTGTGAAAACCCGTTACGCGTAATGAAAGTGAAACTA
GGTGAGAGCTTCGGCGCATCATCGACCGATCCTGATGTTCTCGGATGGATTTGAGTAAG
AGTTTTAACGGACGGACCCGAAAGACAGTGAACTATGCTTGTATAGGGTGAAGCCAGAG
GAAACTCTGGTGGAGGCTCGCAGCGGTTCTGACGTGCAAATCGATCGTCAAATATGAGC
ATGGGGGCGAAAGACTAGCTCTTGGCAAGCTCTTCCGGAACATCGTCAAGAAGCTGACT
GGTGACATGATGGCATACATGAAGCGGTGTGTCAGCGAGGGGAAGCACTTCGATCTCGC
ACTGGGCATCCGCCACTCTACCCTTACAAATGGTCTCAAGTACTCACTTGCGACTGGAAA
CTGGGGCGAACAGAAGAAGGCGATGAGCTCTACAGCTGGCGTCTCTCAGGTGCTGAATC
GATACACTTTTGCGTCTACCCTGTCGCATTTGCGGCGCACAAATACGCCAATCGGCCGTG
ACGGGAAGCTCGCTAAGCCGCGTCAGTTGCACAACACTCATTGGGGTCTGGTCTGTCCA
GCAGAAACTCCCGAAGGCCAGGCTTGTGGTCTTGTCAAGAACCTGTCTCTGATGTGCTA
CGTCAGTGTTGGTTCACCGGCGGAGCCCATCAAGGATTTCTTGGTGCAGCGGAACATGG
AAGTGCTGGAGGAATATGAGCCCGGCTCCAGTCCCGATGCAACCAAGATCTTCATCAAC

GGCACGTGGGTCGGTGTTCACAACGAGCCAGCGCATTTGGTCGGCCTTATCCAAGACCT
TCGACGAAAATGCATCATTTCTCACGAAGTCTCCCTTGTCCGCGATATTCGCGATCGCGA
GTTCAAGATATTCTCCGATGCTGGTCGCGTGATGCGACCCTTGTTCGTTGTCAACCAAGA
TGACAATCCTCAGACGGGAGCGCAGCAAGGCACACTGGCTCTTACCAAGCATCACATTC
GGCGTCTCGAGGAAGATGCTCAGTATCATCGCAAGAAGGACGATGATGACTATTTCGGC
TGGGATGGTCTTCAGACTAACGGCTGGATCGAGTACCTGGATGCCCAAGAAAAAGAAG
ACGGTAATGATCTGTATGAGCCCGGAGGATCTCGAGGACTACCGACAGCGGAAGGCTA
GAGGCAAGGATGCAAAGGATGAAGAGCCTGAAGACGACGGCAGGAGCCTTAACGCTCG
TGTCAAAACCAAGATCAACCCTGACATTCACATGTACACCCACTGCGAGATCCATCCGA
GCATGCTCCTGGGCATCTGCGCCAGCATCATTCCCTTCCCAGATCACAACCAGGTATGTA
CATGTCTTGCAGTACTAACATTGCTCCGCACAAACACCAATCTCAAATGGCCGCGATAA
AAACATCGCGACCGTTTGCCTGGTTCTTCCTGTCTGTCCTTTTTTCCACCCACCACACATT
TTCGCTTTGTGGTGCGGGGTTTTGCGTCTCGGTCTTATCCAGATAAACGTGACCCACCCG
ACCGCCACCTAAGTCATCCTACGCAAACACCCATGCTCTCCTCCCTAAGAACAAAATTAT
CAGCGACGATGATACTAATTCAATTCCCATCACAGCTGCTGAGCTCGGCAAGGGTTCCTT
CAAGTACGCATGGGTCCTTGACAAGCTCAAGGCCGAGCGTGAGCGTGGTATCACCATCG
ACATTGCTCTGTGGAAGTTCGAGACTCCCAAGTACTATGTCACCGTCATTGGTAAGTCCT
CACATCCACCGCCGTTGTCGCGCAGGCCCGGTCGCCCCGCTTAGCGTGTGCTGCCCCGCC
ACGTTCGACCCCGCGGCAAAATCGAGACCTCTCCGCGACCCATTGCTAACATGCACATC
ACCACAGACGCTCCTGGTCACCGTGATTTCATCAAGAACATGATCACTGGTACCTCCCA
GGCTGACTGCGCCGTTCTCATCATTGCCTCCGGTACTGGTGAGTTCGAGGCTGGTATCTC
CAAGGATGGCCAGACCCGTGAGCACGCTCTCCTCGCCTACACCCTCGGTGTCAAGCAGC
TCATTGTTGCTTGCAACAAGATGGACACCGCTGGCTGGAAGCAGGAGCGTTTCGAGGAG
ATCAAGAAGGAGACGACCAACTTCATCAAGAAGGTCGGCTTCAACCCCAAGCAGGTTGC
TTTTGTCCCCATCTCTGGTTTCCACGGCGACAACATGCTTGAGGCCTCCGCCAACATGCC
CTGGTACAAGGGCTGGACCAAGGAGACTCAGGACAAGAAGGAGGCCAAGGGCATGACC
CTGCTCGACGCCATTGACGCCATCGAGCCCCCCAAGCGTCCCACCGAGAAGCCCCTGCG
TCTGCCCCTCCAGGACGTCTACAAGATCGGTGGTATTGGAACTGTGCCCGTCGGCCGTAT
CGAGACTGGTGTCCTCAAGCCCGGTATGGTCGTTACCTTCGCTCCCTCCAACGTCACCAC
TGAGGTCAAGTCCGTCGAGATGCACCACGAGCAGCTTACCGAGGGTCTCCCCGGTGACA
ACGTTGGTTTCAACGTCAAGAACGTCTCCGTCAAGGAGATTCGCCGTGGCAACGTCGCT
GGTGACTCCAAGAACGACCCCCCACTGGGTGCCGCTTCTTTCAACGCCCAGGTCATCGTC
C
